# Dietary Risk-Related Colorectal Cancer Burden: Estimates From 1990 to 2019

**DOI:** 10.3389/fnut.2021.690663

**Published:** 2021-08-24

**Authors:** Yujiao Deng, Bajin Wei, Zhen Zhai, Yi Zheng, Jia Yao, Shuqian Wang, Dong Xiang, Jingjing Hu, Xianghua Ye, Si Yang, Ying Wu, Na Li, Peng Xu, Jun Lyu, Zhijun Dai

**Affiliations:** ^1^Department of Breast Surgery, The First Affiliated Hospital, College of Medicine, Zhejiang University, Hangzhou, China; ^2^Department of Oncology, The Second Affiliated Hospital of Xi'an Jiaotong University, Xi'an, China; ^3^Department of Hepatobiliary and Pancreatic Surgery, The First Affiliated Hospital, College of Medicine, Zhejiang University, Hangzhou, China; ^4^Celilo Cancer Center, Oregon Health Science Center Affiliated Mid-Columbia Medical Center, The Dalles, OR, United States; ^5^Dana-Farber Cancer Institute, Harvard Medical School, Boston, MA, United States; ^6^Department of Radiotherapy, The First Affiliated Hospital, College of Medicine, Zhejiang University, Hangzhou, China; ^7^Department of Clinical Research, The First Affiliated Hospital of Jinan University, Guangzhou, China

**Keywords:** colorectal cancer, nutrient, dietary risk, death, disability-adjusted life- years

## Abstract

**Background:** Colorectal cancer remains a public health problem worldwide. Dietary risk factors play a key role in the carcinogenesis and progression of colorectal cancer. This study aimed to explore the geographical and temporal trends in various dietary factor-related colorectal cancers.

**Methods:** Data were extracted from the Global Burden of Disease (GBD) 2019 study, including the deaths, disability-adjusted life-years (DALYs), age-standardized rate (ASR), and summary exposure value (SEV) among 4 world regions, 11 age groups, 21 regions, and 204 countries and territories between 1990 and 2019. The estimated annual percentage changes (EAPCs) were calculated to evaluate the variation trend of ASR.

**Results:** Dietary factors were the leading cause of colorectal cancer death and DALY rate, regardless of age. Dietary factor-related deaths and DALYs accounted for 32 and 34% of global colorectal cancer, respectively. Further analysis showed that low whole grain intake remained the leading cause of cancer death and DALY rate, followed by milk and calcium. Diets that were low in whole grains, milk, and calcium accounted for 81.61% of deaths and 81.64% of DALYs. Deaths and DALYs of dietary factors related to colorectal cancer grew by half from 1990 to 2019. All ASRs remained higher for men than women. Asia carried the highest colorectal cancer burden attributed to dietary risks, especially for East Asia [age-standardized death rate (ASDR): EAPC = 1.15, 95% CI:0.88–1.42; DALY: EAPC = 1.08, 95% CI:0.82–1.34]. The heavy burden also existed in high-middle and middle socio-demographic index (SDI) quintiles. China has always had the highest deaths and DALYs of colorectal cancer attributable to dietary risks, followed by the USA, India, and Japan.

**Conclusions:** Large variations existed in the dietary risk-related colorectal cancer burdens among sexes, regions, and countries. More targeted interventions to address modifiable dietary risk factors would save 32% of deaths and 34% of DALYs for colorectal cancer.

## Introduction

In 2019, colorectal cancer accounted for more than 24.28 million disability-adjusted life-years (DALYs) and 1.15 million deaths worldwide (1). It was also the second leading cause of cancer deaths. Modifiable risk factors, such as environmental, behavioral, and metabolic risks, played a key role in the carcinogenesis and progression of colorectal cancer, which are potentially preventable (2). Epidemiological and experimental investigations have also demonstrated an association between nutritional intake and the risk of colorectal cancer. Calcium, fiber, milk, and whole grains might reduce this risk, while red and processed meats increased the risk (3). Furthermore, diet can influence the development of colorectal cancer through a variety of interaction mechanisms, including influences on immune reactivity, inflammation, and intestinal microbiota (4). In 2019, dietary factors were the second leading cause of death in women (3.48 million deaths) and the third leading cause of death in men (4.47 million deaths) (2). It was reported that 47% of colorectal cancer cases in the USA and 45% in the UK were attributable to modifiable risk factors (5, 6). However, the distribution of these risk factors varies in different countries and regions, with the influence degree of the same risk factor on the population of different regions and genders being different. Cancer-specific reports support more detailed explorations and provide useful information for colorectal cancer prevention, treatment, and management (7).

The Global Burden of Disease (GBD) 2019 study carried a broad array of data sources and scientific statistical modeling approaches and included up-to-date data of 369 diseases and injuries and their 87 related risk factors globally (8). Therefore, this systematic investigation of dietary consumption patterns of colorectal patients was conducted to provide a comprehensive illustration of prognostic effects of unhealthy dietary habits at the global level.

## Materials and Methods

### Data Sources

Dietary factor-related colorectal cancer deaths and DALY data were collected by the GBD 2019 study using Global Heath Data Exchange Tool (http://ghdx.healthdata.org/). Deaths, DALYs, age-standardized rates (ASRs), summary exposure values (SEV), and annualized rates of change (ARC, %) and their 95% uncertainty intervals (UIs) from 1990 to 2019 among 4 world regions, 5 socio-demographic index (SDI) quintiles, 21 regions, and 204 countries and territories were extracted. The SDI, ranging from 0 to 1, is a comprehensive measure of development and is an indicator of the overall fertility rate, educational attainment, and lagging per capita income distribution in a country. Based on SDI values in 2019, countries and territories were classified into five categories (high, high-middle, middle, low-middle, and low). All the rates were reported per 100,000 persons. The analysis of this study is in accordance with the Guidelines for Accurate and Transparent Health Estimates Reporting (GATHER) (Appendix 1). Detailed data descriptions and search strategies are presented in Appendix 2.

### Exposure Definition

Six dietary risk factors were evaluated in this analysis, including diets low in fiber, high in processed meat, high in red meat, low in calcium, low in milk, and low in whole grains. Diets low in whole grains represent an average daily consumption (in g per day) of less than 140–160 g of whole grains (germ, endosperm, and bran in the natural proportion) from biscuits, pancakes, bread, breakfast cereals, tortillas, rice, muffins, pasta, and other sources. Diets low in milk represent an average daily intake (in grams per day) of less than 360–500 g of milk including full-fat, low-fat, and non-fat milk, excluding soy milk and other plant derivatives. Diets high in red meat represent any intake (in g per day) of red meat including lamb, pork, beef, and goat but excluding eggs, fish, poultry, and all processed meats. Diets high in processed meat represent any intake (in g per day) of meat preserved by curing, smoking, salting, or addition of chemical preservatives. Diets low in fiber represent an average daily consumption (in g per day) of less than 21–22 g of fiber from all sources including grains, fruits, pulses, vegetables, and legumes. Diets low in calcium represent an average daily consumption (in g per day) of less than 1.06–1.1 g of calcium from all sources, including cheese, milk, and yogurt.

### Measures Estimation

The ASR is a measure that can eliminate the influence of population age structure difference to the greatest extent, which was calculated based on the world standard population. The ASR was calculated based on the following formula:

$$\begin{array}{l} ASR=\frac{\overset{A}{\underset{i=1}{\sum }}a_{i}w_{i}}{\overset{A}{\underset{i=1}{\sum }}w_{i}}\times 100,000 \end{array}$$

The ASR (per 100,000 population) is equal to the sum of the product of the specific age ratio (ai) in age group i and the number (or weight) (wi) of the selected reference standard population group i divided by the sum of the number (or weight) of the standard population (i.e., the weight is derived from the GBD 2019 world standard population rather than the global population size in 2019).

Meanwhile, the estimated annual percentage changes (EAPC) were calculated to evaluate the variation trend of ASR based on our previous methods (9). The EAPC, which is approximately equal to the annual change over a specified time period, was calculated using the following regression model to assess the trends in ASR: Y = α + βx + ε, where y refers to ln (ASR), x represents calendar year, ε means error term, and β determines the positive or negative trends in ASR.

The EAPC is calculated as 100 * [exp (β) – 1]. Its 95% CI can be obtained from the linear regression model. If both the EAPC estimates and their lower limits of 95% CIs are more than 0, ASR is in an upward trend. In contrast, if both EAPC estimates and their upper limits of 95% CIs are less than 0, ASR is in a downward trend. Otherwise, ASR is stable.

### Statistical Analysis

The GBD 2019 study used SEV to summarize the exposure distribution of dichotomous, multi-categorical, and continuous risk factors. The summary exposure value is a univariate measure of risk-weighted exposure with a range from 0 to 100%, which is 0 when the population is at no additional risk and 1 when the population is at the highest risk level. A decrease in SEV indicates a decrease in exposure to a particular risk factor, while an increase in SEV indicates an increase in exposure. DALYs were estimated by summing up the years lived with disability (YLDs) and years of life lost (YLLs). Four sequelae including diagnosis or treatment, remission, metastatic disseminate, and terminal phase were used to estimate YLDs. Then, YLDs were calculated by each sequela prevalence multiplied by a disability weight. The YLLs were estimated by multiplying the number of deaths in the age of patients with the standard life expectancy for that age. Data were presented as values with a 95% CI or 95% UI. The main analysis tools covered the Spatiotemporal Gaussian Process Regression and Dismod-MR 2.1. All tests and calculations were conducted by the R program (version 3.7.0).

## Results

### Summary Exposure Value and Risk-Specific Trends

The age-standardized SEV rate of dietary risks among humans decreased globally (ARC = −0.08, 95% UI: −0.14 to 0.04), from 51.31 (95% UI: 40.44−62.42) in 1990 to 47.1 per 100,000 persons (95% UI: 35.39−59.62) in 2019. At the regional level, East Asia has always had the highest age-standardized SEV rate (80.99 in 1990 and 76.33 in 2019), followed by Central Europe and the high-income Asia Pacific (Table 1). It increased the fastest in high-income North America (ARC = 0.15, 95% UI: 0.03–0.3) but decreased the fastest in high-income Asia Pacific (ARC = −0.28, 95% UI: −0.42 to 0.17). In addition, the SDI quintile with the highest age-standardized SEV rate changed from middle SDI (ASR = 59.93, 95% UI: 49.6–68.46) into high-middle SDI (ASR = 55.03, 95% UI: 44.56–64.09), while it remained the lowest in the low SDI quintile. China carried the highest age-standardized SEV rate of dietary risk in 2019 (77.37, 69.58–81.93), especially for women. As for men, Hungary had the top exposure (82.2, 73.79–87.31, Supplementary Table 1).

**Table 1:** Age-standardized summary exposure value rates of dietary risks related to colorectal cancer and annualized rate of changes.

**Location**	**Sex**	**Age-standardized SEV rate (95% UI)**		**ARC (%) (95% UI)**		
		**1990**	**2019**	**1990–2010**	**2010–2019**	**1990–2019**
Global	Both	51.31 (40.44–62.42)	47.1 (35.39–59.62)	−0.06 (−0.1–0.03)	−0.02 (−0.04–0.01)	−0.08 (−0.14–0.04)
	Female	48.52 (38.08–60.29)	43.53 (32.43–56.78)	−0.08 (−0.12–0.04)	−0.03 (−0.05–0.01)	−0.1 (−0.16–0.05)
	Male	54.21 (43.11–64.75)	50.78 (38.2–62.65)	−0.04 (−0.09–0.02)	−0.02 (−0.04–0.01)	−0.06 (−0.12–0.03)
**Sociodemographic Index**						
High SDI	Both	43.59 (33.19–55.53)	41.11 (28.52–54.36)	−0.05 (−0.13–0)	−0.01 (−0.03–0.01)	−0.06 (−0.15–0)
	Female	40.93 (31.33–53.31)	36.13 (26.05–49.54)	−0.11 (−0.17–0.05)	−0.01 (−0.03–0.01)	−0.12 (−0.19–−0.05)
	Male	46.36 (35.27–57.83)	46 (30.89–59.21)	0 (−0.11–0.07)	−0.01 (−0.04–0.02)	−0.01 (−0.13–0.06)
High–middle SDI	Both	56.53 (46.59–65.64)	55.03 (44.56–64.09)	−0.03 (−0.06–0.01)	0 (−0.01–0.02)	−0.03 (−0.06–0)
	Female	51.96 (42.75–61.87)	50.18 (40.43–60.01)	−0.04 (−0.07–0.01)	0 (−0.02–0.02)	−0.03 (−0.08–0)
	Male	61.49 (50.72–69.89)	60.06 (49.12–68.54)	−0.03 (−0.06–0)	0 (−0.01–0.02)	−0.02 (−0.06–0.01)
Low SDI	Both	37.34 (23.04–59.49)	35.02 (22.2–57.69)	−0.05 (−0.11–0)	−0.01 (−0.03–0)	−0.06 (−0.13–0.01)
	Female	37.44 (22.61–59.8)	34.8 (21.63–58.65)	−0.06 (−0.13–0.01)	−0.01 (−0.03–0.01)	−0.07 (−0.15–0.01)
	Male	37.24 (23.14–59.09)	35.26 (22.87–56.95)	−0.04 (−0.1–0.02)	−0.01 (−0.04–0.02)	−0.05 (−0.12–0.02)
Low–middle SDI	Both	44.59 (32.17–58.16)	42.49 (28.81–57.02)	−0.01 (−0.05–0.01)	−0.04 (−0.07–0.01)	−0.05 (−0.11–0.01)
	Female	42.55 (30.37–56.98)	39.15 (26.12–55.07)	−0.03 (−0.07–0.01)	−0.05 (−0.1–0.01)	−0.08 (−0.15–0.03)
	Male	46.55 (33.84–59.44)	45.91 (31.67–59.41)	0.01 (−0.04–0.04)	−0.02 (−0.05–0)	−0.01 (−0.07–0.02)
Middle SDI	Both	59.93 (49.6–68.46)	51.28 (39.84–61.83)	−0.11 (−0.16–0.07)	−0.04 (−0.06–0.02)	−0.14 (−0.21–0.09)
	Female	57.31 (46.72–66.69)	48.15 (36.93–59.31)	−0.12 (−0.18–0.08)	−0.04 (−0.07–0.02)	−0.16 (−0.23–0.1)
	Male	62.56 (52.55–70.53)	54.52 (42.45–64.62)	−0.1 (−0.15–0.05)	−0.04 (−0.07–0.01)	−0.13 (−0.2–0.07)
**Region**						
Andean Latin America	Both	34.85 (20.33–50.29)	33.57 (19.65–49)	−0.04 (−0.11–0.03)	0 (−0.05–0.04)	−0.04 (−0.13–0.04)
	Female	30.66 (18.96–46.79)	28.67 (17.36–43.96)	−0.06 (−0.14–0.01)	−0.01 (−0.06–0.04)	−0.06 (−0.16–0.01)
	Male	39.19 (21.36–54.5)	38.69 (20.97–54.56)	−0.02 (−0.13–0.09)	0.01 (−0.06–0.08)	−0.01 (−0.15–0.12)
Australasia	Both	37.52 (27.78–51)	36.01 (26.13–50.6)	−0.04 (−0.13–0.03)	0 (−0.05–0.04)	−0.04 (−0.14–0.04)
	Female	32.83 (25.18–45.45)	31.92 (23.79–45.38)	−0.02 (−0.08–0.05)	−0.01 (−0.05–0.04)	−0.03 (−0.1–0.06)
	Male	42.22 (28.54–57.53)	40.26 (26.54–56.91)	−0.05 (−0.18–0.07)	0 (−0.08–0.08)	−0.05 (−0.2–0.1)
Caribbean	Both	25.66 (16.87–39.73)	24.11 (15.38–38.5)	−0.07 (−0.12–0.02)	0.01 (−0.02–0.04)	−0.06 (−0.12–0)
	Female	23.09 (15.35–36.12)	21.4 (13.96–34.93)	−0.08 (−0.12–0.02)	0 (−0.03–0.04)	−0.07 (−0.13–0)
	Male	28.39 (17.38–43.83)	26.99 (15.74–42.52)	−0.06 (−0.13–0.01)	0.01 (−0.03–0.06)	−0.05 (−0.13–0.04)
Central Asia	Both	52.7 (39.2–65.64)	39.09 (25.51–54.89)	−0.25 (−0.35–0.15)	−0.02 (−0.05–0.02)	−0.26 (−0.37–0.16)
	Female	43.02 (29.72–58.75)	31.31 (21.09–46.88)	−0.26 (−0.36–0.17)	−0.02 (−0.05–0.01)	−0.27 (−0.38–0.18)
	Male	63.58 (49.64–74.23)	47.6 (29.71–63.67)	−0.24 (−0.41–0.13)	−0.01 (−0.07–0.03)	−0.25 (−0.43–0.13)
Central Europe	Both	67.6 (56–75.98)	59.85 (44.49–71.19)	−0.1 (−0.2–0.04)	−0.01 (−0.04–0)	−0.11 (−0.22–0.05)
	Female	59.12 (44.86–71.08)	46.08 (27.5–62.54)	−0.19 (−0.35–0.08)	−0.04 (−0.08–0.01)	−0.22 (−0.39–0.1)
	Male	76.48 (67.73–81.72)	74.02 (61.69–81.05)	−0.03 (−0.11–0.01)	0 (−0.03–0.03)	−0.03 (−0.11–0.01)
Central Latin America	Both	40.57 (25.34–55.43)	40.17 (25.06–55.13)	0.01 (−0.04–0.1)	−0.02 (−0.08–0.02)	−0.01 (−0.07–0.06)
	Female	34.81 (20.25–50.86)	34.23 (19.85–50.49)	0.04 (−0.05–0.17)	−0.05 (−0.16–0.02)	−0.02 (−0.09–0.06)
	Male	46.68 (30.43–60.42)	46.83 (30.35–60.67)	0 (−0.08–0.08)	0.01 (−0.04–0.06)	0 (−0.08–0.11)
Central Sub–Saharan Africa	Both	26.55 (17.71–48.61)	28.18 (18.82–52.39)	0.08 (−0.01–0.17)	−0.01 (−0.07–0.04)	0.06 (−0.03–0.17)
	Female	26.09 (17.37–47.7)	28.18 (18.8–51.84)	0.09 (−0.03–0.21)	−0.01 (−0.1–0.06)	0.08 (−0.05–0.22)
	Male	27.04 (17.75–49.98)	28.21 (18.75–52.7)	0.06 (−0.06–0.18)	−0.02 (−0.08–0.05)	0.04 (−0.08–0.18)
East Asia	Both	80.99 (74.41–84.33)	76.33 (68.25–81.08)	−0.05 (−0.08–0.01)	−0.01 (−0.03–0)	−0.06 (−0.1–0.02)
	Female	79.15 (71.96–83.25)	74 (64.71–79.75)	−0.05 (−0.11–0.01)	−0.02 (−0.05–0.01)	−0.07 (−0.13–0.02)
	Male	82.96 (76.74–86.27)	78.78 (71.59–83.12)	−0.04 (−0.08–0.01)	−0.01 (−0.03–0.01)	−0.05 (−0.09–0.01)
Eastern Europe	Both	43.15 (30.05–58.2)	39.79 (26.46–55.74)	−0.08 (−0.16–0.01)	0 (−0.05–0.04)	−0.08 (−0.18–0)
	Female	37.28 (26.46–52.94)	33.83 (22.53–50.22)	−0.09 (−0.18–0.02)	0 (−0.06–0.05)	−0.09 (−0.2–0)
	Male	50.21 (33.45–66.06)	46.78 (30.02–63.07)	−0.07 (−0.18–0.04)	0 (−0.07–0.07)	−0.07 (−0.2–0.06)
Eastern Sub–Saharan Africa	Both	46.86 (24.44–75.94)	36.02 (20.74–65.92)	−0.21 (−0.33–0.09)	−0.02 (−0.05–0.01)	−0.23 (−0.35–0.1)
	Female	49.79 (24.05–80.2)	40.23 (20.7–74.68)	−0.19 (−0.32–0.06)	0 (−0.04–0.02)	−0.19 (−0.32–0.07)
	Male	43.75 (24.71–71.33)	31.58 (20.38–57.02)	−0.24 (−0.36–0.09)	−0.05 (−0.1–0.02)	−0.28 (−0.41–0.1)
High–income Asia Pacific	Both	66.23 (53.14–74.48)	48 (31.29–61.12)	−0.25 (−0.39–0.15)	−0.03 (−0.07–0)	−0.28 (−0.42–0.17)
	Female	61.7 (46.29–72.14)	42.07 (25.57–56.85)	−0.29 (−0.44–0.18)	−0.03 (−0.08–0.01)	−0.32 (−0.47–0.2)
	Male	70.97 (59.42–77.39)	53.83 (36.51–66.01)	−0.21 (−0.37–0.12)	−0.04 (−0.09–0.01)	−0.24 (−0.4–0.13)
High–income North America	Both	34.19 (24.34–48.18)	39.48 (26.2–53.68)	0.16 (0.04–0.29)	−0.01 (−0.05–0.04)	0.15 (0.03–0.3)
	Female	34.64 (24.21–49.58)	34.06 (23.53–48.92)	−0.02 (−0.08–0.06)	0 (−0.05–0.05)	−0.02 (−0.1–0.08)
	Male	33.76 (24.58–46.87)	45.08 (28.34–59.42)	0.35 (0.11–0.57)	−0.01 (−0.08–0.05)	0.34 (0.09–0.57)
North Africa and Middle East	Both	20.72 (14.91–30.26)	19.53 (13.84–29.24)	−0.07 (−0.12–0.04)	0.02 (−0.01–0.04)	−0.06 (−0.11–0.01)
	Female	19.45(14.45–26.62)	17.75(13.09–24.64)	−0.1(−0.15–0.05)	0.01(−0.03–0.06)	−0.09(−0.14–0.04)
	Male	21.92(14.5–34.32)	21.15(13.61–33.87)	−0.05(−0.11–0)	0.02(−0.01–0.05)	−0.04(−0.1–0.03)
Oceania	Both	30.66(24.11–40.92)	30.95(22.03–43.22)	0.02(−0.08–0.1)	−0.01(−0.06–0.03)	0.01(−0.11–0.1)
	Female	29.16(22.47–39.62)	29.2(20.74–42.23)	0.01(−0.1–0.11)	−0.01(−0.06–0.04)	0(−0.13–0.12)
	Male	32.11(25.29–42.1)	32.59(23.11–44.73)	0.03(−0.09–0.13)	−0.01(−0.08–0.05)	0.02(−0.13–0.13)
South Asia	Both	39.71(26.36–54.17)	40.5(25.87–54.9)	0.04(0–0.08)	−0.02(−0.04–0)	0.02(−0.04–0.06)
	Female	38.84(25.83–54.13)	36.77(23.47–52.52)	−0.01(−0.05–0.02)	−0.04(−0.08–0.01)	−0.05(−0.11–0.01)
	Male	40.51(26.81–54.63)	44.17(28.35–57.92)	0.09(0.04–0.16)	0(−0.02–0.02)	0.09(0.03–0.16)
Southeast Asia	Both	58.55(42.01–69.9)	45.37(27.48–60.3)	−0.18(−0.3–0.1)	−0.05(−0.11–0.02)	−0.23(−0.36–0.13)
	Female	54.6(36.02–68.21)	41.71(23.8–58.31)	−0.2(−0.33–0.11)	−0.05(−0.11–0.01)	−0.24(−0.38–0.13)
	Male	62.86(47.66–72.66)	49.37(31.39–62.91)	−0.17(−0.29–0.08)	−0.06(−0.12–0.02)	−0.21(−0.36–0.12)
Southern Latin America	Both	43.77(29.56–58.37)	40.91(26.41–56.1)	−0.07(−0.15–0.01)	0(−0.04–0.05)	−0.07(−0.16–0.01)
	Female	39.33(27.31–54.71)	35.97(24.23–52.03)	−0.08(−0.16–0)	−0.01(−0.06–0.03)	−0.09(−0.18–0.01)
	Male	48.51(31.25–63.07)	46.18(28–60.93)	−0.06(−0.17–0.03)	0.02(−0.05–0.09)	−0.05(−0.17–0.07)
Southern Sub–Saharan Africa	Both	40.07(23.86–70.07)	36.12(23.76–64.14)	−0.1(−0.2–0.03)	0(−0.04–0.04)	−0.1(−0.21–0.04)
	Female	38.44(23.49–69.9)	35.77(23.48–64.94)	−0.07(−0.18–0.04)	0(−0.05–0.05)	−0.07(−0.19–0.05)
	Male	41.83(24.08–70.97)	36.47(23.83–63.53)	−0.12(−0.26–0.03)	−0.01(−0.07–0.06)	−0.13(−0.27–0.04)
Tropical Latin America	Both	39.61(24.74–55.19)	39.26(25.4–53.72)	−0.02(−0.11–0.1)	0.01(−0.04–0.07)	−0.01(−0.11–0.13)
	Female	35.99(23.24–52.14)	35.61(24.65–50.05)	−0.03(−0.13–0.08)	0.01(−0.04–0.08)	−0.01(−0.13–0.12)
	Male	43.47(25.11–59.45)	43.23(25.38–59.13)	−0.01(−0.15–0.17)	0(−0.09–0.1)	−0.01(−0.16–0.19)
Western Europe	Both	36.27(25.75–50.06)	37.97(25.95–52.05)	0.01(−0.03–0.05)	0.03(0–0.06)	0.05(−0.02–0.1)
	Female	31.42(23.33–44.81)	31.91(22.9–45.72)	−0.01(−0.04–0.04)	0.02(−0.01–0.06)	0.02(−0.04–0.08)
	Male	41.29(27.23–56.2)	44.1(28.37–59.18)	0.03(−0.04–0.09)	0.04(0–0.09)	0.07(−0.01–0.15)
Western Sub–Saharan Africa	Both	30.54(18.61–59.9)	30.01(17.41–60.69)	−0.02(−0.08–0.04)	0(−0.03–0.04)	−0.02(−0.09–0.05)
	Female	30.09(18.45–59.04)	29.58(17.24–60.32)	−0.02(−0.08–0.05)	0(−0.03–0.04)	−0.02(−0.09–0.07)
	Male	30.96 (18.6–60.85)	30.48(17.53–60.57)	−0.02(−0.1–0.05)	0.01(−0.03–0.05)	−0.02(−0.11–0.08)

*ARC, annualized rate of change; SEV, summary exposure value; SDI, socio–demographic index; UI, uncertainty interval*.

Further analysis showed that age-standardized SEV rate increased for diets high in red meat (ARC = 0.09, 95% UI: 0.04–0.15, Supplementary Table 2) and low in milk (ARC = 0.03, 95% UI: 0.01–0.05, Supplementary Table 3). It decreased for diets low in calcium (ARC = −13, 95%UI: −0.06 to 0.18, Supplementary Table 4), whole grains (ARC = −0.01, 95% UI: −0.02 to 0.01, Supplementary Table 5), and fiber (ARC = −0.25, 95% UI: −0.21 to 0.3, Supplementary Table 6); it was kept stable for diets high in processed meat (ARC = −0.04, 95% UI: −0.11 to 0.04, Supplementary Table 7) for over 30 years globally.

### Global Attributable Burden

In 2019, an estimated 1 million people died from colorectal cancer, with 24 million DALYs. Dietary risk-related deaths and DALYs accounted for 32 and 34%, respectively (Tables 2, 3). The dietary risk was the primary factor of colorectal cancer death and DALY rate (Supplementary Figure 1).

**Table 2:** Deaths, ASDRs, and change trends of colorectal cancer attributable to dietary risks between 1990 and 2019.

**Location**	**Sex**	**Deaths (95% UI)**		**ASDR (95% UI)**		**EAPC (95% CI)**
		**1990**	**2019**	**1990**	**2019**	**1990–2019**
Global	Both	179639.2 (138268.4–212910.39)	365751.8 (272831.26–442144.29)	4.95 (3.82–5.88)	4.61 (3.43–5.58)	−0.29 (−0.36–0.22)
	Female	88528.61 (66627.46–105332.68)	161411.62 (118853.46–195783.1)	4.38 (3.3–5.24)	3.69 (2.72–4.47)	−0.7 (−0.77–0.62)
	Male	91110.58 (70612.93–107476.18)	204340.18 (152123.06–246151.48)	5.72 (4.43–6.73)	5.71 (4.26–6.91)	0.02 (−0.06–0.09)
**Sociodemographic Index**						
High SDI	Both	71931.18 (53299.33–88470.24)	105343.32 (76587.92–129173.68)	6.86 (5.09–8.43)	5.25 (3.83–6.41)	−1.04 (−1.11–0.98)
	Female	35679.85 (26077.35–44361.13)	48754.24 (35264.31–60676.94)	5.64 (4.12–6.98)	4.16 (3.01–5.13)	−1.19 (−1.28–1.11)
	Male	36251.34 (26897.69–44027.87)	56589.08 (41323.31–68668.46)	8.63 (6.42–10.51)	6.57 (4.8–7.96)	−1.06 (−1.11–1.01)
High–middle SDI	Both	57246.81 (42913.76–67889.67)	107585.91 (78263.39–131416.46)	5.66 (4.23–6.74)	5.35 (3.89–6.53)	−0.32 (−0.43–0.2)
	Female	28794.62 (21443.6–34485.75)	46426.25 (32897.5–57824.06)	4.89 (3.64–5.87)	4.04 (2.87–5.04)	−0.86 (−0.98–0.75)
	Male	28452.2 (21775.27–33706.01)	61159.65 (44589.53–75189.32)	6.85 (5.25–8.14)	7.11 (5.2–8.73)	0.09 (−0.04–0.22)
Low SDI	Both	5264.04 (4117.79–6470.27)	13113.09 (10344.37–15521.27)	2.47 (1.93–3.02)	2.8 (2.21–3.3)	0.44 (0.39–0.49)
	Female	2338.87 (1736.23–3084.6)	6206.81 (4830.83–7385.71)	2.21 (1.63–2.9)	2.58 (2.01–3.07)	0.55 (0.48–0.61)
	Male	2925.17 (2244.06–3850.34)	6906.28 (5339.58–8350.87)	2.73 (2.1–3.53)	3.04 (2.37–3.68)	0.38 (0.35–0.42)
Low–middle SDI	Both	13449.7 (10850.92–16002.52)	40684.34 (31497.84–48680.66)	2.48 (2.01–2.94)	3.2 (2.48–3.82)	0.89 (0.84–0.94)
	Female	6447.99 (5085.01–7836.74)	19306.24 (14853.87–23540.99)	2.39 (1.9–2.88)	2.89 (2.24–3.52)	0.62 (0.56–0.69)
	Male	7001.72 (5563.84–8797.55)	21378.09 (16386.79–25630.82)	2.56 (2.04–3.22)	3.53 (2.72–4.23)	1.16 (1.09–1.23)
Middle SDI	Both	31651.65 (25701.02–36920.12)	98820.99 (75848.49–119915.13)	3.36 (2.74–3.91)	4.22 (3.25–5.11)	0.96 (0.81–1.11)
	Female	15219.18 (12170.79–18040.02)	40621.79 (30883.32–50020.19)	3.11 (2.5–3.67)	3.3 (2.52–4.05)	0.27 (0.16–0.37)
	Male	16432.46 (13332.68–19480.45)	58199.2 (44331.31–71174.35)	3.64 (2.95–4.28)	5.28 (4.02–6.44)	1.54 (1.36–1.73)
**Region**						
Africa	Both	7057.68 (5597.92–8465.32)	17607.01 (13630.99–21016.94)	2.76 (2.19–3.3)	3.14 (2.44–3.73)	0.46 (0.42–0.51)
	Female	3308.55 (2582.86–4085.68)	8296.95 (6369.81–10076.31)	2.56 (1.98–3.13)	2.87 (2.21–3.48)	0.45 (0.4–0.51)
	Male	3749.13 (2967.26–4643.35)	9310.06 (7088.87–11151.48)	2.96 (2.34–3.63)	3.43 (2.63–4.09)	0.49 (0.45–0.54)
America	Both	33094.61 (24599.76–40381.23)	57120.16 (41832.33–70031.24)	5.51 (4.1–6.72)	4.44 (3.25–5.43)	−0.81 (−0.87–0.75)
	Female	16558.45 (12159.37–20279.28)	27272.61 (19781.77–33906.3)	4.79 (3.52–5.87)	3.78 (2.74–4.69)	−0.87 (−0.92–0.82)
	Male	16536.16 (12468.35–19938.51)	29847.54 (22140.67–36246.84)	6.43 (4.85–7.78)	5.22 (3.87–6.34)	−0.8 (−0.89–0.72)
Asia	Both	66786.92 (53102.08–77966.39)	195145.69 (147389.53–237221.87)	3.69 (2.93–4.31)	4.34 (3.29–5.28)	0.69 (0.55–0.83)
	Female	31144.97 (24484.32–37202.32)	81357.75 (61820.49–99481)	3.31 (2.6–3.95)	3.39 (2.58–4.15)	0.12 (0.02–0.22)
	Male	35641.95 (28058.24–42167.98)	113787.94 (85312.35–139420.85)	4.15 (3.27–4.87)	5.43 (4.12–6.66)	1.14 (0.97–1.32)
Europe	Both	72433.43 (88178.21–53156.58)	95298.3 (117189.59–67409.52)	7.03 (8.56–5.17)	5.87 (7.2–4.15)	−0.88 (−1.03–0.74)
	Female	37391.5 (45831.54–27002.24)	44218.97 (54941.5–30946.54)	5.88 (7.18–4.24)	4.54 (5.64–3.18)	−1.19 (−1.35–1.04)
	Male	35041.93 (42349.64–25992.69)	51079.33 (62990.88–36812.99)	8.94 (10.82–6.63)	7.71 (9.5–5.57)	−0.73 (−0.87–0.6)
Andean Latin America	Both	564.24 (449.13–676.29)	1897.82 (1352.58–2444.07)	2.95 (2.35–3.53)	3.48(2.48–4.49)	0.74 (0.6–0.88)
	Female	301.53 (237.13–362.13)	1006.72 (721.1–1301.36)	3.08 (2.43–3.7)	3.51 (2.52–4.54)	0.49 (0.35–0.62)
	Male	262.71 (207.71–319.13)	891.1 (639.38–1158.97)	2.8 (2.2–3.4)	3.43 (2.47–4.46)	1.04 (0.87–1.2)
Australasia	Both	1963.87 (1469.98–2384.69)	2953.68 (2192.44–3624.15)	8.5 (6.37–10.32)	5.74 (4.3–7.02)	−1.66 (−1.78–1.54)
	Female	932.18 (695.03–1138.53)	1362.7 (989.48–1696.88)	7.13 (5.3–8.72)	4.78 (3.51–5.91)	−1.63 (−1.74–1.53)
	Male	1031.68 (780.38–1245.66)	1590.98 (1199.44–1940.77)	10.28 (7.8–12.44)	6.83 (5.17–8.33)	−1.77 (−1.92–1.62)
Caribbean	Both	1110.65 (825.56–1321.26)	2501.5 (1734.12–3168.48)	4.45 (3.31–5.3)	4.84 (3.36–6.13)	0.25 (0.18–0.31)
	Female	570.44 (420.32–686.58)	1257.44 (861.55–1620.59)	4.38 (3.23–5.27)	4.47 (3.06–5.78)	0.01 (−0.05–0.07)
	Male	540.21 (403.17–641.78)	1244.06 (874.67–1584.35)	4.51 (3.37–5.36)	5.24 (3.7–6.66)	0.49 (0.41–0.58)
Central Asia	Both	1893.23 (1415.75–2257.29)	2609.51 (1914.55–3145.16)	4.07 (3.04–4.86)	3.91 (2.87–4.72)	0 (−0.12–0.13)
	Female	966.66 (712.59–1165.02)	1270.05 (920.45–1545.39)	3.52 (2.6–4.24)	3.34 (2.41–4.04)	−0.06 (−0.17–0.04)
	Male	926.57 (697.11–1095.76)	1339.47 (975.47–1624.49)	4.9 (3.7–5.8)	4.73 (3.4–5.71)	0.03 (−0.13–0.19)
Central Europe	Both	9515.82 (6655.97–11758.29)	15606.71 (10535.6–19884.82)	6.65 (4.65–8.23)	7.15 (4.84–9.09)	0.27 (0.15–0.38)
	Female	4448.42 (3053.54–5556.24)	6572.32 (4380.02–8479.17)	5.35 (3.68–6.69)	5.08 (3.35–6.58)	−0.24 (−0.35–−0.13)
	Male	5067.4 (3614.83–6250.35)	9034.4 (6186.29–11468.54)	8.5 (6.04–10.45)	10.03 (6.86–12.67)	0.67 (0.54–0.81)
Central Latin America	Both	1895.34 (1455.16–2277.69)	7018.48 (4940.31–8988.84)	2.44 (1.88–2.94)	3.04 (2.14–3.89)	0.73 (0.69–0.78)
	Female	998.35 (757.79–1213.54)	3394.03 (2377.59–4411.01)	2.5 (1.9–3.05)	2.71 (1.9–3.52)	0.26 (0.2–0.32)
	Male	897 (696.44–1063.87)	3624.45 (2556.47–4628.27)	2.37 (1.85–2.81)	3.42 (2.41–4.35)	1.23 (1.18–1.29)
Central Sub–Saharan Africa	Both	590.72 (446.83–758.46)	1402.16 (1017.61–1854.24)	2.98 (2.24–3.83)	2.97 (2.14–4.03)	−0.1 (−0.32–0.13)
	Female	260.33 (187.57–348.34)	663.51 (471.92–926.22)	2.5 (1.85–3.28)	2.54 (1.78–3.62)	0 (−0.19–0.19)
	Male	330.39 (243.36–451.32)	738.65 (516.69–1117.78)	3.51 (2.52–4.95)	3.57 (2.49–5.63)	−0.06 (−0.3–0.17)
East Asia	Both	31018.09 (24136.55–36894.33)	95245.6 (69351.49–119482)	3.86 (3.03–4.6)	4.85 (3.55–6.08)	1.15 (0.88–1.42)
	Female	14332.81 (10968.01–17683.65)	34563.28 (24432.75–45048.48)	3.4 (2.59–4.18)	3.31 (2.34–4.3)	−0.08 (−0.14–0.29)
	Male	16685.28 (12751.75–20675.21)	60682.32 (43189.63–79278.65)	4.54 (3.52–5.55)	6.85 (4.93–8.9)	1.94 (1.64–2.24)
Eastern Europe	Both	17925.21 (13334.61–21587.66)	19858.25 (13870.79–25211.21)	6.47 (4.83–7.8)	5.72 (4–7.27)	−1.02 (−1.29–0.76)
	Female	10177.61 (7492.29–12362.98)	10291.51 (7009.14–13367.37)	5.59 (4.13–6.79)	4.61 (3.12–6)	−1.26 (−1.5–1.02)
	Male	7747.6 (5807.9–9311.99)	9566.73 (6670.42–12252.84)	8.42 (6.33–10.12)	7.71 (5.4–9.84)	−0.93 (−1.22–0.63)
Eastern Sub–Saharan Africa	Both	1877.58 (1469.8–2329.76)	4653.33 (3528.04–5752.95)	2.71 (2.13–3.34)	3.13 (2.4–3.85)	0.5 (0.44–0.56)
	Female	843.25 (609.76–1121.49)	2176.25 (1651.36–2723.77)	2.39 (1.75–3.14)	2.77 (2.11–3.42)	0.54 (0.46–0.61)
	Male	1034.32 (778.49–1410.05)	2477.08 (1840.74–3220.05)	3.06 (2.33–4.01)	3.55 (2.69–4.58)	0.52 (0.47–0.57)
High–income Asia Pacific	Both	10886.35(7944.54–13313.66)	25261.76 (18175.96–31455.85)	5.68 (4.16–6.92)	5.04 (3.64–6.2)	−0.42 (−0.52–0.33)
	Female	4984.87 (3625.16–6146.25)	12023.19 (8176.39–15222.21)	4.5 (3.28–5.54)	3.86 (2.74–4.82)	−0.56 (−0.62–0.49)
	Male	5901.48 (4339.9–7200.45)	13238.57 (9605.88–16100.75)	7.38 (5.41–8.96)	6.46 (4.7–7.87)	−0.48 (−0.6–0.36)
High–income North America	Both	23253.37 (16965.09–28708.35)	30355.13 (21656.69–37839.02)	6.48 (4.73–7.98)	4.75 (3.43–5.9)	−1.22 (−1.32–1.12)
	Female	11604.68 (8455.78–14454.54)	14217.63 (10080.74–17920.27)	5.38 (3.88–6.68)	3.91 (2.78–4.9)	−1.21 (−1.29–1.14)
	Male	11648.69 (8549.76–14262.86)	16137.5 (11850.98–19927.56)	8.02 (5.89–9.84)	5.73 (4.21–7.07)	−1.35 (−1.48–1.21)
North Africa and Middle East	Both	4572.21 (3321.56–5752.11)	13092.2 (9312.48–16064.5)	2.89 (2.11–3.63)	3.3 (2.37–4.05)	0.6 (0.42–0.78)
	Female	2152.74 (1571.19–2760.68)	5839.8 (4120.29–7268.11)	2.74 (1.99–3.49)	3.01 (2.13–3.74)	0.46 (0.28–0.64)
	Male	2419.47 (1780.01–3158.08)	7252.39 (5189.19–8935.34)	3.04 (2.24–3.93)	3.59 (2.55–4.41)	0.72 (0.54–0.91)
Oceania	Both	78.96 (58.06–99.49)	210.95 (155.18–275.36)	3.01 (2.22–3.78)	3.39 (2.55–4.34)	0.4 (0.33–0.47)
	Female	35.42 (24.72–45.7)	93.98 (67.95–124.26)	2.79 (1.96–3.61)	3.12 (2.3–4.07)	0.37 (0.3–0.45)
	Male	43.54 (31.54–57.77)	116.97 (86.44–152.95)	3.24 (2.35–4.26)	3.69 (2.77–4.79)	0.42 (0.36–0.49)
South Asia	Both	9650.42 (7685.01–11679.28)	31191.38 (23747.6–38373.25)	1.96 (1.56–2.36)	2.44 (1.87–3)	0.64 (0.52–0.76)
	Female	4436.53 (3358.92–5620.88)	15450.4 (11326.59–19639.2)	1.86 (1.4–2.36)	2.36 (1.74–2.98)	0.65 (0.51–0.8)
	Male	5213.9 (4054.17–6642.62)	15740.98 (11619.01–19871.6)	2.05 (1.6–2.57)	2.53 (1.88–3.18)	0.66 (0.54–0.77)
Southeast Asia	Both	9939.21 (8112.04–11648.9)	32376.27 (24768.93–40353.41)	4.16 (3.41–4.86)	5.7 (4.37–7.08)	1 (0.94–1.06)
	Female	4842.1 (3876.51–5836.05)	14237.49 (10375.15–18142.7)	3.8 (3.05–4.54)	4.63 (3.41–5.85)	0.59 (0.52–0.67)
	Male	5097.12 (4156.31–6108.26)	18138.78 (14052.84–22556.32)	4.59 (3.76–5.5)	6.99 (5.36–8.65)	1.38 (1.32–1.44)
Southern Latin America	Both	3568.63 (2756.86–4195.37)	6818.04 (5126.41–8143.82)	8.07 (6.23–9.47)	8.07 (6.06–9.62)	0.03 (−0.04–0.11)
	Female	1685.54 (1280.62–1990.43)	3226.9 (2354.38–3897.67)	6.72 (5.12–7.96)	6.54 (4.8–7.9)	−0.09 (−0.17–−0.01)
	Male	1883.09 (1471.05–2207.64)	3591.13 (2738.95–4268.26)	9.8 (7.62–11.49)	10.06 (7.7–11.99)	0.16 (0.08–0.23)
Southern Sub–Saharan Africa	Both	944.18 (734.47–1195.95)	2193.23 (1719.33–2668.29)	3.75 (2.89–4.8)	4.26 (3.35–5.17)	0.45 (0.22–0.67)
	Female	480.66 (370.4–617.37)	1083.67 (835.48–1345.15)	3.34 (2.54–4.35)	3.58 (2.76–4.42)	0.35 (0.2–0.5)
	Male	463.52 (356.34–609.34)	1109.56 (865.85–1344.46)	4.25 (3.26–5.66)	5.27 (4.14–6.35)	0.66 (0.35–0.98)
Tropical Latin America	Both	2908.3 (2215.58–3504.74)	8983.97 (6692.65–11033.7)	3.49 (2.67–4.21)	3.78 (2.81–4.64)	0.31 (0.15–0.47)
	Female	1493.87 (1122.91–1806.85)	4379.91 (3161.21–5467.68)	3.36 (2.52–4.07)	3.3 (2.38–4.12)	−0.04 (−0.21–0.13)
	Male	1414.43 (1092.19–1690.93)	4604.06 (3412.15–5624.17)	3.64 (2.83–4.36)	4.38 (3.25–5.35)	0.71 (0.56–0.87)
Western Europe	Both	43390.97 (31730.84–52970.92)	56340.66 (40795.02–69276.86)	7.38 (5.41–9)	5.67 (4.09–6.95)	−1.11 (−1.26–0.96)
	Female	22020.89 (15888.11–27183.95)	25824.56 (18109.06–32340.04)	6.1 (4.42–7.51)	4.39 (3.13–5.45)	−1.37 (−1.54–1.2)
	Male	21370.09 (15872.37–25925.75)	30516.1 (22348.23–37299.29)	9.32 (6.94–11.29)	7.29 (5.34–8.9)	−1.02 (−1.16–0.89)
Western Sub–Saharan Africa	Both	2091.83 (1606.17–2643.13)	5181.18 (4002.21–6336.71)	2.69 (2.08–3.38)	3.2 (2.49–3.88)	0.77 (0.68–0.87)
	Female	959.73 (712.33–1284.74)	2476.29 (1865.1–3144.12)	2.47 (1.84–3.27)	2.97 (2.28–3.7)	0.83 (0.72–0.95)
	Male	1132.1 (846.76–1497.42)	2704.88 (2039.12–3428.87)	2.89 (2.19–3.76)	3.44 (2.62–4.32)	0.75 (0.67–0.84)

*ASDR, age–standardized death rate; EAPC, estimated annual percentage change; UI, uncertainty interval*.

**Table 3:** DALYs, ASRs, and change trends of colorectal cancer attributable to dietary risks by SDI, regions, and sex.

**Location**	**Sex**	**DALYs (No. × 1000, 95% UI)**		**Age-standardized DALY rate (95% UI)**		**EAPC (95% CI)**
		**1990**	**2019**	**1990**	**2019**	**1990–2019**
Global	Both	4300.02 (3337–5083.52)	8210.62 (6142.38–9952.59)	107.18 (83.19–126.83)	99.79 (74.65–121.14)	−0.28 (−0.35–0.22)
	Female	2015.83 (1543.99–2396.6)	3397 (2529.89–4119.78)	94.3 (72.01–112.15)	78.21 (58.27–94.84)	−0.76 (−0.82–0.71)
	Male	2284.18 (1766.05–2689.63)	4813.62 (3570.52–5803.1)	122.68 (94.83–144.95)	123.94 (91.94–149.22)	0.06 (−0.02–0.14)
**Sociodemographic Index**						
High SDI	Both	1510.19 (1109.33–1853.36)	1993.88 (1452.5–2433.47)	147.35 (108.3–180.75)	112.54 (82.48–136.67)	−1.04 (−1.09–1)
	Female	693.58 (507.27–855.42)	838.99 (610.16–1029.99)	119.27 (87.22–146.74)	87.34 (63.87–106.59)	−1.19 (−1.25–1.14)
	Male	816.61 (604.37–989.71)	1154.9 (845.41–1403.71)	183.8 (136.26–222.79)	140.72 (103.32–170.88)	−1.03 (−1.07–0.99)
High–middle SDI	Both	1388.37 (1047.13–1646.78)	2371.04 (1719.22–2905.27)	127.88 (96.36–151.85)	117.55 (85.31–143.99)	−0.44 (−0.55–0.33)
	Female	665.93 (500.05–797.97)	954.08 (678.38–1182.94)	110.75 (83.11–132.6)	87.25 (62.38–108.16)	−1.07 (−1.18–0.95)
	Male	722.45 (549.12–855.24)	1416.96 (1022.2–1747.8)	151.1 (114.59–178.77)	153.66 (110.9–189.22)	−0.01 (−0.14–0.12)
Low SDI	Both	145.87 (114.56–180.29)	349.92 (273.14–415.11)	57.24 (44.86–70.5)	62.75 (49.32–74.54)	0.3 (0.26–0.35)
	Female	66.24 (49.05–88.18)	165.46 (128.74–197.28)	51.96 (38.51–68.61)	58.01 (45.22–68.98)	0.37 (0.3–0.43)
	Male	79.63 (60.82–105.86)	184.46 (142.06–223.66)	62.28 (47.63–82.1)	67.71 (52.37–81.99)	0.28 (0.25–0.31)
Low–middle SDI	Both	373.4 (300.94–445.74)	1023.64 (783.53–1227.13)	57.84 (46.62–68.98)	72.18 (55.33–86.37)	0.77 (0.73–0.82)
	Female	179.3 (141.11–220.27)	475.67 (363.22–582.54)	56.03 (44.29–68.51)	64.94 (49.91–79.37)	0.45 (0.38–0.52)
	Male	194.11 (155.81–244.32)	547.97 (417.35–661.11)	59.62 (47.38–74.55)	80.01 (61.08–96.16)	1.09 (1.01–1.16)
Middle SDI	Both	879.98 (714.9–1027.6)	2467.6 (1902.01–3001.62)	78.82 (64.06–91.75)	96.3 (74.09–116.96)	0.88 (0.74–1.02)
	Female	409.73 (326.53–483.31)	960.78 (736.77–1185.61)	72.71 (58.19–85.74)	72.82 (55.79–89.62)	0.06 (−0.02–0.14)
	Male	470.25 (380.43–559.15)	1506.82 (1152.36–1838.08)	85.32 (69.17–101.1)	121.61 (93.07–148.49)	1.52 (1.32–1.71)
**Region**						
Africa	Both	188.5 (149.57–227.64)	461.56 (351.92–556.8)	62.1 (49.23–74.63)	68.64 (53.06–82.09)	0.36 (0.32–0.41)
	Female	86.76 (67.76–108.61)	212.53 (159.88–261.18)	56.68 (44.25–70.47)	61.48 (46.91–74.94)	0.34 (0.28–0.41)
	Male	101.74 (80.05–127.17)	249.03 (187.29–301.34)	67.49 (53.43–83.71)	76.25 (57.82–91.72)	0.4 (0.36–0.44)
America	Both	723.73 (543.48–878.44)	1241.73 (913.82–1510.25)	118.39 (88.96–143.81)	99.28 (73.05–120.71)	−0.64 (−0.69–0.58)
	Female	341.58 (252.74–418.03)	557.56 (404.27–683.88)	101.51 (75.28–124.16)	82.83 (60.1–101.41)	−0.72 (−0.76–0.67)
	Male	382.15 (287.81–460.02)	684.16 (509.18–829.26)	138.8 (104.63–167.4)	118.03 (87.83–143)	−0.61 (−0.68–0.53)
Asia	Both	1807.62 (1438.03–2116.73)	4661.07 (3513.52–5655.77)	84.2 (67.01–98.4)	95.8 (72.21–116.44)	0.58 (0.45–0.71)
	Female	821.87 (647.66–978.66)	1832.46 (1401.52–2247.23)	75.79 (59.65–90.5)	73.23 (55.94–89.73)	−0.12 (−0.2–0.05)
	Male	985.75 (775.79–1171.5)	2828.6 (2129.59–3457.95)	93.42 (73.41–110.84)	120.11 (90.04–146.93)	1.11 (0.93–1.28)
Europe	Both	1574.14 (1158.52–1906.23)	1834.29 (1298.71–2249.41)	153.82 (113.23–186.2)	124.47 (88.72–152.48)	−1.04 (−1.17–0.9)
	Female	762.94 (562.83–930.53)	789.36 (551.35–978.4)	127.83 (94.48–155.95)	95.09 (67.1–117.65)	−1.36 (−1.49–1.23)
	Male	811.2 (605.03–976.2)	1044.94 (760.88–1284.06)	191.89 (143.01–230.89)	161.46 (117.94–198.16)	−0.87 (−1.01–0.74)
Andean Latin America	Both	13.7 (10.77–16.47)	41.66 (29.44–54.4)	63.95 (50.53–76.83)	73.43 (51.99–95.52)	0.62 (0.48–0.76)
	Female	7.08 (5.49–8.58)	21.1 (14.85–27.38)	64.8 (50.63–78.51)	71.82 (50.57–92.93)	0.37 (0.23–0.51)
	Male	6.62 (5.21–8.03)	20.56 (14.6–27.28)	62.96 (49.44–76.8)	74.99 (53.33–99.82)	0.89 (0.73–1.05)
Australasia	Both	44.17 (33.13–53.29)	58.12 (43.58–70.89)	191.45 (143.87–231.16)	124.26 (93.49–151.64)	−1.8 (−1.93–1.67)
	Female	19.88 (14.81–24.17)	25.31 (18.83–30.96)	160.66 (120.29–195.65)	102.27 (76.57–124.6)	−1.81 (−1.91–1.7)
	Male	24.29 (18.3–29.13)	32.81 (24.76–39.85)	228.04 (171.91–274.17)	148.31 (112.13–179.96)	−1.84 (−1.99–1.69)
Caribbean	Both	25.31 (18.75–30.25)	53.73 (37.36–68.8)	95.88 (70.97–114.52)	104.08 (72.44–133.26)	0.26 (0.18–0.33)
	Female	12.69 (9.28–15.34)	25.67 (17.56–33.32)	93.07 (68.08–112.42)	93.88 (64.29–121.86)	−0.02 (−0.08–0.04)
	Male	12.62 (9.43–14.97)	28.06 (19.66–35.96)	98.75 (73.67–117.13)	115.18 (80.89–147.56)	0.53 (0.43–0.62)
Central Asia	Both	54 (40.57–64.28)	69.67 (51.26–84.44)	107.98 (80.98–128.78)	89.35 (65.49–108.11)	−0.68 (−0.8–0.55)
	Female	25.85 (19.16–31.07)	32.05 (23.04–39.14)	92.04 (68.25–110.64)	74.8 (53.94–90.95)	−0.77 (−0.87–0.68)
	Male	28.15 (21.22–33.3)	37.62 (27.59–45.69)	129.59 (97.59–153.46)	108.24 (78.42–131.09)	−0.61 (−0.78–0.44)
Central Europe	Both	221.25 (155.24–273.77)	320.19 (217.45–405.66)	150.2 (105.71–185.55)	156.18 (107.13–197.77)	0.15 (0.05–0.26)
	Female	97.96 (68.04–122.22)	125.07 (84.33–161.98)	119.08 (82.83–148.49)	109.27 (74.04–141.75)	−0.33 (−0.42–0.25)
	Male	123.29 (88.26–152.66)	195.12 (133.37–249.52)	190.77 (136.42–235.75)	215.28 (146.9–274.8)	0.48 (0.35–0.61)
Central Latin America	Both	47.63 (36.39–57.08)	166.02 (116.31–213.99)	53.37 (40.88–64.02)	68.95 (48.41–88.73)	0.88 (0.83–0.93)
	Female	24.22 (18.3–29.32)	76.57 (53.41–99.78)	53.04 (39.97–64.43)	59.47 (41.53–77.48)	0.41 (0.35–0.48)
	Male	23.41 (18.09–27.77)	89.46 (63.16–115.17)	53.66 (41.58–63.55)	79.72 (56.41–102.34)	1.34 (1.28–1.4)
Central Sub–Saharan Africa	Both	16.69 (12.48–21.57)	39.32 (28.16–52.12)	67.95 (51.59–87.36)	67.05 (48.71–89.1)	−0.13 (−0.34–0.09)
	Female	7.42 (5.23–10.14)	17.98 (12.82–24.81)	56.83 (41.22–76.12)	57.18 (40.85–79.37)	−0.02 (−0.19–0.16)
	Male	9.27 (6.81–12.83)	21.34 (14.58–32.03)	80.51 (59.53–111.03)	79.55 (55.42–121.94)	−0.17 (−0.4–0.07)
East Asia	Both	873.36 (678.24–1041.45)	2346.39 (1700.99–2967.47)	92.25 (71.83–109.96)	112.83 (82.05–142.19)	1.08 (0.82–1.34)
	Female	391.37 (298.29–486.06)	801.34 (562.65–1043)	82.18 (62.79–102.03)	74.88 (52.67–97.45)	−0.19 (−0.36–0.02)
	Male	481.98 (366.84–598.24)	1545.05 (1098.63–2028.69)	104.23 (79.74–129.38)	154.74 (110.51–201.96)	1.92 (1.6–2.24)
Eastern Europe	Both	443 (327.45–534.45)	441.29 (309.5–560.46)	157.39 (116.55–189.97)	131.46 (92.13–166.79)	−1.3 (−1.6–1)
	Female	238.3 (174.62–289.34)	213.48 (143.59–277.78)	136.73 (100.59–166.19)	105.06 (70.35–136.65)	−1.59 (−1.86–1.31)
	Male	204.7 (153.37–245.81)	227.8 (158.46–293.4)	196.73 (147.81–237.01)	172.95 (120.15–220.57)	−1.12 (−1.44–0.8)
Eastern Sub–Saharan Africa	Both	52.92 (41.08–66.17)	128.27 (96.42–159.97)	64.24 (50.33–79.83)	70.96 (53.6–87.96)	0.33 (0.26–0.4)
	Female	24.34 (17.4–32.86)	59.33 (44.27–75.18)	57.26 (41.22–76.19)	62.77 (47.22–78.73)	0.3 (0.21–0.39)
	Male	28.58 (21.49–39.61)	68.94 (50.7–89.98)	71.28 (53.58–97.37)	80.01 (59.15–104.08)	0.39 (0.34–0.45)
High–income Asia Pacific	Both	255.21 (185.36–314.74)	438.82 (320.95–537.6)	125.9 (91.41–155.07)	107.52 (79.04–131.26)	−0.59 (−0.7–0.47)
	Female	109.74 (79.25–136.56)	182.52 (131.21–226.96)	98.27 (71.02–122.32)	79.78 (58.36–98.12)	−0.78 (−0.87–0.7)
	Male	145.47 (106.53–178.12)	256.3 (187.81–312.13)	161.59 (118.17–197.6)	138.47 (101.99–168.63)	−0.57 (−0.7–0.43)
High–income North America	Both	488.08 (357.16–598.6)	634.9 (456.84–783.79)	142.64 (104.97–174.69)	108.82 (78.24–133.86)	−1.03 (−1.13–0.93)
	Female	227.12 (164–280.55)	275.49 (197.5–344.57)	117.03 (85.56–143.91)	88 (62.68–109.2)	−1.06 (−1.14–0.98)
	Male	260.97 (191.73–318.76)	359.41 (261.95–442.15)	175.41 (128.59–214.29)	132.13 (96.4–162.06)	−1.1 (−1.23–0.98)
North Africa and Middle East	Both	124.72 (91.55–157.94)	334.99 (235.71–413.49)	67.26 (49.09–84.95)	72.57 (51.33–89.26)	0.35 (0.17–0.53)
	Female	58.09 (41.87–75.39)	146.56 (102.18–182.58)	63.59 (46.04–81.94)	65.16 (45.86–80.95)	0.16 (0.01–0.32)
	Male	66.64 (48.53–87.69)	188.42 (132.1–233.31)	70.77 (51.69–92.56)	79.71 (56.79–98.44)	0.5 (0.31–0.7)
Oceania	Both	2.32 (1.68–2.95)	6.13 (4.49–8.14)	69.86 (51.48–88)	77.68 (57.07–101.22)	0.35 (0.3–0.41)
	Female	1.02 (0.71–1.31)	2.66 (1.88–3.59)	64 (44.68–82.59)	70.41 (50.81–93.45)	0.33 (0.26–0.4)
	Male	1.3 (0.93–1.74)	3.47 (2.53–4.61)	75.52 (54.86–99.85)	84.7 (62.59–110.71)	0.38 (0.32–0.43)
South Asia	Both	266.57 (210.7–322.12)	766.17 (572.31–947.95)	44.1 (35.07–53.26)	53.08 (40.06–65.54)	0.54 (0.44–0.65)
	Female	125.57 (94.36–159.35)	377.88 (272.91–482.79)	42.89 (32.33–54.36)	51.62 (37.34–65.73)	0.5 (0.36–0.65)
	Male	141.01 (109.36–180.46)	388.28 (283.57–487.76)	45.25 (35.25–57.57)	54.63 (40.05–68.51)	0.59 (0.49–0.69)
Southeast Asia	Both	277.37 (226.11–327.3)	837.95 (643.88–1045.17)	98.89 (80.66–115.91)	130.74 (100.31–162.77)	0.87 (0.8–0.94)
	Female	132.13 (103.47–161.49)	349.11 (255.34–448.3)	89.67 (70.88–108.73)	103.55 (75.31–132.57)	0.4 (0.32–0.48)
	Male	145.24 (118.77–174.72)	488.85 (377.81–607.63)	109.32 (89.05–131.1)	161.8 (125.53–200.71)	1.27 (1.21–1.33)
Southern Latin America	Both	78.61 (60.88–92.39)	139.37 (104.3–166.42)	170.1 (131.68–200)	170.06 (127.4–202.99)	0.06 (0.01–0.12)
	Female	35.1 (26.78–41.73)	61.44 (45.12–74.44)	138.23 (105.45–164.12)	135.11 (99.7–163.53)	−0.02 (−0.08–0.04)
	Male	43.51 (34.07–50.93)	77.93 (59.1–93.1)	209.02 (163.33–244.67)	212.62 (161.73–254.05)	0.12 (0.06–0.19)
Southern Sub–Saharan Africa	Both	23.87 (18.87–29.31)	54.64 (42.58–66.72)	81.73 (63.95–102.59)	92.62 (72.61–113.46)	0.51 (0.28–0.74)
	Female	11.24 (8.91–13.86)	24.95 (19.07–31.35)	69.66 (54.64–87.27)	75.09 (57.41–94.56)	0.53 (0.38–0.69)
	Male	12.63 (9.82–16.19)	29.69 (22.93–36.28)	96.34 (74.3–125.56)	116.13 (90.29–141.17)	0.58 (0.24–0.91)
Tropical Latin America	Both	75.02 (56.88–90.54)	215.15 (158.74–264.19)	77.47 (58.91–93.63)	87.22 (64.43–106.99)	0.45 (0.26–0.64)
	Female	37.42 (28.08–45.49)	101.2 (73.41–125.42)	73.49 (55.05–89.15)	75.84 (55.08–94.02)	0.15 (−0.04–0.34)
	Male	37.59 (29.02–44.84)	113.95 (84.75–139.21)	81.83 (63.12–97.61)	100.79 (74.92–123.48)	0.77 (0.58–0.96)
Western Europe	Both	863.71 (636.17–1051.3)	987.94 (717.78–1211.32)	154.04 (113.72–187.21)	115.62 (85.26–141.28)	−1.19 (−1.31–1.07)
	Female	406.27 (297.3–497.84)	416.42 (295.08–513.64)	125.72 (92.73–153.54)	88.51 (63.28–108.28)	−1.43 (−1.54–1.31)
	Male	457.44 (340.94–554.48)	571.52 (422.65–697.33)	192.16 (143.24–232.73)	147.05 (108.72–179.45)	−1.11 (−1.24–0.99)
Western Sub–Saharan Africa	Both	52.52 (39.99–66.88)	129.92 (99.06–161.19)	57.94 (44.31–73.44)	66.23 (51.23–81.5)	0.6 (0.51–0.69)
	Female	23.03 (17.08–31.21)	60.87 (44.86–79.64)	52.19 (38.67–70.26)	59.96 (45.01–76.94)	0.65 (0.53–0.77)
	Male	29.49 (21.84–39.18)	69.05 (51.8–88.15)	63.27 (47.35–83.37)	73.04 (54.79–92.42)	0.62 (0.54–0.7)

*DALYs, disability–adjusted life years; EAPC, estimated annual percentage change; SDI, socio–demographic index; UI, uncertainty interval*.

**Figure 1:**
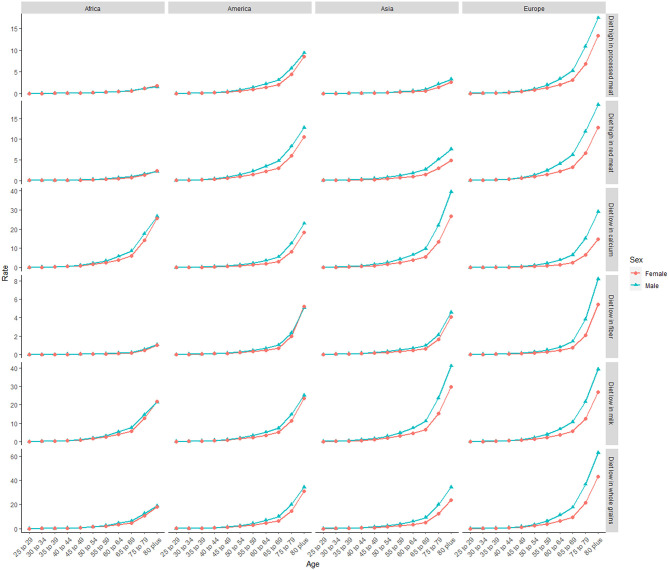
The death rate of six dietary risk factors related to colorectal cancer among four world regions in 2019. The vertical axis is the death rate (per 100,000 persons). The horizontal axis represents different age groups.

**Figure 2:**
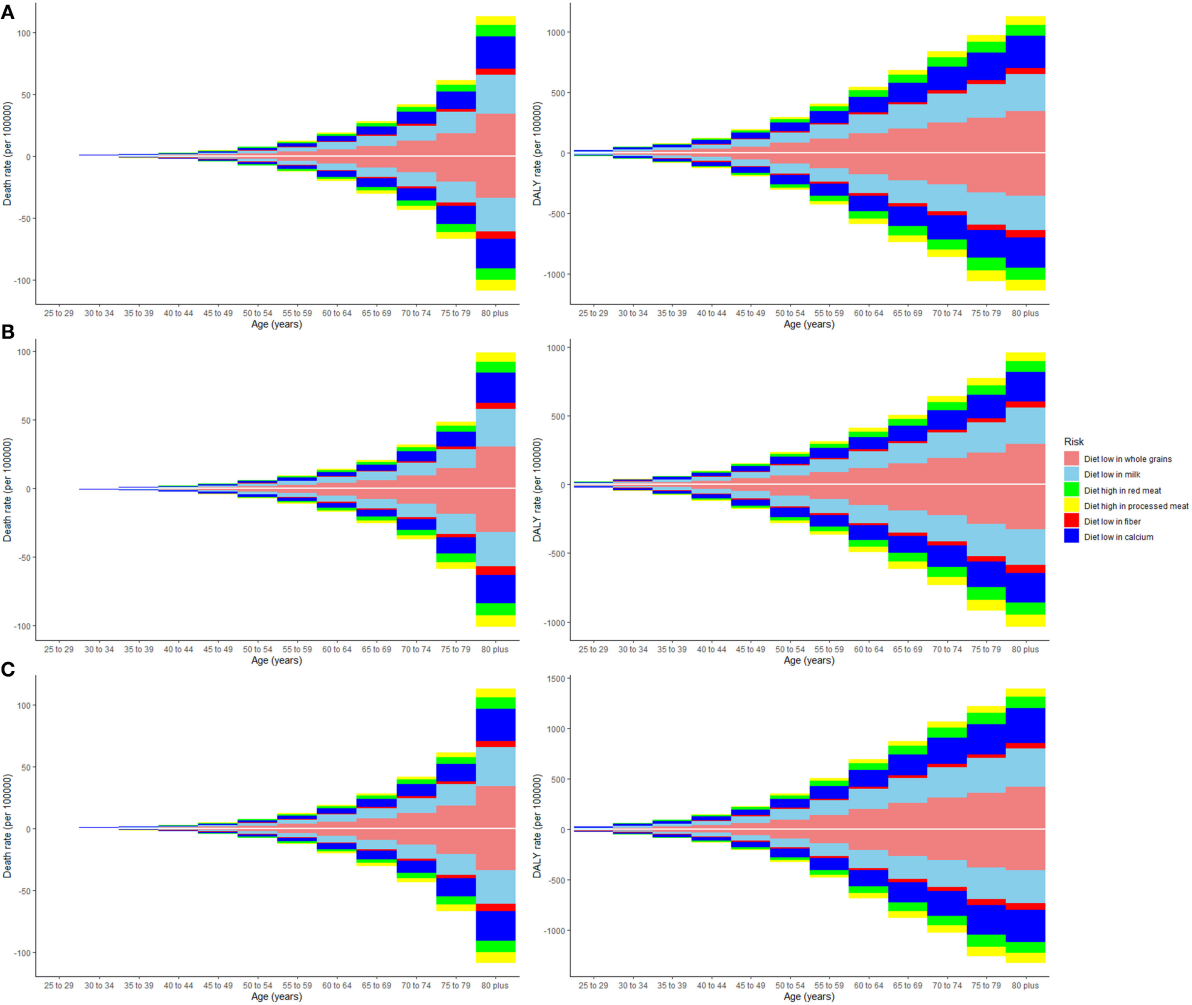
The death and disability-adjusted life-years (DALY) rate of six dietary risk factors related to colorectal cancer among various age groups. The upper column in each group represents data in 2019, and the lower column in each group represents data in 1990. The vertical axis is the death or DALY rate (per 100,000 persons). The horizontal axis represents different age groups. **(A)** Both genders, **(B)** women, and **(C)** men.

Deaths and DALYs of colorectal cancer due to dietary risks increased by 50.88 and 47.63%, respectively. Women accounted for 41.37 and 44.13% of colorectal cancer deaths and DALYs due to dietary risks, with 58.63% and 55.87% for men (Tables 2, 3). Men had higher death and DALY rates than women at all ages, and the gap widened with age (Figure 1 and Supplementary Figure 2). Both age-standardized death rate (ASDR) and age-standardized DALY rate were higher among men (with a stable trend) than among women (with a declining trend). As for risk factors, low-whole grain diets remained the leading cause of colorectal cancer death and DALY rate over time, followed by milk and calcium (Supplementary Figure 3). Whole grains, milk, and calcium accounted for 81.61% of deaths and 81.64% of DALYs for dietary risk factors related to the colorectal cancer burden globally (Supplementary Table 8). The leading cause of colorectal cancer death and DALY rate was low whole grains in America and Europe, low milk in Asia, and low calcium in Africa (Supplementary Figure 4). The ASDR and age-standardized DALY rates of four risk factors related to colorectal cancer decreased in several factors (including grains, fiber, red meat, and processed meat, Supplementary Tables 9–16); increased in one (milk, Supplementary Tables 17, 18); and kept one stable (calcium, Supplementary Tables 19, 20). Alarmingly, diets high in red meat were related to the increase in ASDR of colorectal cancer among men (EAPC = 0.07, 95% CI: 0.01–0.12, Supplementary Table 13). The ASDR of low whole grains was attributable to decreased colorectal cancer among women (EAPC = −0.7, 95% CI: −0.78 to 0.62) but was stable among men (EAPC = 0.02, 95% CI: −0.05 to 0.1, Supplementary Table 8). The ASDR of colorectal cancer due to diets low in milk and calcium decreased among women (milk: EAPC = −0.19, 95% CI: −0.23 to 0.15, Supplementary Table 17; calcium: EAPC = −0.35, 95% CI: −0.42 to 0.28, Supplementary Table 19) but increased among men (milk: EAPC = 0.48, 95% CI: 0.43–0.54; calcium: EAPC = 0.27, 95% CI: 0.15–0.39). The three leading dietary risk factors of the DALY rate were whole grains, milk, and red meat; for the death rate, they were whole grains, milk, and calcium among all age groups and genders (Figure 2).

### Attributable Burden by Socio-Demographic Index

**Figure 3:**
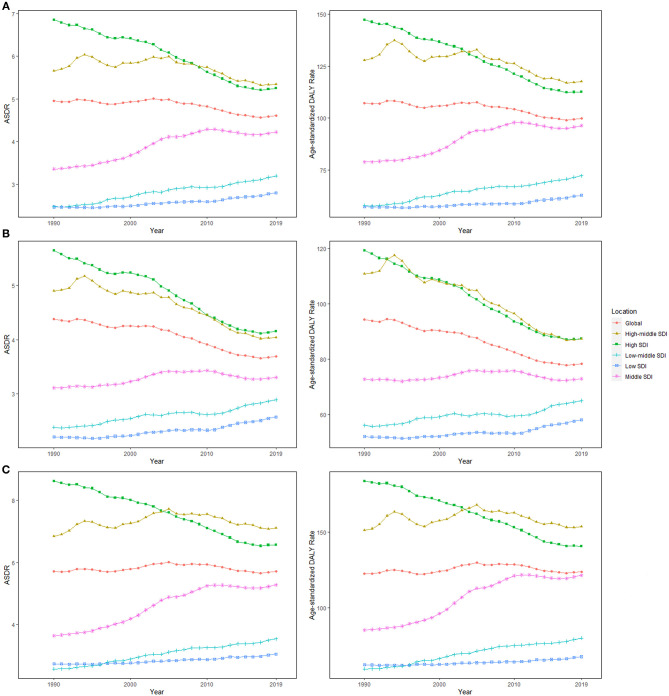
The age-standardized rates (ASRs) for dietary risk-related colorectal cancer among socio-demographic index (SDI) quintiles over the last 30 years. **(A)** Both genders, **(B)** women, and **(C)** men. ASDR, age-standardized death rate; DALY, disability-adjusted life-year; SDI, socio-demographic index.

**Figure 4:**
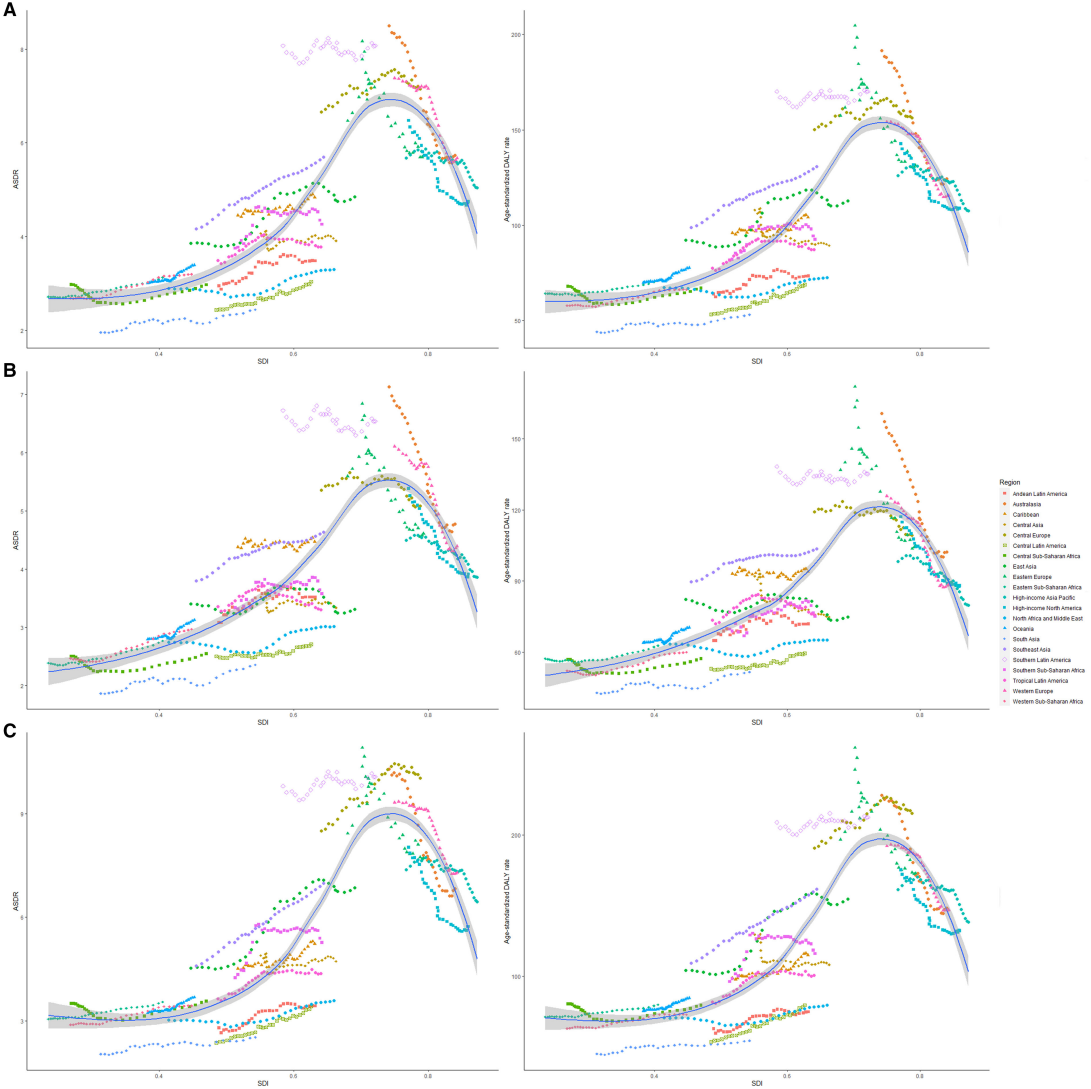
The ASRs of dietary risk related colorectal cancer among 21 regions based on SDI in 2019. The vertical axis is the age-standardized death and DALY rate (per 100,000 person-years), and the horizontal axis is the SDI value in 2019. Each combination of colors and shapes represents a region. Each point represents the age-standardized death and DALY rate (per 100,000 person-years) that year in each region. Each combination of the same color and shape, from front to back, represents the data for each year from 1990 to 2019. **(A)** ASDR or age-standardized DALY rate for both genders; **(B)** ASDR or age-standardized DALY rate for women; and **(C)** ASDR or age-standardized DALY rate for men. ASDR, age-standardized death rate; DALY, disability-adjusted life-year; SDI, socio-demographic index.

High-middle and middle SDI quintiles have always carried the highest deaths of colorectal cancer attributable to dietary risks (Table 2). The district with the highest DALYs changed from high SDI in 1990 into middle SDI in 2019 (Table 3). The ASDR and age-standardized DALY rates showed an upward trend in the middle, low-middle, and low SDI quintiles and increased the fastest in the middle SDI quintile (ASDR: EAPC = 0.96, 95% CI: 0.81–1.11; DALY: EAPC = 0.88, 95% CI: 0.74–1.02, Figure 3). In contrast, ASDR and age-standardized DALY rates declined the fastest in the high SDI quintile (ASDR: EAPC = −1.04, 95% CI: −1.11 to 0.98; DALY: EAPC = −1.04, 95% CI: −1.09 to 1). For men, ASDR and age-standardized DALY rates increased in all SDI quintiles, except for the high SDI quintile (ASDR: EAPC = −1.06, 95% CI: −1.11 to 1.01; DALY: EAPC = −1.03, 95% CI: −1.07 to 0.99). The ASDR and age-standardized DALY rates among regions increased first and then decreased with an increase in SDI value, with the inflection point being 0.75 (Figure 4).

### Regional Attributable Burden

**Figure 5:**
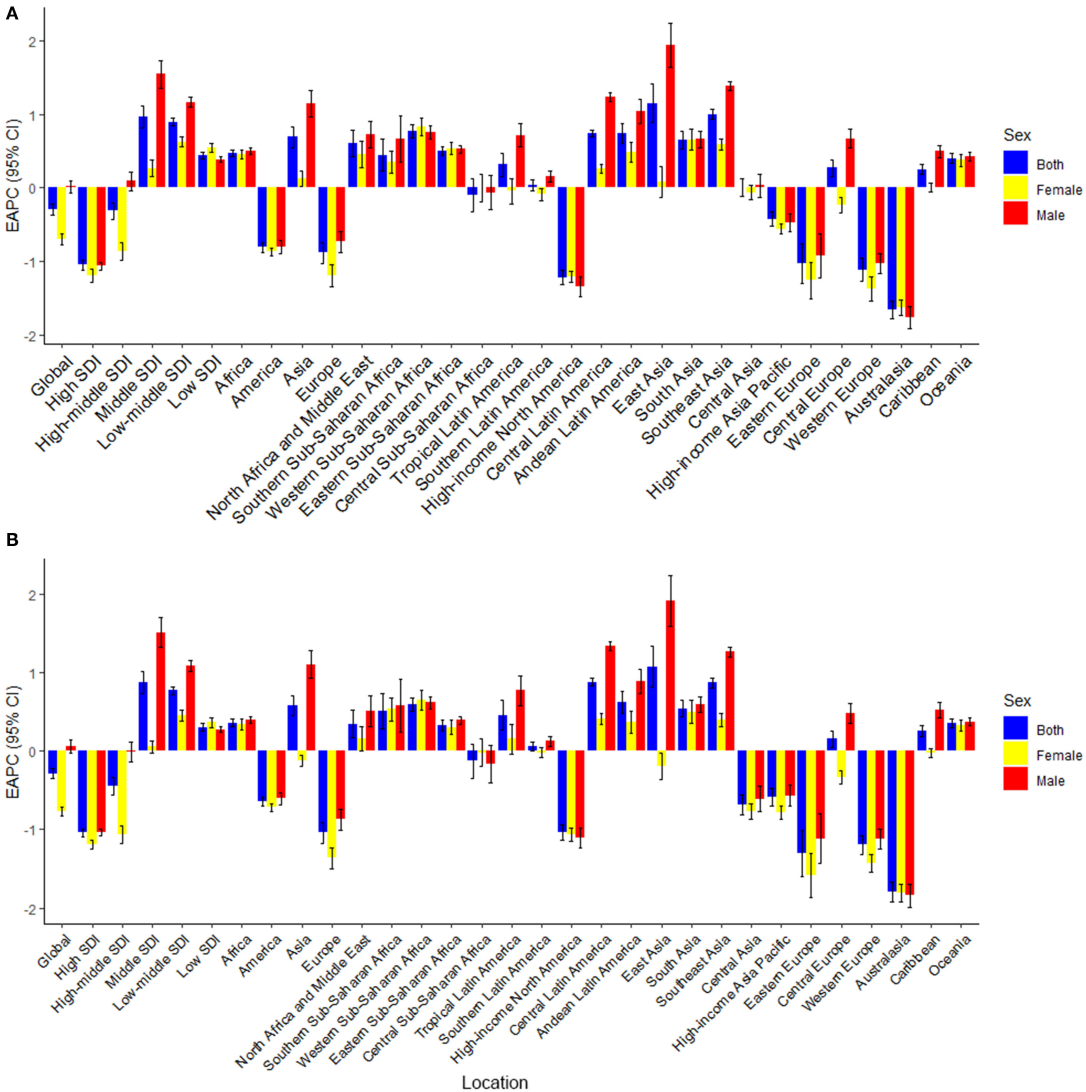
The estimated annual percentage changes (EAPCs) of age-standardized death and DALY rate for dietary risk related colorectal cancer. **(A)** EAPCs of ASDR (per 100,000 population); **(B)** EAPCs of age-standardized DALY rate (per 100,000 population). EAPC, estimated annual percentage changes; DALY, disability-adjusted life-year.

Asia carried the highest colorectal cancer DALYs attributable to dietary risks (accounting for almost half of the world) for over 30 years, especially for East Asia (accounting for almost half of Asia, Table 3). The region with the highest deaths changed from Europe (particularly in Western Europe) in 1990 to Asia (particularly in East Asia) in 2019 (Table 2). On the other hand, Europe has always had the highest ASDR and age-standardized DALY rate with the fastest decreasing rate (ASDR: EAPC = −0.88, 95% CI: −1.03 to 0.74; DALY: EAPC = −1.04, 95% CI: −1.17 to 0.9, Figure 5), while Africa always had the lowest. From 1990 to 2019, both ASDR and age-standardized DALY rate increased the fastest in Asia (ASDR: EAPC = 0.69, 95% CI: 0.5–0.83; DALY: EAPC = 0.58, 95% CI: 0.45−0.71), particularly in East Asia. However, the age-standardized DALY rate showed a downward trend for women in East Asia (EAPC = −0.19, 95% CI: −0.36 to 0.02).

### Attributable Burden by Countries and Territories

China always had the highest deaths (29,656 in 1990 and 90,407 in 2019) and DALY (834,715.34 in 1990 and 2,234,062.97 in 2019) of colorectal cancer attributable to dietary risks, followed by the USA, India, and Japan. From 1990 to 2019, the country with the highest ASDR and age-standardized DALY rate changed from Uruguay (ASDR: 10.91, 95% UI: 8.31–12.95; DALY: 230.92, 95% UI: 175.88–275.31) to Greenland (ASDR: 9.78, 95% UI: 6.83–12.82; DALY: 213.43, 95% UI: 145.89–281.33). The ASDR increased the fastest in Equatorial Guinea (EAPC = 2.62, 95% CI: 2.422.81, Supplementary Table 21), and the age-standardized DALY rate increased the fastest in Lesotho (EAPC = 2.6, 95%CI: 2.37–2.83, Supplementary Table 22) but decreased the fastest in Austria. All the countries and territories among the regions are shown in Supplementary Table 23.

## Discussion

This analysis comprehensively investigated the latest data on dietary risk factors related to colorectal cancer burden. Deaths and DALYs of colorectal cancer grew by 50.88 and 47.63% over the last 30 years. Dietary risk factors pose a greater global burden than smoking or drinking (10), highlighting the urgency of improving diets across regions and countries. The burden of colorectal cancer due to dietary factors differs between men and women. Compared with women, men tend to eat less healthily. Thus, this difference should be considered in the prevention programs of national policymakers. Though the effect of dietary factors varied across regions and countries, three dietary factors (diets low in whole grains, milk, and calcium) accounted for more than four-fifths of the colorectal cancer burden attributable to diet, mirroring the global imbalance of nutrition, which is consistent with the previous study (11). Dietary modifications are an effective strategy to reduce the colorectal cancer burden. Though dietary interventions have been identified, the observed effects of most of these measures fall far short of what is required for an optimal global diet (12).

Obesity is related to the occurrence of colorectal cancer (13). However, balanced nutrition diets can regulate physique and improve obesity. Our findings suggest that people should attach importance to nutritionally balanced diets and not just the restriction of sugar (14) and fat (15, 16).

Alarmingly, there was a significant upward trend in the rate of people exposed to low milk, which reminds the government to take measures, such as scientific education and interventions, to stimulate the production, supply, and consumption of healthy food (17). Previous studies indicated that milk and calcium intake significantly reduced the risk of colorectal cancer (18). It was found that milk intake varied widely from region to region. Interestingly, in high-income North America (Canada, Greenland, and the USA), dietary exposure was growing at a high speed. In Southeast Asia and Western and Central sub-Saharan Africa, people remained highly exposed to low milk intake. In contrast, milk intake in Eastern Europe, Australasia (Australia and New Zealand), Central Asia, and high-income North America improved considerably, especially in Australia. Studies have shown that milk intake plays a protective effect against colon cancer in people (19, 20). According to a research study, calcium, vitamin D, conjugated linoleic acid, butyric acid, and lactose in milk have certain anti-tumor effects (20). In addition, whole grains have the anti-cancer properties of fiber, antioxidants, and phytochemicals (21). It is worth noting that whole grain intakes are low in Central Asia compared to the high intakes of whole grains in Southeast Asia. It has been proven that high fiber intake is associated with better survival of patients with colorectal cancer (22), particularly in cereals (21).

The analysis also presented that the diets of people in Oceania and Western sub-Saharan Africa were rich in fiber, but the opposite was true for Southeast Asians and Southern Latin Americans (Argentina, Chile, and Uruguay). In addition, low calcium intake exposure was severe in Central sub-Saharan Africans and Southeast Asians. According to the published studies, calcium affects the progression of colorectal cancer by inhibiting cell proliferation and DNA oxidative damage. By extension, it promotes cell differentiation and apoptosis and regulates the cell signaling pathways associated with colorectal cancer (4).

In line with this, there is much room for improvement in public management policies (16), community programs, and primary healthcare interventions on dietary risks to have a greater impact on disease outcomes (23).

Calcium, fiber, milk, and whole grains were associated with a lower risk of colorectal cancer, while red and processed meats were correlated with an increase in this risk (4). Effective prevention programs and appropriate strategies, such as limiting junk food advertising, raising taxes on unhealthy foods, offering subsidies for the consumption of healthy foods, and providing supply chain incentives to promote production, are necessary to reduce the dietary risk factors in populations (23). It was observed that high red meat (especially for men) consumption in patients with colorectal cancer increased over the last 30 years. There was a high intake of red meat in Australia and Southern Latin America (Argentina, Chile, and Uruguay), while this intake was the lowest in South Asia (Bangladesh, Bhutan, India, Nepal, and Pakistan). Red meat consumption increased the fastest in East Asia (China and North Korea), which should be taken seriously. As for processed meat, consumption remained high among high-income North Americans and Western Europeans. Processed meat consumption increased globally, particularly in East Asia, Tropical Latin America (Brazil and Paraguay), and Southeast Asia. Heme iron, N-nitro compounds, and heterocyclic amines found in red and processed meats can produce carcinogens when cooked at high temperatures (24). Governments should strengthen assessments based on the life-circle (25) and carry out targeted prevention programs across nations, which is a strategy that is more environmentally sustainable (26).

Without health-related measure values, SDI was found to be a better way to assess the impact of health risk factors (27) than health-related human development indices (28). Deaths and DALYs of dietary factor-related colorectal cancer increased globally, particularly in the high-middle and middle SDI quintiles, and continued to decline in the high SDI quintile. This might have been due to the benefits of balanced diets and the huge improvements in diagnostics and interventions in this quintile.

Similarly, low socioeconomic status was associated with a higher risk of colorectal cancer (7). Our study demonstrated the gaps in regional and national colorectal cancer data on nutrient intake worldwide, suggesting the necessity of national surveillance systems for key dietary factors (29). Asia accounted for almost half of this burden worldwide, particularly in East Asia, which had the fastest increasing rate (30). Thus, it is essential for Chinese women and Hungarian men to cultivate a balanced diet to reduce their high dietary risk exposure (31). Countries should also use innovative technologies to strengthen their distinctive policy programs for the improvement of diet sustainability (32).

Some limitations were inevitable in this study. First, part of the variation in the age-standardized rates might have resulted from test biases or changes in screening regimens. In addition, many underdeveloped countries lack detailed data for further investigation. Though various data sources such as cancer registries and vital registries were used for cancer estimates, estimates for countries with no relevant information were dependent on predictive covariates or trends in neighboring countries.

The joint assessment of multiple dietary risk factors is important for the establishment of public policy. In addition, more detailed dietary surveys are needed to strengthen the accuracy and reliability of the conclusions. Further research studies on dietary factors are required to expand and deepen the understanding of dietary risk factors related to colorectal cancer and improve its detection, treatment, and prognosis.

In conclusion, dietary risk factors affected the burden of colorectal patients regardless of age. In addition, there were large variations of dietary risks related to the colorectal cancer burden among sexes, regions, and countries. The ASDR and age-standardized DALY rate presented downward trends globally, especially in the high SDI quintile. All ASRs remained higher among men (stable) than in women (decline) over the measurement periods, indicating that men need more diet control than women. Lastly, the findings could provide valuable information for policymakers to target interventions and address modifiable risk factors and for researchers to design and conduct studies on the early diagnosis and treatment of colorectal cancer and a balanced diet.

## Data Availability Statement

The original contributions presented in the study are included in the article/Supplementary Material, further inquiries can be directed to the corresponding author/s.

## Ethics Statement

The studies involving human participants were reviewed and approved by The First Affiliated Hospital, College of Medicine, Zhejiang University. Written informed consent for participation was not required for this study in accordance with the national legislation and the institutional requirements.

## Author Contributions

All authors read, critically reviewed, and approved the final manuscript. ZD and JL designed the research. YD, BW, and ZZ collected the data. YZ, JY, and SW verified the accuracy of the data. DX, JH, XY, and SY contributed to the data interpretation. YD, YW, NL, and PX performed the statistical analysis and visualization. YD wrote the manuscript.

## Conflict of Interest

The authors declare that the research was conducted in the absence of any commercial or financial relationships that could be construed as a potential conflict of interest.

## Publisher's Note

All claims expressed in this article are solely those of the authors and do not necessarily represent those of their affiliated organizations, or those of the publisher, the editors and the reviewers. Any product that may be evaluated in this article, or claim that may be made by its manufacturer, is not guaranteed or endorsed by the publisher.
