# Supplementary material for: Dietary Risk-Related Colorectal Cancer Burden: Estimates From 1990 to 2019
Source: Front Nutr. 2021 Aug 24;8:690663. doi: 10.3389/fnut.2021.690663 (PMC8421520; doi:10.3389/fnut.2021.690663)
Supplement: Supplementary file 3 [file Data_Sheet_3.zip › Supplemental tables/Appendix 1.docx]

**
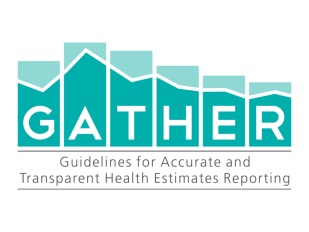
Checklist of information that should be included in new reports of global health estimates**

| Item # | Checklist item | Reported on page # |
| --- | --- | --- |
| Objectives and funding | | |
| 1 | Define the indicator(s), populations (including age, sex, and geographic entities), and time period(s) for which estimates were made. | See main manuscript “Data sources” |
| 2 | List the funding sources for the work. | See main manuscript “Funding” |
| Data Inputs | | |
| *For all data inputs from multiple sources that are synthesized as part of the study:* | | |
| 3 | Describe how the data were identified and how the data were accessed. | See main manuscript “Data sources” |
| 4 | Specify the inclusion and exclusion criteria. Identify all ad-hoc exclusions. | See main manuscript “Statistical analysis” |
| 5 | Provide information on all included data sources and their main characteristics. For each data source used, report reference information or contact name/institution, population represented, data collection method, year(s) of data collection, sex and age range, diagnostic criteria or measurement method, and sample size, as relevant. | http://ghdx.healthdata.org/gbd-2019 |
| 6 | Identify and describe any categories of input data that have potentially important biases (e.g., based on characteristics listed in item 5). | See main manuscript “Discussion” |
| *For data inputs that contribute to the analysis but were not synthesized as part of the study:* | | |
| 7 | Describe and give sources for any other data inputs. | GBD 2019 Diseases and Injuries Collaborators. Global burden of  369 diseases and injuries in 204 countries and territories, 1990–2019: a systematic  analysis for the Global Burden of Disease Study 2019. |
| *For all data inputs:* | | |
| 8 | Provide all data inputs in a file format from which data can be efficiently extracted (e.g., a spreadsheet rather than a PDF), including all relevant meta-data listed in item 5. For any data inputs that cannot be shared because of ethical or legal reasons, such as third-party ownership, provide a contact name or the name of the institution that retains the right to the data. | http://ghdx.healthdata.org/gbd-2019 |
| Data analysis | | |
| 9 | Provide a conceptual overview of the data analysis method. A diagram may be helpful. | See main manuscript “Statistical analysis” |
| 10 | Provide a detailed description of all steps of the analysis, including mathematical formulae. This description should cover, as relevant, data cleaning, data pre-processing, data adjustments and weighting of data sources, and mathematical or statistical model(s). | GBD 2019 Diseases and Injuries Collaborators. Global burden of  369 diseases and injuries in 204 countries and territories, 1990–2019: a systematic  analysis for the Global Burden of Disease Study 2019. |
| 11 | Describe how candidate models were evaluated and how the final model(s) were selected. | GBD 2019 Diseases and Injuries Collaborators. Global burden of  369 diseases and injuries in 204 countries and territories, 1990–2019: a systematic  analysis for the Global Burden of Disease Study 2019. |
| 12 | Provide the results of an evaluation of model performance, if done, as well as the results of any relevant sensitivity analysis. | GBD 2019 Diseases and Injuries Collaborators. Global burden of  369 diseases and injuries in 204 countries and territories, 1990–2019: a systematic  analysis for the Global Burden of Disease Study 2019. |
| 13 | Describe methods for calculating uncertainty of the estimates. State which sources of uncertainty were, and were not, accounted for in the uncertainty analysis. | GBD 2019 Diseases and Injuries Collaborators. Global burden of  369 diseases and injuries in 204 countries and territories, 1990–2019: a systematic  analysis for the Global Burden of Disease Study 2019. |
| 14 | State how analytic or statistical source code used to generate estimates can be accessed. | http://ghdx.healthdata.org/gbd-2019/code |
| Results and Discussion | | |
| 15 | Provide published estimates in a file format from which data can be efficiently extracted. | GBD 2019 estimates are available online (http://vizhub.healthdata.org/gbd-compare and http://ghdx.healthdata.org/gbd-results-tool) |
| 16 | Report a quantitative measure of the uncertainty of the estimates (e.g. uncertainty intervals). | See main manuscript “Results” |
| 17 | Interpret results in light of existing evidence. If updating a previous set of estimates, describe the reasons for changes in estimates. | See main manuscript “Discussion” |
| 18 | Discuss limitations of the estimates. Include a discussion of any modelling assumptions or data limitations that affect interpretation of the estimates. | See main manuscript “Discussion” |

*This checklist should be used in conjunction with the GATHER statement and Explanation and Elaboration document, found on gather-statement.org*
