# Supplementary material for: Dietary Risk-Related Colorectal Cancer Burden: Estimates From 1990 to 2019
Source: Front Nutr. 2021 Aug 24;8:690663. doi: 10.3389/fnut.2021.690663 (PMC8421520; doi:10.3389/fnut.2021.690663)
Supplement: Supplementary file 3 [file Data_Sheet_3.zip › Supplemental tables/Table S1.docx]

**Table S1** Age-standardized summary exposure value rate of dietary risks related colorectal cancer among countries in 2019.

| **Location** | **Both (95%UI)** | **Female (95%UI)** | **Male (95%UI)** |
| --- | --- | --- | --- |
| Afghanistan | 30.57(24.37-39.97) | 30.05(24.6-37.55) | 31.03(23.14-44.02) |
| Albania | 59.91(43.03-72.25) | 45.68(23.98-65.94) | 74.5(61.17-81.25) |
| Algeria | 20.55(13.97-30.56) | 18.99(13.31-26.83) | 22.09(13.58-35.61) |
| American Samoa | 23.74(16.97-35.9) | 23.21(16.55-35.19) | 24.32(17.07-37.41) |
| Andorra | 32.53(21.85-47.84) | 27.8(19.45-41.92) | 37.03(22.46-55.01) |
| Angola | 31.43(17.46-61.51) | 31.6(17.48-61.83) | 31.26(17.27-61.5) |
| Antigua | 26.21(17.39-40.56) | 23.35(15.67-36.62) | 29.37(17.98-46.02) |
| Argentina | 40.01(25.9-55.42) | 35.7(24.12-52.69) | 44.6(27.09-59.8) |
| Armenia | 34.15(20.14-50.03) | 26.44(16.4-41.98) | 42.97(23.06-61.1) |
| Australia | 36.14(26.29-50.77) | 31.95(23.87-45.3) | 40.46(26.85-57.3) |
| Austria | 35.03(21.99-52.14) | 29.95(19.92-46.31) | 40.28(23.11-58.65) |
| Azerbaijan | 34.15(20.02-49.2) | 26.46(16.13-41.7) | 42.4(23.23-58.76) |
| Bahamas | 28.25(19.01-42.78) | 25.39(17.72-38.68) | 31.44(19.81-48.14) |
| Bahrain | 19.05(12.05-30.64) | 16.73(11.19-24.87) | 20.37(11.87-34.43) |
| Bangladesh | 38.13(24.73-54.24) | 37.69(24.55-55.28) | 38.65(24.86-54.25) |
| Barbados | 27.32(19.64-41.2) | 25.66(18.75-38.6) | 29.18(19.96-44.43) |
| Barbuda | 26.21(17.39-40.56) | 23.35(15.67-36.62) | 29.37(17.98-46.02) |
| Belarus | 31.73(22.41-46.91) | 27.26(20.27-39.39) | 36.74(23.12-56.95) |
| Belgium | 35.92(23.81-51.7) | 30.67(21.34-45.5) | 41.27(24.57-59.35) |
| Belize | 19.99(11.37-34.32) | 17.26(10.08-30.62) | 22.77(11.74-38.78) |
| Benin | 33.79(18.45-66.74) | 36.55(18.62-72.1) | 30.76(17.83-61.88) |
| Bermuda | 30.86(22.27-44.62) | 28.09(20.59-40.64) | 33.83(22.79-49.21) |
| Bhutan | 35.18(21.19-51.16) | 33.93(20.84-50.57) | 36.27(21.22-53.13) |
| Bolivia | 37.23(23.01-52.81) | 32.61(21.11-48.59) | 41.99(24-59.11) |
| Bosnia and Herzegovina | 56.31(40.11-68.9) | 42.68(21.26-62.89) | 70.55(57.92-77.48) |
| Botswana | 39.66(23.71-70.24) | 39.44(23.42-70.67) | 39.88(23.71-70.63) |
| Brazil | 39.27(25.43-53.72) | 35.62(24.57-50.06) | 43.26(25.42-59.22) |
| Brunei | 53.9(35.35-67.69) | 46.84(27.62-62.56) | 60.57(40.7-73.5) |
| Bulgaria | 65.18(48.93-76.21) | 50.6(30.21-68.21) | 80.01(66.59-87.05) |
| Burkina Faso | 34.17(21.74-65.06) | 33.81(21.38-64.95) | 34.59(21.86-65.88) |
| Burundi | 29.87(14.23-60.57) | 34.74(14.64-69.57) | 25.41(13.42-53.53) |
| Cabo Verde | 30.49(16.89-62.34) | 29.85(16.82-62.64) | 31.13(16.81-64.8) |
| Cambodia | 48.56(30.1-63.48) | 44.69(26.67-60.52) | 53.45(33.79-68.09) |
| Cameroon | 26.72(13.17-59.04) | 26.31(13.25-58.79) | 27.14(13.08-58.17) |
| Canada | 39.72(25.02-54.36) | 31.56(20.76-47.25) | 48.13(27.79-63.01) |
| Central African Republic | 37.15(23.7-66.72) | 37.42(23.54-68.2) | 36.88(23.57-66.34) |
| Chad | 35.66(23.13-66.38) | 35.25(22.68-66.69) | 36.07(23.17-66.95) |
| Chile | 42.31(26.36-58.6) | 36.07(23.53-52.32) | 48.92(28.28-67.19) |
| China | 77.37(69.58-81.93) | 75(65.95-80.62) | 79.85(72.97-84) |
| Colombia | 53.61(36.68-65.5) | 39.31(20.62-56.15) | 69.6(53.99-78.51) |
| Comoros | 33.73(17.96-65.58) | 38.21(18.11-74.23) | 29.03(16.73-57.97) |
| Cook Islands | 29.63(20.03-43.25) | 27.64(18-42.11) | 31.66(21.1-45.17) |
| Costa Rica | 43.48(23.77-62.24) | 36.09(18.8-57.38) | 51.76(27.87-69.99) |
| Croatia | 60.75(44.21-72.81) | 46.3(25.47-65.2) | 75.6(62.33-82.61) |
| Cuba | 22.76(14.1-36.84) | 20(12.65-32.8) | 25.58(14.43-41.2) |
| Cyprus | 36.25(22.98-52.85) | 29.86(19.97-45.85) | 42.95(24.62-61.7) |
| Czech Republic | 64.94(49.65-75.4) | 49.78(30.03-66.53) | 80.11(67.09-86.78) |
| Democratic Republic of the Congo | 26.04(18.24-47.87) | 25.84(18.03-46.72) | 26.26(18.08-47.95) |
| Denmark | 35.09(22.5-51.42) | 28.85(19.73-43.79) | 41.29(23.32-59.78) |
| Djibouti | 40.6(25.37-72.74) | 45.48(25.69-80.45) | 36.43(24.35-65.77) |
| Dominica | 20.42(11.46-35.19) | 17.33(10.18-30.42) | 23.4(11.71-40.82) |
| Dominican Republic | 21.77(12.89-36.19) | 18.93(11.56-32.42) | 24.62(13.11-40.7) |
| Ecuador | 34.46(20.63-49.97) | 29.39(18.07-45.42) | 39.76(22.37-56.48) |
| Egypt | 17.27(10.97-28.25) | 15.1(10.01-23.87) | 19.29(10.71-33.13) |
| El Salvador | 38.72(20.31-55.4) | 32.53(16.67-50.96) | 46.83(24.21-63.07) |
| Equatorial Guinea | 30.08(16.03-59.43) | 30.33(15.8-60.48) | 29.93(15.6-59.01) |
| Eritrea | 36.22(20.64-67.9) | 40.7(21.05-75.68) | 31.36(19.63-59.26) |
| Estonia | 26.12(20.06-35.97) | 23.8(18.53-31.3) | 28.6(20.03-43.61) |
| Eswatini | 37.5(21.71-68.48) | 37.58(21.59-68.76) | 37.39(21.49-67.42) |
| Ethiopia | 38.13(23.19-67.33) | 42.6(23.3-75.64) | 33.74(22.47-59.09) |
| Fiji | 35.22(25.4-49.27) | 33.5(23.91-48.73) | 37.17(26.57-50.62) |
| Finland | 40.04(26-56.3) | 32.38(22.97-48.25) | 47.59(27.35-65.79) |
| France | 37.37(27.02-52) | 33.03(25.05-45.99) | 41.95(27.87-59.11) |
| Gabon | 31.54(17.59-60.86) | 31.72(17.51-61.55) | 31.37(17.55-61.72) |
| Gambia | 33.61(20.96-66.76) | 33.35(20.62-67.08) | 33.9(21.01-66.34) |
| Georgia | 38.59(23.99-55.07) | 30.36(19.92-46.34) | 47.66(27.65-66.26) |
| Germany | 40.94(26.97-55.86) | 32.71(22.99-48.12) | 48.94(29.39-65.32) |
| Ghana | 32.82(13.02-66.94) | 32.59(12.97-67.7) | 33.08(13.03-67.04) |
| Greece | 33.13(20.54-48.59) | 27.16(18.27-42.32) | 39.29(21.56-56.64) |
| Greenland | 37.02(23.4-52.06) | 32.31(21.35-47.39) | 41.18(24.6-56.91) |
| Grenada | 23.9(15.03-38.24) | 21.03(13.47-34.53) | 26.75(15.47-42.83) |
| Grenadines | 24.25(15.27-38.94) | 21.36(13.73-34.63) | 27.05(15.72-43.64) |
| Guam | 29.45(19.51-42.86) | 27.44(18.07-41.54) | 31.52(20.61-45.15) |
| Guatemala | 39.07(20.13-55.81) | 32.67(16.34-51.32) | 46.73(23.13-63.04) |
| Guinea | 28.21(15.06-59.66) | 27.9(14.93-59.45) | 28.56(14.72-60.22) |
| Guinea-Bissau | 33.33(20.35-65.69) | 33.14(20.35-64.57) | 33.57(20.27-66.24) |
| Guyana | 25.69(17.25-39.98) | 22.93(15.76-36.53) | 28.67(17.68-44.59) |
| Haiti | 27.05(17.8-43.05) | 24.4(16.42-39.75) | 30.05(18.15-47.28) |
| Honduras | 40.03(21.87-56.13) | 33.12(17.69-50.39) | 47.87(25.27-64.18) |
| Hungary | 68.55(54.02-77.89) | 55.78(34.47-71.02) | 82.2(73.79-87.31) |
| Iceland | 39.74(27.34-54.74) | 33.03(24.31-46.84) | 46.23(27.96-63.51) |
| India | 40.93(25.91-55.09) | 36.49(23.09-51.82) | 45.25(28.99-58.62) |
| Indonesia | 45.39(27.07-61.38) | 41.85(23.2-59.52) | 49.07(30.07-64.08) |
| Iran | 18.79(12.8-27.87) | 17.67(12.63-24.42) | 19.88(11.85-32.69) |
| Iraq | 21.67(15.2-32.3) | 19.29(13.92-27.3) | 23.95(15.26-38.27) |
| Ireland | 33.8(23.98-48.91) | 28.59(21.52-40.7) | 39.25(24.52-58.81) |
| Israel | 30.27(16.81-47.05) | 23.86(14.45-40.15) | 36.81(18.34-55.23) |
| Italy | 43.67(27.85-58.27) | 36.68(22.08-53.46) | 50.83(33.13-64.96) |
| Ivory Coast | 32.39(16.87-65.94) | 32.26(16.72-66.74) | 32.49(16.74-65.45) |
| Jamaica | 23.57(14.67-37.88) | 20.93(13.21-34.39) | 26.35(14.96-42.12) |
| Japan | 44.1(27.73-58.95) | 38.95(23.83-54.67) | 49.31(31.26-63.52) |
| Jordan | 23.04(16.35-33.24) | 20.55(15.01-28.52) | 25.07(16.36-38.21) |
| Kazakhstan | 42.47(27.97-60.21) | 34.56(23.4-51.85) | 51.47(31.21-70.78) |
| Kenya | 31.68(21.31-51.64) | 40.63(21.35-73.49) | 22.09(16.74-31.44) |
| Kiribati | 29.56(20.61-42.03) | 27.93(19.27-40.83) | 31.88(21.96-44.51) |
| Kuwait | 28.69(17.67-43.24) | 23.43(15.48-36.51) | 33.03(18.13-49.38) |
| Kyrgyzstan | 38.89(25.86-52.71) | 31.86(22.16-46.01) | 46.6(28.29-61.21) |
| Laos | 44.97(26.5-60.08) | 40.86(22.38-57.82) | 49.23(29.91-64.81) |
| Latvia | 36.17(24.97-51.24) | 27.77(21.25-39.68) | 45.52(26.33-65.89) |
| Lebanon | 18.62(12.27-29.65) | 16.12(11.02-24.08) | 21.33(12.38-36.92) |
| Lesotho | 37.71(22.14-67.6) | 37.95(21.92-69.4) | 37.45(22.12-66.81) |
| Liberia | 33.12(20.37-65.17) | 32.87(20.2-65.18) | 33.36(20.22-64.61) |
| Libya | 21.55(14.43-33.47) | 18.83(12.97-27.69) | 24.07(14.88-40.03) |
| Lithuania | 32.42(22.38-50.24) | 30.98(21.65-47.24) | 34.02(22.69-53.51) |
| Luxembourg | 40.22(26.95-56.89) | 33.47(24.11-48.89) | 46.82(28.07-64.89) |
| Macedonia | 56.69(40.97-68.15) | 41.91(21.32-60.37) | 70.97(56.82-78.04) |
| Madagascar | 37.92(22.2-69.56) | 42.53(22.52-78.43) | 33.18(21.06-61.93) |
| Malawi | 34.72(18.14-67.25) | 39.1(18.36-75.59) | 29.87(17.26-58.56) |
| Malaysia | 44.59(24.78-61.56) | 41.4(20.5-62.9) | 47.67(28.07-63) |
| Maldives | 44.74(25.71-59.91) | 40.51(21.01-58.6) | 47.85(28.64-62.56) |
| Mali | 33.39(20.81-64.98) | 33.24(20.74-65.72) | 33.52(20.75-63.51) |
| Malta | 44.92(27.04-62.49) | 37.3(22.02-56.04) | 52.28(30.46-69.93) |
| Marshall Islands | 31.7(23-43.93) | 29.79(21.35-42.76) | 33.49(23.92-45.76) |
| Mauritania | 36.27(23.48-68.13) | 35.92(23.16-68.43) | 36.64(23.47-69.09) |
| Mauritius | 46.15(27.15-61.62) | 42.46(23.34-60.09) | 50.14(30.62-65.04) |
| Mexico | 33.22(19.87-49.18) | 31.26(18.92-47.25) | 35.42(20.34-52.11) |
| Micronesia | 31.41(22.93-43.66) | 29.64(21.04-42.35) | 33.44(24.23-45.56) |
| Moldova | 28.23(19.05-43.26) | 23.89(16.97-36.2) | 32.94(19.5-51.5) |
| Monaco | 33.43(19.78-51.24) | 27.07(17.27-43.87) | 40.06(20.65-59.59) |
| Mongolia | 52.76(39.05-68.65) | 44.66(35.09-61) | 61.66(42.38-79.35) |
| Montenegro | 58(41.44-69.8) | 43.79(22.89-62.21) | 72.7(58.65-79.55) |
| Morocco | 18.52(11.96-29.82) | 16.33(11.05-25.75) | 20.72(11.94-35.42) |
| Mozambique | 38.01(21.67-70.47) | 42.39(21.7-78.36) | 32.93(20.64-62.55) |
| Myanmar | 47.14(28.95-62.61) | 43.49(24.74-60.55) | 51.61(32.35-66.55) |
| Namibia | 40.38(24.51-70.72) | 40.25(24.34-71.03) | 40.49(24.52-69.91) |
| Nauru | 31.09(21.27-45.79) | 29.26(19.68-44.04) | 33.51(22.82-48.42) |
| Nepal | 35.29(21.63-50.92) | 34.06(21.37-49.42) | 36.83(21.67-53.4) |
| Netherlands | 38.17(25.31-54.34) | 31.98(22.63-46.65) | 44.38(26.38-62.67) |
| New Zealand | 35.04(24.27-49.76) | 31.7(22.81-46.12) | 38.7(24.55-55.31) |
| Nicaragua | 45.16(27.05-61.38) | 39.82(24.09-58.25) | 51.22(29.31-66.91) |
| Niger | 33.69(20.99-64.65) | 33.42(20.59-65.59) | 33.98(21.14-65.19) |
| Nigeria | 27.43(16.16-56.17) | 26.72(15.83-55.08) | 28.21(16.2-57.62) |
| Niue | 30.89(20.83-45.2) | 28.86(19.31-43.05) | 33.24(22.23-47.97) |
| North Korea | 56.45(38.3-67.62) | 53.57(33.84-66.66) | 59.86(41.92-70.79) |
| Northern Mariana Islands | 29.93(20.42-43.9) | 27.93(18.29-42.57) | 31.83(21.72-45.43) |
| Norway | 35.66(24.6-50.7) | 28.67(20.82-42.31) | 42.39(26.12-59.95) |
| Oman | 20.53(13.7-30.87) | 17.81(12.56-26.27) | 21.74(13.68-34.03) |
| Pakistan | 40.15(25.78-57.11) | 39.12(25.15-57.33) | 41.16(26.1-57.96) |
| Palau | 30.4(20.83-43.71) | 28.48(18.99-42.8) | 32.36(22.15-45.14) |
| Palestine | 22.67(16.43-32.16) | 20.55(14.93-27.82) | 24.76(16.49-37.31) |
| Panama | 46.97(28.96-62.82) | 40.65(25.33-57.54) | 53.35(30.76-69.99) |
| Papua New Guinea | 30.75(21.71-43.07) | 28.92(20.28-42.04) | 32.35(22.4-45.03) |
| Paraguay | 38.67(23.94-54.8) | 35.22(23.03-51.69) | 42.11(24.07-59.38) |
| Peru | 31.97(17.52-47.77) | 27.09(15.59-42.68) | 37.11(19-54.08) |
| Philippines | 45.69(28.49-60.53) | 42.29(24.83-58.59) | 49.34(31.31-63.95) |
| Poland | 53.4(36.9-67.34) | 40.76(25.2-58.29) | 66.61(48.61-77.58) |
| Portugal | 41.09(25.85-56.06) | 33.21(21.64-48.78) | 49.6(29.16-65.58) |
| Puerto Rico | 24.74(15.64-39.37) | 22(14.2-35.44) | 27.83(15.95-44.85) |
| Qatar | 18.19(10.93-29.71) | 14.72(9.9-22.4) | 19.15(10.8-32.54) |
| Republic of Congo | 34.11(20.42-64.31) | 34.41(20.28-65.58) | 33.8(20.01-63.71) |
| Romania | 62.66(46.5-74.74) | 48.42(27.36-66.53) | 77.22(64.12-84.64) |
| Russia | 44.18(28.28-60.89) | 37.49(23.36-56.3) | 52.11(33.41-67.6) |
| Rwanda | 26.88(10.75-58.61) | 30.98(10.66-66.86) | 21.96(9.92-49.51) |
| Saint Kitts and Nevis | 32.13(23.35-47.95) | 28.82(21.67-42.51) | 35.47(23.54-54.42) |
| Saint Lucia | 28.4(19.23-43.06) | 25.48(17.84-39.09) | 31.43(19.66-48) |
| Saint Vincent | 24.25(15.27-38.94) | 21.36(13.73-34.63) | 27.05(15.72-43.64) |
| Samoa | 23.76(18.92-31.21) | 23.56(18.64-31.23) | 23.98(18.81-32.05) |
| San Marino | 36.01(22.57-52.64) | 29.83(20.05-45.54) | 42.93(23.61-62.1) |
| Sao Tome and Principe | 25.45(12.3-57) | 25.31(12.09-59.01) | 25.59(11.89-56.66) |
| Saudi Arabia | 21.38(14.6-32.1) | 18.87(13.46-26.59) | 23.02(14.57-36.43) |
| Senegal | 32.39(19.62-63.3) | 32.07(19.44-64.74) | 32.74(19.6-63.79) |
| Serbia | 58.22(41.99-69.73) | 43.66(23.01-62.33) | 73.02(59.06-79.82) |
| Seychelles | 30.54(21.24-41.17) | 28.15(18.95-39.56) | 33.07(23.13-43.44) |
| Sierra Leone | 33.19(20.15-65.77) | 32.99(19.96-65.68) | 33.39(20.04-66.17) |
| Singapore | 48.86(31.26-61.79) | 42.81(24.43-58.26) | 54.62(36.49-67.49) |
| Slovakia | 65.57(49.84-77.16) | 51.09(30.26-69.05) | 80.51(68.33-87.39) |
| Slovenia | 63.55(47.14-75.18) | 48.65(27.65-67.56) | 77.99(64.73-85) |
| Solomon Islands | 29.81(21.23-41.95) | 28.14(19.56-41.14) | 31.42(22.32-43.12) |
| Somalia | 42.21(26.7-73.5) | 46.48(26.9-82.54) | 37.4(25.72-64.84) |
| South Africa | 34.55(23.51-62.47) | 33.98(23.05-62.96) | 35.07(23.46-61.04) |
| South Korea | 56.83(38.53-68.54) | 49.72(30.43-63.65) | 63.41(45.61-74.81) |
| South Sudan | 33.48(17.46-65.45) | 38(17.82-73.43) | 28.94(16.44-58.19) |
| Spain | 36.51(22.98-51.56) | 29.52(19.6-44.11) | 43.47(24.97-59.96) |
| Sri Lanka | 44.02(24.84-60.12) | 40.51(21.04-58.93) | 48.1(28.06-63.36) |
| Sudan | 24.22(17.48-35.66) | 21.99(16-30.52) | 26.56(17.58-41.11) |
| Suriname | 26.02(17.03-40.32) | 23.2(15.7-36.13) | 29.06(17.68-45.03) |
| Sweden | 36.72(24.55-52.31) | 30.98(21.96-46.7) | 42.31(26.02-59.24) |
| Switzerland | 39.29(26.3-54.32) | 32.78(23.69-46.86) | 45.69(28.25-62.07) |
| Syria | 21.36(15.08-31.56) | 19.42(13.81-27.52) | 23.76(15.14-37.65) |
| Taiwan | 35.29(21.34-51.54) | 35.72(21.21-52.5) | 34.83(20.94-51.57) |
| Tajikistan | 38.33(24.44-54) | 30.41(20.44-46.19) | 46.51(27.74-62.84) |
| Tanzania | 39.66(18.26-73.59) | 38.7(17.77-73.63) | 40.71(18.48-74.73) |
| Thailand | 41.64(23.49-56.38) | 37.93(19.89-54.03) | 45.78(27.1-60.59) |
| Timor-Leste | 49.48(29.83-66.73) | 44.36(25.44-61.96) | 54.63(33.24-72.49) |
| Tobago | 28.48(19.29-43.48) | 24.95(17.54-38.69) | 32.04(19.68-49.39) |
| Togo | 32.52(20.1-63.77) | 32.22(19.9-64.37) | 32.89(20.26-64.12) |
| Tokelau | 31.19(21.38-44.17) | 29.19(20.1-42.39) | 33.07(22.86-46) |
| Tonga | 31.79(22.33-45.86) | 29.92(20.41-44.48) | 33.9(23.9-47.29) |
| Trinidad | 28.48(19.29-43.48) | 24.95(17.54-38.69) | 32.04(19.68-49.39) |
| Tunisia | 18.2(11.95-28.21) | 16.22(11.09-24.68) | 20.27(12.03-33.61) |
| Turkey | 13.27(8.49-21.07) | 12.66(8.42-19.63) | 13.87(8.04-25.26) |
| Turkmenistan | 41.99(27.53-58.57) | 33.75(23.03-50.2) | 50.22(30.58-68.46) |
| Tuvalu | 31.78(22.61-44.91) | 30.01(21.08-43.56) | 33.82(24.07-47.29) |
| Uganda | 30.1(14.02-61.5) | 34.46(14.16-69.78) | 25.03(12.86-52.19) |
| UK | 32.64(24.67-44.91) | 29.62(22.73-40.65) | 35.69(24.85-49.58) |
| Ukraine | 29.3(20.21-44.13) | 24.96(18.4-37.26) | 34.26(20.78-53.33) |
| United Arab Emirates | 21.33(13.87-33.13) | 17.97(12.6-25.52) | 22.37(13.62-35.79) |
| United States Virgin Islands | 22.99(14.02-37.71) | 20.29(12.61-34.1) | 26.16(14.4-43.25) |
| Uruguay | 44(28.72-60.01) | 38.68(25.93-55.94) | 49.88(31.04-66.15) |
| USA | 39.46(26.22-53.84) | 34.35(23.83-49.21) | 44.76(28.28-59.21) |
| Uzbekistan | 37.81(23.89-53.75) | 30.02(19.59-46.22) | 46.24(26.96-63.38) |
| Vanuatu | 32.7(22.54-46.67) | 30.71(20.91-45.46) | 34.51(23.76-48.77) |
| Venezuela | 44.92(26.07-61.83) | 37.38(21.69-54.15) | 53.07(29.09-71.43) |
| Vietnam | 47.52(28.39-63.8) | 43.29(24.49-60.71) | 52.41(31.88-68.48) |
| Yemen | 27.59(21.7-37.32) | 25.59(20.65-33.17) | 29.62(22.04-42.52) |
| Zambia | 37.78(22.73-68.89) | 42.12(22.88-76.75) | 33.41(21.99-61.64) |
| Zimbabwe | 42.25(22.37-73.4) | 42.1(22.22-73.65) | 42.59(22.43-74.25) |
