# Supplementary material for: Dietary Risk-Related Colorectal Cancer Burden: Estimates From 1990 to 2019
Source: Front Nutr. 2021 Aug 24;8:690663. doi: 10.3389/fnut.2021.690663 (PMC8421520; doi:10.3389/fnut.2021.690663)
Supplement: Supplementary file 3 [file Data_Sheet_3.zip › Supplemental tables/Table S10.docx]

**Table S10** DALYs, ASRs and change trends of colorectal cancer DALYs attributable to diet low in whole grains between 1990 and 2019 by SDI, regions and sex.

| **Location** | **Sex** | **DALYs (No.×1000, 95%UI)** | | **ASR (95%UI)** | | **EAPC (95%CI)** |
| --- | --- | --- | --- | --- | --- | --- |
|  |  | **1990** | **2019** | **1990** | **2019** | **1990-2019** |
| Global | Both | 1974.78(782.17-2571.76) | 3806.86(1462.36-5024.32) | 49.42(19.54-64.27) | 46.3(17.81-61.05) | -0.29(-0.35--0.23) |
| Global | Female | 936.39(372.96-1216.5) | 1592.83(618.09-2121.28) | 43.9(17.49-56.94) | 36.65(14.22-48.81) | -0.76(-0.82--0.71) |
| Global | Male | 1038.39(406.93-1353.22) | 2214.03(849.24-2921.39) | 56.07(21.89-72.9) | 57.13(21.93-75.37) | 0.06(-0.01-0.13) |
| **Sociodemographic Index** | | | | | | |
| High SDI | Both | 705.19(264.42-935.35) | 966.99(360-1270.52) | 68.7(25.75-91.14) | 54.39(20.19-71.44) | -0.96(-1.02--0.89) |
| High SDI | Female | 327.82(123.04-434.43) | 410.47(153.47-545.75) | 56.19(21.08-74.47) | 42.58(15.88-56.46) | -1.12(-1.19--1.05) |
| High SDI | Male | 377.37(141.38-500.5) | 556.52(206.96-729.09) | 85.01(31.85-112.59) | 67.64(25.15-88.59) | -0.94(-1--0.88) |
| High-middle SDI | Both | 710.28(297.29-897.97) | 1203.31(474.81-1580.37) | 65.5(27.47-82.88) | 59.56(23.5-78.25) | -0.48(-0.57--0.38) |
| High-middle SDI | Female | 345.8(146.17-437.77) | 494.37(197.64-645.22) | 57.46(24.28-72.73) | 45.04(18.03-58.79) | -1.07(-1.17--0.98) |
| High-middle SDI | Male | 364.48(152.08-462.47) | 708.93(275.97-944.76) | 76.44(31.98-96.81) | 76.9(30.06-102.37) | -0.06(-0.17-0.05) |
| Low SDI | Both | 59.63(23.98-81.94) | 148.77(58.95-199.46) | 23.33(9.45-31.99) | 26.55(10.53-35.49) | 0.44(0.39-0.49) |
| Low SDI | Female | 27.21(11.23-39.9) | 70.66(27.87-95.01) | 21.26(8.75-31.13) | 24.63(9.68-33.16) | 0.5(0.44-0.57) |
| Low SDI | Male | 32.42(13.11-46.86) | 78.11(31.07-106.69) | 25.3(10.21-36.33) | 28.55(11.35-38.74) | 0.42(0.38-0.45) |
| Low-middle SDI | Both | 150.46(59.42-199.94) | 439.96(169.88-592.13) | 23.23(9.22-30.77) | 30.88(11.93-41.38) | 0.99(0.95-1.02) |
| Low-middle SDI | Female | 72.24(29.09-98.86) | 205.92(79.89-281.87) | 22.49(9.03-30.6) | 27.97(10.83-38.28) | 0.69(0.61-0.77) |
| Low-middle SDI | Male | 78.22(30.7-108.65) | 234.04(88.96-309.37) | 23.95(9.43-33.24) | 34.02(12.94-45.13) | 1.27(1.22-1.33) |
| Middle SDI | Both | 348.21(132.91-463.82) | 1045.73(399.54-1407.76) | 31.15(11.95-41.49) | 40.84(15.62-54.89) | 1.13(0.98-1.28) |
| Middle SDI | Female | 162.83(61.46-216.92) | 410.45(159.13-560.6) | 28.86(10.97-38.27) | 31.11(12.07-42.36) | 0.31(0.23-0.39) |
| Middle SDI | Male | 185.38(70.73-249.46) | 635.28(243.94-867.35) | 33.59(12.81-45.19) | 51.34(19.75-69.85) | 1.77(1.57-1.97) |
| **Region** | | | | | | |
| Africa | Both | 76.92(30.51-102.99) | 200.09(78.23-273.42) | 25.26(9.97-33.6) | 29.72(11.59-40.47) | 0.61(0.57-0.65) |
| Africa | Female | 35.76(14.42-48.97) | 93.14(36.3-128.75) | 23.26(9.38-31.89) | 26.85(10.43-37.11) | 0.59(0.53-0.65) |
| Africa | Male | 41.16(16.44-56.5) | 106.94(41.94-146.31) | 27.26(10.98-37.45) | 32.79(12.84-44.32) | 0.65(0.61-0.68) |
| America | Both | 335.09(126.26-440.47) | 578.62(214.96-772.21) | 54.84(20.66-72.14) | 46.19(17.17-61.67) | -0.67(-0.73--0.61) |
| America | Female | 160.1(60.67-211.66) | 262.89(98.09-350.42) | 47.57(18.03-62.88) | 38.95(14.54-51.91) | -0.75(-0.81--0.7) |
| America | Male | 174.99(65.75-228.96) | 315.74(116.83-418.95) | 63.64(23.92-83.24) | 54.42(20.14-72.19) | -0.64(-0.71--0.56) |
| Asia | Both | 736.74(282.07-979.82) | 2028.55(771.48-2706.81) | 34.36(13.23-45.71) | 41.72(15.88-55.69) | 0.78(0.65-0.91) |
| Asia | Female | 335.43(128.17-446.11) | 799.16(304.96-1078.75) | 30.96(11.8-41.17) | 31.93(12.2-43.04) | 0.07(-0.01-0.14) |
| Asia | Male | 401.31(152.38-541.52) | 1229.38(473.52-1669.75) | 38.11(14.48-51.43) | 52.29(20.09-70.77) | 1.31(1.13-1.48) |
| Europe | Both | 823.02(339.14-1043.96) | 993.39(393.17-1282.05) | 80.37(33.13-101.9) | 67.37(26.65-86.84) | -0.89(-1--0.77) |
| Europe | Female | 403.74(167.18-513.38) | 434.94(172.88-568.3) | 67.6(28.04-85.91) | 52.37(20.83-68.64) | -1.18(-1.29--1.07) |
| Europe | Male | 419.28(171.02-532.89) | 558.46(218.49-714.29) | 99.09(40.34-125.88) | 86.24(33.8-110.45) | -0.73(-0.85--0.62) |
| Andean Latin America | Both | 5.83(2.22-7.7) | 18.74(6.82-26.83) | 27.17(10.34-35.92) | 33.03(12.05-47.31) | 0.86(0.71-1.01) |
| Andean Latin America | Female | 3.04(1.17-4.09) | 9.65(3.62-13.79) | 27.85(10.72-37.39) | 32.85(12.32-46.85) | 0.63(0.48-0.78) |
| Andean Latin America | Male | 2.78(1.05-3.71) | 9.09(3.3-12.89) | 26.41(9.94-35.24) | 33.14(12.07-47.04) | 1.11(0.95-1.28) |
| Australasia | Both | 18.5(6.89-24.74) | 25.09(9.28-33.51) | 80.16(29.8-106.92) | 53.5(19.82-71.43) | -1.74(-1.88--1.6) |
| Australasia | Female | 8.36(3.09-11.24) | 10.98(4.15-14.81) | 67.49(24.97-90.48) | 44.24(16.68-59.57) | -1.74(-1.86--1.62) |
| Australasia | Male | 10.14(3.79-13.54) | 14.11(5.24-18.85) | 95.23(35.62-127.18) | 63.64(23.6-84.86) | -1.78(-1.95--1.62) |
| Caribbean | Both | 12.24(4.62-15.94) | 26.99(10-37.08) | 46.43(17.49-60.35) | 52.25(19.34-71.74) | 0.45(0.38-0.52) |
| Caribbean | Female | 6.25(2.36-8.14) | 13.15(4.77-18.45) | 45.87(17.33-59.74) | 48.04(17.42-67.45) | 0.18(0.12-0.23) |
| Caribbean | Male | 6(2.26-7.69) | 13.84(5.18-18.98) | 46.95(17.71-60.24) | 56.79(21.31-77.48) | 0.71(0.62-0.8) |
| Central Asia | Both | 30.66(15.18-37.82) | 42.14(20.63-53.24) | 61.47(30.49-75.72) | 54.32(26.65-68.16) | -0.32(-0.49--0.15) |
| Central Asia | Female | 14.96(7.44-18.47) | 19.82(9.77-25.17) | 53.27(26.52-65.75) | 46.39(22.73-58.02) | -0.4(-0.53--0.27) |
| Central Asia | Male | 15.7(7.72-19.34) | 22.32(10.92-28.11) | 72.61(35.78-89.2) | 64.63(31.48-80.91) | -0.26(-0.47--0.05) |
| Central Europe | Both | 121.67(47.51-156.29) | 170.36(63.16-228.24) | 82.46(32.21-105.83) | 82.78(30.68-111.14) | -0.01(-0.1-0.07) |
| Central Europe | Female | 54.99(21.53-70.53) | 67.75(25.34-91.3) | 66.68(26.09-85.54) | 58.84(21.89-79.44) | -0.52(-0.59--0.45) |
| Central Europe | Male | 66.69(25.87-85.63) | 102.61(38.15-137.81) | 102.99(40-132.23) | 112.99(41.97-151.74) | 0.35(0.24-0.46) |
| Central Latin America | Both | 19.65(7.34-26.31) | 72.82(26.78-101.12) | 21.98(8.23-29.44) | 30.22(11.11-41.96) | 1.09(1.05-1.14) |
| Central Latin America | Female | 10.12(3.8-13.68) | 34.23(12.64-48.76) | 22.12(8.31-29.93) | 26.57(9.82-37.79) | 0.65(0.6-0.7) |
| Central Latin America | Male | 9.53(3.53-12.67) | 38.59(13.98-54.24) | 21.81(8.09-28.99) | 34.35(12.47-48.16) | 1.55(1.48-1.61) |
| Central Sub-Saharan Africa | Both | 6.18(2.36-9.06) | 14.77(5.36-21.56) | 25.08(9.54-36.67) | 25.11(9.02-36.98) | -0.08(-0.29-0.13) |
| Central Sub-Saharan Africa | Female | 2.74(1.04-4.15) | 6.74(2.35-10.04) | 20.89(8.02-31.3) | 21.35(7.34-32.1) | 0.04(-0.13-0.21) |
| Central Sub-Saharan Africa | Male | 3.45(1.3-5.27) | 8.04(2.77-12.82) | 29.82(11.22-45.5) | 29.87(10.65-47.67) | -0.12(-0.36-0.11) |
| East Asia | Both | 362.23(134.83-481.56) | 1068.79(398.54-1473.38) | 38.29(14.27-51.08) | 51.39(19.19-70.55) | 1.35(1.1-1.61) |
| East Asia | Female | 163.44(60.37-222.47) | 368.65(136.83-517.19) | 34.33(12.68-46.55) | 34.41(12.76-48.21) | 0.09(-0.09-0.27) |
| East Asia | Male | 198.8(73.26-270.45) | 700.13(257.72-978.5) | 43.01(15.89-58.35) | 70.14(25.83-98.19) | 2.2(1.89-2.52) |
| Eastern Europe | Both | 235.45(102.94-295.81) | 242.4(91.62-319.19) | 83.59(36.48-104.96) | 72.08(27.29-94.93) | -1.07(-1.31--0.82) |
| Eastern Europe | Female | 127.68(55.59-160.49) | 118.86(45.84-161.27) | 73.08(31.94-91.84) | 58.43(22.55-79.64) | -1.32(-1.54--1.09) |
| Eastern Europe | Male | 107.77(46.95-135.34) | 123.54(46.21-165.31) | 103.57(44.8-130.01) | 93.54(35.06-125.22) | -0.9(-1.16--0.64) |
| Eastern Sub-Saharan Africa | Both | 20.47(7.86-28.31) | 51.49(18.83-72.91) | 24.75(9.51-34.35) | 28.41(10.42-40.03) | 0.49(0.42-0.56) |
| Eastern Sub-Saharan Africa | Female | 9.43(3.61-14.07) | 23.97(8.89-34.02) | 22.09(8.5-32.75) | 25.29(9.34-35.52) | 0.48(0.39-0.57) |
| Eastern Sub-Saharan Africa | Male | 11.03(4.24-16.75) | 27.52(9.89-39.96) | 27.42(10.54-40.81) | 31.86(11.41-46.03) | 0.54(0.49-0.6) |
| High-income Asia Pacific | Both | 108.01(39.79-148.35) | 194.59(72.7-259.32) | 53.32(19.65-73.31) | 47.34(17.81-63.35) | -0.51(-0.61--0.4) |
| High-income Asia Pacific | Female | 46.48(17.38-64.64) | 79.79(29.82-107.99) | 41.63(15.58-57.85) | 34.72(12.92-46.76) | -0.74(-0.81--0.67) |
| High-income Asia Pacific | Male | 61.53(22.78-84.01) | 114.8(43.36-153.64) | 68.52(25.37-93.54) | 61.54(23.27-82.53) | -0.46(-0.6--0.32) |
| High-income North America | Both | 229.56(85.19-304.42) | 308.08(114.91-407.6) | 66.97(24.83-88.84) | 52.62(19.64-69.82) | -1(-1.1--0.91) |
| High-income North America | Female | 108.22(40.33-144.14) | 134.96(50.54-179.14) | 55.63(20.69-74.24) | 42.9(15.98-57.13) | -1.05(-1.13--0.96) |
| High-income North America | Male | 121.33(45-159.22) | 173.12(64.42-229.76) | 81.53(30.24-106.97) | 63.5(23.59-84.35) | -1.06(-1.17--0.94) |
| North Africa and Middle East | Both | 71.35(33.25-93.64) | 199.01(92.74-257.62) | 38.49(17.92-50.38) | 43.29(20.2-55.98) | 0.53(0.33-0.73) |
| North Africa and Middle East | Female | 33.81(15.91-45.69) | 88.72(41.05-116.53) | 37.04(17.36-49.53) | 39.65(18.58-51.97) | 0.35(0.18-0.53) |
| North Africa and Middle East | Male | 37.54(18.19-52.39) | 110.29(51.05-142.52) | 39.86(19.21-55.27) | 46.79(21.65-60.51) | 0.69(0.47-0.92) |
| Oceania | Both | 1.01(0.38-1.39) | 2.66(0.98-3.77) | 30.36(11.38-41.62) | 33.77(12.69-47.34) | 0.36(0.29-0.43) |
| Oceania | Female | 0.45(0.17-0.63) | 1.17(0.43-1.66) | 28.03(10.84-39.52) | 30.88(11.54-43.35) | 0.33(0.25-0.41) |
| Oceania | Male | 0.56(0.22-0.81) | 1.5(0.55-2.16) | 32.61(12.56-46.82) | 36.56(13.58-52.08) | 0.38(0.31-0.44) |
| South Asia | Both | 112.18(44.17-152.1) | 350.06(136.9-477.76) | 18.37(7.29-24.9) | 23.99(9.37-32.61) | 0.79(0.67-0.91) |
| South Asia | Female | 52.7(21-74.25) | 173.16(65.55-241.76) | 17.8(7.17-25.14) | 23.42(8.88-32.77) | 0.78(0.61-0.94) |
| South Asia | Male | 59.48(23.44-82.87) | 176.9(70.07-246.99) | 18.91(7.5-26.33) | 24.59(9.71-34.3) | 0.82(0.72-0.91) |
| Southeast Asia | Both | 85.77(32.28-120.62) | 269.59(103.35-399.21) | 30.46(11.52-42.72) | 41.98(16.04-62.05) | 1.01(0.94-1.09) |
| Southeast Asia | Female | 40.23(15.11-58.39) | 111.28(43.02-172.35) | 27.2(10.16-39.17) | 32.94(12.7-50.86) | 0.56(0.48-0.65) |
| Southeast Asia | Male | 45.55(17.36-64.64) | 158.31(59.41-230.81) | 34.15(13.02-48.43) | 52.31(19.43-76.97) | 1.38(1.32-1.45) |
| Southern Latin America | Both | 37.17(15.73-47.02) | 62.61(23.1-80.95) | 80.41(34.01-101.68) | 76.29(28.13-98.48) | -0.18(-0.25--0.12) |
| Southern Latin America | Female | 16.79(6.99-21.47) | 28.04(10.44-36.82) | 66.08(27.58-84.46) | 61.48(22.91-80.6) | -0.25(-0.32--0.18) |
| Southern Latin America | Male | 20.38(8.59-25.75) | 34.57(12.89-44.37) | 97.87(41.35-123.42) | 94.3(35.22-121.28) | -0.12(-0.19--0.06) |
| Southern Sub-Saharan Africa | Both | 8.08(3.08-11.76) | 18.86(6.86-26.88) | 27.56(10.48-40.58) | 31.86(11.65-45.6) | 0.59(0.36-0.82) |
| Southern Sub-Saharan Africa | Female | 3.76(1.47-5.44) | 8.54(3.16-12.45) | 23.23(9.12-34.21) | 25.63(9.49-37.52) | 0.64(0.48-0.79) |
| Southern Sub-Saharan Africa | Male | 4.32(1.59-6.45) | 10.32(3.8-14.77) | 32.81(12.03-49.68) | 40.23(14.93-57.63) | 0.64(0.3-0.97) |
| Tropical Latin America | Both | 33.02(12.35-43.55) | 94.33(35.16-126.83) | 34.06(12.75-44.98) | 38.27(14.27-51.49) | 0.46(0.29-0.63) |
| Tropical Latin America | Female | 16.75(6.39-22.07) | 45.02(16.72-61.46) | 32.86(12.54-43.33) | 33.74(12.53-46.06) | 0.14(-0.03-0.32) |
| Tropical Latin America | Male | 16.27(5.97-21.44) | 49.3(18.35-65.74) | 35.37(13.02-46.72) | 43.65(16.25-58.35) | 0.79(0.62-0.97) |
| Western Europe | Both | 434.95(173.92-559.86) | 520.2(207.11-667.34) | 77.37(30.91-99.55) | 60.59(24.1-77.45) | -1.05(-1.19--0.92) |
| Western Europe | Female | 207.07(82.76-267.2) | 223.29(89.52-288.14) | 63.86(25.53-82.43) | 47.19(18.79-60.71) | -1.26(-1.39--1.14) |
| Western Europe | Male | 227.88(91-292.67) | 296.91(117.9-380.04) | 95.66(38.21-122.79) | 76.14(30.25-97.26) | -0.99(-1.13--0.85) |
| Western Sub-Saharan Africa | Both | 20.8(8.07-29.5) | 53.29(20.23-73.7) | 22.92(8.92-32.32) | 27.13(10.25-37.27) | 0.75(0.66-0.83) |
| Western Sub-Saharan Africa | Female | 9.13(3.56-13.32) | 25.05(9.21-35.45) | 20.68(8.05-30.22) | 24.62(9.11-34.67) | 0.8(0.68-0.92) |
| Western Sub-Saharan Africa | Male | 11.67(4.46-17.05) | 28.24(10.68-39.74) | 25.01(9.59-36.35) | 29.84(11.23-41.75) | 0.76(0.68-0.84) |

ASDR, age-standardized death rate; DALYs, disability-adjusted life years; SDI, socio-demographic index; UI, uncertainty interval.
