# Supplementary material for: Dietary Risk-Related Colorectal Cancer Burden: Estimates From 1990 to 2019
Source: Front Nutr. 2021 Aug 24;8:690663. doi: 10.3389/fnut.2021.690663 (PMC8421520; doi:10.3389/fnut.2021.690663)
Supplement: Supplementary file 3 [file Data_Sheet_3.zip › Supplemental tables/Table S11.docx]

**Table S11** Deaths, ASDRs and change trends of colorectal cancer attributable to diet low in fiber between 1990 and 2019 by SDI, regions and sex.

| **Location** | **Sex** | **Deaths (95%UI)** | | **ASDR (95%UI)** | | **EAPC (95%CI)** |
| --- | --- | --- | --- | --- | --- | --- |
|  |  | **1990** | **2019** | **1990** | **2019** | **1990-2019** |
| Global | Both | 12547.51(4964.17-23333.8) | 20498.8(8205.27-39828.84) | 0.36(0.14-0.66) | 0.26(0.11-0.51) | -1.13(-1.23--1.02) |
| Global | Female | 6422.12(2520.95-12173.21) | 9742.16(3951.83-18875.16) | 0.33(0.13-0.61) | 0.22(0.09-0.43) | -1.41(-1.53--1.3) |
| Global | Male | 6125.39(2441.05-11276.21) | 10756.64(4253.64-20675.42) | 0.4(0.16-0.73) | 0.31(0.12-0.59) | -0.88(-0.98--0.79) |
| **Sociodemographic Index** | | | | | | |
| High SDI | Both | 5662.69(2153.87-10850.41) | 7284.34(2772.39-14363.77) | 0.54(0.21-1.03) | 0.35(0.13-0.7) | -1.5(-1.54--1.47) |
| High SDI | Female | 2986.31(1137.67-5678.57) | 3672.2(1469.7-7186.15) | 0.46(0.18-0.88) | 0.3(0.12-0.59) | -1.57(-1.62--1.52) |
| High SDI | Male | 2676.38(1009-5100.53) | 3612.14(1391.05-7111.88) | 0.65(0.25-1.24) | 0.42(0.16-0.82) | -1.56(-1.59--1.52) |
| High-middle SDI | Both | 2725.33(988.2-5696.46) | 4441.79(1739.74-9415.19) | 0.28(0.1-0.57) | 0.22(0.09-0.47) | -0.92(-1.17--0.68) |
| High-middle SDI | Female | 1398.22(527.13-2980.74) | 2037.06(778.87-4326.98) | 0.24(0.09-0.51) | 0.18(0.07-0.38) | -1.38(-1.64--1.12) |
| High-middle SDI | Male | 1327.1(502.89-2732.57) | 2404.73(959.56-5026.22) | 0.33(0.12-0.68) | 0.29(0.11-0.6) | -0.57(-0.8--0.33) |
| Low SDI | Both | 233.8(91.89-475.91) | 556.09(219.15-1070.64) | 0.11(0.05-0.23) | 0.12(0.05-0.23) | 0.12(-0.03-0.28) |
| Low SDI | Female | 106.14(42.2-229.66) | 271.37(103.91-520.71) | 0.1(0.04-0.22) | 0.11(0.04-0.22) | 0.28(0.12-0.44) |
| Low SDI | Male | 127.66(50.53-250.26) | 284.72(118.36-559.23) | 0.13(0.05-0.24) | 0.13(0.05-0.25) | 0.01(-0.14-0.17) |
| Low-middle SDI | Both | 1250.02(566.72-2075.89) | 2771.05(1209.95-5053.62) | 0.23(0.11-0.38) | 0.22(0.1-0.4) | -0.28(-0.42--0.15) |
| Low-middle SDI | Female | 604.43(275.84-1033.6) | 1363.25(570.94-2444.32) | 0.22(0.1-0.39) | 0.21(0.09-0.37) | -0.45(-0.59--0.31) |
| Low-middle SDI | Male | 645.59(293.83-1072.6) | 1407.8(603.91-2602.52) | 0.24(0.11-0.39) | 0.24(0.1-0.44) | -0.11(-0.26-0.04) |
| Middle SDI | Both | 2668.13(1153.22-4500.21) | 5432.99(2335.68-9959.01) | 0.28(0.12-0.47) | 0.23(0.1-0.43) | -0.61(-0.75--0.46) |
| Middle SDI | Female | 1323.13(553.3-2258.69) | 2392.39(1024.77-4305.66) | 0.27(0.11-0.46) | 0.2(0.08-0.35) | -1.12(-1.25--1) |
| Middle SDI | Male | 1345(595.19-2265.67) | 3040.6(1321.76-5482.79) | 0.3(0.13-0.5) | 0.28(0.12-0.5) | -0.14(-0.3-0.03) |
| **Region** | | | | | | |
| Africa | Both | 170.32(72.08-370.02) | 336.32(161.35-659.31) | 0.07(0.03-0.15) | 0.06(0.03-0.12) | -0.6(-0.69--0.51) |
| Africa | Female | 79.45(34.44-179.19) | 159.8(73.86-314.79) | 0.06(0.03-0.14) | 0.06(0.03-0.11) | -0.5(-0.57--0.43) |
| Africa | Male | 90.87(37.78-188.88) | 176.51(85.68-335.16) | 0.08(0.03-0.16) | 0.07(0.03-0.13) | -0.69(-0.8--0.58) |
| America | Both | 2886.16(1116.34-5294.42) | 3435.68(1321.33-6761.19) | 0.49(0.19-0.89) | 0.27(0.1-0.52) | -2.1(-2.25--1.95) |
| America | Female | 1546.65(609.87-2844.67) | 1780.81(696.65-3490.85) | 0.44(0.18-0.82) | 0.24(0.09-0.48) | -2.11(-2.22--2.01) |
| America | Male | 1339.51(516.34-2475.71) | 1654.86(626.45-3251.14) | 0.53(0.2-0.98) | 0.29(0.11-0.58) | -2.08(-2.29--1.86) |
| Asia | Both | 5430.6(2295.63-9532.69) | 11364.46(4786.88-21062.42) | 0.3(0.13-0.52) | 0.26(0.11-0.47) | -0.47(-0.63--0.31) |
| Asia | Female | 2651.39(1096.28-4725.88) | 5240.74(2200.2-9620.38) | 0.28(0.12-0.5) | 0.22(0.09-0.41) | -0.83(-0.97--0.7) |
| Asia | Male | 2779.22 (1196.34-4808.08) | 6123.72(2575.67-11166.11) | 0.32(0.14-0.56) | 0.3(0.13-0.55) | -0.15(-0.35-0.04) |
| Europe | Both | 4041.38(1500.63-8495.27) | 5328.62(2009.44-11086.52) | 0.4(0.15-0.83) | 0.32(0.12-0.67) | -1.05(-1.29--0.8) |
| Europe | Female | 2135.32(797.02-4487.18) | 2544.97(933.87-5367.51) | 0.33(0.12-0.7) | 0.25(0.09-0.53) | -1.33(-1.61--1.06) |
| Europe | Male | 1906.06(712.3-4005.65) | 2783.64(1053.21-5795.74) | 0.51(0.19-1.05) | 0.42(0.16-0.88) | -0.91(-1.12--0.7) |
| Andean Latin America | Both | 47.37(16.93-92.16) | 128.15(49.17-242.45) | 0.25(0.09-0.48) | 0.24(0.09-0.45) | -0.01(-0.13-0.11) |
| Andean Latin America | Female | 25.8(9.32-50.79) | 69.54(26.77-136.91) | 0.26(0.09-0.52) | 0.24(0.09-0.48) | -0.23(-0.35--0.11) |
| Andean Latin America | Male | 21.56(8.09-41.3) | 58.61(21.94-110.29) | 0.23(0.09-0.44) | 0.23(0.08-0.43) | 0.26(0.11-0.41) |
| Australasia | Both | 156.64(55.42-306.63) | 186.87(69.06-365.12) | 0.68(0.24-1.32) | 0.36(0.13-0.71) | -2.59(-2.77--2.4) |
| Australasia | Female | 75.83(26.49-150.22) | 94.82(34.61-182.61) | 0.58(0.2-1.14) | 0.33(0.12-0.64) | -2.26(-2.41--2.11) |
| Australasia | Male | 80.8(29.24-160.09) | 92.05(33.86-186.13) | 0.81(0.29-1.59) | 0.39(0.15-0.79) | -3(-3.23--2.77) |
| Caribbean | Both | 85.65(30.56-169.62) | 109.74(43.09-222.52) | 0.34(0.12-0.68) | 0.21(0.08-0.43) | -1.81(-2.1--1.52) |
| Caribbean | Female | 45.17(15.86-91.45) | 54.77(22.01-111.02) | 0.35(0.12-0.7) | 0.19(0.08-0.39) | -2.21(-2.51--1.9) |
| Caribbean | Male | 40.47(14.92-78.22) | 54.97(21.39-111.29) | 0.34(0.12-0.65) | 0.23(0.09-0.47) | -1.4(-1.68--1.13) |
| Central Asia | Both | 118.66(41.9-243.78) | 115.43(40.58-255.96) | 0.26(0.09-0.53) | 0.18(0.06-0.4) | -1.81(-2.24--1.37) |
| Central Asia | Female | 60.94(21.16-127.2) | 56.49(19.82-128.12) | 0.22(0.08-0.47) | 0.15(0.05-0.35) | -1.89(-2.37--1.42) |
| Central Asia | Male | 57.72(20.75-114.82) | 58.94(20.76-127.65) | 0.31(0.11-0.61) | 0.22(0.08-0.47) | -1.75(-2.14--1.37) |
| Central Europe | Both | 494.83(177.6-1101.93) | 763.95(280.45-1622.35) | 0.35(0.13-0.78) | 0.35(0.13-0.74) | -0.1(-0.4-0.21) |
| Central Europe | Female | 233.64(84.07-522.32) | 331.33(121.17-712.08) | 0.28(0.1-0.63) | 0.25(0.09-0.54) | -0.54(-0.84--0.24) |
| Central Europe | Male | 261.19(93.14-569.13) | 432.62(161.13-907.17) | 0.46(0.16-0.98) | 0.49(0.18-1.02) | 0.27(-0.05-0.59) |
| Central Latin America | Both | 95.25(38-179.95) | 319.11(121.89-654.32) | 0.12(0.05-0.23) | 0.14(0.05-0.29) | 0.47(0.38-0.57) |
| Central Latin America | Female | 52.15(20.35-101.02) | 163.06(62.63-335.14) | 0.13(0.05-0.26) | 0.13(0.05-0.27) | 0.06(-0.04-0.16) |
| Central Latin America | Male | 43.1(17.19-81.03) | 156.05(59.92-322.12) | 0.11(0.05-0.22) | 0.15(0.06-0.31) | 0.94(0.8-1.07) |
| Central Sub-Saharan Africa | Both | 16.95(6.25-39.45) | 55.58(18.8-121.15) | 0.09(0.03-0.21) | 0.12(0.04-0.27) | 0.98(0.77-1.19) |
| Central Sub-Saharan Africa | Female | 7.3(2.76-17.99) | 26.72(8.55-58.92) | 0.08(0.03-0.18) | 0.11(0.03-0.24) | 1.16(0.91-1.41) |
| Central Sub-Saharan Africa | Male | 9.65(3.43-21.92) | 28.86(10.09-62.87) | 0.11(0.04-0.24) | 0.15(0.05-0.33) | 0.96(0.75-1.17) |
| East Asia | Both | 2025.24(797.76-3824.56) | 2898.4(1196.87-6261.18) | 0.25(0.1-0.47) | 0.15(0.06-0.33) | -1.32(-1.64--1) |
| East Asia | Female | 991.38(368.42-1926.76) | 1166.73(449.67-2617.28) | 0.24(0.09-0.45) | 0.11(0.04-0.25) | -2.28(-2.55--2.01) |
| East Asia | Male | 1033.86(399.77-1990.3) | 1731.67(693.71-3726.96) | 0.28(0.11-0.54) | 0.21(0.08-0.44) | -0.42(-0.78--0.06) |
| Eastern Europe | Both | 622.75(235.12-1418.66) | 892.11(333.67-1988.12) | 0.23(0.09-0.52) | 0.26(0.1-0.57) | -0.7(-1.34--0.05) |
| Eastern Europe | Female | 360.59(134.18-842.77) | 474.4(172.08-1060.59) | 0.2(0.07-0.47) | 0.21(0.08-0.47) | -0.92(-1.54--0.29) |
| Eastern Europe | Male | 262.16(98.99-587.78) | 417.71(158.05-931.89) | 0.3(0.11-0.67) | 0.34(0.13-0.76) | -0.63(-1.29-0.04) |
| Eastern Sub-Saharan Africa | Both | 41.99(18.02-92.75) | 84.32(40.57-164.19) | 0.06(0.03-0.14) | 0.06(0.03-0.11) | -0.58(-0.65--0.52) |
| Eastern Sub-Saharan Africa | Female | 19.36(8.42-45.38) | 40.64(19.37-77.94) | 0.06(0.02-0.13) | 0.05(0.02-0.1) | -0.49(-0.55--0.43) |
| Eastern Sub-Saharan Africa | Male | 22.62(9.45-47.4) | 43.68(21.22-83.22) | 0.07(0.03-0.15) | 0.06(0.03-0.12) | -0.63(-0.71--0.55) |
| High-income Asia Pacific | Both | 637.7(241.34-1309.6) | 1912.36(770.57-3631.14) | 0.34(0.13-0.69) | 0.38(0.15-0.71) | 0.5(0.34-0.66) |
| High-income Asia Pacific | Female | 328.17(124.78-656.29) | 1019.85(416.48-1934.82) | 0.3(0.11-0.6) | 0.32(0.13-0.6) | 0.33(0.22-0.44) |
| High-income Asia Pacific | Male | 309.53(116.54-641.51) | 892.5(350.8-1712.77) | 0.4(0.15-0.83) | 0.44(0.17-0.85) | 0.49(0.27-0.7) |
| High-income North America | Both | 2160.61(832.54-3969.63) | 1910.15(731.57-3778.24) | 0.6(0.23-1.09) | 0.29(0.11-0.58) | -2.51(-2.68--2.33) |
| High-income North America | Female | 1170.62(475.83-2122.27) | 1017.87(396.48-2001.15) | 0.52(0.21-0.97) | 0.27(0.1-0.52) | -2.32(-2.42--2.21) |
| High-income North America | Male | 989.98(378.21-1833.81) | 892.28(333.06-1827.3) | 0.69(0.27-1.27) | 0.32(0.12-0.65) | -2.75(-3.01--2.48) |
| North Africa and Middle East | Both | 109.7(47.79-242.37) | 309.06(138.93-614.4) | 0.07(0.03-0.16) | 0.08(0.04-0.16) | 0.24(0.16-0.32) |
| North Africa and Middle East | Female | 52.36(22.85-116.66) | 143.04(62.77-285.23) | 0.07(0.03-0.15) | 0.07(0.03-0.15) | 0.21(0.14-0.29) |
| North Africa and Middle East | Male | 57.34(24.33-121.05) | 166.02(75.26-324.38) | 0.07(0.03-0.16) | 0.08(0.04-0.16) | 0.27(0.18-0.35) |
| Oceania | Both | 0.98(0.52-2.08) | 1.72(1.11-3.11) | 0.04(0.02-0.08) | 0.03(0.02-0.05) | -0.89(-1.16--0.61) |
| Oceania | Female | 0.44(0.23-0.95) | 0.76(0.48-1.41) | 0.04(0.02-0.08) | 0.03(0.02-0.05) | -0.89(-1.19--0.6) |
| Oceania | Male | 0.54(0.28-1.1) | 0.96(0.59-1.77) | 0.04(0.02-0.09) | 0.03(0.02-0.06) | -0.87(-1.13--0.61) |
| South Asia | Both | 902.04(378.89-1592.9) | 2157.32(871.62-4123.61) | 0.19(0.08-0.33) | 0.17(0.07-0.33) | -0.49(-0.71--0.26) |
| South Asia | Female | 417.17(162.95-793.18) | 1077.68(424.86-2051.26) | 0.18(0.07-0.34) | 0.17(0.07-0.31) | -0.48(-0.71--0.25) |
| South Asia | Male | 484.88(205.87-842.78) | 1079.64(452.28-2061.55) | 0.2(0.08-0.33) | 0.18(0.08-0.34) | -0.47(-0.7--0.23) |
| Southeast Asia | Both | 1556.83(790.79-2285.74) | 3919.59(1783.25-6397.99) | 0.66(0.34-0.96) | 0.7(0.32-1.14) | 0.09(0.02-0.16) |
| Southeast Asia | Female | 762.98(387.51-1127.4) | 1744.31(781.54-2836.77) | 0.6(0.3-0.89) | 0.57(0.26-0.94) | -0.29(-0.37--0.21) |
| Southeast Asia | Male | 793.85(403.53-1159.99) | 2175.28(971.87-3579.55) | 0.72(0.37-1.05) | 0.85(0.38-1.4) | 0.46(0.39-0.52) |
| Southern Latin America | Both | 288.42(101.49-536.97) | 493.78(174.04-954.88) | 0.66(0.24-1.22) | 0.58(0.21-1.13) | -0.17(-0.31--0.03) |
| Southern Latin America | Female | 141.47(50.13-266.29) | 238.13(86.79-474.91) | 0.57(0.2-1.06) | 0.48(0.17-0.96) | -0.37(-0.49--0.25) |
| Southern Latin America | Male | 146.95(52.4-273) | 255.65(88.9-491.23) | 0.78(0.29-1.45) | 0.72(0.25-1.39) | 0.04(-0.12-0.2) |
| Southern Sub-Saharan Africa | Both | 20.9(8.59-46.85) | 53.19(21.67-117.89) | 0.09(0.03-0.19) | 0.11(0.04-0.23) | 0.63(0.41-0.85) |
| Southern Sub-Saharan Africa | Female | 10.9(4.4-25.33) | 25.99(9.9-58.27) | 0.08(0.03-0.18) | 0.09(0.03-0.2) | 0.44(0.3-0.58) |
| Southern Sub-Saharan Africa | Male | 10(4.03-21.77) | 27.2(11.34-60.03) | 0.1(0.04-0.21) | 0.13(0.05-0.29) | 0.94(0.62-1.26) |
| Tropical Latin America | Both | 223.45(87.32-421.7) | 499.91(198.41-1019.53) | 0.27(0.11-0.51) | 0.21(0.08-0.43) | -1.22(-1.36--1.08) |
| Tropical Latin America | Female | 118.48(46.34-225.51) | 249.37(99.27-524.18) | 0.27(0.11-0.52) | 0.19(0.08-0.4) | -1.62(-1.78--1.46) |
| Tropical Latin America | Male | 104.97(41.23-196.59) | 250.53(98.91-506.42) | 0.27(0.11-0.51) | 0.24(0.09-0.48) | -0.76(-0.88--0.64) |
| Western Europe | Both | 2892.37(1074.48-5896.3) | 3624.39(1342.54-7417.69) | 0.49(0.18-0.99) | 0.35(0.13-0.73) | -1.28(-1.4--1.16) |
| Western Europe | Female | 1525.6(565.14-3135.95) | 1716.42(634.7-3615.32) | 0.41(0.15-0.85) | 0.28(0.1-0.59) | -1.55(-1.7--1.4) |
| Western Europe | Male | 1366.77(495.2-2774.81) | 1907.97(707.84-3914.95) | 0.61(0.22-1.22) | 0.45(0.17-0.92) | -1.18(-1.26--1.09) |
| Western Sub-Saharan Africa | Both | 49.2(21.77-105.66) | 63.68(37.83-114.27) | 0.07(0.03-0.14) | 0.04(0.02-0.07) | -1.75(-2.05--1.46) |
| Western Sub-Saharan Africa | Female | 21.75(9.74-47.86) | 30.23(18.05-54.11) | 0.06(0.03-0.13) | 0.04(0.02-0.07) | -1.5(-1.78--1.22) |
| Western Sub-Saharan Africa | Male | 27.45(12.05-55.84) | 33.45(19.56-59.78) | 0.07(0.03-0.15) | 0.04(0.03-0.08) | -1.97(-2.28--1.65) |

ASDR, age-standardized death rate, SDI, socio-demographic index; UI, uncertainty interval.
