# Supplementary material for: Dietary Risk-Related Colorectal Cancer Burden: Estimates From 1990 to 2019
Source: Front Nutr. 2021 Aug 24;8:690663. doi: 10.3389/fnut.2021.690663 (PMC8421520; doi:10.3389/fnut.2021.690663)
Supplement: Supplementary file 3 [file Data_Sheet_3.zip › Supplemental tables/Table S12.docx]

**Table S12** DALYs, ASRs and change trends of colorectal cancer DALYs attributable to diet low in fiber between 1990 and 2019 by SDI, regions and sex.

| **Location** | **Sex** | **DALYs (No.×1000, 95%UI)** | | **ASR (95%UI)** | | **EAPC (95%CI)** |
| --- | --- | --- | --- | --- | --- | --- |
|  |  | **1990** | **2019** | **1990** | **2019** | **1990-2019** |
| Global | Both | 296.44(117.32-554.17) | 448.7(178.29-858.39) | 7.44(2.95-13.87) | 5.49(2.18-10.52) | -1.11(-1.23--0.99) |
| Global | Female | 143.01(56.61-274.69) | 197.33(80.16-381.27) | 6.72(2.65-12.88) | 4.56(1.85-8.79) | -1.48(-1.6--1.36) |
| Global | Male | 153.43(61.71-284.23) | 251.36(99.64-475.59) | 8.31(3.33-15.35) | 6.53(2.59-12.42) | -0.82(-0.94--0.7) |
| **Sociodemographic Index** | | | | | | |
| High SDI | Both | 111.31(41.75-217.35) | 128.03(48.31-256.96) | 10.82(4.06-21.15) | 7.13(2.67-14.27) | -1.45(-1.48--1.42) |
| High SDI | Female | 53.65(19.85-105.79) | 58.17(21.97-117.09) | 9.06(3.36-17.92) | 5.89(2.2-11.96) | -1.51(-1.55--1.48) |
| High SDI | Male | 57.65(21.67-112.63) | 69.86(25.95-140.26) | 13.13(4.96-25.59) | 8.52(3.17-17.11) | -1.49(-1.53--1.45) |
| High-middle SDI | Both | 66.42(24.82-138.66) | 96.05(37.38-200.91) | 6.18(2.31-12.86) | 4.82(1.88-10.08) | -1.08(-1.33--0.83) |
| High-middle SDI | Female | 32.28(12.25-69) | 40.56(15.57-88.46) | 5.44(2.06-11.55) | 3.74(1.44-8.09) | -1.65(-1.92--1.38) |
| High-middle SDI | Male | 34.14(13.37-69.46) | 55.48(21.9-113.56) | 7.2(2.85-14.67) | 6.1(2.41-12.47) | -0.67(-0.92--0.43) |
| Low SDI | Both | 6.53(2.57-13.22) | 14.98(5.87-28.76) | 2.57(1.01-5.2) | 2.65(1.03-5.12) | 0.04(-0.15-0.22) |
| Low SDI | Female | 3.06(1.17-6.51) | 7.35(2.78-14.31) | 2.38(0.94-5.08) | 2.52(0.96-4.87) | 0.13(-0.06-0.32) |
| Low SDI | Male | 3.47(1.34-6.78) | 7.63(3.18-14.92) | 2.76(1.09-5.38) | 2.78(1.16-5.48) | -0.02(-0.2-0.15) |
| Low-middle SDI | Both | 35.56(16.19-59.07) | 70.38(30.31-129.74) | 5.44(2.48-9.05) | 4.94(2.15-9.06) | -0.45(-0.59--0.3) |
| Low-middle SDI | Female | 17.34(7.87-29.66) | 34.03(14.01-61.59) | 5.32(2.45-9.12) | 4.62(1.91-8.35) | -0.68(-0.82--0.53) |
| Low-middle SDI | Male | 18.22(8.16-30.57) | 36.35(15.48-66.4) | 5.55(2.51-9.29) | 5.3(2.26-9.75) | -0.22(-0.38--0.06) |
| Middle SDI | Both | 76.45(32.5-129.01) | 138.99(59.17-252.74) | 6.75(2.89-11.39) | 5.42(2.32-9.87) | -0.7(-0.84--0.56) |
| Middle SDI | Female | 36.6(15.36-62.42) | 57.11(24.12-104.31) | 6.42(2.69-10.93) | 4.34(1.85-7.88) | -1.38(-1.49--1.27) |
| Middle SDI | Male | 39.85(17.4-66.93) | 81.88(35.49-147.03) | 7.09(3.12-11.95) | 6.56(2.85-11.79) | -0.14(-0.32-0.03) |
| **Region** | | | | | | |
| Africa | Both | 4.55(1.91-9.97) | 8.89(4.24-16.97) | 1.5(0.63-3.27) | 1.3(0.63-2.52) | -0.64(-0.72--0.56) |
| Africa | Female | 2.1(0.91-4.77) | 4.11(1.88-8.04) | 1.36(0.59-3.06) | 1.17(0.54-2.29) | -0.6(-0.67--0.53) |
| Africa | Male | 2.45(1.02-5.09) | 4.78(2.3-9.1) | 1.64(0.68-3.4) | 1.44(0.7-2.74) | -0.66(-0.76--0.57) |
| America | Both | 58.96(22.65-110.77) | 69.74(26.58-138.12) | 9.66(3.72-18.14) | 5.59(2.12-11.05) | -1.86(-2.01--1.71) |
| America | Female | 29.1(11.01-54.86) | 33.54(12.79-66.98) | 8.56(3.23-16.21) | 4.95(1.88-9.89) | -1.85(-1.95--1.74) |
| America | Male | 29.86(11.41-56.1) | 36.2(13.7-71.72) | 10.93(4.19-20.51) | 6.3(2.39-12.44) | -1.88(-2.08--1.68) |
| Asia | Both | 152.42(64.16-266.42) | 274.96(114.84-508.58) | 6.99(2.94-12.21) | 5.67(2.38-10.53) | -0.66(-0.82--0.49) |
| Asia | Female | 72.34(30.53-126.59) | 117.74(48.93-218.23) | 6.59(2.76-11.63) | 4.73(1.97-8.74) | -1.19(-1.32--1.06) |
| Asia | Male | 80.08(34.18-138.82) | 157.21(65.54-284.28) | 7.43(3.19-12.86) | 6.66(2.79-12.1) | -0.23(-0.43--0.02) |
| Europe | Both | 80.1(29.25-173.73) | 94.43(34.77-201.39) | 7.86(2.89-17) | 6.3(2.32-13.43) | -1.15(-1.4--0.9) |
| Europe | Female | 39.28(14.36-85.58) | 41.64(14.87-89.64) | 6.49(2.37-14.21) | 4.87(1.73-10.55) | -1.43(-1.7--1.16) |
| Europe | Male | 40.82(87.06-15.02) | 52.79(111.77-19.88) | 9.96(21.05-3.72) | 8.16(17.29-3.07) | -1.02(-1.25--0.78) |
| Andean Latin America | Both | 1.14(0.4-2.21) | 2.8(1.07-5.27) | 5.27(1.86-10.24) | 4.91(1.88-9.25) | -0.06(-0.19-0.07) |
| Andean Latin America | Female | 0.6(0.21-1.18) | 1.45(0.55-2.81) | 5.41(1.9-10.82) | 4.9(1.86-9.57) | -0.25(-0.38--0.12) |
| Andean Latin America | Male | 0.54(0.2-1.04) | 1.35(0.52-2.5) | 5.11(1.88-9.85) | 4.9(1.86-9.11) | 0.15(0.01-0.3) |
| Australasia | Both | 3.47(1.21-6.89) | 3.7(1.33-7.31) | 15(5.23-29.78) | 7.91(2.87-15.75) | -2.65(-2.85--2.45) |
| Australasia | Female | 1.59(0.56-3.15) | 1.72(0.61-3.32) | 12.75(4.48-25.42) | 6.89(2.5-13.41) | -2.49(-2.67--2.32) |
| Australasia | Male | 1.88(0.67-3.75) | 1.98(0.72-4.07) | 17.7(6.34-35.27) | 8.97(3.28-18.29) | -2.86(-3.08--2.63) |
| Caribbean | Both | 1.93(0.69-3.85) | 2.34(0.9-4.82) | 7.25(2.61-14.52) | 4.56(1.76-9.39) | -1.71(-2.03--1.39) |
| Caribbean | Female | 0.99(0.35-2.03) | 1.09(0.43-2.29) | 7.18(2.51-14.75) | 4(1.57-8.42) | -2.17(-2.51--1.84) |
| Caribbean | Male | 0.94(0.34-1.82) | 1.25(0.49-2.58) | 7.31(2.64-14.25) | 5.17(2-10.64) | -1.27(-1.57--0.97) |
| Central Asia | Both | 3.44(1.21-6.98) | 3.05(1.07-6.65) | 6.84(2.41-13.96) | 3.98(1.39-8.73) | -2.67(-3.13--2.21) |
| Central Asia | Female | 1.64(0.57-3.41) | 1.39(0.49-3.15) | 5.83(2.03-12.16) | 3.3(1.16-7.51) | -2.81(-3.31--2.3) |
| Central Asia | Male | 1.8(0.65-3.54) | 1.67(0.59-3.53) | 8.18(2.95-16.24) | 4.86(1.72-10.44) | -2.56(-2.96--2.15) |
| Central Europe | Both | 10.95(3.92-24.56) | 14.81(5.38-31.82) | 7.56(2.7-16.88) | 7.21(2.6-15.42) | -0.22(-0.52-0.08) |
| Central Europe | Female | 4.86(1.74-11.16) | 5.94(2.16-12.79) | 5.97(2.13-13.59) | 5.12(1.86-10.98) | -0.64(-0.93--0.36) |
| Central Europe | Male | 6.08(2.19-13.41) | 8.87(3.27-18.8) | 9.67(3.45-21.22) | 9.91(3.64-21.02) | 0.08(-0.24-0.4) |
| Central Latin America | Both | 2.37(0.93-4.5) | 7.29(2.76-15) | 2.64(1.04-5.03) | 3.04(1.15-6.27) | 0.62(0.51-0.74) |
| Central Latin America | Female | 1.25(0.48-2.43) | 3.55(1.33-7.42) | 2.73(1.06-5.28) | 2.77(1.04-5.77) | 0.23(0.11-0.36) |
| Central Latin America | Male | 1.12(0.44-2.11) | 3.74(1.43-7.73) | 2.54(1.01-4.82) | 3.35(1.28-6.9) | 1.04(0.9-1.18) |
| Central Sub-Saharan Africa | Both | 0.49(0.18-1.15) | 1.56(0.54-3.39) | 2.02(0.74-4.62) | 2.65(0.9-5.76) | 0.91(0.69-1.12) |
| Central Sub-Saharan Africa | Female | 0.21(0.08-0.54) | 0.72(0.23-1.58) | 1.66(0.62-4.09) | 2.27(0.72-5.1) | 1.15(0.87-1.43) |
| Central Sub-Saharan Africa | Male | 0.28(0.1-0.63) | 0.84(0.29-1.83) | 2.42(0.85-5.57) | 3.13(1.1-6.83) | 0.77(0.58-0.96) |
| East Asia | Both | 61.76(24.29-114.94) | 76.62(31.59-162.4) | 6.36(2.51-11.83) | 3.76(1.53-7.9) | -1.42(-1.73--1.11) |
| East Asia | Female | 29.11(10.91-55.12) | 28.34(11.22-62.23) | 6.01(2.25-11.41) | 2.71(1.08-5.81) | -2.62(-2.85--2.39) |
| East Asia | Male | 32.64(12.84-62.31) | 48.29(18.74-103.45) | 6.78(2.66-13.11) | 4.92(1.92-10.55) | -0.5(-0.89--0.11) |
| Eastern Europe | Both | 14.85(5.53-34.13) | 19.12(7.17-42.86) | 5.39(2.01-12.37) | 5.78(2.16-12.91) | -0.93(-1.61--0.26) |
| Eastern Europe | Female | 8.06(2.99-19.16) | 9.42(3.43-21.48) | 4.7(1.76-11.24) | 4.67(1.69-10.6) | -1.2(-1.87--0.54) |
| Eastern Europe | Male | 6.79(2.57-15.25) | 9.7(3.73-22.28) | 6.74(2.53-15.06) | 7.55(2.95-17.12) | -0.77(-1.45--0.08) |
| Eastern Sub-Saharan Africa | Both | 1.21(0.51-2.71) | 2.41(1.15-4.63) | 1.45(0.62-3.21) | 1.28(0.62-2.49) | -0.64(-0.7--0.57) |
| Eastern Sub-Saharan Africa | Female | 0.57(0.24-1.36) | 1.15(0.54-2.22) | 1.32(0.57-3.1) | 1.17(0.56-2.22) | -0.63(-0.7--0.56) |
| Eastern Sub-Saharan Africa | Male | 0.64(0.26-1.34) | 1.26(0.61-2.42) | 1.58(0.66-3.3) | 1.41(0.69-2.68) | -0.62(-0.69--0.55) |
| High-income Asia Pacific | Both | 14.35(5.37-29.22) | 32.05(12.43-61.02) | 7.2(2.71-14.59) | 7.99(3.07-15.11) | 0.55(0.34-0.76) |
| High-income Asia Pacific | Female | 6.86(2.59-13.8) | 14.8(5.86-27.97) | 6.21(2.34-12.49) | 6.48(2.52-12.07) | 0.3(0.16-0.43) |
| High-income Asia Pacific | Male | 7.49(2.83-15.54) | 17.26(6.74-33.52) | 8.49(3.19-17.6) | 9.63(3.76-18.55) | 0.64(0.38-0.9) |
| High-income North America | Both | 41.92(16.23-79.12) | 36.1(13.67-73.31) | 12.12(4.68-22.89) | 6.13(2.32-12.42) | -2.31(-2.48--2.14) |
| High-income North America | Female | 20.61(7.86-38.76) | 17.57(6.73-35.13) | 10.29(3.87-19.44) | 5.46(2.06-11) | -2.1(-2.19--2) |
| High-income North America | Male | 21.31(8.13-40.21) | 18.53(6.98-39.21) | 14.38(5.49-27.19) | 6.86(2.55-14.51) | -2.56(-2.82--2.3) |
| North Africa and Middle East | Both | 3.02(1.32-6.72) | 8.2(3.6-16.12) | 1.62(0.71-3.58) | 1.72(0.77-3.38) | 0.12(0.04-0.2) |
| North Africa and Middle East | Female | 1.45(0.62-3.17) | 3.81(1.66-7.68) | 1.56(0.67-3.43) | 1.63(0.71-3.26) | 0.06(0-0.12) |
| North Africa and Middle East | Male | 1.57(0.66-3.32) | 4.39(1.99-8.6) | 1.68(0.71-3.54) | 1.81(0.82-3.6) | 0.18(0.08-0.28) |
| Oceania | Both | 0.03(0.02-0.06) | 0.05(0.03-0.09) | 0.88(0.46-1.88) | 0.63(0.41-1.15) | -1.06(-1.32--0.81) |
| Oceania | Female | 0.01(0.01-0.03) | 0.02(0.01-0.04) | 0.8(0.42-1.73) | 0.56(0.36-1.05) | -1.09(-1.37--0.82) |
| Oceania | Male | 0.02(0.01-0.03) | 0.03(0.02-0.05) | 0.96(0.48-1.99) | 0.7(0.44-1.31) | -1.04(-1.28--0.8) |
| South Asia | Both | 25.24(10.56-44.68) | 53.78(21.75-105.21) | 4.17(1.75-7.34) | 3.71(1.51-7.19) | -0.55(-0.76--0.33) |
| South Asia | Female | 12.02(4.77-22.37) | 26.96(10.42-52.89) | 4.08(1.61-7.69) | 3.66(1.42-7.11) | -0.56(-0.77--0.36) |
| South Asia | Male | 13.22(5.56-23.13) | 26.82(11.4-51.35) | 4.25(1.81-7.41) | 3.78(1.6-7.19) | -0.51(-0.75--0.28) |
| Southeast Asia | Both | 43.32(21.81-63.85) | 100.93(44.74-165.14) | 15.46(7.81-22.77) | 15.81(7.11-25.73) | -0.06(-0.14-0.02) |
| Southeast Asia | Female | 20.72(10.57-31.07) | 42.24(18.52-69.09) | 14.07(7.16-20.88) | 12.59(5.53-20.53) | -0.51(-0.6--0.42) |
| Southeast Asia | Male | 22.6(11.44-33.03) | 58.69(26.45-97.21) | 17.02(8.65-24.87) | 19.48(8.72-32.08) | 0.33(0.25-0.4) |
| Southern Latin America | Both | 6.13(2.15-11.58) | 9.75(3.4-19.1) | 13.37(4.71-25.31) | 11.89(4.16-23.26) | -0.08(-0.24-0.08) |
| Southern Latin America | Female | 2.83(1-5.38) | 4.36(1.55-8.8) | 11.18(3.96-21.37) | 9.53(3.34-19.35) | -0.24(-0.39--0.09) |
| Southern Latin America | Male | 3.3(1.16-6.2) | 5.39(1.88-10.58) | 16.07(5.66-30) | 14.8(5.2-28.87) | 0.05(-0.12-0.23) |
| Southern Sub-Saharan Africa | Both | 0.52(0.21-1.18) | 1.32(0.55-2.89) | 1.76(0.73-3.96) | 2.23(0.91-4.89) | 0.83(0.58-1.08) |
| Southern Sub-Saharan Africa | Female | 0.25(0.1-0.58) | 0.59(0.23-1.29) | 1.51(0.63-3.56) | 1.76(0.68-3.89) | 0.75(0.59-0.91) |
| Southern Sub-Saharan Africa | Male | 0.27(0.11-0.61) | 0.74(0.3-1.62) | 2.05(0.84-4.48) | 2.85(1.18-6.24) | 0.99(0.62-1.35) |
| Tropical Latin America | Both | 5.79(2.26-10.87) | 11.94(4.69-24.22) | 5.94(2.33-11.19) | 4.85(1.9-9.82) | -1.04(-1.18--0.91) |
| Tropical Latin America | Female | 2.97(1.16-5.71) | 5.73(2.24-11.83) | 5.81(2.25-11.08) | 4.31(1.69-8.9) | -1.39(-1.53--1.25) |
| Tropical Latin America | Male | 2.81(1.13-5.17) | 6.21(2.5-12.3) | 6.08(2.42-11.31) | 5.51(2.19-10.97) | -0.67(-0.79--0.55) |
| Western Europe | Both | 53.33(19.27-110.8) | 59.3(21.5-123.95) | 9.42(3.39-19.62) | 6.78(2.42-14.35) | -1.31(-1.4--1.22) |
| Western Europe | Female | 25.92(9.28-54.99) | 25.77(9.12-55.18) | 7.8(2.79-16.61) | 5.28(1.86-11.36) | -1.55(-1.65--1.45) |
| Western Europe | Male | 27.41(10.05-56.84) | 33.53(12.26-69.97) | 11.67(4.3-24.06) | 8.54(3.12-17.9) | -1.23(-1.31--1.15) |
| Western Sub-Saharan Africa | Both | 1.21(0.54-2.61) | 1.56(0.94-2.75) | 1.35(0.6-2.9) | 0.8(0.48-1.42) | -1.92(-2.25--1.59) |
| Western Sub-Saharan Africa | Female | 0.51(0.23-1.13) | 0.73(0.43-1.29) | 1.16(0.52-2.57) | 0.72(0.43-1.27) | -1.73(-2.05--1.42) |
| Western Sub-Saharan Africa | Male | 0.69(0.3-1.44) | 0.84(0.49-1.48) | 1.53(0.67-3.14) | 0.89(0.53-1.56) | -2.04(-2.39--1.68) |

ASDR, age-standardized death rate; DALYs, disability-adjusted life years; SDI, socio-demographic index; UI, uncertainty interval.
