# Supplementary material for: Dietary Risk-Related Colorectal Cancer Burden: Estimates From 1990 to 2019
Source: Front Nutr. 2021 Aug 24;8:690663. doi: 10.3389/fnut.2021.690663 (PMC8421520; doi:10.3389/fnut.2021.690663)
Supplement: Supplementary file 3 [file Data_Sheet_3.zip › Supplemental tables/Table S13.docx]

**Table S13** Deaths, ASDRs and change trends of colorectal cancer attributable to diet high in red meat between 1990 and 2019 by SDI, regions and sex

| **Location** | **Sex** | **Deaths (95%UI)** | | **ASDR (95%UI)** | | **EAPC (95%CI)** |
| --- | --- | --- | --- | --- | --- | --- |
|  |  | **1990** | **2019** | **1990** | **2019** | **1990-2019** |
| Global | Both | 26087.14(6690.26-50231.48) | 52811.03(13598.48-100688.32) | 0.71(0.18-1.38) | 0.66(0.17-1.26) | -0.32(-0.37--0.28) |
| Global | Female | 12951.4(3302.45-24856.09) | 22759.72(5663-44003.85) | 0.64(0.16-1.23) | 0.52(0.13-1.01) | -0.83(-0.88--0.78) |
| Global | Male | 13135.74(3427.05-25156.42) | 30051.31(7627.47-57327.51) | 0.81(0.21-1.56) | 0.83(0.21-1.59) | 0.07(0.01-0.12) |
| **Sociodemographic Index** | | | | | | |
| High SDI | Both | 14062.93(3943.84-25419.46) | 18849.06(4893.27-35349.35) | 1.34(0.38-2.42) | 0.97(0.26-1.79) | -1.29(-1.38--1.2) |
| High SDI | Female | 6916.82(1919.59-12838.06) | 8485.65(2154.77-16301.7) | 1.12(0.31-2.05) | 0.77(0.2-1.44) | -1.48(-1.58--1.38) |
| High SDI | Male | 7146.12(2031.16-12816.21) | 10363.41(2748.08-18956.24) | 1.66(0.47-3.01) | 1.22(0.33-2.21) | -1.26(-1.33--1.18) |
| High-middle SDI | Both | 8864.25(2276.2-17224.56) | 18793.09(5032.76-35131.68) | 0.87(0.22-1.7) | 0.93(0.25-1.74) | 0.09(0.01-0.17) |
| High-middle SDI | Female | 4510.44(1119.7-8739.06) | 8078.26(2097.63-15404.3) | 0.76(0.19-1.48) | 0.71(0.19-1.35) | -0.5(-0.59--0.42) |
| High-middle SDI | Male | 4353.81(1143.23-8453.27) | 10714.83(2810.52-20218.72) | 1.02(0.26-2) | 1.22(0.32-2.32) | 0.54(0.46-0.62) |
| Low SDI | Both | 221.38(31.67-565.48) | 597.84(97.74-1464.28) | 0.1(0.01-0.26) | 0.12(0.02-0.3) | 0.81(0.71-0.9) |
| Low SDI | Female | 98.47(13.71-258.02) | 281.28(46.25-688.55) | 0.09(0.01-0.23) | 0.11(0.02-0.27) | 0.89(0.79-0.98) |
| Low SDI | Male | 122.91(17.25-323.3) | 316.56(50.4-782.73) | 0.11(0.02-0.29) | 0.13(0.02-0.33) | 0.78(0.68-0.87) |
| Low-middle SDI | Both | 648.75(121.95-1496.93) | 2826.45(617.01-5903.56) | 0.12(0.02-0.27) | 0.22(0.05-0.45) | 2.38(2.27-2.48) |
| Low-middle SDI | Female | 313.7(57.3-733.37) | 1266.58(292.11-2700.23) | 0.11(0.02-0.27) | 0.19(0.04-0.4) | 1.87(1.79-1.95) |
| Low-middle SDI | Male | 335.05(61.5-801.36) | 1559.88(349.15-3273.81) | 0.12(0.02-0.28) | 0.25(0.05-0.53) | 2.87(2.73-3) |
| Middle SDI | Both | 2278.09(297.13-5661.88) | 11723.43(2560.29-23586.8) | 0.23(0.03-0.59) | 0.49(0.1-0.99) | 2.95(2.76-3.13) |
| Middle SDI | Female | 1106.05(139.95-2751.29) | 4637.92(1019.53-9603.39) | 0.22(0.03-0.55) | 0.37(0.08-0.77) | 2.05(1.92-2.18) |
| Middle SDI | Male | 1172.04(151.24-2931.18) | 7085.51(1493.44-14452.72) | 0.25(0.03-0.63) | 0.62(0.13-1.27) | 3.67(3.45-3.9) |
| **Region** | | | | | | |
| Africa | Both | 380.69(48.11-979.9) | 1068.35(130.96-2734.14) | 0.15(0.02-0.37) | 0.18(0.02-0.47) | 0.97(0.9-1.03) |
| Africa | Female | 181.23(21.72-463.96) | 508.04(62.07-1318.61) | 0.14(0.02-0.35) | 0.17(0.02-0.44) | 0.93(0.86-1.01) |
| Africa | Male | 199.46(25.39-520.33) | 560.31(67.28-1429.24) | 0.15(0.02-0.4) | 0.2(0.02-0.5) | 1.03(0.97-1.09) |
| America | Both | 6371.71(1784.33-11568.36) | 11686.15(3496.92-20712.03) | 1.05(0.29-1.91) | 0.91(0.27-1.61) | -0.52(-0.58--0.46) |
| America | Female | 3091.87(825.89-5733.45) | 5433.13(1553.04-9862.12) | 0.9(0.24-1.67) | 0.77(0.23-1.38) | -0.57(-0.63--0.5) |
| America | Male | 3279.84(958.46-5853.79) | 6253.02(1927.58-10883.73) | 1.24(0.36-2.24) | 1.09(0.33-1.89) | -0.54(-0.6--0.47) |
| Asia | Both | 5430.54(999.15-12448.97) | 23080.67(5419.02-45484.27) | 0.3(0.05-0.68) | 0.5(0.12-0.99) | 2.08(1.94-2.23) |
| Asia | Female | 2544.07(452.37-5833.35) | 8958.35(2087.75-18049.26) | 0.27(0.05-0.62) | 0.37(0.09-0.75) | 1.22(1.12-1.32) |
| Asia | Male | 2886.48(530.28-6686.04) | 14122.32(3183.95-27971.98) | 0.33(0.06-0.76) | 0.65(0.14-1.3) | 2.74(2.56-2.93) |
| Europe | Both | 13878.23(3854.61-25651.47) | 16920.86(4132.08-33040.8) | 1.35(0.38-2.49) | 1.06(0.26-2.05) | -1.14(-1.24--1.04) |
| Europe | Female | 7121.77(1956.42-13348.78) | 7834.76(1898.56-15460.64) | 1.14(0.32-2.11) | 0.83(0.21-1.6) | -1.45(-1.57--1.34) |
| Europe | Male | 6756.45(1901.97-12312.97) | 9086.09(2220.3-17235.3) | 1.69(0.47-3.09) | 1.38(0.34-2.61) | -0.97(-0.88--1.06) |
| Andean Latin America | Both | 35.6(3.83-92.08) | 154.44(17.48-391.64) | 0.18(0.02-0.47) | 0.28(0.03-0.71) | 1.81(1.67-1.96) |
| Andean Latin America | Female | 19.45(2.07-49.72) | 82.39(8.8-210.67) | 0.19(0.02-0.5) | 0.29(0.03-0.73) | 1.54(1.41-1.68) |
| Andean Latin America | Male | 16.14(1.74-42.43) | 72.05(8.73-181.69) | 0.17(0.02-0.44) | 0.27(0.03-0.69) | 2.14(1.97-2.3) |
| Australasia | Both | 630.44(275.66-959.22) | 887.65(367.3-1386.68) | 2.72(1.19-4.15) | 1.75(0.74-2.71) | -1.8(-1.97--1.62) |
| Australasia | Female | 301.2(129.12-460.45) | 411.18(164.63-650.93) | 2.32(1-3.54) | 1.47(0.62-2.3) | -1.79(-1.96--1.63) |
| Australasia | Male | 329.24(142.11-499.68) | 476.48(197.93-734.76) | 3.23(1.36-4.94) | 2.06(0.86-3.16) | -1.87(-2.06--1.67) |
| Caribbean | Both | 83.14(7.83-219.66) | 209.2(19.74-560.44) | 0.33(0.03-0.87) | 0.4(0.04-1.08) | 0.9(0.79-1.01) |
| Caribbean | Female | 43.63(4.04-115.7) | 107.97(9.93-290.42) | 0.33(0.03-0.88) | 0.38(0.04-1.04) | 0.69(0.6-0.79) |
| Caribbean | Male | 39.5(3.76-104.54) | 101.23(9.63-271.68) | 0.33(0.03-0.86) | 0.42(0.04-1.13) | 1.11(0.98-1.25) |
| Central Asia | Both | 271.4(69.96-523.58) | 389.27(98.31-756.01) | 0.57(0.14-1.11) | 0.56(0.14-1.11) | 0.39(-0.15-0.94) |
| Central Asia | Female | 139.78(35.04-270.58) | 191.3(47.94-373) | 0.51(0.13-0.98) | 0.49(0.12-0.95) | 0.31(-0.2-0.82) |
| Central Asia | Male | 131.63(33.97-250.48) | 197.97(51.17-378.16) | 0.67(0.17-1.3) | 0.66(0.16-1.3) | 0.43(-0.16-1.02) |
| Central Europe | Both | 1538.23(321.9-3190.13) | 2862.48(698.95-5678.47) | 1.06(0.22-2.23) | 1.33(0.33-2.61) | 1.04(0.91-1.18) |
| Central Europe | Female | 723.73(148.86-1519.88) | 1204.82(291.74-2388.88) | 0.87(0.18-1.83) | 0.97(0.25-1.88) | 0.53(0.41-0.65) |
| Central Europe | Male | 814.5(175.27-1681.53) | 1657.65(390.3-3310.78) | 1.33(0.28-2.75) | 1.83(0.43-3.64) | 1.45(1.31-1.59) |
| Central Latin America | Both | 188.84(24.4-460.74) | 806.87(129.64-1945.81) | 0.24(0.03-0.58) | 0.34(0.05-0.84) | 1.38(1.32-1.43) |
| Central Latin America | Female | 100.72(12.53-246.41) | 392.47(57.97-950.27) | 0.25(0.03-0.61) | 0.31(0.05-0.75) | 0.89(0.84-0.94) |
| Central Latin America | Male | 88.12(11.41-212.31) | 414.4(65.25-962.29) | 0.23(0.03-0.55) | 0.38(0.06-0.9) | 1.89(1.83-1.96) |
| Central Sub-Saharan Africa | Both | 27.39(4.91-67.42) | 60.77(9.56-149.6) | 0.13(0.02-0.32) | 0.12(0.02-0.3) | -0.18(-0.52-0.17) |
| Central Sub-Saharan Africa | Female | 12.05(2.01-29.97) | 28.01(4.29-71.51) | 0.11(0.02-0.28) | 0.1(0.02-0.26) | -0.23(-0.57-0.1) |
| Central Sub-Saharan Africa | Male | 15.34(2.79-39.17) | 32.77(5.07-81.3) | 0.15(0.03-0.39) | 0.15(0.02-0.37) | -0.01(-0.34-0.33) |
| East Asia | Both | 2850.88(407.73-6969.64) | 16497.73(4056.31-31568.86) | 0.34(0.05-0.84) | 0.82(0.2-1.58) | 3.6(3.33-3.87) |
| East Asia | Female | 1323.28(176.88-3251.75) | 5982.49(1518.99-11873.13) | 0.31(0.04-0.76) | 0.57(0.14-1.13) | 2.49(2.29-2.69) |
| East Asia | Male | 1527.6(216.56-3767.78) | 10515.23(2460.41-20689.81) | 0.4(0.05-0.98) | 1.13(0.25-2.23) | 4.38(4.07-4.69) |
| Eastern Europe | Both | 2994.93(772.03-5768.5) | 2707.64(468.29-5946.85) | 1.07(0.28-2.07) | 0.79(0.14-1.72) | -1.91(-2.22--1.6) |
| Eastern Europe | Female | 1702.79(429.99-3293.34) | 1422.61(237.97-3210.24) | 0.94(0.24-1.81) | 0.65(0.11-1.46) | -2.13(-2.42--1.83) |
| Eastern Europe | Male | 1292.14(345.9-2477.92) | 1285.02(230.06-2855.83) | 1.35(0.35-2.62) | 1.02(0.18-2.26) | -1.8(-2.12--1.47) |
| Eastern Sub-Saharan Africa | Both | 88.08(10.31-230.84) | 240.76(27.1-625.84) | 0.12(0.01-0.32) | 0.16(0.02-0.4) | 0.9(0.78-1.02) |
| Eastern Sub-Saharan Africa | Female | 40.29(4.41-108.47) | 115.37(12.62-304.45) | 0.11(0.01-0.29) | 0.14(0.02-0.37) | 0.99(0.85-1.12) |
| Eastern Sub-Saharan Africa | Male | 47.8(5.51-128.32) | 125.39(13.82-324.19) | 0.14(0.02-0.37) | 0.17(0.02-0.44) | 0.87(0.76-0.97) |
| High-income Asia Pacific | Both | 909.7(86.89-2420.55) | 2195.96(274.82-5546.4) | 0.47(0.05-1.25) | 0.47(0.06-1.14) | -0.25(-0.35--0.15) |
| High-income Asia Pacific | Female | 419.97(39.47-1117.15) | 980.89(110.74-2573.58) | 0.38(0.04-1.01) | 0.34(0.04-0.86) | -0.63(-0.71--0.55) |
| High-income Asia Pacific | Male | 489.73(47.37-1299.97) | 1215.07(154.19-2970.62) | 0.6(0.06-1.6) | 0.61(0.08-1.47) | -0.13(-0.26-0) |
| High-income North America | Both | 4797.76(1331.95-8718.79) | 6385.67(1712.39-11567.11) | 1.36(0.38-2.44) | 1.02(0.28-1.82) | -1.21(-1.33--1.08) |
| High-income North America | Female | 2314.64(611.58-4277.34) | 2849.09(725.67-5409.45) | 1.12(0.31-2.04) | 0.82(0.22-1.53) | -1.29(-1.41--1.18) |
| High-income North America | Male | 2483.11(719.76-4426.62) | 3536.59(1011.81-6222.1) | 1.68(0.49-3.01) | 1.26(0.36-2.22) | -1.26(-1.4--1.12) |
| North Africa and Middle East | Both | 301.04(28.85-820.49) | 881.71(86.01-2352.22) | 0.18(0.02-0.5) | 0.22(0.02-0.57) | 0.76(0.5-1.03) |
| North Africa and Middle East | Female | 143.64(13.39-387.21) | 398.71(38.66-1076.01) | 0.18(0.02-0.48) | 0.2(0.02-0.54) | 0.62(0.37-0.88) |
| North Africa and Middle East | Male | 157.41(14.79-441.24) | 483(46.74-1282.11) | 0.19(0.02-0.53) | 0.23(0.02-0.62) | 0.9(0.62-1.18) |
| Oceania | Both | 6.16(0.73-16.1) | 15.32(1.61-40.78) | 0.22(0.03-0.59) | 0.24(0.03-0.64) | 0.11(-0.03-0.25) |
| Oceania | Female | 2.77(0.31-7.34) | 6.86(0.68-18.76) | 0.21(0.02-0.55) | 0.22(0.02-0.6) | 0.08(-0.06-0.23) |
| Oceania | Male | 3.39(0.4-9.19) | 8.46(0.93-22.06) | 0.24(0.03-0.65) | 0.26(0.03-0.68) | 0.15(0.02-0.29) |
| South Asia | Both | 267.48(69.95-582.64) | 947.46(228.12-2161.25) | 0.05(0.01-0.12) | 0.07(0.02-0.16) | 0.84(0.71-0.97) |
| South Asia | Female | 121.41(31.04-269.58) | 460.46(113.3-1054.8) | 0.05(0.01-0.11) | 0.07(0.02-0.16) | 0.84(0.67-1.01) |
| South Asia | Male | 146.07(37.68-327.36) | 486.99(111.25-1126.95) | 0.06(0.01-0.13) | 0.08(0.02-0.17) | 0.87(0.76-0.97) |
| Southeast Asia | Both | 405.56(51.4-1035.95) | 1821.6(220.19-4549.72) | 0.17(0.02-0.42) | 0.31(0.04-0.77) | 2.15(2.08-2.21) |
| Southeast Asia | Female | 194.54(24.31-497.5) | 777.91(93.89-1981.7) | 0.15(0.02-0.38) | 0.25(0.03-0.63) | 1.7(1.64-1.77) |
| Southeast Asia | Male | 211.03(26.27-543.95) | 1043.69(124-2590.38) | 0.18(0.02-0.47) | 0.39(0.05-0.96) | 2.54(2.47-2.62) |
| Southern Latin America | Both | 895.6(375.78-1386.56) | 1768.17(704.97-2777.48) | 2(0.82-3.09) | 2.1(0.84-3.3) | 0.15(0.05-0.24) |
| Southern Latin America | Female | 419.93(170.74-657.53) | 831.2(321.06-1326.61) | 1.66(0.68-2.61) | 1.71(0.68-2.7) | 0.05(-0.06-0.15) |
| Southern Latin America | Male | 475.67(200.03-732.46) | 936.97(371.79-1470.63) | 2.42(1-3.74) | 2.61(1.02-4.1) | 0.25(0.16-0.33) |
| Southern Sub-Saharan Africa | Both | 82.67(10.52-211.31) | 209.49(30.3-497.56) | 0.32(0.04-0.84) | 0.4(0.06-0.95) | 1.01(0.83-1.18) |
| Southern Sub-Saharan Africa | Female | 42.17(5.11-108.85) | 102.17(12.76-252.68) | 0.29(0.03-0.76) | 0.33(0.04-0.83) | 0.82(0.69-0.96) |
| Southern Sub-Saharan Africa | Male | 40.51(5.18-104.69) | 107.32(15.84-251.72) | 0.36(0.04-0.94) | 0.49(0.07-1.16) | 1.3(1.04-1.56) |
| Tropical Latin America | Both | 387.97(77.53-833.59) | 2402.9(890.19-3925.12) | 0.45(0.09-0.98) | 1(0.36-1.64) | 3.02(2.38-3.67) |
| Tropical Latin America | Female | 201.68(37.77-434.63) | 1189.27(425.98-1953.09) | 0.44(0.08-0.96) | 0.89(0.32-1.47) | 2.69(2.05-3.33) |
| Tropical Latin America | Male | 186.29(39.71-395.54) | 1213.62(468.46-1981.98) | 0.46(0.09-1) | 1.13(0.43-1.86) | 3.4(2.76-4.05) |
| Western Europe | Both | 9231.8(2731.48-16475.67) | 11104.05(2953.28-20564.82) | 1.59(0.48-2.82) | 1.16(0.32-2.11) | -1.33(-1.45--1.22) |
| Western Europe | Female | 4642.17(1357.57-8453) | 5099.76(1325.99-9597.36) | 1.33(0.4-2.38) | 0.91(0.25-1.68) | -1.56(-1.69--1.43) |
| Western Europe | Male | 4589.63(1352.12-8027.93) | 6004.29(1579.88-10976.04) | 1.97(0.57-3.46) | 1.47(0.4-2.66) | -1.25(-1.36--1.15) |
| Western Sub-Saharan Africa | Both | 92.46(10.48-244.39) | 261.9(28.68-684.2) | 0.11(0.01-0.3) | 0.16(0.02-0.41) | 1.35(1.22-1.48) |
| Western Sub-Saharan Africa | Female | 41.57(4.63-111.4) | 124.79(13.75-328.64) | 0.1(0.01-0.28) | 0.14(0.02-0.38) | 1.46(1.31-1.61) |
| Western Sub-Saharan Africa | Male | 50.9(5.55-137.41) | 137.11(15-361.19) | 0.12(0.01-0.33) | 0.17(0.02-0.44) | 1.3(1.17-1.43) |

ASDR, age-standardized death rate, SDI, socio-demographic index; UI, uncertainty interval.
