# Supplementary material for: Dietary Risk-Related Colorectal Cancer Burden: Estimates From 1990 to 2019
Source: Front Nutr. 2021 Aug 24;8:690663. doi: 10.3389/fnut.2021.690663 (PMC8421520; doi:10.3389/fnut.2021.690663)
Supplement: Supplementary file 3 [file Data_Sheet_3.zip › Supplemental tables/Table S14.docx]

**Table S14** DALYs, ASRs and change trends of colorectal cancer attributable to diet high in red meat between 1990 and 2019 by SDI, regions and sex.

| **Location** | **Sex** | **DALYs (No.×1000, 95%UI)** | | **ASR (95%UI)** | | **EAPC (95%CI)** |
| --- | --- | --- | --- | --- | --- | --- |
|  |  | **1990** | **2019** | **1990** | **2019** | **1990-2019** |
| Global | Both | 627.83(165.2-1192.28) | 1234.68(332.7-2306.84) | 15.63(4.08-29.8) | 14.95(4.02-27.99) | -0.18(-0.26--0.11) |
| Global | Female | 296.27(76.86-562.56) | 496.4(132.36-944.79) | 13.86(3.6-26.4) | 11.43(3.05-21.75) | -0.77(-0.84--0.71) |
| Global | Male | 331.56(88.53-627.39) | 738.28(198.24-1373.85) | 17.73(4.67-33.68) | 18.83(5.03-35.12) | 0.24(0.15-0.32) |
| **Sociodemographic Index** | | | | | | |
| High SDI | Both | 314.92(92.48-557.56) | 389.92(108.48-703.5) | 30.93(9.16-54.6) | 22.76(6.57-40.43) | -1.2(-1.28--1.11) |
| High SDI | Female | 145.27(42.28-258.3) | 162(44.17-299.91) | 25.53(7.52-45.21) | 17.77(5.09-32.24) | -1.39(-1.49--1.29) |
| High SDI | Male | 169.65(50.46-297.65) | 227.92(64.94-405.24) | 37.79(11.14-66.57) | 28.26(8.22-49.97) | -1.15(-1.22--1.07) |
| High-middle SDI | Both | 221.81(58.21-422.21) | 440.05(124.03-802.9) | 20.29(5.29-38.8) | 21.86(6.2-39.94) | 0.09(-0.02-0.19) |
| High-middle SDI | Female | 108.06(27.88-204.48) | 176.57(50.12-326.16) | 17.96(4.65-34.01) | 16.33(4.75-30.12) | -0.59(-0.7--0.49) |
| High-middle SDI | Male | 113.75(30.48-217.77) | 263.48(72.69-481.39) | 23.4(6.24-45.02) | 28.29(7.78-51.96) | 0.56(0.45-0.67) |
| Low SDI | Both | 6.32(0.89-16.23) | 16.78(2.64-41.4) | 2.43(0.35-6.21) | 2.92(0.47-7.18) | 0.73(0.63-0.82) |
| Low SDI | Female | 2.87(0.4-7.56) | 7.9(1.27-19.5) | 2.21(0.31-5.77) | 2.69(0.44-6.6) | 0.77(0.67-0.87) |
| Low SDI | Male | 3.45(0.47-9.12) | 8.88(1.35-22.17) | 2.63(0.37-6.93) | 3.15(0.49-7.82) | 0.72(0.63-0.81) |
| Low-middle SDI | Both | 18.65(3.61-42.69) | 75.49(17.51-155.03) | 2.83(0.54-6.52) | 5.22(1.19-10.78) | 2.39(2.28-2.49) |
| Low-middle SDI | Female | 8.93(1.68-20.61) | 32.74(7.93-68.83) | 2.76(0.51-6.39) | 4.42(1.06-9.33) | 1.8(1.74-1.86) |
| Low-middle SDI | Male | 9.72(1.84-23.11) | 42.75(9.95-88.19) | 2.9(0.54-6.92) | 6.07(1.39-12.59) | 2.92(2.77-3.07) |
| Middle SDI | Both | 65.86(8.88-161.39) | 311.98(73.75-608.41) | 5.77(0.75-14.25) | 11.96(2.77-23.44) | 3(2.81-3.2) |
| Middle SDI | Female | 31.01(4.03-75.94) | 116.98(27.02-236.75) | 5.4(0.69-13.35) | 8.75(2.01-17.74) | 1.95(1.83-2.07) |
| Middle SDI | Male | 34.85(4.59-86.32) | 195(43.91-386.71) | 6.15(0.8-15.36) | 15.37(3.39-30.7) | 3.8(3.55-4.05) |
| **Region** | | | | | | |
| Africa | Both | 10.55(1.31-27) | 29.1(3.57-74.93) | 3.4(0.43-8.73) | 4.22(0.52-10.86) | 0.92(0.85-0.99) |
| Africa | Female | 4.93(0.59-12.57) | 13.51(1.64-35.1) | 3.16(0.38-8.04) | 3.82(0.47-9.96) | 0.89(0.8-0.97) |
| Africa | Male | 5.63(0.71-14.69) | 15.59(1.9-39.91) | 3.64(0.46-9.47) | 4.64(0.56-11.85) | 0.97(0.92-1.03) |
| America | Both | 149.56(44.37-262.84) | 271.94(84.72-470.85) | 24.42(7.24-42.94) | 21.83(6.82-37.73) | -0.37(-0.42--0.32) |
| America | Female | 69.54(19.98-124.03) | 120.57(37.68-212.24) | 20.84(6.06-37.02) | 18.13(5.72-31.82) | -0.43(-0.48--0.37) |
| America | Male | 80.02(24.44-139.2) | 151.37(47.23-257.51) | 28.73(8.64-50.11) | 26.04(8.13-44.33) | -0.36(-0.41--0.3) |
| Asia | Both | 149.52(27.94-342.39) | 590.08(151.55-1129.01) | 6.91(1.29-15.8) | 11.96(3.03-23.08) | 2.25(2.07-2.42) |
| Asia | Female | 67.89(12.37-155.7) | 214.52(56.02-422.18) | 6.24(1.12-14.3) | 8.52(2.21-16.83) | 1.22(1.11-1.33) |
| Asia | Male | 81.64(14.93-188.2) | 375.56(90.91-724.4) | 7.62(1.38-17.66) | 15.59(3.69-30.27) | 2.96(2.74-3.18) |
| Europe | Both | 317.6(91.96-569.71) | 342.38(88.48-650.04) | 31.18(9.09-55.75) | 23.65(6.23-44.42) | -1.29(-1.39--1.18) |
| Europe | Female | 153.65(43.85-277.5) | 147.29(37.42-283.32) | 26.18(7.59-46.83) | 18.27(4.78-34.54) | -1.61(-1.73--1.49) |
| Europe | Male | 163.95(48.11-292.71) | 195.09(51.07-362.25) | 38.31(11.16-68.6) | 30.34(8.03-56.23) | -1.11(-1.21--1.01) |
| Andean Latin America | Both | 0.91(0.1-2.36) | 3.69(0.44-9.19) | 4.18(0.44-10.82) | 6.43(0.76-16.05) | 1.8(1.66-1.94) |
| Andean Latin America | Female | 0.48(0.05-1.23) | 1.87(0.21-4.75) | 4.36(0.46-11.14) | 6.33(0.71-16.03) | 1.49(1.35-1.63) |
| Andean Latin America | Male | 0.43(0.05-1.12) | 1.82(0.23-4.41) | 3.98(0.43-10.49) | 6.52(0.82-15.95) | 2.14(1.98-2.3) |
| Australasia | Both | 14.7(6.62-22.08) | 18.26(7.95-28.01) | 63.82(28.97-95.77) | 39.61(17.84-60.29) | -1.91(-2.09--1.73) |
| Australasia | Female | 6.7(3.01-10.12) | 8.05(3.45-12.32) | 54.66(24.86-82.24) | 33.2(14.25-50.37) | -1.92(-2.09--1.76) |
| Australasia | Male | 8(3.59-11.96) | 10.21(4.48-15.6) | 74.54(33.19-111.44) | 46.59(20.75-70.64) | -1.93(-2.13--1.73) |
| Caribbean | Both | 1.96(0.18-5.17) | 4.61(0.44-12.28) | 7.39(0.7-19.52) | 8.91(0.85-23.75) | 0.87(0.74-0.99) |
| Caribbean | Female | 1(0.09-2.65) | 2.26(0.21-6.07) | 7.35(0.67-19.36) | 8.27(0.77-22.17) | 0.62(0.51-0.72) |
| Caribbean | Male | 0.95(0.09-2.51) | 2.35(0.22-6.32) | 7.42(0.71-19.45) | 9.59(0.92-25.8) | 1.11(0.97-1.26) |
| Central Asia | Both | 8.02(2.14-15.2) | 10.91(2.93-21.06) | 15.9(4.21-30.17) | 13.59(3.49-26.08) | -0.19(-0.72-0.35) |
| Central Asia | Female | 3.91(1.04-7.4) | 5.13(1.36-9.9) | 13.9(3.69-26.31) | 11.68(3.05-22.81) | -0.29(-0.79-0.21) |
| Central Asia | Male | 4.11(1.08-7.77) | 5.79(1.52-10.97) | 18.59(4.85-35.27) | 16.03(4-30.61) | -0.11(-0.69-0.48) |
| Central Europe | Both | 37.99(8.51-76.66) | 63.02(16.26-122) | 25.72(5.77-51.88) | 31.44(8.24-60.21) | 0.99(0.83-1.14) |
| Central Europe | Female | 17(3.76-34.52) | 24.79(6.62-47.64) | 20.84(4.63-42.09) | 22.51(6-42.53) | 0.51(0.36-0.66) |
| Central Europe | Male | 20.99(4.78-42.25) | 38.22(9.73-74.33) | 31.92(7.19-64.66) | 42.33(10.81-81.96) | 1.32(1.16-1.47) |
| Central Latin America | Both | 5.03(0.68-11.89) | 20.61(3.63-48.28) | 5.5(0.72-13.21) | 8.47(1.47-19.89) | 1.59(1.54-1.65) |
| Central Latin America | Female | 2.6(0.35-6.21) | 9.61(1.55-22.55) | 5.55(0.72-13.49) | 7.41(1.19-17.43) | 1.12(1.07-1.18) |
| Central Latin America | Male | 2.43(0.33-5.71) | 11(1.9-24.68) | 5.43(0.7-12.99) | 9.66(1.63-21.77) | 2.07(1.99-2.15) |
| Central Sub-Saharan Africa | Both | 0.8(0.14-1.95) | 1.77(0.27-4.47) | 3.15(0.57-7.77) | 2.92(0.46-7.19) | -0.17(-0.49-0.16) |
| Central Sub-Saharan Africa | Female | 0.35(0.06-0.88) | 0.79(0.12-2.07) | 2.64(0.43-6.57) | 2.45(0.36-6.3) | -0.11(-0.41-0.19) |
| Central Sub-Saharan Africa | Male | 0.45(0.08-1.15) | 0.98(0.15-2.44) | 3.74(0.68-9.52) | 3.5(0.55-8.67) | -0.16(-0.49-0.17) |
| East Asia | Both | 83.02(12.43-200.52) | 436.25(115.07-813.02) | 8.61(1.25-20.89) | 20.8(5.44-38.62) | 3.72(3.43-4.01) |
| East Asia | Female | 37.49(5.27-91.33) | 149.19(41.02-286.06) | 7.77(1.07-19.01) | 13.91(3.81-26.88) | 2.38(2.2-2.57) |
| East Asia | Male | 45.53(6.76-110.77) | 287.06(73.71-547.4) | 9.59(1.38-23.64) | 28.16(7.16-53.9) | 4.59(4.24-4.95) |
| Eastern Europe | Both | 77.52(20.85-145.61) | 62.82(11.28-137.39) | 27.49(7.36-51.58) | 18.92(3.42-41.18) | -2.2(-2.54--1.86) |
| Eastern Europe | Female | 41.95(11.01-79) | 30.86(5.55-69.09) | 24.39(6.49-45.49) | 15.56(2.84-34.73) | -2.47(-2.8--2.15) |
| Eastern Europe | Male | 35.57(9.78-66.58) | 31.96(5.74-69.63) | 33.25(8.94-63.46) | 24.06(4.36-52.56) | -2.01(-2.36--1.66) |
| Eastern Sub-Saharan Africa | Both | 2.55(0.29-6.78) | 6.91(0.74-18.08) | 3.04(0.35-8) | 3.71(0.41-9.69) | 0.79(0.68-0.91) |
| Eastern Sub-Saharan Africa | Female | 1.2(0.13-3.27) | 3.29(0.34-8.76) | 2.76(0.3-7.44) | 3.38(0.36-8.96) | 0.81(0.67-0.95) |
| Eastern Sub-Saharan Africa | Male | 1.36(0.15-3.67) | 3.62(0.38-9.5) | 3.32(0.38-8.9) | 4.07(0.45-10.55) | 0.81(0.71-0.91) |
| High-income Asia Pacific | Both | 22.15(2.13-59.22) | 41.77(5.72-101.56) | 10.88(1.05-29.1) | 10.94(1.65-25.79) | -0.2(-0.32--0.07) |
| High-income Asia Pacific | Female | 9.67(0.91-25.82) | 16.28(1.97-41.03) | 8.68(0.82-23.22) | 7.7(1-18.8) | -0.68(-0.77--0.59) |
| High-income Asia Pacific | Male | 12.49(1.2-32.84) | 25.49(3.62-60.18) | 13.69(1.32-36.12) | 14.47(2.28-33.43) | 0(-0.16-0.15) |
| High-income North America | Both | 110.89(32.93-193.18) | 144.67(42.18-251.56) | 32.99(9.96-57.08) | 25.35(7.51-43.92) | -1.08(-1.19--0.96) |
| High-income North America | Female | 51.08(14.91-90.69) | 60.93(16.98-111.85) | 27.38(8.29-47.94) | 20.18(5.87-36.38) | -1.2(-1.31--1.08) |
| High-income North America | Male | 59.81(18.23-103.22) | 83.74(24.13-143.66) | 40.07(12.22-69.21) | 31.12(9.08-53.21) | -1.07(-1.19--0.94) |
| North Africa and Middle East | Both | 8.53(0.8-23.2) | 23.44(2.22-62.63) | 4.5(0.43-12.31) | 4.98(0.48-13.24) | 0.51(0.24-0.78) |
| North Africa and Middle East | Female | 4.03(0.37-10.8) | 10.37(0.98-28.14) | 4.32(0.4-11.55) | 4.54(0.43-12.28) | 0.32(0.08-0.55) |
| North Africa and Middle East | Male | 4.5(0.42-12.71) | 13.07(1.23-35) | 4.66(0.44-13.16) | 5.41(0.52-14.36) | 0.68(0.38-0.98) |
| Oceania | Both | 0.19(0.02-0.48) | 0.45(0.05-1.21) | 5.47(0.66-14.24) | 5.65(0.59-15.03) | -0.02(-0.14-0.11) |
| Oceania | Female | 0.08(0.01-0.21) | 0.2(0.02-0.54) | 5.02(0.56-13.28) | 5.16(0.51-14.08) | -0.04(-0.17-0.09) |
| Oceania | Male | 0.11(0.01-0.28) | 0.26(0.03-0.67) | 5.89(0.71-15.86) | 6.13(0.67-16.06) | 0(-0.12-0.13) |
| South Asia | Both | 7.51(2.04-16.22) | 24.35(5.85-55.57) | 1.23(0.32-2.67) | 1.66(0.4-3.79) | 0.86(0.73-1) |
| South Asia | Female | 3.49(0.94-7.66) | 11.77(2.88-26.93) | 1.18(0.3-2.61) | 1.59(0.39-3.64) | 0.8(0.61-0.99) |
| South Asia | Male | 4.02(1.07-8.93) | 12.57(2.89-28.82) | 1.27(0.33-2.85) | 1.73(0.4-3.98) | 0.94(0.84-1.03) |
| Southeast Asia | Both | 11.63(1.43-29.88) | 49.49(5.93-123.35) | 4.08(0.51-10.45) | 7.56(0.91-18.86) | 2.1(2.04-2.17) |
| Southeast Asia | Female | 5.43(0.64-14.11) | 20.03(2.43-50.89) | 3.65(0.44-9.39) | 5.85(0.71-14.94) | 1.61(1.55-1.67) |
| Southeast Asia | Male | 6.2(0.75-16.13) | 29.46(3.51-72.47) | 4.56(0.56-11.83) | 9.47(1.12-23.4) | 2.5(2.42-2.58) |
| Southern Latin America | Both | 20.6(9.15-31.55) | 38.06(16.08-58.94) | 44.3(19.61-67.88) | 46.7(19.81-72.14) | 0.17(0.1-0.24) |
| Southern Latin America | Female | 9.19(3.97-14.08) | 16.77(7.11-26.15) | 36.16(15.75-55.36) | 37.4(15.96-57.87) | 0.12(0.04-0.2) |
| Southern Latin America | Male | 11.41(5.09-17.32) | 21.3(8.97-32.86) | 54.14(23.86-82.26) | 57.9(24.34-89.35) | 0.21(0.14-0.28) |
| Southern Sub-Saharan Africa | Both | 2.22(0.3-5.42) | 5.49(0.88-12.74) | 7.39(0.98-18.32) | 9.1(1.4-21.36) | 1.09(0.9-1.28) |
| Southern Sub-Saharan Africa | Female | 1.05(0.14-2.61) | 2.48(0.35-5.98) | 6.38(0.82-16.03) | 7.36(1.01-17.87) | 1.01(0.83-1.18) |
| Southern Sub-Saharan Africa | Male | 1.16(0.15-2.89) | 3.01(0.47-6.86) | 8.58(1.13-21.95) | 11.38(1.67-26.56) | 1.24(0.97-1.5) |
| Tropical Latin America | Both | 10.57(2.21-22.08) | 61.17(23.98-97.09) | 10.65(2.16-22.52) | 24.61(9.58-39.24) | 3.15(2.46-3.84) |
| Tropical Latin America | Female | 5.36(1.06-11.25) | 29.5(11.34-47.11) | 10.29(1.99-21.76) | 22.06(8.47-35.21) | 2.88(2.2-3.56) |
| Tropical Latin America | Male | 5.21(1.16-10.79) | 31.67(12.69-50.81) | 11.03(2.4-23.04) | 27.64(10.92-44.43) | 3.44(2.74-4.14) |
| Western Europe | Both | 198.62(60.79-339.98) | 210.07(59.87-372.02) | 36.14(11.18-61.52) | 25.63(7.6-44.82) | -1.42(-1.52--1.33) |
| Western Europe | Female | 93.14(28.56-162.05) | 89(25.05-160.12) | 29.98(9.33-50.98) | 20.02(5.89-34.96) | -1.63(-1.73--1.54) |
| Western Europe | Male | 105.47(32.75-180) | 121.07(34.66-215.93) | 44.13(13.73-75.31) | 31.99(9.46-56.32) | -1.35(-1.45--1.26) |
| Western Sub-Saharan Africa | Both | 2.43(0.26-6.5) | 6.85(0.71-18.05) | 2.61(0.29-6.94) | 3.39(0.37-8.86) | 1.16(1.04-1.28) |
| Western Sub-Saharan Africa | Female | 1.05(0.11-2.84) | 3.22(0.34-8.46) | 2.34(0.25-6.28) | 3.07(0.33-8.09) | 1.24(1.09-1.38) |
| Western Sub-Saharan Africa | Male | 1.37(0.14-3.73) | 3.64(0.38-9.63) | 2.87(0.31-7.73) | 3.75(0.41-9.91) | 1.16(1.04-1.27) |

ASDR, age-standardized death rate; DALYs, disability-adjusted life years; SDI, socio-demographic index; UI, uncertainty interval.
