# Supplementary material for: Dietary Risk-Related Colorectal Cancer Burden: Estimates From 1990 to 2019
Source: Front Nutr. 2021 Aug 24;8:690663. doi: 10.3389/fnut.2021.690663 (PMC8421520; doi:10.3389/fnut.2021.690663)
Supplement: Supplementary file 3 [file Data_Sheet_3.zip › Supplemental tables/Table S15.docx]

**Table S15** Deaths, ASDRs and change trends of colorectal cancer attributable to diet high in processed meat between 1990 and 2019 by SDI, regions and sex

| **Location** | **Sex** | **Deaths (95%UI)** | | **ASDR (95%UI)** | | **EAPC (95%CI)** |
| --- | --- | --- | --- | --- | --- | --- |
|  |  | **1990** | **2019** | **1990** | **2019** | **1990-2019** |
| Global | Both | 20185.18(7514.54-30917.34) | 33927.57(11645.16-52095.67) | 0.56(0.2-0.87) | 0.43(0.15-0.66) | -1.08(-1.2--0.95) |
| Global | Female | 10612.48(4011.25-16494.03) | 16191.08(5606.86-24925.08) | 0.53(0.2-0.82) | 0.37(0.13-0.57) | -1.41(-1.55--1.26) |
| Global | Male | 9572.69(3517.42-14555.91) | 17736.48(6025.17-27213.87) | 0.61(0.22-0.93) | 0.5(0.17-0.77) | -0.77(-0.87--0.67) |
| **Sociodemographic Index** | | | | | | |
| High SDI | Both | 12344.14(4087.4-18742.78) | 17957.26(6062.65-27711.02) | 1.18(0.39-1.79) | 0.91(0.32-1.41) | -1.04(-1.19--0.88) |
| High SDI | Female | 6356.27(2126.6-9731.83) | 8517.46(2876.18-13282.31) | 1.01(0.35-1.55) | 0.76(0.27-1.18) | -1.18(-1.36--1.01) |
| High SDI | Male | 5987.87(1921.62-9071.07) | 9439.8(3186.47-14497.35) | 1.41(0.44-2.14) | 1.1(0.37-1.69) | -1.01(-1.15--0.88) |
| High-middle SDI | Both | 6473.44(2283.01-10458.6) | 10455.98(2857.06-16318.91) | 0.63(0.22-1.02) | 0.52(0.14-0.81) | -0.88(-1.02--0.73) |
| High-middle SDI | Female | 3577.24(1322.35-5877.7) | 5105.57(1435.49-7959.84) | 0.6(0.22-0.99) | 0.44(0.12-0.69) | -1.29(-1.43--1.15) |
| High-middle SDI | Male | 2896.2(988.51-4588.89) | 5350.41(1408.4-8411.99) | 0.69(0.23-1.08) | 0.62(0.16-0.98) | -0.47(-0.62--0.31) |
| Low SDI | Both | 208.16(46.97-364.09) | 586.76(138.5-986.79) | 0.09(0.02-0.16) | 0.12(0.03-0.2) | 0.89(0.84-0.94) |
| Low SDI | Female | 96.26(20.44-180.09) | 285.81(64.42-484.27) | 0.09(0.02-0.16) | 0.11(0.03-0.19) | 0.97(0.9-1.04) |
| Low SDI | Male | 111.89(24.99-197.03) | 300.95(69.26-511.36) | 0.1(0.02-0.18) | 0.12(0.03-0.21) | 0.84(0.79-0.88) |
| Low-middle SDI | Both | 425.88(172.4-660.88) | 1620.04(653.61-2474.76) | 0.08(0.03-0.12) | 0.12(0.05-0.19) | 1.79(1.71-1.86) |
| Low-middle SDI | Female | 207.92(82.39-327.56) | 801.13(332.08-1230.38) | 0.07(0.03-0.12) | 0.12(0.05-0.18) | 1.63(1.54-1.73) |
| Low-middle SDI | Male | 217.96(89.4-341.4) | 818.9(324.09-1280.63) | 0.08(0.03-0.12) | 0.13(0.05-0.2) | 1.95(1.88-2.02) |
| Middle SDI | Both | 725.38(307.21-1102.05) | 3293.44(1028.1-5402.14) | 0.08(0.03-0.11) | 0.14(0.04-0.22) | 2.47(2.31-2.63) |
| Middle SDI | Female | 370.43(149.04-578.22) | 1474.11(437.39-2456.42) | 0.07(0.03-0.12) | 0.12(0.04-0.19) | 1.86(1.74-1.99) |
| Middle SDI | Male | 354.95(156.75-540.17) | 1819.33(588.06-3074.32) | 0.08(0.03-0.12) | 0.16(0.05-0.27) | 3.05(2.86-3.24) |
| **Region** | | | | | | |
| Africa | Both | 285.59(48.46-512.01) | 848.42(137.06-1468.76) | 0.11(0.02-0.19) | 0.14(0.02-0.25) | 1.21(1.13-1.3) |
| Africa | Female | 141.8(23.26-262.08) | 433.68(71.31-762.27) | 0.11(0.02-0.19) | 0.14(0.02-0.25) | 1.29(1.18-1.4) |
| Africa | Male | 143.79(25.17-259.44) | 414.75(67.49-723.73) | 0.11(0.02-0.19) | 0.14(0.02-0.25) | 1.16(1.09-1.23) |
| America | Both | 4771.08(1580.3-7291.39) | 8007.96(2840.45-12421.29) | 0.79(0.26-1.21) | 0.62(0.22-0.96) | -0.99(-1.07--0.92) |
| America | Female | 2398.73(776.06-3683.62) | 3864.31(1355.38-6030.2) | 0.7(0.22-1.07) | 0.54(0.19-0.84) | -1.03(-1.11--0.95) |
| America | Male | 2372.34(788.01-3594.98) | 4143.65(1528.37-6445.44) | 0.92(0.3-1.4) | 0.72(0.26-1.12) | -1.01(-1.11--0.92) |
| Asia | Both | 3069.53(1095.41-4608.73) | 9266.4(2883.78-14630.62) | 0.17(0.06-0.26) | 0.21(0.06-0.33) | 0.73(0.6-0.86) |
| Asia | Female | 1470.1(512.52-2245.83) | 4090.78(1307.56-6525.67) | 0.16(0.05-0.24) | 0.17(0.05-0.27) | 0.28(0.18-0.38) |
| Asia | Male | 1599.42(570.82-2427.15) | 5175.62(1620.55-8197.34) | 0.18(0.06-0.28) | 0.25(0.08-0.39) | 1.12(0.97-1.28) |
| Europe | Both | 12044.47(4302.37-18853.96) | 15775.3(4883.08-24171.45) | 1.17(0.42-1.84) | 0.98(0.31-1.5) | -0.85(-1.05--0.65) |
| Europe | Female | 6594.37(2347.49-10434.81) | 7787.76(2544.79-11943.96) | 1.04(0.38-1.65) | 0.81(0.27-1.24) | -1.15(-1.35--0.94) |
| Europe | Male | 5450.1(1897.38-8487.03) | 7987.54(2389.61-12203.71) | 1.38(0.47-2.13) | 1.21(0.36-1.84) | -0.65(-0.84--0.46) |
| Andean Latin America | Both | 9.92(5.25-14.59) | 44.42(17.11-74.55) | 0.05(0.03-0.07) | 0.08(0.03-0.13) | 2.03(1.87-2.19) |
| Andean Latin America | Female | 5.54(2.71-8.34) | 24.61(8.63-42.82) | 0.05(0.03-0.08) | 0.08(0.03-0.15) | 1.82(1.66-1.97) |
| Andean Latin America | Male | 4.38(2.37-6.43) | 19.81(7.71-33.3) | 0.04(0.03-0.07) | 0.07(0.03-0.12) | 2.3(2.12-2.48) |
| Australasia | Both | 278.87(69.3-434.54) | 449.88(119.54-692.84) | 1.21(0.31-1.89) | 0.89(0.24-1.36) | -1.27(-1.41--1.14) |
| Australasia | Female | 141.96(36.94-220.79) | 218.68(62.89-339.96) | 1.1(0.29-1.7) | 0.78(0.23-1.2) | -1.35(-1.47--1.23) |
| Australasia | Male | 136.91(29.62-218.82) | 231.21(56.67-361.13) | 1.35(0.29-2.17) | 1(0.25-1.55) | -1.24(-1.41--1.08) |
| Caribbean | Both | 38.53(7.35-66.69) | 100.34(18.13-181.26) | 0.15(0.03-0.26) | 0.19(0.04-0.35) | 1.02(0.95-1.1) |
| Caribbean | Female | 21.37(3.82-37.83) | 54.89(9.37-100.53) | 0.16(0.03-0.29) | 0.2(0.03-0.36) | 0.85(0.78-0.92) |
| Caribbean | Male | 17.16(3.53-30.16) | 45.45(8.73-83.68) | 0.14(0.03-0.25) | 0.19(0.04-0.35) | 1.21(1.12-1.3) |
| Central Asia | Both | 190.48(33.01-306.01) | 280.08(49.69-457.47) | 0.4(0.07-0.65) | 0.4(0.07-0.66) | 0.31(0.01-0.61) |
| Central Asia | Female | 104.15(19.32-167.29) | 147.5(28.02-239.9) | 0.38(0.07-0.61) | 0.37(0.07-0.61) | 0.27(0-0.54) |
| Central Asia | Male | 86.33(14.29-140.94) | 132.59(23.3-222.14) | 0.44(0.07-0.72) | 0.44(0.07-0.74) | 0.32(-0.02-0.66) |
| Central Europe | Both | 1001.88(177.55-1614.64) | 2007.27(436.26-3288.98) | 0.7(0.12-1.13) | 0.93(0.21-1.52) | 1.35(1.18-1.51) |
| Central Europe | Female | 507.22(92.94-816.34) | 913.28(207.07-1484.33) | 0.61(0.11-0.99) | 0.73(0.18-1.18) | 0.85(0.69-1) |
| Central Europe | Male | 494.66(79.33-811.72) | 1093.99(225.11-1806.91) | 0.81(0.13-1.33) | 1.21(0.25-1.99) | 1.79(1.61-1.98) |
| Central Latin America | Both | 77.59(15.26-130.99) | 341.74(64.94-602.65) | 0.1(0.02-0.16) | 0.15(0.03-0.26) | 1.55(1.49-1.6) |
| Central Latin America | Female | 43.94(8.71-74.46) | 179.11(33.49-316.2) | 0.11(0.02-0.18) | 0.14(0.03-0.25) | 1.11(1.04-1.18) |
| Central Latin America | Male | 33.65(6.58-58.12) | 162.63(30.64-294.97) | 0.09(0.02-0.15) | 0.15(0.03-0.27) | 2.06(2-2.13) |
| Central Sub-Saharan Africa | Both | 24.21(3.39-47.22) | 53.92(7.73-105.23) | 0.11(0.02-0.22) | 0.11(0.02-0.21) | -0.22(-0.56-0.13) |
| Central Sub-Saharan Africa | Female | 11.49(1.55-23.38) | 26.53(3.78-53.24) | 0.1(0.01-0.21) | 0.1(0.01-0.19) | -0.18(-0.5-0.14) |
| Central Sub-Saharan Africa | Male | 12.73(1.83-25.94) | 27.4(3.8-55.49) | 0.13(0.02-0.26) | 0.12(0.02-0.25) | -0.17(-0.52-0.18) |
| East Asia | Both | 622.38(277.28-987.27) | 3377.58(700.51-6070.53) | 0.08(0.03-0.12) | 0.17(0.04-0.3) | 3.41(3.12-3.7) |
| East Asia | Female | 302.82(120.63-504.95) | 1352.41(249.07-2510.92) | 0.07(0.03-0.12) | 0.13(0.02-0.24) | 2.51(2.28-2.74) |
| East Asia | Male | 319.56(144.61-511.89) | 2025.17(437.27-3771.66) | 0.08(0.04-0.13) | 0.22(0.05-0.4) | 4.13(3.79-4.46) |
| Eastern Europe | Both | 3312.71(1174.57-5893.22) | 3346.69(1057.94-5208.39) | 1.19(0.42-2.11) | 0.96(0.3-1.5) | -1.2(-1.5--0.9) |
| Eastern Europe | Female | 1973.85(702.56-3573.4) | 1846.92(592.41-2918.49) | 1.09(0.39-1.97) | 0.83(0.27-1.31) | -1.42(-1.7--1.15) |
| Eastern Europe | Male | 1338.86(474.97-2344.88) | 1499.77(449.18-2372.43) | 1.42(0.51-2.43) | 1.2(0.36-1.89) | -1.06(-1.39--0.72) |
| Eastern Sub-Saharan Africa | Both | 72.61(11.24-134.13) | 200.41(30.04-369.53) | 0.1(0.02-0.18) | 0.13(0.02-0.23) | 0.95(0.84-1.07) |
| Eastern Sub-Saharan Africa | Female | 34.93(5.09-69.32) | 100.03(14.53-181.58) | 0.09(0.01-0.18) | 0.12(0.02-0.22) | 1.01(0.88-1.14) |
| Eastern Sub-Saharan Africa | Male | 37.68(5.88-70.44) | 100.37(15.33-192.81) | 0.11(0.02-0.2) | 0.13(0.02-0.25) | 0.93(0.83-1.02) |
| High-income Asia Pacific | Both | 1480.18(384.47-2309.84) | 3002.72(761.2-4758.47) | 0.74(0.19-1.16) | 0.66(0.18-1.02) | -0.5(-0.76--0.23) |
| High-income Asia Pacific | Female | 688.68(172.91-1082.99) | 1344.61(355.77-2205.49) | 0.61(0.15-0.95) | 0.51(0.15-0.8) | -0.74(-0.97--0.51) |
| High-income Asia Pacific | Male | 791.5(206.21-1253.05) | 1658.11(406.93-2615.98) | 0.92(0.23-1.46) | 0.83(0.21-1.31) | -0.42(-0.71--0.13) |
| High-income North America | Both | 4261.4(1413.58-6493.55) | 6315.86(2234.32-10038.81) | 1.19(0.4-1.82) | 1(0.36-1.6) | -0.82(-0.92--0.73) |
| High-income North America | Female | 2126.24(685.25-3256.13) | 2978.98(1047.34-4702.45) | 1.01(0.33-1.54) | 0.84(0.3-1.35) | -0.81(-0.91--0.7) |
| High-income North America | Male | 2135.15(725.58-3260.42) | 3336.88(1195.14-5200.12) | 1.46(0.49-2.22) | 1.19(0.43-1.85) | -0.96(-1.06--0.86) |
| North Africa and Middle East | Both | 116.42(32.02-207.61) | 410.73(95.49-725.2) | 0.07(0.02-0.13) | 0.1(0.02-0.17) | 1.45(1.2-1.71) |
| North Africa and Middle East | Female | 58.37(14.72-107.28) | 196.39(42.26-353.83) | 0.07(0.02-0.13) | 0.1(0.02-0.17) | 1.34(1.09-1.6) |
| North Africa and Middle East | Male | 58.06(16.56-106.54) | 214.34(52.82-381.05) | 0.07(0.02-0.13) | 0.1(0.03-0.18) | 1.57(1.31-1.83) |
| Oceania | Both | 1.66(0.56-2.93) | 4.63(1.4-8.39) | 0.06(0.02-0.1) | 0.07(0.02-0.13) | 0.58(0.49-0.66) |
| Oceania | Female | 0.79(0.23-1.45) | 2.19(0.6-4.11) | 0.06(0.02-0.11) | 0.07(0.02-0.13) | 0.56(0.46-0.66) |
| Oceania | Male | 0.87(0.29-1.57) | 2.44(0.77-4.51) | 0.06(0.02-0.11) | 0.07(0.03-0.13) | 0.61(0.52-0.69) |
| South Asia | Both | 339.04(177.13-506.85) | 1421.33(852.44-2054.02) | 0.07(0.03-0.1) | 0.11(0.06-0.15) | 1.6(1.51-1.69) |
| South Asia | Female | 154.02(82.69-226.23) | 684.53(397.31-996.41) | 0.06(0.03-0.1) | 0.1(0.06-0.15) | 1.56(1.44-1.69) |
| South Asia | Male | 185.02(94.51-281.33) | 736.8(431.63-1081.55) | 0.07(0.03-0.11) | 0.11(0.07-0.17) | 1.68(1.61-1.75) |
| Southeast Asia | Both | 136.65(73.27-195.37) | 592.49(263.88-926.29) | 0.06(0.03-0.08) | 0.1(0.05-0.16) | 2.06(1.94-2.18) |
| Southeast Asia | Female | 68.25(34.59-99.59) | 274.95(111.78-446.9) | 0.05(0.03-0.08) | 0.09(0.04-0.14) | 1.78(1.67-1.9) |
| Southeast Asia | Male | 68.4(37.84-99.27) | 317.54(145.25-494.23) | 0.06(0.03-0.09) | 0.12(0.06-0.18) | 2.34(2.22-2.47) |
| Southern Latin America | Both | 280.38(46.57-456.07) | 724.8(163.06-1151.55) | 0.63(0.1-1.02) | 0.86(0.19-1.37) | 1.16(1.09-1.23) |
| Southern Latin America | Female | 143.92(25.45-235.34) | 369.73(87.49-586.8) | 0.57(0.1-0.94) | 0.76(0.18-1.21) | 1.02(0.94-1.11) |
| Southern Latin America | Male | 136.46(22.34-226.08) | 355.07(77.85-573.33) | 0.7(0.11-1.16) | 0.99(0.22-1.59) | 1.32(1.26-1.39) |
| Southern Sub-Saharan Africa | Both | 32.27(5.89-59.81) | 85.33(13.78-151.57) | 0.12(0.02-0.23) | 0.16(0.03-0.28) | 1.08(0.9-1.27) |
| Southern Sub-Saharan Africa | Female | 17.34(3.01-32.56) | 44.44(6.99-79.71) | 0.12(0.02-0.22) | 0.14(0.02-0.26) | 0.99(0.84-1.13) |
| Southern Sub-Saharan Africa | Male | 14.93(2.83-29) | 40.89(6.64-73.4) | 0.13(0.03-0.26) | 0.18(0.03-0.33) | 1.29(1.02-1.56) |
| Tropical Latin America | Both | 111.42(19.61-193.91) | 500.61(68.01-871.91) | 0.13(0.02-0.22) | 0.21(0.03-0.36) | 2.04(1.92-2.17) |
| Tropical Latin America | Female | 61.84(10.34-110.44) | 266.89(35.37-471.61) | 0.13(0.02-0.24) | 0.2(0.03-0.36) | 1.77(1.65-1.89) |
| Tropical Latin America | Male | 49.58(9.45-87.02) | 233.72(32.85-409.36) | 0.12(0.02-0.21) | 0.22(0.03-0.38) | 2.4(2.26-2.53) |
| Western Europe | Both | 7676.41(2453.57-11668.63) | 10299.08(3467.29-15699.14) | 1.31(0.42-2) | 1.05(0.36-1.61) | -0.94(-1.14--0.74) |
| Western Europe | Female | 4086.71(1313.44-6247.27) | 4971.45(1625.16-7691.09) | 1.14(0.39-1.75) | 0.86(0.29-1.33) | -1.18(-1.39--0.96) |
| Western Europe | Male | 3589.7(1155.87-5433.22) | 5327.63(1721.38-8071.77) | 1.56(0.49-2.35) | 1.28(0.41-1.95) | -0.82(-1.01--0.63) |
| Western Sub-Saharan Africa | Both | 120.16(16.05-221.87) | 367.65(55.34-643.77) | 0.15(0.02-0.27) | 0.21(0.03-0.37) | 1.57(1.45-1.68) |
| Western Sub-Saharan Africa | Female | 59.06(7.86-114.39) | 192.97(31.01-344.25) | 0.15(0.02-0.28) | 0.22(0.03-0.39) | 1.69(1.56-1.82) |
| Western Sub-Saharan Africa | Male | 61.1(8.04-115.94) | 174.68(24.75-313.63) | 0.15(0.02-0.28) | 0.21(0.03-0.38) | 1.48(1.36-1.59) |

ASDR, age-standardized death rate, SDI, socio-demographic index; UI, uncertainty interval.
