# Supplementary material for: Dietary Risk-Related Colorectal Cancer Burden: Estimates From 1990 to 2019
Source: Front Nutr. 2021 Aug 24;8:690663. doi: 10.3389/fnut.2021.690663 (PMC8421520; doi:10.3389/fnut.2021.690663)
Supplement: Supplementary file 3 [file Data_Sheet_3.zip › Supplemental tables/Table S16.docx]

**Table S16** DALYs, ASRs and change trends of colorectal cancer attributable to diet high in processed meat between 1990 and 2019 by SDI, regions and sex.

| **Location** | **Sex** | **DALYs (No.×1000, 95%UI)** | | **ASR (95%UI)** | | **EAPC (95%CI)** |
| --- | --- | --- | --- | --- | --- | --- |
|  |  | **1990** | **2019** | **1990** | **2019** | **1990-2019** |
| Global | Both | 462.27(176.03-707.33) | 735.04(262.81-1127.2) | 11.71(4.48-17.96) | 8.96(3.2-13.74) | -1.06(-1.16--0.96) |
| Global | Female | 230.27(88.35-356.8) | 329.55(116.74-509.26) | 10.86(4.15-16.83) | 7.57(2.68-11.7) | -1.42(-1.52--1.32) |
| Global | Male | 232(87.63-352.9) | 405.5(139.43-623.1) | 12.72(4.78-19.31) | 10.52(3.6-16.13) | -0.75(-0.85--0.66) |
| **Sociodemographic Index** | | | | | | |
| High SDI | Both | 266(92.05-404.8) | 358.29(126.86-554.32) | 25.99(9.05-39.58) | 20.47(7.27-31.7) | -0.96(-1.09--0.84) |
| High SDI | Female | 127.79(44.15-195.51) | 157.38(56.11-245.57) | 22.13(7.7-33.95) | 16.82(6.07-26.54) | -1.09(-1.22--0.97) |
| High SDI | Male | 138.21(44.5-209.51) | 200.91(70.1-308.73) | 30.93(10.01-46.82) | 24.53(8.68-37.77) | -0.94(-1.06--0.81) |
| High-middle SDI | Both | 157(58.19-253.73) | 228.84(62.89-356.76) | 14.41(5.27-23.23) | 11.3(3.11-17.61) | -1.08(-1.23--0.93) |
| High-middle SDI | Female | 83.22(30.02-137.16) | 104.94(29.98-163.22) | 13.77(4.99-22.71) | 9.56(2.74-14.89) | -1.55(-1.69--1.4) |
| High-middle SDI | Male | 73.78(25.59-118.2) | 123.9(32.94-194.53) | 15.33(5.23-24.38) | 13.38(3.56-21.03) | -0.66(-0.83--0.5) |
| Low SDI | Both | 6.05(1.35-10.55) | 16.86(3.95-28.48) | 2.29(0.52-4.02) | 2.88(0.67-4.84) | 0.85(0.8-0.9) |
| Low SDI | Female | 2.86(0.63-5.33) | 8.25(1.87-13.97) | 2.17(0.47-4.06) | 2.76(0.62-4.68) | 0.9(0.83-0.97) |
| Low SDI | Male | 3.19(0.7-5.65) | 8.61(1.93-14.78) | 2.41(0.53-4.24) | 3.01(0.69-5.14) | 0.83(0.8-0.86) |
| Low-middle SDI | Both | 12.2(4.98-18.86) | 43.43(16.89-66.78) | 1.85(0.75-2.87) | 2.99(1.18-4.59) | 1.8(1.72-1.89) |
| Low-middle SDI | Female | 5.99(2.42-9.41) | 21.3(8.65-32.84) | 1.83(0.73-2.88) | 2.85(1.17-4.4) | 1.63(1.52-1.74) |
| Low-middle SDI | Male | 6.21(2.53-9.68) | 22.13(8.24-34.91) | 1.87(0.76-2.94) | 3.15(1.21-4.97) | 1.99(1.91-2.06) |
| Middle SDI | Both | 20.86(8.54-31.83) | 87.33(25.25-143.83) | 1.83(0.77-2.79) | 3.36(0.99-5.54) | 2.53(2.35-2.71) |
| Middle SDI | Female | 10.32(3.99-16.24) | 37.54(10.54-62.72) | 1.8(0.71-2.82) | 2.81(0.8-4.68) | 1.84(1.69-1.99) |
| Middle SDI | Male | 10.53(4.48-16.15) | 49.79(14.59-84.67) | 1.86(0.81-2.84) | 3.94(1.2-6.65) | 3.14(2.93-3.35) |
| **Region** | | | | | | |
| Africa | Both | 8.03(1.31-14.49) | 23.72(3.73-41.32) | 2.56(0.43-4.6) | 3.38(0.54-5.84) | 1.15(1.06-1.24) |
| Africa | Female | 3.93(0.63-7.29) | 11.91(1.98-21.09) | 2.49(0.41-4.59) | 3.3(0.54-5.82) | 1.22(1.1-1.35) |
| Africa | Male | 4.1(0.68-7.44) | 11.81(1.81-20.84) | 2.62(0.45-4.75) | 3.45(0.55-6.04) | 1.1(1.03-1.16) |
| America | Both | 105.99(36.52-161.93) | 178.7(64.14-279.64) | 17.35(5.98-26.47) | 14.25(5.11-22.35) | -0.77(-0.83--0.71) |
| America | Female | 50.67(16.8-77.5) | 81.51(29.17-128.11) | 15.08(5.05-23.11) | 12.11(4.33-19.07) | -0.84(-0.9--0.78) |
| America | Male | 55.32(19.01-84.31) | 97.19(35.28-152.85) | 20.09(6.89-30.57) | 16.68(6.05-26.2) | -0.75(-0.83--0.67) |
| Asia | Both | 82.13(29.97-122.67) | 223.68(69.99-352.6) | 3.87(1.38-5.81) | 4.6(1.43-7.24) | 0.65(0.56-0.75) |
| Asia | Female | 38.17(13.86-57.68) | 94.07(30.56-149.18) | 3.56(1.27-5.43) | 3.76(1.22-5.94) | 0.15(0.1-0.2) |
| Asia | Male | 43.96(16.09-66.14) | 129.61(39.18-207.12) | 4.2(1.49-6.37) | 5.5(1.66-8.78) | 1.06(0.93-1.19) |
| Europe | Both | 265.78(98.46-420.45) | 308.34(103.06-471.85) | 25.99(9.61-41.24) | 21(7.04-32.12) | -0.99(-1.17--0.82) |
| Europe | Female | 137.33(51.78-219.4) | 141.78(47.12-216.94) | 23.12(8.64-37.13) | 17.21(5.76-26.24) | -1.31(-1.48--1.14) |
| Europe | Male | 128.45(46.14-201.33) | 166.55(52.57-253.23) | 30.22(10.75-47.26) | 25.76(8.2-39.17) | -0.78(-0.97--0.59) |
| Andean Latin America | Both | 0.27(0.13-0.41) | 1.13(0.35-1.99) | 1.2(0.59-1.79) | 1.95(0.63-3.4) | 2.07(1.9-2.25) |
| Andean Latin America | Female | 0.15(0.06-0.23) | 0.6(0.17-1.08) | 1.28(0.58-1.98) | 2(0.59-3.59) | 1.85(1.67-2.02) |
| Andean Latin America | Male | 0.12(0.06-0.19) | 0.53(0.17-0.94) | 1.11(0.57-1.67) | 1.89(0.63-3.3) | 2.34(2.15-2.54) |
| Australasia | Both | 6.51(1.69-10.03) | 9.25(2.48-14.05) | 28.36(7.42-43.71) | 20.02(5.48-30.25) | -1.4(-1.53--1.26) |
| Australasia | Female | 3.15(0.84-4.8) | 4.25(1.32-6.48) | 25.71(6.86-39.04) | 17.42(5.4-26.33) | -1.53(-1.65--1.4) |
| Australasia | Male | 3.36(0.81-5.33) | 5(1.35-7.66) | 31.39(7.44-49.65) | 22.82(6.28-34.81) | -1.32(-1.48--1.15) |
| Caribbean | Both | 0.94(0.17-1.61) | 2.28(0.39-4.18) | 3.51(0.63-6.08) | 4.42(0.75-8.09) | 0.99(0.91-1.07) |
| Caribbean | Female | 0.51(0.08-0.9) | 1.2(0.19-2.21) | 3.71(0.62-6.57) | 4.38(0.69-8.09) | 0.79(0.72-0.86) |
| Caribbean | Male | 0.43(0.08-0.75) | 1.09(0.2-2.03) | 3.3(0.64-5.8) | 4.44(0.81-8.25) | 1.22(1.12-1.31) |
| Central Asia | Both | 5.68(1.04-9.04) | 7.88(1.51-12.82) | 11.2(1.95-17.88) | 9.77(1.8-15.98) | -0.31(-0.6--0.02) |
| Central Asia | Female | 2.93(0.58-4.67) | 3.96(0.79-6.41) | 10.39(2.08-16.5) | 8.99(1.83-14.56) | -0.37(-0.62--0.12) |
| Central Asia | Male | 2.75(0.51-4.47) | 3.92(0.75-6.55) | 12.25(2.05-19.98) | 10.74(1.88-18) | -0.26(-0.59-0.08) |
| Central Europe | Both | 24.43(4.46-39.24) | 43.81(10.13-71.02) | 16.57(3.09-26.55) | 21.77(5.26-35.18) | 1.29(1.12-1.47) |
| Central Europe | Female | 11.81(2.31-18.89) | 18.64(4.64-29.92) | 14.47(2.83-23.14) | 16.79(4.36-26.87) | 0.79(0.63-0.96) |
| Central Europe | Male | 12.62(2.08-20.59) | 25.17(5.48-41.25) | 19.27(3.22-31.37) | 27.86(6.16-45.61) | 1.69(1.5-1.88) |
| Central Latin America | Both | 2.11(0.39-3.55) | 8.86(1.58-15.61) | 2.28(0.43-3.82) | 3.63(0.65-6.4) | 1.75(1.68-1.81) |
| Central Latin America | Female | 1.16(0.22-1.96) | 4.48(0.8-7.97) | 2.45(0.47-4.13) | 3.44(0.62-6.11) | 1.33(1.25-1.41) |
| Central Latin America | Male | 0.95(0.17-1.64) | 4.38(0.76-7.93) | 2.09(0.39-3.59) | 3.84(0.67-6.97) | 2.21(2.15-2.28) |
| Central Sub-Saharan Africa | Both | 0.72(0.09-1.41) | 1.6(0.22-3.18) | 2.79(0.39-5.44) | 2.59(0.37-5.09) | -0.24(-0.58-0.09) |
| Central Sub-Saharan Africa | Female | 0.35(0.04-0.7) | 0.77(0.11-1.54) | 2.51(0.34-5.07) | 2.33(0.33-4.67) | -0.19(-0.51-0.12) |
| Central Sub-Saharan Africa | Male | 0.37(0.05-0.76) | 0.83(0.11-1.69) | 3.11(0.45-6.3) | 2.92(0.41-5.89) | -0.25(-0.6-0.09) |
| East Asia | Both | 17.96(7.58-28.87) | 88.8(16.46-160.03) | 1.87(0.81-2.99) | 4.24(0.8-7.64) | 3.56(3.24-3.88) |
| East Asia | Female | 8.51(3.18-14.52) | 33.77(5.53-62.88) | 1.77(0.68-2.99) | 3.15(0.52-5.87) | 2.47(2.23-2.7) |
| East Asia | Male | 9.46(4.08-15.37) | 55.03(10.72-103.51) | 2.01(0.9-3.22) | 5.42(1.1-10.07) | 4.37(3.98-4.75) |
| Eastern Europe | Both | 83.57(29.11-151.16) | 74.8(23.82-115.8) | 29.6(10.31-53.67) | 22.29(7.09-34.49) | -1.55(-1.89--1.21) |
| Eastern Europe | Female | 47.56(16.58-86.53) | 38.71(12.6-60.96) | 27.43(9.51-50.08) | 19.1(6.18-30.08) | -1.84(-2.16--1.53) |
| Eastern Europe | Male | 36(12.64-64.08) | 36.09(10.6-56.94) | 34.07(12.04-59.81) | 27.3(8.17-43.13) | -1.31(-1.67--0.94) |
| Eastern Sub-Saharan Africa | Both | 2.15(0.32-4.01) | 5.9(0.84-10.87) | 2.52(0.39-4.66) | 3.1(0.46-5.73) | 0.83(0.72-0.95) |
| Eastern Sub-Saharan Africa | Female | 1.06(0.15-2.13) | 2.93(0.4-5.39) | 2.41(0.35-4.79) | 2.95(0.42-5.4) | 0.82(0.68-0.96) |
| Eastern Sub-Saharan Africa | Male | 1.09(0.16-2.03) | 2.97(0.43-5.71) | 2.63(0.4-4.9) | 3.27(0.5-6.3) | 0.87(0.77-0.96) |
| High-income Asia Pacific | Both | 37.96(10.58-58.29) | 60.39(16.64-94.04) | 18.38(5.07-28.31) | 15.67(4.48-24.16) | -0.65(-0.92--0.38) |
| High-income Asia Pacific | Female | 16.91(4.74-26.1) | 24.84(7.33-39.16) | 15.05(4.24-23.21) | 12.09(3.72-18.7) | -0.88(-1.12--0.64) |
| High-income Asia Pacific | Male | 21.05(6.07-32.87) | 35.55(9.42-55.24) | 22.53(6.27-35.38) | 19.6(5.33-30.49) | -0.57(-0.86--0.27) |
| High-income North America | Both | 93.26(31.53-142.24) | 138.13(52.09-221.23) | 27.42(9.35-41.97) | 23.89(8.88-38.68) | -0.63(-0.71--0.56) |
| High-income North America | Female | 44.08(15.02-67.27) | 61.12(22.81-98.78) | 23.08(7.73-35.45) | 19.85(7.57-32.43) | -0.66(-0.75--0.57) |
| High-income North America | Male | 49.18(17.31-75.44) | 77.01(28.6-121.6) | 32.97(11.49-50.59) | 28.39(10.62-45.14) | -0.69(-0.78--0.61) |
| North Africa and Middle East | Both | 3.35(0.84-6.09) | 11.27(2.35-20.23) | 1.75(0.46-3.15) | 2.35(0.52-4.19) | 1.27(1.02-1.53) |
| North Africa and Middle East | Female | 1.67(0.38-3.13) | 5.28(1.03-9.68) | 1.77(0.43-3.29) | 2.27(0.47-4.14) | 1.12(0.89-1.35) |
| North Africa and Middle East | Male | 1.69(0.44-3.16) | 5.99(1.32-10.81) | 1.73(0.48-3.19) | 2.43(0.57-4.35) | 1.43(1.15-1.71) |
| Oceania | Both | 0.05(0.02-0.09) | 0.14(0.04-0.26) | 1.47(0.49-2.59) | 1.71(0.51-3.1) | 0.5(0.42-0.57) |
| Oceania | Female | 0.02(0.01-0.04) | 0.06(0.02-0.12) | 1.43(0.42-2.63) | 1.65(0.45-3.11) | 0.48(0.4-0.56) |
| Oceania | Male | 0.03(0.01-0.05) | 0.07(0.02-0.14) | 1.51(0.5-2.7) | 1.77(0.56-3.28) | 0.52(0.44-0.59) |
| South Asia | Both | 9.71(5.16-14.38) | 37.58(22.25-54.35) | 1.56(0.81-2.33) | 2.53(1.5-3.66) | 1.68(1.59-1.76) |
| South Asia | Female | 4.55(2.5-6.74) | 18.33(10.65-26.59) | 1.51(0.81-2.22) | 2.43(1.43-3.56) | 1.63(1.5-1.76) |
| South Asia | Male | 5.16(2.65-7.89) | 19.25(10.97-28.26) | 1.62(0.82-2.48) | 2.63(1.53-3.85) | 1.74(1.67-1.81) |
| Southeast Asia | Both | 3.98(2.02-5.77) | 16.23(6.82-25.69) | 1.38(0.73-1.98) | 2.48(1.06-3.9) | 2.04(1.91-2.18) |
| Southeast Asia | Female | 1.94(0.94-2.88) | 7.23(2.73-11.93) | 1.29(0.64-1.89) | 2.1(0.81-3.45) | 1.75(1.62-1.88) |
| Southeast Asia | Male | 2.04(1.07-3) | 9(3.92-14.27) | 1.49(0.81-2.16) | 2.9(1.3-4.55) | 2.31(2.17-2.44) |
| Southern Latin America | Both | 6.55(1.13-10.58) | 15.73(3.77-24.76) | 14.12(2.43-22.83) | 19.33(4.68-30.41) | 1.2(1.11-1.29) |
| Southern Latin America | Female | 3.21(0.59-5.18) | 7.56(1.97-11.83) | 12.69(2.33-20.45) | 16.92(4.45-26.38) | 1.1(1-1.19) |
| Southern Latin America | Male | 3.34(0.57-5.51) | 8.17(1.84-13.1) | 15.84(2.7-26.12) | 22.22(5-35.6) | 1.3(1.22-1.38) |
| Southern Sub-Saharan Africa | Both | 0.87(0.15-1.56) | 2.26(0.34-4.03) | 2.9(0.5-5.29) | 3.73(0.58-6.62) | 1.16(0.97-1.35) |
| Southern Sub-Saharan Africa | Female | 0.44(0.07-0.79) | 1.1(0.16-2) | 2.66(0.43-4.82) | 3.26(0.49-5.89) | 1.17(1-1.35) |
| Southern Sub-Saharan Africa | Male | 0.43(0.08-0.81) | 1.16(0.18-2.1) | 3.17(0.58-6.1) | 4.35(0.69-7.83) | 1.22(0.94-1.5) |
| Tropical Latin America | Both | 3.07(0.49-5.35) | 13(1.67-22.39) | 3.07(0.52-5.33) | 5.22(0.67-8.98) | 2.24(2.11-2.38) |
| Tropical Latin America | Female | 1.67(0.26-2.96) | 6.76(0.87-11.75) | 3.18(0.5-5.67) | 5.06(0.65-8.79) | 2.02(1.88-2.16) |
| Tropical Latin America | Male | 1.4(0.24-2.47) | 6.24(0.82-10.92) | 2.94(0.54-5.18) | 5.41(0.72-9.45) | 2.51(2.38-2.65) |
| Western Europe | Both | 155.9(53.82-237.24) | 186.05(63.97-283.6) | 27.96(9.56-42.57) | 22.09(7.46-33.78) | -0.98(-1.15--0.82) |
| Western Europe | Female | 77.06(26.19-118.14) | 82.82(28.13-127.49) | 24.08(8.04-37.07) | 17.95(6.27-27.75) | -1.19(-1.34--1.05) |
| Western Europe | Male | 78.84(24.62-119.22) | 103.23(35.4-156.68) | 33.06(10.33-49.92) | 26.81(9.27-40.88) | -0.88(-1.06--0.71) |
| Western Sub-Saharan Africa | Both | 3.23(0.43-5.94) | 9.96(1.52-17.45) | 3.43(0.47-6.31) | 4.8(0.72-8.44) | 1.41(1.31-1.52) |
| Western Sub-Saharan Africa | Female | 1.54(0.22-2.95) | 5.15(0.84-9.25) | 3.37(0.46-6.46) | 4.79(0.75-8.57) | 1.51(1.39-1.63) |
| Western Sub-Saharan Africa | Male | 1.7(0.23-3.21) | 4.8(0.7-8.6) | 3.47(0.46-6.59) | 4.81(0.69-8.65) | 1.35(1.26-1.45) |

ASDR, age-standardized death rate; DALYs, disability-adjusted life years; SDI, socio-demographic index; UI, uncertainty interval.
