# Supplementary material for: Dietary Risk-Related Colorectal Cancer Burden: Estimates From 1990 to 2019
Source: Front Nutr. 2021 Aug 24;8:690663. doi: 10.3389/fnut.2021.690663 (PMC8421520; doi:10.3389/fnut.2021.690663)
Supplement: Supplementary file 3 [file Data_Sheet_3.zip › Supplemental tables/Table S17.docx]

**Table S17** Deaths, ASDRs and change trends of colorectal cancer attributable to diet low in milk between 1990 and 2019 by SDI, regions and sex

| **Location** | **Sex** | **Deaths (95%UI)** | | **ASDR (95%UI)** | | **EAPC (95%CI)** |
| --- | --- | --- | --- | --- | --- | --- |
|  |  | **2019** | **1990** | **1990** | **2019** | **1990-2019** |
| Global | Both | 72199.11(44384.42-99966.07) | 166456.36(107220.8-226027.21) | 1.98(1.2-2.74) | 2.09(1.34-2.84) | 0.19(0.15-0.23) |
| Global | Female | 35728.96(21868.77-49886.84) | 74359.78(46930.76-99956.58) | 1.76(1.08-2.47) | 1.7(1.07-2.28) | -0.19(-0.23--0.15) |
| Global | Male | 36470.15(22456.83-50699.66) | 92096.57(59298.2-125756.05) | 2.26(1.37-3.16) | 2.56(1.65-3.51) | 0.48(0.43-0.54) |
| **Sociodemographic Index** | | | | | | |
| High SDI | Both | 26999.06(14173.84-39994.89) | 39255.37(21761.65-57567.73) | 2.58(1.36-3.81) | 1.95(1.08-2.85) | -1.14(-1.2--1.09) |
| High SDI | Female | 13582.07(7089.09-20241.78) | 18401.1(10282.25-27076.87) | 2.16(1.13-3.21) | 1.55(0.86-2.28) | -1.33(-1.4--1.26) |
| High SDI | Male | 13416.98(7169.14-19861.77) | 20854.27(11490.85-30775.98) | 3.19(1.69-4.72) | 2.42(1.33-3.57) | -1.11(-1.16--1.07) |
| High-middle SDI | Both | 19832.4(11648.2-28460.69) | 46114.73(28371.19-63888.82) | 1.98(1.16-2.84) | 2.29(1.41-3.17) | 0.55(0.49-0.62) |
| High-middle SDI | Female | 9843.47(5750.26-14170.14) | 19796.42(11902.02-27442.62) | 1.69(0.98-2.44) | 1.73(1.04-2.39) | 0.03(-0.02-0.07) |
| High-middle SDI | Male | 9988.93(5873.69-14321.94) | 26318.31(16417.13-36637.59) | 2.42(1.42-3.48) | 3.05(1.89-4.24) | 0.91(0.82-1) |
| Low SDI | Both | 2606.66(1743.66-3556.71) | 6513.89(4307.82-8719.46) | 1.21(0.81-1.65) | 1.39(0.92-1.86) | 0.46(0.38-0.54) |
| Low SDI | Female | 1176.16(755.97-1709.98) | 3150.08(2073.92-4210.15) | 1.1(0.71-1.6) | 1.31(0.86-1.74) | 0.59(0.5-0.68) |
| Low SDI | Male | 1430.5(921.3-2043.78) | 3363.82(2196.54-4575.31) | 1.32(0.85-1.87) | 1.47(0.95-2) | 0.37(0.3-0.44) |
| Low-middle SDI | Both | 6808.29(4699.82-9068.63) | 22079.93(14880.19-29018.04) | 1.23(0.85-1.65) | 1.72(1.16-2.25) | 1.13(1.08-1.18) |
| Low-middle SDI | Female | 3354.47(2280.93-4573.21) | 10930.54(7230.94-14485.48) | 1.22(0.83-1.67) | 1.63(1.07-2.15) | 0.91(0.83-1) |
| Low-middle SDI | Male | 3453.83(2337.37-4771.97) | 11149.39(7517.61-14868.59) | 1.24(0.83-1.71) | 1.83(1.23-2.44) | 1.36(1.3-1.41) |
| Middle SDI | Both | 15909.57(11068.66-20548.01) | 52393.69(34927.86-69154.88) | 1.68(1.17-2.18) | 2.24(1.49-2.96) | 1.13(0.99-1.26) |
| Middle SDI | Female | 7750.71(5324.89-10148.85) | 22033.54(14357.1-29310.1) | 1.58(1.09-2.07) | 1.79(1.17-2.38) | 0.46(0.37-0.55) |
| Middle SDI | Male | 8158.86(5606.65-10635.3) | 30360.14(20369.04-40332.41) | 1.8(1.24-2.34) | 2.75(1.85-3.65) | 1.69(1.53-1.86) |
| **Region** | | | | | | |
| Africa | Both | 3541.84(2395.85-4746.35) | 9063.74(6011.6-12205.97) | 1.38(0.94-1.85) | 1.61(1.06-2.15) | 0.57(0.52-0.63) |
| Africa | Female | 1679.42(1134.65-2309.54) | 4351.42(2847.86-5880.33) | 1.29(0.87-1.78) | 1.5(0.98-2.02) | 0.59(0.53-0.65) |
| Africa | Male | 1862.42(1216.11-2541.79) | 4712.33(3104.44-6335.73) | 1.46(0.96-1.99) | 1.73(1.14-2.31) | 0.58(0.52-0.63) |
| America | Both | 11610.85(5951.73-17468.26) | 20460.25(10808.89-30701.64) | 1.93(2.9-0.99) | 1.59(0.84-2.39) | -0.74(-0.83--0.65) |
| America | Female | 5962.98(3064.16-8927.37) | 10114.7(5400-15209.44) | 1.73(2.59-0.89) | 1.41(0.75-2.12) | -0.77(-0.84--0.69) |
| America | Male | 5647.86(2920.25-8464.34) | 10345.55(5523.49-15452.3) | 2.2(1.14-3.3) | 1.81(0.97-2.7) | -0.76(-0.87--0.64) |
| Asia | Both | 33946.49(23162.29-44247.67) | 105033.13(69720.82-137593.24) | 1.87(1.27-2.45) | 2.33(1.55-3.06) | 0.85(0.73-0.96) |
| Asia | Female | 16116.39(10986-21265.84) | 44869.37(29349.14-59151.75) | 1.71(1.17-2.25) | 1.87(1.22-2.47) | 0.29(0.22-0.37) |
| Asia | Male | 17830.1(23437.68-12126.88) | 60163.76(40022.08-80343.88) | 2.07(1.4-2.72) | 2.87(1.92-3.83) | 1.3(1.15-1.45) |
| Europe | Both | 22958.96(10993.46-35738.93) | 31573.33(14857.68-49233.51) | 2.24(1.07-3.48) | 1.95(0.92-3.02) | -0.68(-0.76--0.6) |
| Europe | Female | 11902.46(5702.72-18498) | 14870.64(6838.47-22986.22) | 1.87(0.9-2.91) | 1.53(0.71-2.37) | -0.93(-1.03--0.83) |
| Europe | Male | 11056.5(5232.6-17162.55) | 16702.69(7703.4-26030.6) | 2.85(1.34-4.41) | 2.53(1.17-3.94) | -0.58(-0.64--0.51) |
| Andean Latin America | Both | 269.25(177.22-366.57) | 997.55(628.86-1408.38) | 1.4(0.92-1.91) | 1.83(1.16-2.58) | 1.13(0.98-1.29) |
| Andean Latin America | Female | 145.88(96.11-197.42) | 543.29(344.25-757.68) | 1.49(0.98-2) | 1.9(1.2-2.64) | 0.92(0.77-1.07) |
| Andean Latin America | Male | 123.37(80.54-170.45) | 454.25(284-654.87) | 1.31(0.86-1.81) | 1.75(1.1-2.53) | 1.4(1.22-1.57) |
| Australasia | Both | 466.53(166.82-779.77) | 410.05(141.55-805.88) | 2.02(0.74-3.38) | 0.78(0.26-1.53) | -4.1(-4.56--3.63) |
| Australasia | Female | 223.99(84.42-374.39) | 196.18(69.69-386.87) | 1.72(0.66-2.88) | 0.65(0.22-1.31) | -4.06(-4.51--3.6) |
| Australasia | Male | 242.55(88.41-405.15) | 213.87(69.93-423.18) | 2.42(0.89-4.03) | 0.92(0.3-1.82) | -4.17(-4.65--3.69) |
| Caribbean | Both | 579.78(376.07-784.66) | 1430.58(909.55-1992.92) | 2.32(1.51-3.15) | 2.77(1.76-3.85) | 0.63(0.58-0.68) |
| Caribbean | Female | 304.84(196.37-412.39) | 741.14(469.83-1035.47) | 2.34(1.51-3.16) | 2.63(1.66-3.68) | 0.43(0.38-0.48) |
| Caribbean | Male | 274.94(179.75-370.81) | 689.44(439.96-968.85) | 2.29(1.5-3.08) | 2.9(1.85-4.07) | 0.85(0.78-0.92) |
| Central Asia | Both | 480.95(231.9-754.55) | 690.63(340.47-1097.98) | 1.03(0.5-1.62) | 1.04(0.51-1.63) | -0.09(-0.29-0.11) |
| Central Asia | Female | 243.69(114.7-384.56) | 334.42(165.33-531.1) | 0.89(0.42-1.4) | 0.89(0.43-1.4) | -0.14(-0.31-0.03) |
| Central Asia | Male | 237.26(115.33-371.92) | 356.21(177.25-568.06) | 1.24(0.6-1.94) | 1.25(0.63-2.02) | -0.08(-0.32-0.16) |
| Central Europe | Both | 3710.52(1771.33-5619.9) | 6315.11(3246.02-9514.77) | 2.61(1.25-3.94) | 2.88(1.49-4.34) | 0.3(0.17-0.43) |
| Central Europe | Female | 1768.91(841.07-2674.86) | 2741.93(1390.37-4136) | 2.14(1.02-3.23) | 2.11(1.08-3.18) | -0.18(-0.31--0.06) |
| Central Europe | Male | 1941.61(930.26-2929.42) | 3573.18(1838.9-5437.9) | 3.27(1.57-4.94) | 3.98(2.05-6.05) | 0.72(0.57-0.87) |
| Central Latin America | Both | 924.44(570.51-1281.08) | 3462.73(1971.75-5057.46) | 1.19(0.73-1.64) | 1.5(0.85-2.19) | 0.78(0.73-0.83) |
| Central Latin America | Female | 499.17(307.6-690.58) | 1726.89(982.35-2538.69) | 1.25(0.76-1.72) | 1.38(0.78-2.02) | 0.33(0.27-0.4) |
| Central Latin America | Male | 425.27(262.32-592.34) | 1735.83(981.28-2539.49) | 1.12(0.69-1.56) | 1.63(0.92-2.38) | 1.27(1.21-1.33) |
| Central Sub-Saharan Africa | Both | 412.37(299.08-545.54) | 928.67(631.68-1316.42) | 2.07(1.5-2.75) | 1.98(1.33-2.84) | -0.27(-0.53--0.01) |
| Central Sub-Saharan Africa | Female | 185.35(132.35-254.77) | 446.35(295.57-638.79) | 1.76(1.26-2.41) | 1.72(1.12-2.5) | -0.16(-0.39-0.07) |
| Central Sub-Saharan Africa | Male | 227.02(157.38-328.82) | 482.31(317.59-745.86) | 2.42(1.68-3.75) | 2.34(1.55-3.77) | -0.27(-0.54-0.01) |
| East Asia | Both | 15903.22(10805.6-20941.58) | 52876.58(35241.16-71141.72) | 1.98(1.35-2.61) | 2.7(1.8-3.61) | 1.39(1.14-1.64) |
| East Asia | Female | 7485.13(5004.52-10099.12) | 19714.08(12732.66-26649.74) | 1.78(1.19-2.38) | 1.89(1.22-2.55) | 0.34(0.14-0.54) |
| East Asia | Male | 8418.09(5569.09-11335.24) | 33162.49(21391.25-45767.1) | 2.29(1.52-3.05) | 3.76(2.45-5.12) | 2.19(1.91-2.47) |
| Eastern Europe | Both | 3463.95(1195.3-6059.29) | 5911.57(2385.67-9538.95) | 1.28(0.45-2.21) | 1.71(0.7-2.76) | 0.92(0.55-1.29) |
| Eastern Europe | Female | 1962.29(647.31-3444.74) | 3059.26(1190.65-4971.08) | 1.1(0.37-1.93) | 1.38(0.55-2.25) | 0.72(0.33-1.12) |
| Eastern Europe | Male | 1501.66(527.59-2610.98) | 2852.31(1167.01-4680.87) | 1.67(0.58-2.89) | 2.29(0.94-3.75) | 0.94(0.61-1.27) |
| Eastern Sub-Saharan Africa | Both | 893.57(571.42-1252.99) | 2265.21(1440.05-3134.64) | 1.29(0.83-1.79) | 1.52(0.97-2.09) | 0.58(0.51-0.66) |
| Eastern Sub-Saharan Africa | Female | 406.76(245.22-607.09) | 1081.67(677.43-1531.72) | 1.15(0.71-1.7) | 1.37(0.86-1.92) | 0.65(0.56-0.74) |
| Eastern Sub-Saharan Africa | Male | 486.81(305.18-727.66) | 1183.55(743.92-1703.03) | 1.44(0.91-2.08) | 1.69(1.06-2.42) | 0.57(0.51-0.63) |
| High-income Asia Pacific | Both | 5862.43(3679.66-8025.34) | 13123.56(8062.43-18132.69) | 3.06(1.92-4.17) | 2.61(1.58-3.59) | -0.65(-0.71--0.59) |
| High-income Asia Pacific | Female | 2742.09(1721.68-3748.45) | 6263.33(3805.92-8767.3) | 2.47(1.55-3.38) | 2(1.23-2.77) | -0.85(-0.9--0.8) |
| High-income Asia Pacific | Male | 3120.34(1965.72-4291.76) | 6860.23(4234.59-9503.66) | 3.9(2.44-5.37) | 3.34(2.07-4.6) | -0.65(-0.74--0.56) |
| High-income North America | Both | 7357.52(3206.64-11459.64) | 8476.12(3296.58-13802.11) | 2.05(0.9-3.2) | 1.32(0.51-2.16) | -1.72(-1.92--1.52) |
| High-income North America | Female | 3762.67(1647.99-5908.32) | 4096.11(1634.59-6732.23) | 1.75(0.77-2.73) | 1.12(0.44-1.83) | -1.69(-1.87--1.52) |
| High-income North America | Male | 3594.85(1570.91-5637.98) | 4380(1690.42-7181.86) | 2.49(1.09-3.9) | 1.56(0.6-2.55) | -1.85(-2.08--1.62) |
| North Africa and Middle East | Both | 2005.68(1240.03-2903.75) | 6185.43(3789.91-8725.26) | 1.27(0.78-1.84) | 1.55(0.94-2.19) | 0.81(0.66-0.95) |
| North Africa and Middle East | Female | 966.09(595.52-1395.93) | 2820.75(1710.37-3958.22) | 1.23(0.76-1.79) | 1.45(0.88-2.04) | 0.67(0.53-0.82) |
| North Africa and Middle East | Male | 1039.6(626.27-1539.53) | 3364.68(2036.7-4752.18) | 1.3(0.78-1.93) | 1.65(1-2.33) | 0.93(0.78-1.08) |
| Oceania | Both | 38.64(24.97-54.05) | 104.04(66.25-144.59) | 1.47(0.95-2.05) | 1.68(1.09-2.3) | 0.43(0.36-0.5) |
| Oceania | Female | 17.59(11.11-25.58) | 47.02(30.54-67.02) | 1.38(0.88-1.99) | 1.56(1.02-2.21) | 0.4(0.32-0.48) |
| Oceania | Male | 21.05(13.15-29.91) | 57.02(36.06-79.83) | 1.56(0.99-2.19) | 1.8(1.16-2.48) | 0.46(0.4-0.53) |
| South Asia | Both | 5419.37(3766.42-7189.81) | 19002.34(12875.49-24980.8) | 1.07(0.74-1.42) | 1.47(1-1.93) | 0.94(0.8-1.07) |
| South Asia | Female | 2597.26(1759.82-3585.11) | 9876.64(6508.89-13220.32) | 1.07(0.73-1.48) | 1.5(0.99-1.99) | 0.95(0.78-1.12) |
| South Asia | Male | 2822.11(1916.21-3883.88) | 9125.7(5992.49-12478.29) | 1.07(0.72-1.47) | 1.45(0.95-1.96) | 0.91(0.8-1.02) |
| Southeast Asia | Both | 5071.12(3678.93-6465.74) | 16263.01(11213.55-21732.36) | 2.12(1.54-2.7) | 2.86(1.97-3.82) | 0.91(0.85-0.97) |
| Southeast Asia | Female | 2478.1(1760.31-3198.66) | 7274.02(4664-9962.62) | 1.94(1.39-2.49) | 2.36(1.51-3.22) | 0.55(0.49-0.61) |
| Southeast Asia | Male | 2593.02(1872.04-3340.07) | 8988.99(6240.26-12106.57) | 2.33(1.69-2.98) | 3.45(2.39-4.67) | 1.25(1.18-1.31) |
| Southern Latin America | Both | 1316.71(748.01-1881.24) | 2704.79(1541.54-3832.54) | 2.98(1.68-4.26) | 3.2(1.83-4.54) | 0.26(0.14-0.38) |
| Southern Latin America | Female | 632.18(357.81-898.93) | 1301.23(750.67-1864.42) | 2.52(1.43-3.58) | 2.64(1.53-3.77) | 0.14(0.01-0.26) |
| Southern Latin America | Male | 684.53(386.76-976.24) | 1403.57(793.18-1992.77) | 3.55(2.02-5.08) | 3.93(2.22-5.58) | 0.38(0.25-0.51) |
| Southern Sub-Saharan Africa | Both | 451.95(282.94-641.14) | 1016.79(636.55-1423.24) | 1.8(1.12-2.57) | 1.98(1.23-2.76) | 0.32(0.1-0.54) |
| Southern Sub-Saharan Africa | Female | 233.78(144.93-340.9) | 511.91(310.02-718.65) | 1.62(1-2.4) | 1.69(1.03-2.37) | 0.23(0.08-0.37) |
| Southern Sub-Saharan Africa | Male | 218.18(133.84-316.33) | 504.88(310.55-718.67) | 2(1.21-2.94) | 2.4(1.48-3.41) | 0.53(0.22-0.84) |
| Tropical Latin America | Both | 1278.54(704.8-1845.37) | 3658.69(1821.6-5417.37) | 1.53(0.84-2.22) | 1.54(0.77-2.28) | -0.01(-0.17-0.14) |
| Tropical Latin America | Female | 673.61(368.08-968.24) | 1834.94(920.97-2723.51) | 1.51(0.82-2.17) | 1.38(0.69-2.05) | -0.36(-0.53--0.2) |
| Tropical Latin America | Male | 604.93(337.16-867.76) | 1823.75(920.89-2712.93) | 1.55(0.85-2.23) | 1.74(0.87-2.6) | 0.4(0.25-0.54) |
| Western Europe | Both | 15267.68(7360.85-23212.72) | 17955.52(8091.08-28062.62) | 2.6(1.25-3.95) | 1.8(0.81-2.84) | -1.57(-1.71--1.44) |
| Western Europe | Female | 7926.97(3811.33-12029.86) | 8450.1(3803.29-13144.73) | 2.2(1.06-3.34) | 1.43(0.65-2.23) | -1.82(-1.98--1.67) |
| Western Europe | Male | 7340.71(3544.23-11090.82) | 9505.42(4229.13-14949.26) | 3.2(1.55-4.84) | 2.27(1.02-3.58) | -1.46(-1.57--1.34) |
| Western Sub-Saharan Africa | Both | 1024.88(680.87-1431.33) | 2677.38(1794.24-3618.63) | 1.31(0.88-1.83) | 1.65(1.1-2.21) | 1.02(0.9-1.14) |
| Western Sub-Saharan Africa | Female | 472.63(302.62-682.33) | 1298.5(850.7-1802.68) | 1.21(0.79-1.74) | 1.55(1.01-2.11) | 1.12(0.98-1.26) |
| Western Sub-Saharan Africa | Male | 552.25(350.66-786.55) | 1378.89(909.82-1899.17) | 1.41(0.9-1.97) | 1.75(1.16-2.36) | 0.98(0.86-1.09) |

ASDR, age-standardized death rate, SDI, socio-demographic index; UI, uncertainty interval.
