# Supplementary material for: Dietary Risk-Related Colorectal Cancer Burden: Estimates From 1990 to 2019
Source: Front Nutr. 2021 Aug 24;8:690663. doi: 10.3389/fnut.2021.690663 (PMC8421520; doi:10.3389/fnut.2021.690663)
Supplement: Supplementary file 3 [file Data_Sheet_3.zip › Supplemental tables/Table S18.docx]

**Table S18** DALYs, ASRs and change trends of colorectal cancer DALYs attributable to diet low in milk between 1990 and 2019 by SDI, regions and sex.

| **Location** | **Sex** | **DALYs (No.×1000, 95%UI)** | | **ASR (95%UI)** | | **EAPC (95%CI)** |
| --- | --- | --- | --- | --- | --- | --- |
|  |  | **1990** | **2019** | **1990** | **2019** | **1990-2019** |
| Global | Both | 1764.17(1115.03-2420.49) | 3799.3(2457.77-5124.45) | 43.71(27.48-60.09) | 46.09(29.83-62.18) | 0.19(0.15-0.22) |
| Global | Female | 833.68(527.32-1156.77) | 1597.63(1013.55-2129.09) | 38.9(24.49-54.09) | 36.8(23.35-49.03) | -0.27(-0.31--0.23) |
| Global | Male | 930.49(583.53-1290.97) | 2201.66(1426.2-3006.6) | 49.5(30.61-68.76) | 56.46(36.57-77.15) | 0.53(0.46-0.59) |
| **Sociodemographic Index** | | | | | | |
| High SDI | Both | 574.93(306.46-847.76) | 734.09(405.53-1078.05) | 56.29(30.2-82.89) | 41.4(22.87-61.09) | -1.25(-1.3--1.19) |
| High SDI | Female | 268.11(141.65-397.6) | 311.28(170.72-459.53) | 46.44(24.67-68.63) | 32.3(17.71-47.75) | -1.45(-1.53--1.38) |
| High SDI | Male | 306.81(165.93-449.98) | 422.8(234.85-625.16) | 68.99(37.27-101.33) | 51.58(28.78-76.16) | -1.18(-1.23--1.13) |
| High-middle SDI | Both | 484.54(285.91-693.75) | 1027.48(642.66-1415.78) | 44.76(26.41-64.08) | 51(31.94-70.25) | 0.5(0.43-0.57) |
| High-middle SDI | Female | 229.6(134.86-331.97) | 410.79(252.07-565.47) | 38.41(22.59-55.39) | 37.66(23.17-51.72) | -0.14(-0.18--0.09) |
| High-middle SDI | Male | 254.93(150.44-364.59) | 616.68(389.61-860.29) | 53.42(31.43-76.53) | 66.8(42.13-92.97) | 0.9(0.8-1) |
| Low SDI | Both | 72.42(48.39-98.93) | 173.99(115.15-233.93) | 28.34(18.89-38.67) | 31.22(20.64-41.86) | 0.31(0.22-0.4) |
| Low SDI | Female | 33.36(21.08-48.48) | 83.88(55.25-112.65) | 26.14(16.76-37.99) | 29.48(19.42-39.49) | 0.4(0.29-0.5) |
| Low SDI | Male | 39.06(25.26-56.27) | 90.11(58.58-123) | 30.44(19.63-43.6) | 33.03(21.52-45.12) | 0.26(0.19-0.33) |
| Low-middle SDI | Both | 190.76(130.93-255.21) | 561.04(376.26-741.26) | 29.35(20.18-39.2) | 39.43(26.46-51.97) | 1(0.95-1.04) |
| Low-middle SDI | Female | 94.2(63.52-128.87) | 272.66(180.66-363.31) | 29.26(19.76-40.12) | 37.1(24.5-49.41) | 0.72(0.62-0.82) |
| Low-middle SDI | Male | 96.56(65.11-133.09) | 288.38(195.15-385.35) | 29.44(19.81-40.69) | 41.94(28.32-56.05) | 1.27(1.21-1.32) |
| Middle SDI | Both | 440.52(306.41-572.16) | 1300.48(868.15-1713.81) | 39.54(27.45-51.13) | 50.78(34-66.86) | 1.02(0.9-1.15) |
| Middle SDI | Female | 207.91(141.08-273.93) | 517.99(341.54-690.71) | 36.97(25.22-48.62) | 39.27(25.86-52.36) | 0.23(0.17-0.3) |
| Middle SDI | Male | 232.61(158.99-304.69) | 782.49(525.74-1044.66) | 42.27(29.01-55.13) | 63.17(42.21-84.25) | 1.65(1.47-1.82) |
| **Region** | | | | | | |
| Africa | Both | 94.74(64.09-127.94) | 238.68(157.37-323.52) | 31.18(21.12-41.89) | 35.42(23.47-47.81) | 0.48(0.42-0.54) |
| Africa | Female | 44.12(29.71-61.61) | 111.9(72.85-152.77) | 28.81(19.46-39.94) | 32.33(21.13-43.82) | 0.48(0.41-0.56) |
| Africa | Male | 50.62(32.8-69.77) | 126.79(82.93-171.29) | 33.52(21.79-45.92) | 38.68(25.53-52.16) | 0.5(0.45-0.54) |
| America | Both | 256.9(135.22-384.31) | 447.9(242.96-671.24) | 42.03(22.11-62.92) | 35.9(19.52-53.7) | -0.6(-0.69--0.51) |
| America | Female | 124.83(65.17-187.33) | 208.78(111.37-314.83) | 37.2(19.49-55.73) | 31.12(16.68-46.77) | -0.65(-0.73--0.58) |
| America | Male | 132.08(68.87-196.82) | 239.12(128.66-356.19) | 47.86(24.95-71.41) | 41.31(22.25-61.55) | -0.59(-0.69--0.48) |
| Asia | Both | 917.21(630.2-1200.65) | 2499.77(1676.41-3272.47) | 42.75(29.25-55.89) | 51.37(34.33-67.42) | 0.72(0.62-0.82) |
| Asia | Female | 424.94(290.81-564.42) | 1009.39(662.46-1332.49) | 39.21(26.81-51.9) | 40.31(26.4-53.18) | 0.04(-0.01-0.09) |
| Asia | Male | 492.26(333.99-649.93) | 1490.38(999.48-2001.32) | 46.65(31.67-61.3) | 63.29(42.42-84.58) | 1.25(1.09-1.4) |
| Europe | Both | 492.1(236.35-767.82) | 606.19(284.05-949.08) | 48.17(23.12-75.1) | 41.24(19.48-64.39) | -0.76(-0.86--0.66) |
| Europe | Female | 238.32(114.02-371.6) | 264.6(124.43-411.58) | 39.91(19.12-62.33) | 31.98(15.1-49.96) | -1(-1.12--0.87) |
| Europe | Male | 253.78(120.54-395.82) | 341.59(159.52-537.01) | 60.29(28.62-93.79) | 52.9(24.81-83) | -0.66(-0.74--0.58) |
| Andean Latin America | Both | 6.57(4.32-9.05) | 21.94(13.75-31.01) | 30.67(20.27-41.98) | 38.7(24.22-54.61) | 1(0.85-1.16) |
| Andean Latin America | Female | 3.45(2.26-4.68) | 11.43(7.15-16.16) | 31.57(20.7-42.53) | 38.93(24.28-55.04) | 0.79(0.64-0.95) |
| Andean Latin America | Male | 3.12(2.03-4.29) | 10.51(6.49-15.24) | 29.68(19.3-40.86) | 38.35(23.68-55.57) | 1.25(1.07-1.42) |
| Australasia | Both | 10.6(3.76-17.62) | 7.42(2.23-14.87) | 46.09(16.5-76.43) | 15.82(4.63-31.74) | -4.58(-5.11--4.06) |
| Australasia | Female | 4.83(1.76-8.04) | 3.3(1.1-6.64) | 39.22(14.28-65.14) | 13.11(4.27-26.52) | -4.6(-5.12--4.08) |
| Australasia | Male | 5.78(2.09-9.6) | 4.12(1.21-8.41) | 54.21(19.91-90.34) | 18.82(5.42-38.14) | -4.6(-5.13--4.06) |
| Caribbean | Both | 13.27(8.62-17.84) | 30.71(19.48-43.04) | 50.31(32.67-67.74) | 59.44(37.71-83.28) | 0.61(0.56-0.67) |
| Caribbean | Female | 6.81(4.35-9.23) | 15.13(9.57-21.14) | 49.99(31.96-67.57) | 55.28(34.95-77.23) | 0.37(0.33-0.42) |
| Caribbean | Male | 6.46(4.23-8.69) | 15.58(9.97-21.95) | 50.56(33.14-67.99) | 63.89(40.77-89.98) | 0.86(0.78-0.93) |
| Central Asia | Both | 13.95(6.76-21.82) | 18.78(9.25-29.84) | 27.71(13.41-43.53) | 23.81(11.77-37.7) | -0.85(-1.12--0.59) |
| Central Asia | Female | 6.61(3.14-10.37) | 8.53(4.14-13.5) | 23.52(11.16-36.97) | 19.81(9.58-31.48) | -0.95(-1.18--0.71) |
| Central Asia | Male | 7.34(3.62-11.53) | 10.25(5.06-16.12) | 33.29(16.21-52.29) | 28.91(14.44-45.88) | -0.78(-1.08--0.49) |
| Central Europe | Both | 85.9(41.31-130.02) | 127.69(66.24-192.34) | 58.38(28.14-88.38) | 62.04(32.35-93.38) | 0.15(0.04-0.27) |
| Central Europe | Female | 38.73(18.52-58.68) | 51.25(26.42-77.88) | 47.1(22.55-71.27) | 44.47(22.73-67.9) | -0.32(-0.42--0.22) |
| Central Europe | Male | 47.17(22.85-71.45) | 76.44(38.77-116.76) | 73.08(35.33-110.4) | 84.25(42.92-128.52) | 0.5(0.36-0.63) |
| Central Latin America | Both | 23.32(14.42-32.35) | 82.46(47.13-121.32) | 26.15(16.18-36.33) | 34.22(19.49-50.25) | 0.92(0.87-0.97) |
| Central Latin America | Female | 12.18(7.49-16.81) | 39.3(22.29-57.54) | 26.67(16.44-36.88) | 30.51(17.31-44.66) | 0.48(0.41-0.55) |
| Central Latin America | Male | 11.14(6.91-15.46) | 43.15(24.19-63.21) | 25.55(15.85-35.56) | 38.41(21.54-56.22) | 1.38(1.32-1.44) |
| Central Sub-Saharan Africa | Both | 11.59(8.32-15.45) | 25.93(17.55-36.94) | 47.27(34.16-62.7) | 44.4(30.15-63.01) | -0.31(-0.57--0.05) |
| Central Sub-Saharan Africa | Female | 5.27(3.59-7.27) | 12.04(8.02-17.18) | 40.28(28.74-55.17) | 38.44(25.45-55.19) | -0.2(-0.43-0.02) |
| Central Sub-Saharan Africa | Male | 6.32(4.34-9.07) | 13.89(9.14-21.5) | 55.18(38.06-80.7) | 51.96(34.31-80.32) | -0.35(-0.63--0.07) |
| East Asia | Both | 442.32(299.37-585.15) | 1281.31(851.75-1717.8) | 46.96(31.88-61.93) | 61.6(41.17-82.37) | 1.28(1.03-1.52) |
| East Asia | Female | 202.04(134.51-275.93) | 447.95(291.27-605.37) | 42.58(28.53-57.79) | 41.78(27.1-56.45) | 0.02(-0.14-0.19) |
| East Asia | Male | 240.28(159.23-325.9) | 833.36(541.92-1160.86) | 52.25(34.63-70.31) | 83.54(54.29-116.39) | 2.13(1.83-2.44) |
| Eastern Europe | Both | 86.07(29.51-150.61) | 133.28(54.09-216.68) | 30.88(10.73-53.98) | 39.85(16.27-64.62) | 0.72(0.3-1.13) |
| Eastern Europe | Female | 45.95(15.33-80.23) | 64.5(25.18-105.37) | 26.77(9.06-46.66) | 32.01(12.56-52.01) | 0.49(0.06-0.93) |
| Eastern Europe | Male | 40.13(13.6-69.28) | 68.78(27.73-113.09) | 38.66(13.66-66.86) | 52.12(21.04-85.69) | 0.84(0.45-1.23) |
| Eastern Sub-Saharan Africa | Both | 25.11(16-35.67) | 62.47(39.25-87.12) | 30.55(19.51-43.06) | 34.58(21.88-47.82) | 0.43(0.35-0.51) |
| Eastern Sub-Saharan Africa | Female | 11.69(7.01-17.69) | 29.49(18.35-42.09) | 27.6(16.52-41.17) | 31.25(19.49-44.39) | 0.44(0.33-0.54) |
| Eastern Sub-Saharan Africa | Male | 13.42(8.43-20.43) | 32.98(20.5-47.39) | 33.53(21.04-50.16) | 38.26(23.93-54.89) | 0.46(0.4-0.52) |
| High-income Asia Pacific | Both | 137.63(86.81-188.16) | 226.51(140.68-311.17) | 67.87(42.77-92.93) | 55.14(34.43-75.73) | -0.85(-0.93--0.78) |
| High-income Asia Pacific | Female | 60.66(38-83.01) | 94.58(57.82-130.65) | 54.3(34.03-74.26) | 41.09(25.46-56.28) | -1.12(-1.17--1.07) |
| High-income Asia Pacific | Male | 76.97(48.8-105.3) | 131.93(81.57-182.15) | 85.47(54.05-117.28) | 70.88(44.3-97.79) | -0.77(-0.88--0.67) |
| High-income North America | Both | 154.2(66.69-242.68) | 175.71(66.26-288.99) | 45.17(19.67-70.86) | 30.23(11.48-49.67) | -1.56(-1.77--1.35) |
| High-income North America | Female | 73.42(31.82-116.59) | 78.38(30.15-127.73) | 37.92(16.37-60) | 25.08(9.6-40.87) | -1.57(-1.76--1.38) |
| High-income North America | Male | 80.78(35.15-125.94) | 97.34(37.71-158.97) | 54.43(23.86-84.87) | 35.98(13.87-58.83) | -1.63(-1.86--1.41) |
| North Africa and Middle East | Both | 54.99(34.08-79.59) | 160.21(97.5-226) | 29.57(18.3-42.95) | 34.47(20.93-48.64) | 0.59(0.45-0.73) |
| North Africa and Middle East | Female | 26.19(16.11-37.98) | 71.61(43.35-101.14) | 28.6(17.67-41.16) | 31.67(19.21-44.63) | 0.42(0.3-0.53) |
| North Africa and Middle East | Male | 28.8(17.55-42.88) | 88.61(53.94-125.19) | 30.48(18.44-45.25) | 37.17(22.51-52.61) | 0.75(0.59-0.91) |
| Oceania | Both | 1.13(0.74-1.59) | 3.02(1.92-4.27) | 34.17(22.04-47.92) | 38.3(24.37-53.33) | 0.37(0.31-0.43) |
| Oceania | Female | 0.5(0.32-0.73) | 1.33(0.86-1.92) | 31.77(20.1-45.83) | 35.23(22.86-50.41) | 0.34(0.27-0.41) |
| Oceania | Male | 0.63(0.39-0.9) | 1.69(1.05-2.41) | 36.48(22.79-51.92) | 41.28(26.04-57.77) | 0.4(0.35-0.45) |
| South Asia | Both | 151.92(105.63-202.15) | 474.52(320.19-626.88) | 24.77(17.15-32.94) | 32.69(22.04-43.1) | 0.79(0.66-0.93) |
| South Asia | Female | 74.16(50.09-101.3) | 244.5(159.15-329.99) | 25.09(16.96-34.58) | 33.3(21.84-44.78) | 0.76(0.57-0.95) |
| South Asia | Male | 77.76(52.77-106.76) | 230.02(152.49-313.07) | 24.5(16.69-33.73) | 32.06(21.18-43.77) | 0.81(0.71-0.92) |
| Southeast Asia | Both | 141.56(102.79-181.68) | 421(287.71-562.04) | 50.47(36.61-64.3) | 65.65(45.15-87.69) | 0.78(0.72-0.84) |
| Southeast Asia | Female | 67.47(47.02-88.51) | 178.67(115-245.84) | 45.85(32.17-59.56) | 52.95(34.07-72.61) | 0.37(0.31-0.43) |
| Southeast Asia | Male | 74.09(53.45-95.53) | 242.33(168.65-323.92) | 55.66(40.39-71.67) | 80.14(55.66-107.4) | 1.13(1.06-1.2) |
| Southern Latin America | Both | 29.11(16.6-41.79) | 55.46(31.96-78.2) | 62.97(35.94-90.38) | 67.66(38.95-95.06) | 0.28(0.18-0.38) |
| Southern Latin America | Female | 13.23(7.55-18.83) | 24.9(14.33-35.34) | 52.12(29.83-74.14) | 54.77(31.56-77.45) | 0.2(0.11-0.29) |
| Southern Latin America | Male | 15.88(9.01-22.75) | 30.56(17.49-43.29) | 76.19(43.33-109.26) | 83.33(47.71-117.96) | 0.35(0.24-0.46) |
| Southern Sub-Saharan Africa | Both | 11.4(7.22-15.9) | 25.25(15.88-35.47) | 39.13(24.76-55.14) | 42.89(27.02-59.99) | 0.37(0.15-0.59) |
| Southern Sub-Saharan Africa | Female | 5.47(3.45-7.65) | 11.77(7.1-16.6) | 33.93(21.34-48.25) | 35.46(21.29-49.86) | 0.4(0.26-0.54) |
| Southern Sub-Saharan Africa | Male | 5.94(3.71-8.47) | 13.48(8.43-19.17) | 45.36(28.04-65.53) | 52.83(32.64-75.06) | 0.43(0.1-0.76) |
| Tropical Latin America | Both | 33.04(18.38-47.52) | 87.06(43.45-129.38) | 34.12(19-48.94) | 35.3(17.68-52.36) | 0.09(-0.08-0.26) |
| Tropical Latin America | Female | 16.93(9.36-24.28) | 42.05(21.04-62.46) | 33.24(18.3-47.77) | 31.51(15.77-46.76) | -0.22(-0.39--0.04) |
| Tropical Latin America | Male | 16.11(9-23.07) | 45.01(22.71-66.26) | 35.04(19.5-50.24) | 39.82(20.13-58.73) | 0.43(0.25-0.6) |
| Western Europe | Both | 304.74(147.81-461.61) | 311.33(139.74-493.49) | 54.41(26.35-82.33) | 36.45(16.41-57.97) | -1.7(-1.81--1.58) |
| Western Europe | Female | 146.77(71.4-221.63) | 134.95(61.63-211.48) | 45.5(22.16-68.55) | 28.71(13.2-44.93) | -1.91(-2.04--1.78) |
| Western Europe | Male | 157.97(76.1-239.75) | 176.38(78.83-279.88) | 66.35(31.97-100.92) | 45.48(20.38-72.2) | -1.61(-1.72--1.5) |
| Western Sub-Saharan Africa | Both | 25.75(16.85-35.96) | 67.24(44.37-91.86) | 28.4(18.72-39.7) | 34.27(22.93-46.35) | 0.86(0.76-0.97) |
| Western Sub-Saharan Africa | Female | 11.34(7.2-16.48) | 31.97(20.95-44.98) | 25.71(16.39-37.25) | 31.49(20.61-43.87) | 0.95(0.81-1.09) |
| Western Sub-Saharan Africa | Male | 14.41(9.12-20.76) | 35.27(22.98-49.12) | 30.87(19.6-43.99) | 37.28(24.46-51.49) | 0.85(0.75-0.95) |

ASDR, age-standardized death rate; DALYs, disability-adjusted life years; SDI, socio-demographic index; UI, uncertainty interval.
