# Supplementary material for: Dietary Risk-Related Colorectal Cancer Burden: Estimates From 1990 to 2019
Source: Front Nutr. 2021 Aug 24;8:690663. doi: 10.3389/fnut.2021.690663 (PMC8421520; doi:10.3389/fnut.2021.690663)
Supplement: Supplementary file 3 [file Data_Sheet_3.zip › Supplemental tables/Table S19.docx]

**Table S19** Deaths, ASDRs and change trends of colorectal cancer attributable to diet low in calcium between 1990 and 2019 by SDI, regions and sex

| **Location** | **Sex** | **Deaths (95%UI)** | | **ASDR (95%UI)** | | **EAPC (95%CI)** |
| --- | --- | --- | --- | --- | --- | --- |
|  |  | **1990** | **2019** | **1990** | **2019** | **1990-2019** |
| Global | Both | 63708.69(46056.78-87779.23) | 137895.94(96834.76-189221.04) | 1.73(1.24-2.41) | 1.73(1.21-2.38) | 0.04(-0.06-0.13) |
| Global | Female | 29535.12(20735.9-41356.1) | 57916.83(40226.64-80456.02) | 1.45(1.01-2.04) | 1.32(0.92-1.84) | -0.35(-0.42--0.28) |
| Global | Male | 34173.57(24919.32-46574.32) | 79979.11(56679.84-108752.08) | 2.12(1.53-2.91) | 2.22(1.57-3.04) | 0.27(0.15-0.39) |
| **Sociodemographic Index** | | | | | | |
| High SDI | Both | 16353.05(9361.99-26403.7) | 24723.22(14298.41-38860.76) | 1.56(0.89-2.51) | 1.2(0.69-1.88) | -0.85(-0.89--0.81) |
| High SDI | Female | 7398.57(4044.06-12353.99) | 10835.7(6196.85-16974.37) | 1.15(0.63-1.91) | 0.87(0.49-1.36) | -0.92(-0.96--0.89) |
| High SDI | Male | 8954.49(5228.2-14122.84) | 13887.52(8160.92-21673.03) | 2.16(1.27-3.41) | 1.6(0.94-2.5) | -0.99(-1.04--0.95) |
| High-middle SDI | Both | 18119.76(11963.39-26824.88) | 33329.64(20306.18-50206.43) | 1.8(1.19-2.66) | 1.66(1.01-2.5) | -0.37(-0.55--0.2) |
| High-middle SDI | Female | 8386.55(5299.28-12827.96) | 12959.95(7632.01-20317.85) | 1.43(0.91-2.19) | 1.13(0.67-1.77) | -1.04(-1.21--0.86) |
| High-middle SDI | Male | 9733.21(6597.04-14001.34) | 20369.69(12854.69-29956.16) | 2.36(1.62-3.4) | 2.38(1.5-3.49) | 0.03(-0.16-0.22) |
| Low SDI | Both | 3228.1(2501.39-4132.53) | 7704.02(6157.55-9579.34) | 1.52(1.18-1.94) | 1.66(1.34-2.06) | 0.3(0.25-0.34) |
| Low SDI | Female | 1419.82(1027.84-1947.95) | 3610.6(2842.67-4506.7) | 1.35(0.98-1.85) | 1.52(1.2-1.9) | 0.4(0.34-0.46) |
| Low SDI | Male | 1808.27(1326.86-2415.79) | 4093.41(3232.71-5219.38) | 1.69(1.24-2.22) | 1.82(1.43-2.32) | 0.25(0.21-0.28) |
| Low-middle SDI | Both | 7730.36(6177.1-9751.77) | 21802.57(16913.53-27978.85) | 1.44(1.15-1.79) | 1.73(1.35-2.21) | 0.65(0.58-0.71) |
| Low-middle SDI | Female | 3673.68(2861.28-4705.23) | 10215.71(7770.46-13269.59) | 1.37(1.06-1.74) | 1.55(1.18-2.01) | 0.36(0.29-0.43) |
| Low-middle SDI | Male | 4056.68(3199.16-5299.75) | 11586.86(8872.94-14768.6) | 1.5(1.18-1.94) | 1.94(1.5-2.46) | 0.93(0.85-1.02) |
| Middle SDI | Both | 18240.09(14735.2-22770.71) | 50249.57(37787.42-65537.25) | 1.95(1.57-2.42) | 2.16(1.62-2.81) | 0.51(0.34-0.67) |
| Middle SDI | Female | 8638.64(6852.28-10855.5) | 20255.62(14942.15-26605.04) | 1.78(1.42-2.22) | 1.65(1.22-2.17) | -0.21(-0.33--0.08) |
| Middle SDI | Male | 9601.45(7722.45-12062.67) | 29993.94(22598.67-39250.15) | 2.14(1.72-2.69) | 2.74(2.07-3.57) | 1.09(0.88-1.29) |
| **Region** | | | | | | |
| Africa | Both | 4341.99(3436.07-5477.56) | 10080.42(7996.65-12690.94) | 1.71(1.35-2.15) | 1.81(1.44-2.28) | 0.17(0.12-0.22) |
| Africa | Female | 2008.89(1544.85-2592.45) | 4665.78(3668.7-5948.66) | 1.57(1.2-2) | 1.63(1.29-2.08) | 0.14(0.09-0.2) |
| Africa | Male | 2333.1(1776.55-2991.5) | 5414.64(4289.61-6782.87) | 1.85(1.41-2.36) | 2.01(1.59-2.5) | 0.22(0.17-0.27) |
| America | Both | 8639.99(5356.47-13151.84) | 15344.61(9460.03-23261.61) | 1.45(0.9-2.2) | 1.19(0.74-1.81) | -0.58(-0.69--0.46) |
| America | Female | 4135.83(2463.59-6399.35) | 7009.13(4173.54-10919.82) | 1.19(0.71-1.84) | 0.97(0.58-1.5) | -0.64(-0.74--0.55) |
| America | Male | 4504.17(2856.61-6775.95) | 8335.48(5256.85-12416.51) | 1.78(1.13-2.68) | 1.47(0.93-2.18) | -0.59(-0.72--0.45) |
| Asia | Both | 35464.9(28002.08-44990.65) | 93739.78(68178.55-123941.94) | 1.96(1.53-2.49) | 2.09(1.52-2.76) | 0.39(0.22-0.56) |
| Asia | Female | 16276.79(12514.74-20872.83) | 38698.88(27654.07-51491) | 1.73(1.33-2.22) | 1.62(1.16-2.15) | -0.16(-0.28--0.03) |
| Asia | Male | 19188.11(15142.19-24639.19) | 55040.9(40158.26-73318.84) | 2.23(1.76-2.84) | 2.64(1.91-3.5) | 0.82(0.61-1.02) |
| Europe | Both | 15153.13(8108-25826.16) | 18501.32(9519.58-31781.77) | 1.47(0.78-2.5) | 1.12(0.58-1.93) | -1.28(-1.49--1.07) |
| Europe | Female | 7065.54(3414.93-12564.09) | 7444.17(3439.37-13362.83) | 1.09(0.53-1.95) | 0.74(0.34-1.33) | -1.79(-2.03--1.56) |
| Europe | Male | 8087.59(4439.52-13317.67) | 11057.15(6004.77-18594.23) | 2.09(1.16-3.45) | 1.66(0.9-2.8) | -1.08(-1.28--0.89) |
| Andean Latin America | Both | 315.29(241.89-400.06) | 975(684.46-1335.49) | 1.65(1.27-2.1) | 1.79(1.26-2.45) | 0.37(0.22-0.52) |
| Andean Latin America | Female | 165.39(125.9-211.71) | 503.67(349.71-702.12) | 1.7(1.29-2.16) | 1.76(1.22-2.45) | 0.07(-0.08-0.23) |
| Andean Latin America | Male | 149.9(115.46-190.92) | 471.33(331.2-634.93) | 1.61(1.24-2.05) | 1.82(1.29-2.45) | 0.71(0.54-0.88) |
| Australasia | Both | 391.73(198.73-684.57) | 633.59(343.87-1054.3) | 1.7(0.86-2.96) | 1.21(0.66-2.01) | -1.47(-1.62--1.31) |
| Australasia | Female | 166.51(76.45-304.34) | 264.11(131.02-458.76) | 1.25(0.57-2.29) | 0.89(0.44-1.54) | -1.38(-1.55--1.21) |
| Australasia | Male | 225.22(120.72-376.33) | 369.48(208.81-595.7) | 2.3(1.24-3.82) | 1.57(0.89-2.55) | -1.66(-1.82--1.51) |
| Caribbean | Both | 515.1(372.75-694.75) | 1118.93(761.19-1575.96) | 2.07(1.5-2.78) | 2.17(1.48-3.05) | -0.03(-0.12-0.06) |
| Caribbean | Female | 254.11(176.27-350.08) | 537.38(345.87-768.96) | 1.95(1.35-2.69) | 1.91(1.24-2.73) | -0.31(-0.41--0.21) |
| Caribbean | Male | 260.99(194.83-340.75) | 581.55(404.74-806.83) | 2.19(1.64-2.86) | 2.45(1.7-3.39) | 0.25(0.16-0.34) |
| Central Asia | Both | 527(325.56-810.97) | 591.98(344.42-935.97) | 1.14(0.7-1.75) | 0.91(0.53-1.42) | -1.02(-1.23--0.81) |
| Central Asia | Female | 246.15(144.71-394.12) | 251.77(141.16-418.22) | 0.9(0.53-1.44) | 0.68(0.38-1.12) | -1.27(-1.54--1) |
| Central Asia | Male | 280.86(179.59-417.57) | 340.21(204.45-517.7) | 1.51(0.97-2.24) | 1.24(0.75-1.87) | -0.85(-1.01--0.7) |
| Central Europe | Both | 2435.52(1320.81-4075.49) | 3566.06(1852.54-6001.54) | 1.72(0.93-2.87) | 1.61(0.84-2.72) | -0.32(-0.58--0.06) |
| Central Europe | Female | 1020.77(506.41-1816.85) | 1323.76(633.24-2346.89) | 1.23(0.61-2.19) | 0.99(0.47-1.75) | -0.95(-1.23--0.67) |
| Central Europe | Male | 1414.76(803.04-2287.44) | 2242.3(1200.48-3666.8) | 2.43(1.39-3.91) | 2.51(1.35-4.08) | 0.1(-0.17-0.36) |
| Central Latin America | Both | 1006.88(755.68-1303.37) | 3368.75(2286.04-4734.1) | 1.31(0.98-1.69) | 1.47(0.99-2.06) | 0.33(0.27-0.4) |
| Central Latin America | Female | 516.51(378.12-680.02) | 1566.33(1041.18-2231.52) | 1.31(0.96-1.72) | 1.25(0.83-1.79) | -0.19(-0.28--0.11) |
| Central Latin America | Male | 490.37(378.48-617.71) | 1802.42(1248.64-2508.48) | 1.31(1-1.65) | 1.71(1.19-2.38) | 0.86(0.8-0.92) |
| Central Sub-Saharan Africa | Both | 386.25(285.71-514.63) | 907.24(646.72-1243.66) | 1.96(1.45-2.61) | 1.94(1.37-2.72) | -0.16(-0.38-0.07) |
| Central Sub-Saharan Africa | Female | 169.4(122.65-229.91) | 428.4(302.04-608.48) | 1.64(1.18-2.22) | 1.65(1.13-2.41) | -0.05(-0.24-0.13) |
| Central Sub-Saharan Africa | Male | 216.85(153.49-307.62) | 478.84(333.92-721.91) | 2.32(1.65-3.46) | 2.33(1.62-3.68) | -0.13(-0.37-0.11) |
| East Asia | Both | 17222.02(13608.19-22105.95) | 42151(28994.66-58467.49) | 2.16(1.71-2.77) | 2.16(1.49-2.97) | 0.38(0.07-0.69) |
| East Asia | Female | 7811.47(5910.9-10090.83) | 14702.27(9417.92-21398.36) | 1.86(1.41-2.4) | 1.41(0.91-2.05) | -0.76(-1.02--0.5) |
| East Asia | Male | 9410.56(7086.05-12442.51) | 27448.73(18581.81-38363.89) | 2.58(1.99-3.38) | 3.14(2.14-4.33) | 1.19(0.85-1.53) |
| Eastern Europe | Both | 4279.36(2342.85-7048.67) | 4610.76(2415.96-7892.92) | 1.56(0.86-2.55) | 1.32(0.69-2.27) | -1.51(-1.96--1.05) |
| Eastern Europe | Female | 2245.38(1148.03-3845.83) | 2144.03(1009.61-3895.01) | 1.23(0.63-2.1) | 0.94(0.44-1.72) | -1.88(-2.32--1.44) |
| Eastern Europe | Male | 2033.98(1168.7-3201.74) | 2466.73(1349.01-4073.58) | 2.29(1.34-3.55) | 2.02(1.11-3.31) | -1.36(-1.82--0.9) |
| Eastern Sub-Saharan Africa | Both | 1208.13(930.08-1564.57) | 2877.78(2244.15-3642.96) | 1.76(1.35-2.25) | 1.95(1.54-2.45) | 0.35(0.29-0.4) |
| Eastern Sub-Saharan Africa | Female | 537.2(384.01-730.29) | 1326.16(1033.43-1691.19) | 1.53(1.1-2.06) | 1.7(1.33-2.17) | 0.36(0.29-0.43) |
| Eastern Sub-Saharan Africa | Male | 670.93(488.74-916.45) | 1551.61(1186.28-2054.22) | 1.99(1.46-2.64) | 2.25(1.73-2.94) | 0.39(0.35-0.44) |
| High-income Asia Pacific | Both | 4702.61(3110.44-6629.4) | 10448.63(6634.95-15091.62) | 2.47(1.63-3.47) | 2.03(1.27-2.95) | -0.56(-0.65--0.46) |
| High-income Asia Pacific | Female | 2094.47(1322.68-3007.91) | 5103.57(3199.77-7364.09) | 1.89(1.2-2.72) | 1.56(0.98-2.26) | -0.55(-0.63--0.47) |
| High-income Asia Pacific | Male | 2608.14(1758.46-3616.17) | 5345.06(3390.25-7701.1) | 3.29(2.24-4.56) | 2.58(1.63-3.72) | -0.73(-0.84--0.63) |
| High-income North America | Both | 4461.5(2308.86-7662.84) | 5201.78(2577.53-9028.36) | 1.23(0.63-2.1) | 0.79(0.39-1.37) | -1.27(-1.56--0.98) |
| High-income North America | Female | 2094.1(1031.41-3656.04) | 2308.44(1068.8-4110.74) | 0.92(0.46-1.6) | 0.6(0.27-1.06) | -1.17(-1.46--0.88) |
| High-income North America | Male | 2367.4(1282.61-3934.89) | 2893.34(1449.96-4933.03) | 1.66(0.9-2.76) | 1.02(0.51-1.75) | -1.51(-1.81--1.2) |
| North Africa and Middle East | Both | 2010.45(1444.71-2756.65) | 5230.58(3621.17-7272.54) | 1.28(0.92-1.76) | 1.32(0.91-1.84) | 0.18(0.06-0.31) |
| North Africa and Middle East | Female | 910.51(637.43-1280.21) | 2209.69(1510.8-3106.17) | 1.17(0.81-1.62) | 1.14(0.77-1.6) | -0.02(-0.14-0.1) |
| North Africa and Middle East | Male | 1099.94(785.8-1515.34) | 3020.89(2109.96-4200.07) | 1.39(1-1.91) | 1.51(1.06-2.07) | 0.34(0.21-0.47) |
| Oceania | Both | 47.06(34.3-62.05) | 127.04(94.35-170.24) | 1.81(1.33-2.37) | 2.05(1.56-2.7) | 0.42(0.38-0.47) |
| Oceania | Female | 20.88(14.76-28.37) | 55.92(40.85-76.28) | 1.66(1.19-2.23) | 1.86(1.38-2.51) | 0.4(0.34-0.46) |
| Oceania | Male | 26.18(18.66-35.32) | 71.12(52.03-96.09) | 1.96(1.41-2.67) | 2.25(1.67-2.98) | 0.45(0.4-0.49) |
| South Asia | Both | 5469.4(4328.39-6908.63) | 16734.07(12700.78-21942.15) | 1.13(0.89-1.43) | 1.34(1.02-1.74) | 0.5(0.38-0.62) |
| South Asia | Female | 2501.35(1848.2-3306.26) | 8223.08(6023.55-11047.51) | 1.07(0.79-1.42) | 1.28(0.94-1.7) | 0.5(0.36-0.63) |
| South Asia | Male | 2968.05(2301.12-3878.21) | 8510.99(6227.86-11394.88) | 1.18(0.92-1.53) | 1.4(1.03-1.85) | 0.54(0.42-0.66) |
| Southeast Asia | Both | 6431.02(4999.57-7928.3) | 20593.01(15533.78-26290.73) | 2.7(2.09-3.31) | 3.63(2.74-4.61) | 0.94(0.88-1.01) |
| Southeast Asia | Female | 3139.53(2390.01-3961.47) | 9042.57(6538.12-11755.63) | 2.46(1.89-3.07) | 2.95(2.14-3.83) | 0.52(0.44-0.6) |
| Southeast Asia | Male | 3291.49(2539.09-4093.95) | 11550.44(8777.6-14773.25) | 2.97(2.29-3.69) | 4.46(3.38-5.71) | 1.34(1.28-1.4) |
| Southern Latin America | Both | 1096.19(704.92-1593.41) | 1962.91(1193.95-3008.39) | 2.5(1.61-3.62) | 2.31(1.41-3.55) | -0.08(-0.19-0.04) |
| Southern Latin America | Female | 486.23(296.5-733.51) | 861.46(499.56-1375.4) | 1.95(1.19-2.93) | 1.72(0.99-2.76) | -0.25(-0.36--0.14) |
| Southern Latin America | Male | 609.96(404.66-859.75) | 1101.45(685.55-1635.4) | 3.23(2.17-4.55) | 3.11(1.94-4.61) | 0.08(-0.05-0.2) |
| Southern Sub-Saharan Africa | Both | 614.82(474.53-778.08) | 1389.97(1118.17-1705.04) | 2.45(1.88-3.13) | 2.72(2.18-3.34) | 0.31(0.08-0.55) |
| Southern Sub-Saharan Africa | Female | 312.48(238.57-403.98) | 686.32(537.74-857.55) | 2.18(1.64-2.86) | 2.28(1.79-2.84) | 0.22(0.06-0.37) |
| Southern Sub-Saharan Africa | Male | 302.34(231.32-397.57) | 703.65(556.41-886.12) | 2.79(2.13-3.72) | 3.37(2.68-4.2) | 0.53(0.21-0.86) |
| Tropical Latin America | Both | 1331.59(961.77-1784.97) | 2892.76(1714.36-4445.03) | 1.62(1.17-2.17) | 1.23(0.73-1.89) | -1.22(-1.34--1.09) |
| Tropical Latin America | Female | 657.6(454.43-904.61) | 1307.96(735.23-2095.78) | 1.5(1.04-2.05) | 0.99(0.56-1.58) | -1.74(-1.89--1.59) |
| Tropical Latin America | Male | 673.98(500.12-889.11) | 1584.8(990.57-2334.03) | 1.76(1.31-2.32) | 1.53(0.96-2.25) | -0.68(-0.79--0.58) |
| Western Europe | Both | 7941.39(3973.22-13939.81) | 9416.26(4699.83-16258.44) | 1.33(0.66-2.33) | 0.91(0.45-1.58) | -1.45(-1.55--1.35) |
| Western Europe | Female | 3585.39(1635.97-6503.69) | 3611.26(1578.06-6480.14) | 0.96(0.43-1.73) | 0.57(0.25-1.02) | -1.99(-2.13--1.86) |
| Western Europe | Male | 4356.01(2327.89-7348.88) | 5805.01(3057.13-9876.73) | 1.92(1.04-3.24) | 1.36(0.71-2.31) | -1.3(-1.39--1.21) |
| Western Sub-Saharan Africa | Both | 1315.36(1004.59-1725.15) | 3097.86(2366.45-3964.42) | 1.7(1.3-2.22) | 1.93(1.48-2.45) | 0.57(0.49-0.66) |
| Western Sub-Saharan Africa | Female | 599.7(431.19-821.05) | 1458.67(1119.8-1939.59) | 1.55(1.12-2.11) | 1.77(1.37-2.31) | 0.61(0.5-0.72) |
| Western Sub-Saharan Africa | Male | 715.67(522.99-972.13) | 1639.18(1243.71-2107.42) | 1.84(1.35-2.47) | 2.1(1.6-2.72) | 0.58(0.5-0.66) |

ASDR, age-standardized death rate, SDI, socio-demographic index; UI, uncertainty interval.
