# Supplementary material for: Dietary Risk-Related Colorectal Cancer Burden: Estimates From 1990 to 2019
Source: Front Nutr. 2021 Aug 24;8:690663. doi: 10.3389/fnut.2021.690663 (PMC8421520; doi:10.3389/fnut.2021.690663)
Supplement: Supplementary file 3 [file Data_Sheet_3.zip › Supplemental tables/Table S2.docx]

**Table S2** Age-standardized summary exposure value of diet high in red meat attributable to colorectal cancer and annualized rate of changes.

| **Location** | **Sex** | **Age-standardized summary exposure value (SEV) rate (per 100,000) (95% UI)** | | **Annualized rate of change (ARC, %) (95% UI)** | | |
| --- | --- | --- | --- | --- | --- | --- |
|  |  | **1990** | **2019** | **1990-2010** | **2010-2019** | **1990-2019** |
| Global | Both | 40.5(33.75-47.06) | 43.94(38.03-49.58) | 0.07(0.03-0.11) | 0.02(0-0.05) | 0.09(0.04-0.15) |
| Global | Female | 41.07(34.34-47.52) | 43.93(37.88-49.64) | 0.05(0.01-0.11) | 0.02(-0.02-0.05) | 0.07(0.02-0.14) |
| Global | Male | 39.84(33.07-46.69) | 43.95(38.16-49.54) | 0.08(0.03-0.13) | 0.02(-0.01-0.06) | 0.1(0.05-0.18) |
| **Sociodemographic Index** | | | | | | |
| High SDI | Both | 69.59(63.38-75.13) | 69.11(62.59-75.04) | -0.02(-0.03-0) | 0.01(-0.01-0.03) | -0.01(-0.03-0.01) |
| High SDI | Female | 69.76(63.71-75.2) | 68.81(61.94-74.92) | -0.02(-0.04-0) | 0.01(-0.02-0.04) | -0.01(-0.05-0.02) |
| High SDI | Male | 69.45(63.17-75.11) | 69.44(63.01-75.27) | -0.01(-0.02-0.01) | 0.01(-0.02-0.03) | 0(-0.03-0.03) |
| High-middle SDI | Both | 54.52(46.48-62.15) | 61.86(55-67.91) | 0.09(0.05-0.14) | 0.04(0.02-0.08) | 0.13(0.08-0.21) |
| High-middle SDI | Female | 55.55(47.31-63.03) | 61.53(54.49-67.93) | 0.07(0.02-0.12) | 0.04(0-0.08) | 0.11(0.04-0.19) |
| High-middle SDI | Male | 53.37(45.4-61.46) | 62.19(55.13-68.54) | 0.11(0.05-0.18) | 0.05(0.01-0.1) | 0.17(0.08-0.26) |
| Low SDI | Both | 15.38(9.82-20.84) | 16.26(10.59-22.01) | 0.03(0.01-0.05) | 0.03(0.01-0.05) | 0.06(0.03-0.09) |
| Low SDI | Female | 15.26(9.93-20.63) | 16.12(10.61-21.75) | 0.02(0-0.05) | 0.03(0.01-0.06) | 0.06(0.02-0.1) |
| Low SDI | Male | 15.49(9.85-21.05) | 16.41(10.54-22.17) | 0.03(0.01-0.06) | 0.03(0.01-0.05) | 0.06(0.03-0.1) |
| Low-middle SDI | Both | 15.32(11.07-19.87) | 20.63(16.35-25.17) | 0.3(0.22-0.43) | 0.04(0.02-0.07) | 0.35(0.26-0.48) |
| Low-middle SDI | Female | 15.36(11.2-19.72) | 20.65(16.31-25.24) | 0.3(0.22-0.42) | 0.04(0.01-0.07) | 0.34(0.26-0.48) |
| Low-middle SDI | Male | 15.28(10.91-19.98) | 20.62(16.17-25.29) | 0.29(0.21-0.44) | 0.04(0.01-0.08) | 0.35(0.25-0.51) |
| Middle SDI | Both | 28.09(20.52-35.98) | 38.18(31.52-44.62) | 0.28(0.17-0.45) | 0.07(0.03-0.12) | 0.36(0.23-0.59) |
| Middle SDI | Female | 26.68(19.34-34.02) | 34.78(28.18-41.39) | 0.24(0.15-0.38) | 0.06(0.02-0.1) | 0.3(0.19-0.5) |
| Middle SDI | Male | 29.49(20.83-38.02) | 41.65(34.47-48.37) | 0.31(0.18-0.54) | 0.08(0.02-0.15) | 0.41(0.24-0.71) |
| **Region** | | | | | | |
| Andean Latin America | Both | 26.18(17.78-34.39) | 34.34(25.68-43.15) | 0.22(0.15-0.33) | 0.08(0.03-0.13) | 0.31(0.22-0.49) |
| Andean Latin America | Female | 26.2(17.8-34.46) | 33.2(24-42.29) | 0.2(0.14-0.3) | 0.05(0-0.12) | 0.27(0.18-0.42) |
| Andean Latin America | Male | 26.15(17.83-34.28) | 35.53(26.84-44.14) | 0.23(0.16-0.35) | 0.1(0.03-0.19) | 0.36(0.23-0.58) |
| Australasia | Both | 99.2(98.77-99.58) | 98.52(97.5-99.28) | 0(-0.01-0) | 0(-0.01-0) | -0.01(-0.02-0) |
| Australasia | Female | 99.2(98.68-99.63) | 98.53(97.14-99.48) | 0(-0.01-0) | 0(-0.01-0) | -0.01(-0.02-0) |
| Australasia | Male | 99.2(98.71-99.6) | 98.51(97.02-99.37) | 0(-0.01-0) | 0(-0.02-0) | -0.01(-0.02-0) |
| Caribbean | Both | 27.48(18.52-36.18) | 28.45(19.46-37.54) | 0.01(-0.01-0.04) | 0.02(-0.01-0.07) | 0.04(0-0.09) |
| Caribbean | Female | 27.37(18.39-36.03) | 28.25(19.12-37.45) | 0.01(-0.02-0.04) | 0.02(-0.02-0.07) | 0.03(-0.02-0.1) |
| Caribbean | Male | 27.59(18.53-36.28) | 28.66(19.4-37.56) | 0.02(-0.01-0.05) | 0.02(-0.02-0.09) | 0.04(-0.01-0.11) |
| Central Asia | Both | 55.4(47.25-63.19) | 58.7(50.69-66.43) | -0.05(-0.07--0.03) | 0.11(0.07-0.17) | 0.06(0.03-0.1) |
| Central Asia | Female | 55.45(47.3-63.31) | 58.79(50.65-66.52) | -0.05(-0.07--0.03) | 0.11(0.06-0.18) | 0.06(0.02-0.12) |
| Central Asia | Male | 55.28(47.18-62.94) | 58.55(50.16-66.29) | -0.05(-0.07--0.03) | 0.11(0.07-0.17) | 0.06(0.01-0.11) |
| Central Europe | Both | 59.4(50.5-67.48) | 68.96(61.1-75.9) | 0.11(0.08-0.14) | 0.05(0.02-0.08) | 0.16(0.11-0.23) |
| Central Europe | Female | 59.45(50.67-67.59) | 68.92(60.64-75.93) | 0.1(0.07-0.14) | 0.05(0.02-0.09) | 0.16(0.11-0.24) |
| Central Europe | Male | 59.33(50.43-67.55) | 68.99(61.56-76.18) | 0.11(0.08-0.15) | 0.05(0.02-0.09) | 0.16(0.11-0.24) |
| Central Latin America | Both | 41.52(31.92-51.13) | 44.76(35.24-54.07) | 0.06(0.05-0.1) | 0.01(-0.02-0.06) | 0.08(0.04-0.14) |
| Central Latin America | Female | 41.41(31.73-50.98) | 44.47(34.99-53.92) | 0.06(0.04-0.1) | 0.01(-0.04-0.07) | 0.07(0.02-0.15) |
| Central Latin America | Male | 41.62(31.99-51.12) | 45.08(35.5-54.64) | 0.07(0.05-0.11) | 0.01(-0.03-0.07) | 0.08(0.03-0.16) |
| Central Sub-Saharan Africa | Both | 14.88(9.84-20.12) | 14.66(9.93-19.43) | -0.04(-0.08-0.02) | 0.03(-0.01-0.06) | -0.01(-0.06-0.05) |
| Central Sub-Saharan Africa | Female | 14.74(9.72-19.93) | 14.73(9.98-19.58) | -0.03(-0.08-0.02) | 0.04(-0.01-0.08) | 0(-0.05-0.07) |
| Central Sub-Saharan Africa | Male | 15.03(9.98-20.31) | 14.61(9.94-19.39) | -0.05(-0.08-0.01) | 0.02(-0.03-0.06) | -0.03(-0.08-0.04) |
| East Asia | Both | 40.23(29.57-50.66) | 70.03(61.33-77.33) | 0.52(0.33-0.85) | 0.14(0.08-0.23) | 0.74(0.47-1.2) |
| East Asia | Female | 40.56(29.48-51.51) | 70.5(60.39-78.56) | 0.53(0.3-0.9) | 0.14(0.05-0.25) | 0.74(0.44-1.29) |
| East Asia | Male | 39.92(28.49-51.18) | 69.56(60.26-77.76) | 0.52(0.3-0.88) | 0.15(0.06-0.26) | 0.74(0.43-1.27) |
| Eastern Europe | Both | 69.59(61.79-76.61) | 53.47(43.73-62.38) | -0.23(-0.29--0.17) | -0.01(-0.06-0.04) | -0.23(-0.31--0.17) |
| Eastern Europe | Female | 69.54(61.81-76.59) | 53.41(43.5-63.06) | -0.23(-0.29--0.17) | -0.01(-0.07-0.06) | -0.23(-0.32--0.16) |
| Eastern Europe | Male | 69.58(61.9-76.71) | 53.54(42.98-62.83) | -0.23(-0.29--0.17) | -0.01(-0.08-0.06) | -0.23(-0.32--0.16) |
| Eastern Sub-Saharan Africa | Both | 17.91(11.07-24.65) | 18.75(11.58-25.94) | 0.01(-0.03-0.03) | 0.04(0.01-0.08) | 0.05(0-0.09) |
| Eastern Sub-Saharan Africa | Female | 18.19(11.3-25.04) | 19.2(11.93-26.55) | 0.01(-0.05-0.06) | 0.04(0-0.1) | 0.06(-0.02-0.13) |
| Eastern Sub-Saharan Africa | Male | 17.63(10.81-24.3) | 18.27(10.96-25.51) | 0(-0.03-0.02) | 0.04(0.01-0.08) | 0.04(0-0.07) |
| High-income Asia Pacific | Both | 31.47(21.53-41.22) | 45.66(36.67-54.15) | 0.36(0.23-0.59) | 0.07(0.03-0.12) | 0.45(0.29-0.76) |
| High-income Asia Pacific | Female | 30.97(21.07-40.56) | 42.1(31.91-51.55) | 0.29(0.18-0.49) | 0.05(-0.01-0.13) | 0.36(0.2-0.61) |
| High-income Asia Pacific | Male | 31.97(21.99-41.86) | 49.06(40.65-57.07) | 0.42(0.27-0.69) | 0.08(0.03-0.14) | 0.53(0.35-0.91) |
| High-income North America | Both | 80.1(74.02-85.49) | 77.32(70.02-83.29) | -0.03(-0.06--0.01) | 0(-0.04-0.04) | -0.03(-0.08-0.01) |
| High-income North America | Female | 79.87(73.76-85.28) | 76.33(67.6-83.23) | -0.04(-0.08--0.01) | 0(-0.07-0.06) | -0.04(-0.12-0.02) |
| High-income North America | Male | 80.44(74.4-85.99) | 78.4(71.09-85.09) | -0.02(-0.05-0) | 0(-0.06-0.05) | -0.03(-0.08-0.03) |
| North Africa and Middle East | Both | 23.74(15.16-31.96) | 23.63(15.09-31.83) | -0.05(-0.07--0.04) | 0.05(0.02-0.08) | 0(-0.03-0.02) |
| North Africa and Middle East | Female | 23.45(14.87-31.72) | 23.34(14.77-31.96) | -0.05(-0.07--0.03) | 0.05(0.02-0.08) | 0(-0.04-0.03) |
| North Africa and Middle East | Male | 24(15.44-32.36) | 23.9(15.37-32.22) | -0.05(-0.07--0.03) | 0.05(0.02-0.08) | 0(-0.04-0.03) |
| Oceania | Both | 33.7(23.99-44.19) | 29.76(20.31-39.51) | -0.12(-0.21--0.05) | 0.01(-0.05-0.07) | -0.12(-0.22--0.03) |
| Oceania | Female | 33.68(23.63-44.19) | 29.73(19.42-40.48) | -0.12(-0.24--0.02) | 0.01(-0.07-0.09) | -0.12(-0.25-0.02) |
| Oceania | Male | 33.71(23.72-44.11) | 29.79(20.16-39.49) | -0.13(-0.23--0.02) | 0.01(-0.06-0.09) | -0.12(-0.24-0.01) |
| South Asia | Both | 7.44(4.92-10.27) | 7.77(5.09-10.8) | -0.02(-0.04-0.01) | 0.06(0.03-0.09) | 0.04(0-0.09) |
| South Asia | Female | 7.13(4.74-9.73) | 7.48(4.9-10.4) | -0.02(-0.06-0.03) | 0.07(0.02-0.11) | 0.05(-0.02-0.12) |
| South Asia | Male | 7.71(5.03-10.75) | 8.06(5.23-11.21) | -0.02(-0.03-0) | 0.06(0.03-0.1) | 0.04(0.01-0.09) |
| Southeast Asia | Both | 15.33(9.33-21.32) | 23.56(16.63-30.56) | 0.4(0.28-0.62) | 0.1(0.06-0.15) | 0.54(0.38-0.83) |
| Southeast Asia | Female | 15.37(9.31-21.44) | 23.57(16.61-30.58) | 0.4(0.28-0.62) | 0.09(0.05-0.15) | 0.53(0.38-0.84) |
| Southeast Asia | Male | 15.28(9.31-21.21) | 23.52(16.52-30.47) | 0.4(0.28-0.61) | 0.1(0.05-0.16) | 0.54(0.38-0.83) |
| Southern Latin America | Both | 85.82(82.6-88.86) | 91.26(87.79-94.23) | 0.06(0.03-0.08) | 0.01(-0.01-0.02) | 0.06(0.04-0.09) |
| Southern Latin America | Female | 85.75(82.55-88.76) | 91.36(87.44-94.48) | 0.06(0.03-0.08) | 0.01(-0.01-0.03) | 0.07(0.03-0.1) |
| Southern Latin America | Male | 85.89(82.6-88.82) | 91.16(87.12-94.42) | 0.05(0.03-0.08) | 0.01(-0.02-0.03) | 0.06(0.03-0.1) |
| Southern Sub-Saharan Africa | Both | 38.55(28.87-48.19) | 43.49(33.34-53.14) | 0.1(0.07-0.14) | 0.03(-0.02-0.1) | 0.13(0.06-0.23) |
| Southern Sub-Saharan Africa | Female | 38.57(28.96-48.22) | 43.21(32.89-53.17) | 0.09(0.06-0.14) | 0.03(-0.05-0.11) | 0.12(0.03-0.24) |
| Southern Sub-Saharan Africa | Male | 38.51(28.71-48.01) | 43.79(33.67-53.18) | 0.1(0.06-0.16) | 0.03(-0.04-0.13) | 0.14(0.05-0.27) |
| Tropical Latin America | Both | 54.74(44.8-63.8) | 86.52(81.23-90.68) | 0.63(0.44-0.93) | -0.03(-0.07-0) | 0.58(0.41-0.86) |
| Tropical Latin America | Female | 54.64(44.64-63.8) | 86.67(80.66-91.53) | 0.64(0.44-0.93) | -0.03(-0.08-0.01) | 0.59(0.4-0.86) |
| Tropical Latin America | Male | 54.84(44.42-64.13) | 86.36(80.32-90.97) | 0.63(0.43-0.94) | -0.03(-0.08-0.01) | 0.57(0.39-0.85) |
| Western Europe | Both | 82.53(77.57-86.99) | 78.74(73.01-84.03) | -0.04(-0.06--0.03) | 0(-0.02-0.01) | -0.05(-0.07--0.03) |
| Western Europe | Female | 82.53(77.59-87.07) | 78.72(72.63-84.12) | -0.04(-0.06--0.03) | 0(-0.03-0.02) | -0.05(-0.08--0.02) |
| Western Europe | Male | 82.52(77.62-86.97) | 78.77(72.98-84.1) | -0.04(-0.06--0.03) | 0(-0.03-0.02) | -0.05(-0.07--0.02) |
| Western Sub-Saharan Africa | Both | 17.4(10.77-24.12) | 19.28(12.04-26.48) | 0.08(0.06-0.1) | 0.03(0-0.06) | 0.11(0.07-0.15) |
| Western Sub-Saharan Africa | Female | 17.55(10.84-24.33) | 19.14(11.93-26.39) | 0.07(0.05-0.09) | 0.02(-0.01-0.06) | 0.09(0.05-0.14) |
| Western Sub-Saharan Africa | Male | 17.28(10.7-23.92) | 19.44(12.06-26.62) | 0.09(0.07-0.12) | 0.03(0-0.07) | 0.13(0.08-0.19) |

SDI, socio-demographic index; UI, uncertainty interval.
