# Supplementary material for: Dietary Risk-Related Colorectal Cancer Burden: Estimates From 1990 to 2019
Source: Front Nutr. 2021 Aug 24;8:690663. doi: 10.3389/fnut.2021.690663 (PMC8421520; doi:10.3389/fnut.2021.690663)
Supplement: Supplementary file 3 [file Data_Sheet_3.zip › Supplemental tables/Table S20.docx]

**Table S20** DALYs, ASRs and change trends of colorectal cancer attributable to diet low in calcium between 1990 and 2019 by SDI, regions and sex.

| **Location** | **Sex** | **DALYs (No.×1000, 95%UI)** | | **ASR (95%UI)** | | **EAPC (95%CI)** |
| --- | --- | --- | --- | --- | --- | --- |
|  |  | **1990** | **2019** | **1990** | **2019** | **1990-2019** |
| Global | Both | 1577.74(1166.95-2139.2) | 3144.6(2248.01-4259.66) | 38.84(28.56-53.06) | 38.18(27.22-51.79) | -0.03(-0.12-0.07) |
| Global | Female | 701.61(502.46-959.33) | 1243.78(887.24-1702.85) | 32.58(23.2-44.63) | 28.68(20.49-39.24) | -0.51(-0.57--0.45) |
| Global | Male | 876.12(646.64-1184.73) | 1900.82(1364.05-2578.45) | 46.43(33.89-63.1) | 48.83(34.94-66.08) | 0.27(0.15-0.4) |
| **Sociodemographic Index** | | | | | | |
| High SDI | Both | 332.02(191.26-530.68) | 434.23(250.82-679.68) | 32.34(18.63-51.57) | 23.93(13.79-37.19) | -0.98(-1.03--0.94) |
| High SDI | Female | 136.11(75.21-224.63) | 166.68(94.5-262.26) | 23.1(12.85-37.86) | 16.47(9.35-25.81) | -1.1(-1.15--1.05) |
| High SDI | Male | 195.91(114.15-305.73) | 267.55(155.51-413.98) | 44.44(25.94-69.32) | 32.33(18.81-50.06) | -1.05(-1.1--1) |
| High-middle SDI | Both | 440.92(293.56-647.31) | 731.73(454.06-1092.35) | 40.64(27.06-59.82) | 36.38(22.61-54.21) | -0.5(-0.68--0.33) |
| High-middle SDI | Female | 194.09(124.94-291.11) | 265.24(157.65-410.93) | 32.36(20.84-48.51) | 24.32(14.5-37.62) | -1.25(-1.42--1.07) |
| High-middle SDI | Male | 246.83(166.31-353.69) | 466.49(294.11-690.92) | 51.78(34.92-74.38) | 50.85(32.09-75.18) | -0.09(-0.28-0.11) |
| Low SDI | Both | 88.81(68.98-113.83) | 202.69(161.18-252.08) | 35.02(27.2-44.9) | 36.68(29.34-45.68) | 0.13(0.08-0.18) |
| Low SDI | Female | 39.85(29.28-55) | 94.62(74.27-117.87) | 31.45(22.96-43.15) | 33.51(26.39-41.72) | 0.19(0.12-0.26) |
| Low SDI | Male | 48.96(36.17-66.3) | 108.06(84.52-138.5) | 38.44(28.32-51.6) | 40(31.53-51.05) | 0.11(0.07-0.15) |
| Low-middle SDI | Both | 212.08(169.91-268.05) | 535.45(413.7-692.79) | 33.1(26.49-41.61) | 38.08(29.5-49.21) | 0.49(0.42-0.56) |
| Low-middle SDI | Female | 100.75(78.16-130.04) | 244.43(184.5-320.17) | 31.73(24.65-40.57) | 33.64(25.45-43.89) | 0.14(0.07-0.21) |
| Low-middle SDI | Male | 111.33(87.93-145.76) | 291.02(221.69-373.68) | 34.44(27.17-44.76) | 42.87(32.76-54.94) | 0.82(0.73-0.92) |
| Middle SDI | Both | 503.02(407.4-627.5) | 1238.48(933.06-1623.8) | 45.26(36.63-56.33) | 48.51(36.59-63.65) | 0.4(0.24-0.56) |
| Middle SDI | Female | 230.4(182.07-292.1) | 471.94(349.2-620.63) | 41.06(32.61-51.91) | 35.88(26.56-47.19) | -0.44(-0.53--0.34) |
| Middle SDI | Male | 272.62(217.99-341.45) | 766.54(578.05-1005.94) | 49.74(39.92-62.58) | 62.19(47.12-81.16) | 1.03(0.82-1.23) |
| **Region** | | | | | | |
| Africa | Both | 115.1(91.21-144.52) | 261.62(205.64-328.69) | 38.1(30.15-48.17) | 39.14(31.01-49.3) | 0.05(0-0.1) |
| Africa | Female | 52.11(39.92-68.02) | 117.8(91.75-150.71) | 34.25(26.36-44.32) | 34.36(26.91-43.99) | 0.01(-0.06-0.09) |
| Africa | Male | 62.98(48.07-80.94) | 143.82(113.31-181.26) | 41.94(31.96-53.69) | 44.23(34.96-55.57) | 0.11(0.07-0.16) |
| America | Both | 183.63(114.89-275.47) | 321.52(196.2-480.49) | 30.02(18.74-45.06) | 25.76(15.76-38.47) | -0.44(-0.55--0.32) |
| America | Female | 81.95(50.85-124.56) | 136.71(81.93-209.64) | 24.24(15.09-36.8) | 20.26(12.17-30.96) | -0.52(-0.62--0.43) |
| America | Male | 101.68(64.2-152.85) | 184.81(115.95-273.92) | 37.07(23.37-55.9) | 32.06(20.15-47.43) | -0.42(-0.54--0.29) |
| Asia | Both | 957.94(759.09-1215.12) | 2215.33(1615.53-2939.33) | 44.6(35.34-56.63) | 45.62(33.4-60.44) | 0.24(0.09-0.4) |
| Asia | Female | 428.96(328.52-550.32) | 862.49(622.42-1144.76) | 39.52(30.4-50.59) | 34.5(24.92-45.81) | -0.44(-0.54--0.34) |
| Asia | Male | 528.98(414.19-682.1) | 1352.84(983.92-1803.83) | 50.16(39.44-64.43) | 57.63(42.02-76.69) | 0.74(0.53-0.95) |
| Europe | Both | 318.62(169.43-542.67) | 341.38(175.11-585.88) | 31.06(16.52-52.95) | 22.86(11.69-39.2) | -1.51(-1.73--1.29) |
| Europe | Female | 137.56(67.03-243.08) | 124.88(57.29-222.07) | 22.74(11.08-40.23) | 14.6(6.58-25.98) | -2.07(-2.31--1.82) |
| Europe | Male | 181.06(98.67-299.16) | 216.49(115.87-364.03) | 43.25(23.58-71.36) | 33.34(17.88-56.12) | -1.28(-1.49--1.07) |
| Andean Latin America | Both | 7.57(5.79-9.71) | 20.93(14.5-29.05) | 35.49(27.19-45.23) | 37.01(25.67-51.39) | 0.21(0.06-0.36) |
| Andean Latin America | Female | 3.83(2.9-4.96) | 10.3(7.06-14.43) | 35.22(26.66-45.35) | 35.14(24.13-49.05) | -0.09(-0.24-0.07) |
| Andean Latin America | Male | 3.74(2.86-4.74) | 10.63(7.36-14.5) | 35.71(27.36-45.25) | 38.94(27.11-52.89) | 0.52(0.35-0.68) |
| Australasia | Both | 8.3(4.15-14.51) | 11.59(6.28-19.38) | 35.8(17.9-62.73) | 24.21(13.03-40.55) | -1.66(-1.82--1.5) |
| Australasia | Female | 3.27(1.43-5.93) | 4.45(2.2-7.65) | 25.89(11.36-46.79) | 17.33(8.58-29.69) | -1.61(-1.79--1.42) |
| Australasia | Male | 5.02(2.67-8.55) | 7.13(3.97-11.63) | 47.77(25.43-80.73) | 31.82(17.65-52.12) | -1.76(-1.92--1.61) |
| Caribbean | Both | 11.64(8.38-15.65) | 23.98(16.44-33.64) | 44.12(31.83-59.3) | 46.51(31.91-65.2) | 0(-0.08-0.09) |
| Caribbean | Female | 5.6(3.87-7.79) | 10.96(7.14-15.55) | 41.04(28.2-57.2) | 40.12(26.13-57.06) | -0.31(-0.4--0.21) |
| Caribbean | Male | 6.04(4.49-7.92) | 13.03(9.06-18.18) | 47.35(35.15-62.04) | 53.55(37.41-74.72) | 0.29(0.19-0.39) |
| Central Asia | Both | 14.91(9.22-22.85) | 15.56(8.97-24.69) | 29.84(18.42-45.8) | 20.19(11.7-32.06) | -1.79(-2.01--1.57) |
| Central Asia | Female | 6.44(3.74-10.31) | 6.11(3.35-10.32) | 22.97(13.35-36.78) | 14.52(8.03-24.27) | -2.1(-2.39--1.81) |
| Central Asia | Male | 8.46(5.41-12.54) | 9.44(5.63-14.4) | 39.17(25.09-58.17) | 27.62(16.57-42) | -1.57(-1.74--1.4) |
| Central Europe | Both | 54.21(29.08-91.96) | 68.91(35.4-117.72) | 36.97(19.89-62.69) | 33.02(16.93-56.49) | -0.51(-0.76--0.26) |
| Central Europe | Female | 21.2(10.26-37.86) | 23.2(10.9-40.95) | 25.67(12.39-45.85) | 19.49(9.09-34.41) | -1.15(-1.41--0.9) |
| Central Europe | Male | 33.02(18.3-54.11) | 45.72(24.04-76.66) | 51.9(29-84.82) | 50.44(26.5-84.19) | -0.16(-0.42-0.11) |
| Central Latin America | Both | 24.81(18.68-32.09) | 77.24(51.72-109.56) | 28.03(21.04-36.25) | 32.22(21.52-45.69) | 0.43(0.36-0.5) |
| Central Latin America | Female | 12.23(8.93-16.16) | 33.94(21.93-49.45) | 27.02(19.72-35.61) | 26.46(17.15-38.46) | -0.1(-0.2--0.01) |
| Central Latin America | Male | 12.58(9.76-15.98) | 43.3(29.42-60.46) | 29.06(22.51-36.71) | 38.79(26.46-54.12) | 0.93(0.87-0.99) |
| Central Sub-Saharan Africa | Both | 10.85(8.04-14.41) | 25.28(17.87-34.32) | 44.4(32.94-59.42) | 43.36(30.85-59.39) | -0.19(-0.41-0.03) |
| Central Sub-Saharan Africa | Female | 4.8(3.42-6.59) | 11.52(8.1-16.2) | 36.96(26.74-50.03) | 36.84(26.03-51.74) | -0.08(-0.25-0.1) |
| Central Sub-Saharan Africa | Male | 6.05(4.25-8.53) | 13.76(9.44-20.87) | 52.82(37.26-75.17) | 51.61(35.98-77.94) | -0.23(-0.47-0.01) |
| East Asia | Both | 480.86(377.29-622.35) | 1021.67(703.64-1418.86) | 51.01(40.24-65.38) | 49.27(33.83-68.34) | 0.28(-0.02-0.58) |
| East Asia | Female | 211.33(159.38-275.34) | 336.63(216.96-491.18) | 44.51(33.68-57.91) | 31.49(20.36-45.95) | -1.03(-1.25--0.82) |
| East Asia | Male | 269.53(203.6-356.36) | 685.04(455.99-964.19) | 58.68(44.46-77.42) | 69.1(46.67-96.54) | 1.12(0.76-1.48) |
| Eastern Europe | Both | 102.26(54.86-170.1) | 98.92(51.41-170.77) | 36.48(19.66-60.55) | 29.38(15.24-50.76) | -1.79(-2.27--1.3) |
| Eastern Europe | Female | 50.18(24.91-86.93) | 42.19(19.18-77.96) | 28.58(14.16-49.59) | 20.43(9.11-38.07) | -2.23(-2.7--1.75) |
| Eastern Europe | Male | 52.08(29.32-82.79) | 56.73(30.79-95.1) | 51.24(29.16-80.61) | 43.57(23.72-72.77) | -1.55(-2.03--1.07) |
| Eastern Sub-Saharan Africa | Both | 33.84(26.08-43.9) | 78.58(60.66-100.37) | 41.27(31.74-53.6) | 43.77(34.06-55.67) | 0.16(0.09-0.22) |
| Eastern Sub-Saharan Africa | Female | 15.4(10.83-21.32) | 35.73(27.48-45.85) | 36.4(25.95-49.58) | 38.07(29.48-48.69) | 0.1(0.01-0.19) |
| Eastern Sub-Saharan Africa | Male | 18.44(13.38-25.83) | 42.84(32.33-57.11) | 46.18(33.69-63.58) | 50.05(38.11-66.45) | 0.24(0.2-0.29) |
| High-income Asia Pacific | Both | 107.45(71.09-152.14) | 173.19(109.15-251.28) | 53.22(35.28-75.3) | 41.41(26.34-60.11) | -0.78(-0.88--0.67) |
| High-income Asia Pacific | Female | 44.44(28.12-64.54) | 73.03(46.18-106.19) | 39.79(25.23-57.73) | 30.54(18.96-44.6) | -0.83(-0.92--0.75) |
| High-income Asia Pacific | Male | 63.01(43.01-87.32) | 100.17(63.9-144.47) | 70.56(47.91-97.7) | 53.55(34.31-77.38) | -0.86(-0.98--0.74) |
| High-income North America | Both | 84.67(42.29-145.2) | 98.22(47.23-169.58) | 24.31(12.12-41.6) | 16.42(7.86-28.35) | -1.01(-1.34--0.69) |
| High-income North America | Female | 35.44(16.86-62.35) | 38.96(17.33-68.67) | 17.35(8.06-30.23) | 11.87(5.29-20.78) | -0.87(-1.21--0.53) |
| High-income North America | Male | 49.23(25.6-82.75) | 59.27(29.29-102.12) | 33.31(17.42-55.84) | 21.58(10.7-36.96) | -1.24(-1.55--0.92) |
| North Africa and Middle East | Both | 54.41(39.43-75.17) | 133.32(92.91-185.16) | 29.47(21.29-40.71) | 28.88(20.03-40.29) | -0.06(-0.19-0.06) |
| North Africa and Middle East | Female | 24.39(17.04-34.25) | 55.57(37.48-77.47) | 26.8(18.81-37.55) | 24.64(16.71-34.55) | -0.29(-0.39--0.18) |
| North Africa and Middle East | Male | 30.03(21.32-42.01) | 77.74(54.26-108.65) | 32.07(22.84-44.3) | 32.99(23.1-45.91) | 0.11(-0.03-0.25) |
| Oceania | Both | 1.37(0.99-1.82) | 3.69(2.7-5.07) | 41.63(30.37-54.89) | 46.78(34.77-62.83) | 0.4(0.36-0.45) |
| Oceania | Female | 0.6(0.42-0.81) | 1.58(1.14-2.19) | 37.69(26.61-51.08) | 41.87(30.59-57.15) | 0.38(0.32-0.43) |
| Oceania | Male | 0.78(0.55-1.06) | 2.11(1.52-2.89) | 45.43(32.32-61.37) | 51.5(37.72-69.51) | 0.42(0.39-0.46) |
| South Asia | Both | 148.45(116.9-188.4) | 395.31(295.44-525.27) | 24.9(19.64-31.6) | 27.8(21-36.66) | 0.33(0.23-0.43) |
| South Asia | Female | 69.39(51.74-91.86) | 192.51(139.57-261.58) | 24.05(17.85-31.77) | 26.67(19.38-36.1) | 0.27(0.15-0.39) |
| South Asia | Male | 79.06(60.65-103.28) | 202.8(145.58-275.83) | 25.69(19.91-33.65) | 28.99(21.06-39.07) | 0.4(0.29-0.51) |
| Southeast Asia | Both | 178.91(138.61-221.32) | 530(401.15-673.32) | 63.92(49.73-78.86) | 82.87(62.63-105.53) | 0.8(0.73-0.87) |
| Southeast Asia | Female | 85.43(64.16-109.48) | 220.34(159.24-287.05) | 58.08(44.01-73.83) | 65.46(47.22-85.46) | 0.31(0.22-0.39) |
| Southeast Asia | Male | 93.48(71.82-116.64) | 309.66(235.42-397.15) | 70.53(54.48-87.96) | 102.78(78.3-131.72) | 1.22(1.15-1.28) |
| Southern Latin America | Both | 23.23(14.67-34.29) | 38.48(23.05-59.49) | 50.53(32.04-74.46) | 46.72(27.99-72.28) | -0.02(-0.14-0.09) |
| Southern Latin America | Female | 9.62(5.78-14.79) | 15.54(8.7-25.19) | 37.91(22.67-58.25) | 33.69(18.98-54.87) | -0.15(-0.26--0.04) |
| Southern Latin America | Male | 13.61(8.9-19.59) | 22.94(14.11-34.49) | 66.14(43.56-94.73) | 62.82(38.81-94.45) | 0.06(-0.06-0.18) |
| Southern Sub-Saharan Africa | Both | 15.37(12.03-19.21) | 34.25(27.6-42.38) | 52.91(41.05-66.42) | 58.35(47.1-71.95) | 0.37(0.13-0.61) |
| Southern Sub-Saharan Africa | Female | 7.21(5.66-9.09) | 15.6(12.18-19.6) | 44.9(34.83-57.01) | 47.12(36.87-58.92) | 0.4(0.25-0.55) |
| Southern Sub-Saharan Africa | Male | 8.16(6.3-10.53) | 18.65(14.81-23.19) | 62.64(48.08-81.88) | 73.47(57.97-92.51) | 0.44(0.09-0.79) |
| Tropical Latin America | Both | 33.6(24.02-45.26) | 66.09(38.74-102.35) | 35.05(25.21-47.1) | 26.97(15.82-41.69) | -1.17(-1.31--1.04) |
| Tropical Latin America | Female | 16.01(10.94-22.1) | 28.39(15.64-45.8) | 31.78(21.8-43.73) | 21.33(11.78-34.36) | -1.67(-1.82--1.53) |
| Tropical Latin America | Male | 17.59(13.06-23.3) | 37.7(23.22-56.16) | 38.69(28.82-51.02) | 33.72(20.83-50.04) | -0.72(-0.84--0.59) |
| Western Europe | Both | 148.24(73.3-259.16) | 152.61(75.79-263.59) | 25.98(12.8-45.45) | 17.09(8.47-29.77) | -1.59(-1.66--1.52) |
| Western Europe | Female | 60.57(26.92-109.42) | 51.91(22.94-91.76) | 17.92(7.85-31.97) | 10.16(4.4-17.6) | -2.14(-2.23--2.05) |
| Western Europe | Male | 87.67(45.63-149.57) | 100.71(51.85-170.92) | 37.04(19.35-63.14) | 25.28(12.88-42.89) | -1.44(-1.52--1.36) |
| Western Sub-Saharan Africa | Both | 32.78(24.86-43.66) | 76.78(58.56-99.14) | 36.32(27.62-48.05) | 39.46(30.05-50.75) | 0.38(0.3-0.46) |
| Western Sub-Saharan Africa | Female | 14.24(10.19-19.54) | 35.34(26.4-47.44) | 32.42(23.29-44.64) | 35.16(26.7-47.16) | 0.4(0.28-0.52) |
| Western Sub-Saharan Africa | Male | 18.54(13.51-25.38) | 41.44(30.89-53.47) | 39.95(29.17-54.31) | 44.13(33.38-56.9) | 0.43(0.36-0.5) |

ASDR, age-standardized death rate; DALYs, disability-adjusted life years; SDI, socio-demographic index; UI, uncertainty interval.
