# Supplementary material for: Dietary Risk-Related Colorectal Cancer Burden: Estimates From 1990 to 2019
Source: Front Nutr. 2021 Aug 24;8:690663. doi: 10.3389/fnut.2021.690663 (PMC8421520; doi:10.3389/fnut.2021.690663)
Supplement: Supplementary file 3 [file Data_Sheet_3.zip › Supplemental tables/Table S21.docx]

**Table S21** Deaths, ASDRs and change trends of colorectal cancer attributable to dietary risks between 1990 and 2019 in countries and territories.

| **Location** | **Sex** | **Deaths (95%UI)** | | **ASDR (95%UI)** | | **EAPC (95%CI)** |
| --- | --- | --- | --- | --- | --- | --- |
|  |  | **1990** | **2019** | **1990** | **2019** | **1990-2019** |
| Afghanistan | Both | 225.59(132.94-344.03) | 443.88(282.62-623.44) | 3.33(2.06-4.96) | 3.63(2.53-4.93) | 0.43(0.28-0.58) |
| Afghanistan | Female | 112.67(60.37-194.33) | 247.35(141.95-371.62) | 3.41(2.04-5.63) | 3.78(2.42-5.33) | 0.48(0.32-0.64) |
| Afghanistan | Male | 112.91(63.51-213.88) | 196.54(129.18-344.72) | 3.21(1.86-6.05) | 3.5(2.4-5.99) | 0.45(0.3-0.59) |
| Albania | Both | 59.26(43.15-72.19) | 126.78(81.97-180) | 3.04(2.23-3.71) | 3.01(1.94-4.27) | 0.28(0.01-0.56) |
| Albania | Female | 26.18(19.15-32.31) | 59.27(38.06-83.49) | 2.47(1.81-3.05) | 2.6(1.68-3.65) | 0.47(0.28-0.65) |
| Albania | Male | 33.08(24.02-40.46) | 67.51(43.6-95.87) | 3.82(2.82-4.66) | 3.48(2.23-4.92) | 0.01(-0.39-0.41) |
| Algeria | Both | 268(189.98-344.63) | 744.58(481.48-1001.25) | 2.67(1.92-3.39) | 2.54(1.63-3.39) | -0.26(-0.29--0.22) |
| Algeria | Female | 129.37(89.47-168.6) | 348.45(224.94-475.69) | 2.6(1.85-3.36) | 2.48(1.6-3.38) | -0.17(-0.2--0.14) |
| Algeria | Male | 138.64(97.95-181.49) | 396.13(259.15-541.89) | 2.75(1.98-3.52) | 2.62(1.72-3.59) | -0.3(-0.35--0.26) |
| American Samoa | Both | 1.12(0.81-1.4) | 2.54(1.84-3.21) | 5.72(4.1-7.18) | 5.87(4.33-7.4) | 0.16(0.02-0.29) |
| American Samoa | Female | 0.45(0.32-0.59) | 1.09(0.78-1.44) | 4.82(3.44-6.25) | 4.85(3.44-6.37) | 0.07(-0.11-0.25) |
| American Samoa | Male | 0.67(0.47-0.85) | 1.44(1.06-1.82) | 6.65(4.71-8.54) | 7.03(5.21-8.82) | 0.29(0.18-0.4) |
| Andorra | Both | 4.4(2.91-6.24) | 10.39(6.76-14.07) | 8.9(5.97-12.41) | 7.09(4.61-9.65) | -0.83(-0.88--0.78) |
| Andorra | Female | 0.96(0.61-1.46) | 2.71(1.67-3.84) | 4.06(2.62-6.09) | 3.44(2.13-4.95) | -0.57(-0.74--0.39) |
| Andorra | Male | 3.44(2.26-4.84) | 7.68(5.01-10.22) | 14.1(9.39-19.28) | 11.11(7.22-14.78) | -0.83(-0.87--0.79) |
| Angola | Both | 117.99(80.98-175.06) | 362.53(259.51-477.79) | 3.36(2.29-4.79) | 3.72(2.74-4.78) | 0.35(0.19-0.51) |
| Angola | Female | 48.26(29.42-79.36) | 163.84(114.98-224.72) | 2.73(1.66-4.28) | 3.07(2.26-4.08) | 0.39(0.28-0.5) |
| Angola | Male | 69.73(43.86-106.15) | 198.69(135.65-266.05) | 4.04(2.53-5.94) | 4.58(3.21-6.01) | 0.44(0.23-0.64) |
| Antigua | Both | 2.53(1.92-3.02) | 5.37(3.83-6.68) | 4.55(3.44-5.43) | 5.8(4.17-7.19) | 0.71(0.56-0.85) |
| Antigua | Female | 1.41(1.07-1.72) | 3.21(2.27-4.01) | 4.17(3.12-5.1) | 6.35(4.53-7.95) | 1.15(0.97-1.33) |
| Antigua | Male | 1.12(0.85-1.35) | 2.16(1.56-2.75) | 5.01(3.81-6.01) | 5.11(3.72-6.46) | 0.2(-0.15-0.54) |
| Argentina | Both | 2660.65(2033.94-3139.41) | 4878.96(3646.37-5847.16) | 8.54(6.55-10.08) | 8.93(6.67-10.7) | 0.14(0.03-0.25) |
| Argentina | Female | 1210.59(909.64-1439.37) | 2227.43(1602.46-2722.98) | 6.84(5.13-8.14) | 6.96(5.04-8.49) | 0.02(-0.09-0.14) |
| Argentina | Male | 1450.06(1122.39-1707.99) | 2651.53(2022.74-3193.13) | 10.8(8.43-12.72) | 11.57(8.82-13.94) | 0.26(0.14-0.37) |
| Armenia | Both | 134.94(97.8-161.46) | 188.58(127.95-244.65) | 5.1(3.69-6.11) | 4.61(3.14-5.96) | -0.59(-0.7--0.48) |
| Armenia | Female | 71.22(50.34-86.85) | 101.58(68.12-133.19) | 4.7(3.33-5.72) | 4.24(2.83-5.55) | -0.56(-0.68--0.44) |
| Armenia | Male | 63.72(46.86-75.65) | 86.99(60.15-112.91) | 5.72(4.27-6.76) | 5.12(3.54-6.65) | -0.67(-0.83--0.5) |
| Australia | Both | 1562.15(1171.23-1899.74) | 2319.9(1706.8-2869.29) | 8.12(6.09-9.86) | 5.35(3.99-6.58) | -1.8(-1.95--1.65) |
| Australia | Female | 730.13(545.48-893.7) | 1066.24(766.3-1343.63) | 6.71(5.01-8.21) | 4.43(3.23-5.54) | -1.76(-1.9--1.62) |
| Australia | Male | 832.02(626.12-1006.77) | 1253.66(939.64-1539.08) | 9.96(7.52-12.09) | 6.39(4.79-7.84) | -1.92(-2.1--1.75) |
| Austria | Both | 1030.84(754.15-1289.98) | 786.95(559.92-991.66) | 8.58(6.28-10.7) | 4.11(2.91-5.16) | -2.93(-3.07--2.79) |
| Austria | Female | 521.47(375.91-657.5) | 340.7(233.89-438.41) | 6.68(4.82-8.44) | 2.98(2.06-3.78) | -3.11(-3.24--2.97) |
| Austria | Male | 509.37(374.44-629.82) | 446.26(324.18-561.48) | 11.66(8.58-14.42) | 5.57(4.06-7.02) | -3(-3.14--2.85) |
| Azerbaijan | Both | 210.45(161.06-248.01) | 368.08(255.25-481.62) | 4.15(3.19-4.88) | 4.24(2.9-5.59) | 0.41(0.04-0.77) |
| Azerbaijan | Female | 100.24(75.89-119.77) | 167.26(112.79-230.41) | 3.43(2.61-4.11) | 3.59(2.37-5.07) | 0.54(0.21-0.88) |
| Azerbaijan | Male | 110.21(84.8-131.75) | 200.82(132.03-270.15) | 5.19(3.97-6.17) | 5.07(3.36-6.88) | 0.19(-0.23-0.62) |
| Bahamas | Both | 8.57(6.18-10.61) | 22.87(16.15-30.32) | 5.84(4.2-7.19) | 6.26(4.42-8.23) | 0.45(0.35-0.55) |
| Bahamas | Female | 3.92(2.74-4.86) | 9.95(6.81-13.37) | 4.68(3.28-5.84) | 4.92(3.39-6.6) | 0.38(0.24-0.53) |
| Bahamas | Male | 4.65(3.29-5.79) | 12.92(9.17-17.17) | 7.44(5.28-9.26) | 7.96(5.69-10.46) | 0.44(0.31-0.57) |
| Bahrain | Both | 5.95(4.04-7.71) | 24.78(15.2-34.1) | 3.96(2.69-5.08) | 3.43(2.16-4.6) | -0.66(-0.94--0.37) |
| Bahrain | Female | 2.24(1.46-2.94) | 8.5(5.13-11.79) | 3.17(2.05-4.14) | 2.85(1.77-3.88) | -0.32(-0.62--0.02) |
| Bahrain | Male | 3.71(2.55-4.85) | 16.28(10.28-23.05) | 4.85(3.31-6.24) | 3.98(2.52-5.44) | -0.94(-1.27--0.6) |
| Bangladesh | Both | 910.46(623.16-1232.11) | 2567.79(1730.04-3796.76) | 2.08(1.4-2.83) | 2.11(1.42-3.12) | -0.05(-0.21-0.1) |
| Bangladesh | Female | 368.36(237.27-526.98) | 1177.59(721.86-1831.81) | 1.87(1.16-2.78) | 2.03(1.23-3.15) | 0.21(0.09-0.34) |
| Bangladesh | Male | 542.1(360.46-792.44) | 1390.2(947.21-2145.93) | 2.24(1.48-3.25) | 2.18(1.5-3.35) | -0.24(-0.45--0.03) |
| Barbados | Both | 17.1(12.21-21.27) | 37.18(25.12-48.05) | 5.68(4.05-7.11) | 7.54(5.1-9.76) | 0.89(0.68-1.1) |
| Barbados | Female | 9.29(6.48-11.69) | 19.17(12.4-25.21) | 5.12(3.59-6.44) | 6.9(4.47-9.11) | 1.34(1.08-1.59) |
| Barbados | Male | 7.81(5.65-9.74) | 18.01(12.52-23.48) | 6.43(4.61-7.95) | 8.3(5.79-10.81) | 0.38(0.09-0.66) |
| Barbuda | Both | 2.53(1.92-3.02) | 5.37(3.83-6.68) | 4.55(3.44-5.43) | 5.8(4.17-7.19) | 0.71(0.56-0.85) |
| Barbuda | Female | 1.41(1.07-1.72) | 3.21(2.27-4.01) | 4.17(3.12-5.1) | 6.35(4.53-7.95) | 1.15(0.97-1.33) |
| Barbuda | Male | 1.12(0.85-1.35) | 2.16(1.56-2.75) | 5.01(3.81-6.01) | 5.11(3.72-6.46) | 0.2(-0.15-0.54) |
| Belarus | Both | 796.97(589.65-955.84) | 910.55(627.92-1223.56) | 6.2(4.59-7.44) | 5.67(3.9-7.61) | -1.11(-1.42--0.8) |
| Belarus | Female | 438.62(321.56-532.46) | 451.95(307.88-607.64) | 5.34(3.94-6.5) | 4.36(2.98-5.9) | -1.54(-1.88--1.2) |
| Belarus | Male | 358.35(265.2-426.83) | 458.6(314.47-624.56) | 7.8(5.72-9.37) | 8.12(5.55-10.91) | -0.62(-0.92--0.32) |
| Belgium | Both | 1225.99(864.34-1542.5) | 1382.47(975.83-1713.36) | 7.86(5.55-9.9) | 5.5(3.91-6.82) | -1.29(-1.39--1.2) |
| Belgium | Female | 650.96(459.58-829.96) | 651.26(448.47-823.3) | 6.69(4.69-8.56) | 4.31(3.01-5.42) | -1.59(-1.73--1.45) |
| Belgium | Male | 575.03(407.3-716.57) | 731.22(530.58-911.9) | 9.71(6.9-12.11) | 7.01(5.06-8.7) | -1.18(-1.26--1.11) |
| Belize | Both | 1.98(1.38-2.44) | 8.22(5.55-10.43) | 2.2(1.53-2.71) | 3.14(2.13-3.98) | 1.1(0.65-1.55) |
| Belize | Female | 1.07(0.74-1.35) | 3.47(2.32-4.44) | 2.3(1.6-2.91) | 2.7(1.81-3.44) | 0.71(0.37-1.05) |
| Belize | Male | 0.92(0.65-1.14) | 4.75(3.31-6.11) | 2.06(1.48-2.55) | 3.56(2.49-4.57) | 1.48(0.89-2.07) |
| Benin | Both | 48.31(38.38-59.04) | 122.62(89.93-162.55) | 2.59(2.06-3.17) | 2.86(2.14-3.73) | 0.42(0.38-0.46) |
| Benin | Female | 19.91(15.37-24.9) | 57.9(42.29-78.19) | 2.08(1.6-2.6) | 2.5(1.86-3.28) | 0.78(0.73-0.83) |
| Benin | Male | 28.4(21.93-35.86) | 64.72(45.49-88.27) | 3.13(2.43-3.96) | 3.28(2.32-4.38) | 0.19(0.15-0.23) |
| Bermuda | Both | 4.8(3.27-6.13) | 8.56(5.94-11.09) | 8.12(5.52-10.41) | 6.3(4.39-8.12) | -0.82(-0.88--0.76) |
| Bermuda | Female | 2.4(1.62-3.11) | 3.92(2.58-5.28) | 6.92(4.67-8.98) | 4.78(3.15-6.41) | -1.62(-1.79--1.45) |
| Bermuda | Male | 2.4(1.66-3.07) | 4.64(3.28-6.01) | 9.67(6.74-12.34) | 8.23(5.83-10.64) | -0.1(-0.29-0.1) |
| Bhutan | Both | 4.15(2.33-6.01) | 13.2(8.14-18.74) | 1.9(1.07-2.74) | 2.57(1.62-3.66) | 1.04(1.02-1.05) |
| Bhutan | Female | 2.23(1.24-3.51) | 6.48(3.95-9.71) | 1.95(1.04-2.99) | 2.59(1.6-3.88) | 0.86(0.8-0.92) |
| Bhutan | Male | 1.92(0.98-3.12) | 6.72(3.8-10.24) | 1.84(0.97-2.93) | 2.56(1.48-3.9) | 1.26(1.2-1.32) |
| Bolivia | Both | 108.98(74.58-142.28) | 398.24(260.6-548.74) | 3.73(2.61-4.82) | 4.94(3.23-6.76) | 0.9(0.86-0.94) |
| Bolivia | Female | 62.76(41.85-86.75) | 221.26(140.95-311.99) | 3.99(2.74-5.46) | 5.21(3.37-7.33) | 0.84(0.8-0.88) |
| Bolivia | Male | 46.22(30.26-62.35) | 176.98(111.39-247.31) | 3.4(2.23-4.5) | 4.62(2.86-6.43) | 1.01(0.93-1.09) |
| Bosnia and Herzegovina | Both | 167.35(118.62-204.63) | 339.9(201.51-466.71) | 4.45(3.19-5.46) | 5.76(3.42-7.89) | 1.13(0.9-1.36) |
| Bosnia and Herzegovina | Female | 84.22(58.38-105.41) | 140.21(84.31-193.57) | 3.97(2.73-4.94) | 4.18(2.51-5.77) | 0.24(0.1-0.38) |
| Bosnia and Herzegovina | Male | 83.13(61.06-101.75) | 199.7(118.16-276.79) | 5.12(3.77-6.27) | 7.86(4.71-10.81) | 1.88(1.54-2.22) |
| Botswana | Both | 18.03(12.48-24.32) | 59.75(38.23-85.27) | 3.66(2.6-4.92) | 5.04(3.3-7.03) | 0.82(0.5-1.14) |
| Botswana | Female | 8.42(5.6-11.9) | 27.99(17.15-41.99) | 3.1(2.1-4.29) | 4.18(2.66-6.14) | 1.07(0.92-1.23) |
| Botswana | Male | 9.62(6.67-12.94) | 31.75(20.62-44.36) | 4.35(3.09-5.8) | 6.22(4.16-8.41) | 0.71(0.22-1.21) |
| Brazil | Both | 2857.98(2180.07-3447.2) | 8765.51(6546.27-10782.53) | 3.53(2.7-4.24) | 3.78(2.8-4.64) | 0.27(0.11-0.43) |
| Brazil | Female | 1467.4(1101.1-1774.35) | 4280.38(3086.3-5338.97) | 3.39(2.54-4.1) | 3.3(2.37-4.11) | -0.08(-0.25-0.09) |
| Brazil | Male | 1390.58(1072.15-1664.12) | 4485.13(3324.62-5465.92) | 3.68(2.85-4.41) | 4.37(3.25-5.34) | 0.67(0.52-0.83) |
| Brunei | Both | 7.95(5.54-10.36) | 22.62(15.72-28.98) | 9.65(6.83-12.49) | 9.54(6.65-11.98) | 0.64(0.34-0.93) |
| Brunei | Female | 3.2(2.19-4.25) | 9.42(6.39-12.34) | 7.29(4.99-9.57) | 7.25(4.89-9.47) | 0.39(0.24-0.54) |
| Brunei | Male | 4.75(3.26-6.41) | 13.19(9.13-17.35) | 13.63(9.34-18.23) | 14.13(10-18.09) | 1.06(0.53-1.59) |
| Bulgaria | Both | 792.85(559.9-978.08) | 1266.15(868.6-1715.09) | 6.5(4.61-8.02) | 8.68(5.94-11.8) | 2.29(1.78-2.8) |
| Bulgaria | Female | 346.66(243.17-431.49) | 534.79(355.69-724.28) | 5.3(3.74-6.59) | 6.32(4.22-8.57) | 1.77(1.27-2.27) |
| Bulgaria | Male | 446.19(316.81-553.24) | 731.36(498.43-984.22) | 7.99(5.7-9.85) | 11.95(8.15-16.05) | 2.79(2.28-3.32) |
| Burkina Faso | Both | 91.67(69.32-119.53) | 225.02(167.65-297.79) | 2.38(1.81-3.09) | 2.82(2.14-3.66) | 0.6(0.45-0.76) |
| Burkina Faso | Female | 39.99(28.41-54.18) | 109.46(79.21-145.06) | 2.01(1.44-2.71) | 2.52(1.84-3.33) | 0.83(0.69-0.96) |
| Burkina Faso | Male | 51.68(38.57-70.58) | 115.56(86.26-160.93) | 2.78(2.08-3.78) | 3.19(2.43-4.37) | 0.46(0.27-0.65) |
| Burundi | Both | 65.14(48.55-84.62) | 117.25(78.96-172.25) | 2.93(2.2-3.78) | 2.8(1.93-4.05) | -0.32(-0.39--0.24) |
| Burundi | Female | 29.64(19.2-43.4) | 49.76(32.88-70.37) | 2.43(1.63-3.52) | 2.45(1.63-3.43) | -0.14(-0.21--0.07) |
| Burundi | Male | 35.51(26.54-45.61) | 67.5(44.42-107.29) | 3.55(2.69-4.55) | 3.15(2.09-4.85) | -0.58(-0.66--0.49) |
| Cambodia | Both | 172.67(128.27-232.92) | 649.16(480.84-816.13) | 4.05(3.05-5.47) | 5.86(4.39-7.28) | 1.29(1.25-1.33) |
| Cambodia | Female | 88.32(62.69-118.92) | 317.25(232.88-406.14) | 3.6(2.66-4.75) | 4.93(3.66-6.2) | 1.03(1-1.07) |
| Cambodia | Male | 84.34(60.24-136.7) | 331.91(245.71-417.56) | 4.65(3.31-7.42) | 7.23(5.41-8.97) | 1.6(1.55-1.66) |
| Cameroon | Both | 143.88(106.23-183.81) | 410.44(291.46-568.8) | 3.66(2.73-4.67) | 3.97(2.89-5.32) | 0.27(0.24-0.3) |
| Cameroon | Female | 60.4(44.72-78.17) | 173.67(121.66-243.88) | 3.02(2.26-3.9) | 3.26(2.32-4.52) | 0.36(0.31-0.41) |
| Cameroon | Male | 83.48(59.35-109.97) | 236.77(157.28-340.33) | 4.34(3.12-5.7) | 4.74(3.23-6.67) | 0.22(0.16-0.29) |
| Canada | Both | 2051.35(1503.66-2551.61) | 3524.77(2515.1-4451.51) | 6.38(4.68-7.93) | 4.94(3.55-6.22) | -0.88(-0.97--0.8) |
| Canada | Female | 975.28(701.39-1221.38) | 1613.33(1096.23-2096.55) | 5.26(3.81-6.57) | 4.01(2.79-5.18) | -0.92(-1.02--0.82) |
| Canada | Male | 1076.06(792.13-1324.41) | 1911.44(1362.82-2404.4) | 7.9(5.8-9.74) | 6.04(4.34-7.59) | -0.96(-1.04--0.89) |
| Central African Republic | Both | 31.52(22.35-42.06) | 50.44(33.85-73) | 2.93(2.18-3.85) | 2.52(1.78-3.57) | -0.44(-0.5--0.38) |
| Central African Republic | Female | 13.2(8.87-19.04) | 21.65(14.21-31.35) | 2.32(1.58-3.25) | 2.04(1.36-2.89) | -0.36(-0.42--0.3) |
| Central African Republic | Male | 18.32(12.43-26.59) | 28.8(18.54-45.4) | 3.66(2.63-5.18) | 3.16(2.18-4.89) | -0.46(-0.52--0.39) |
| Chad | Both | 62.36(46.09-79.9) | 155.91(115.06-209.61) | 2.36(1.76-3.03) | 3.16(2.34-4.2) | 1.14(1.07-1.2) |
| Chad | Female | 27.69(19.19-37.14) | 66.15(47.73-89.58) | 2.05(1.41-2.78) | 2.92(2.12-3.87) | 1.47(1.37-1.57) |
| Chad | Male | 34.67(24.29-47.05) | 89.76(62.85-124.11) | 2.69(1.9-3.65) | 3.33(2.37-4.52) | 0.79(0.71-0.87) |
| Chile | Both | 484.07(378.62-564.95) | 1404.05(1087.43-1674.29) | 5.29(4.15-6.16) | 5.86(4.54-6.99) | 0.69(0.57-0.81) |
| Chile | Female | 254.52(198.3-297.91) | 721(549.04-872.23) | 5.07(3.95-5.92) | 5.29(4.03-6.4) | 0.43(0.33-0.53) |
| Chile | Male | 229.55(181.94-269.47) | 683.05(531.22-808.69) | 5.54(4.4-6.5) | 6.53(5.08-7.73) | 0.95(0.79-1.11) |
| China | Both | 29656.01(23146.43-35344.01) | 90406.62(65690.67-114669.41) | 3.84(3.01-4.57) | 4.76(3.48-6.01) | 1.11(0.84-1.39) |
| China | Female | 13720.72(10438.38-17033.46) | 32549.92(22879.91-42873.01) | 3.39(2.58-4.17) | 3.23(2.27-4.25) | 0.01(-0.21-0.23) |
| China | Male | 15935.29(12164.67-19822.84) | 57856.7(41148.37-76523.23) | 4.5(3.48-5.55) | 6.75(4.82-8.82) | 1.92(1.61-2.23) |
| Colombia | Both | 543.3(417.21-643.7) | 1642.85(1082.17-2255.79) | 3.36(2.6-3.97) | 3.08(2.03-4.23) | -0.24(-0.36--0.11) |
| Colombia | Female | 302.24(228.58-360.99) | 874.98(566.72-1204.07) | 3.63(2.76-4.34) | 2.96(1.92-4.08) | -0.68(-0.78--0.57) |
| Colombia | Male | 241.06(189.61-285.31) | 767.87(521.87-1066.56) | 3.04(2.4-3.6) | 3.21(2.19-4.47) | 0.28(0.09-0.47) |
| Comoros | Both | 6.53(3.94-8.97) | 16.05(10.92-21.27) | 3.16(2.03-4.27) | 3.5(2.41-4.59) | 0.24(0.14-0.34) |
| Comoros | Female | 3.08(1.81-4.51) | 8.4(5.64-11.37) | 2.85(1.78-4.09) | 3.32(2.24-4.48) | 0.44(0.35-0.54) |
| Comoros | Male | 3.45(2.01-4.75) | 7.65(5.02-10.95) | 3.51(2.22-4.75) | 3.74(2.49-5.26) | 0.08(-0.06-0.21) |
| Costa Rica | Both | 53.81(38.92-66.33) | 236.9(151.4-322.92) | 3.19(2.3-3.94) | 4.63(2.96-6.31) | 1.43(1.3-1.56) |
| Costa Rica | Female | 27.47(19.37-34.25) | 112.49(70.31-151.97) | 3.14(2.22-3.93) | 3.99(2.5-5.4) | 0.94(0.8-1.07) |
| Costa Rica | Male | 26.34(19.39-32.31) | 124.4(80.81-171.55) | 3.24(2.39-3.98) | 5.37(3.48-7.37) | 1.93(1.77-2.1) |
| Croatia | Both | 406.16(267.95-510.41) | 647.27(411.19-879.49) | 6.67(4.4-8.36) | 7.06(4.46-9.64) | 0.44(0.26-0.63) |
| Croatia | Female | 194.36(126.3-246.09) | 256.64(157.64-353.52) | 5.25(3.42-6.65) | 4.67(2.9-6.46) | -0.09(-0.32-0.14) |
| Croatia | Male | 211.8(143.56-264.31) | 390.63(245.6-530.06) | 9.02(6.18-11.2) | 10.58(6.64-14.35) | 0.73(0.53-0.93) |
| Cuba | Both | 510.12(366.51-622.14) | 995.87(663.19-1339.85) | 5.04(3.61-6.15) | 5.09(3.4-6.86) | -0.22(-0.39--0.05) |
| Cuba | Female | 268.85(191.43-333.5) | 539.76(355.1-731.58) | 5.22(3.72-6.46) | 5.06(3.3-6.89) | -0.32(-0.44--0.2) |
| Cuba | Male | 241.28(175.01-291.98) | 456.11(312.52-611.42) | 4.84(3.51-5.86) | 5.1(3.49-6.85) | -0.13(-0.39-0.13) |
| Cyprus | Both | 33.38(23.67-42.08) | 91.25(64.38-115.84) | 4.63(3.34-5.86) | 4.94(3.47-6.29) | 0.49(0.25-0.73) |
| Cyprus | Female | 15.91(10.95-21.51) | 39.94(27.6-51.14) | 4.26(2.94-5.83) | 4.01(2.77-5.13) | 0.11(-0.08-0.29) |
| Cyprus | Male | 17.47(12.64-22.32) | 51.31(34.89-66.06) | 5.08(3.7-6.48) | 6.04(4.17-7.86) | 0.98(0.6-1.36) |
| Czech Republic | Both | 1363.85(962.11-1732.76) | 1334.4(882.56-1812.14) | 9.93(7.01-12.63) | 6.17(4.1-8.38) | -1.77(-1.95--1.58) |
| Czech Republic | Female | 594.29(408.5-767.24) | 541.07(354.32-740.23) | 7.11(4.94-9.15) | 4.27(2.8-5.81) | -1.93(-2.09--1.77) |
| Czech Republic | Male | 769.56(551.69-969.04) | 793.33(520.5-1081.17) | 14.34(10.26-18.11) | 8.72(5.74-11.86) | -1.8(-2.01--1.58) |
| Democratic Republic of the Congo | Both | 358.14(265.93-480.5) | 805.66(528.42-1209.52) | 2.63(1.93-3.67) | 2.53(1.62-3.92) | -0.27(-0.55-0) |
| Democratic Republic of the Congo | Female | 161.74(116.18-217.26) | 395.07(245.47-620.49) | 2.24(1.64-3.1) | 2.23(1.35-3.61) | -0.1(-0.34-0.14) |
| Democratic Republic of the Congo | Male | 196.39(139.17-323.86) | 410.58(246.52-770.31) | 3.06(2.12-5.23) | 2.97(1.78-5.82) | -0.31(-0.58--0.03) |
| Denmark | Both | 640.92(467.45-800.8) | 825.74(578.92-1039.42) | 7.68(5.61-9.57) | 6.8(4.79-8.56) | -0.83(-1.05--0.6) |
| Denmark | Female | 333.88(239.08-418.43) | 389.6(265.59-497.5) | 6.78(4.88-8.46) | 5.72(3.93-7.27) | -0.95(-1.16--0.73) |
| Denmark | Male | 307.04(225.74-380.81) | 436.14(309.81-547.02) | 8.94(6.55-11.1) | 8.11(5.81-10.14) | -0.8(-1.05--0.55) |
| Djibouti | Both | 4.79(3.2-6.73) | 23.24(15.51-33.45) | 3.9(2.71-5.35) | 4.41(3.17-6) | 0.37(0.32-0.42) |
| Djibouti | Female | 2.18(1.46-3.2) | 9.46(6.24-13.57) | 3.58(2.45-5.09) | 3.87(2.72-5.34) | 0.23(0.17-0.29) |
| Djibouti | Male | 2.61(1.57-3.84) | 13.78(8.74-20.26) | 4.26(2.63-6.16) | 4.93(3.3-6.93) | 0.43(0.38-0.48) |
| Dominica | Both | 2.8(1.9-3.5) | 4.03(2.53-5.36) | 3.81(2.58-4.8) | 4.43(2.8-5.91) | 0.74(0.66-0.82) |
| Dominica | Female | 1.56(1.05-1.98) | 1.97(1.23-2.61) | 3.47(2.33-4.41) | 4(2.49-5.3) | 0.82(0.7-0.94) |
| Dominica | Male | 1.24(0.84-1.58) | 2.06(1.28-2.82) | 4.27(2.92-5.44) | 4.87(3.04-6.64) | 0.54(0.47-0.6) |
| Dominican Republic | Both | 97.79(77.13-117.98) | 388.51(262.71-530.68) | 2.9(2.28-3.5) | 4.34(2.94-5.92) | 2.01(1.82-2.2) |
| Dominican Republic | Female | 50.72(39.64-62.12) | 176.69(121.02-242.47) | 3.06(2.4-3.73) | 3.79(2.61-5.19) | 1.28(1.04-1.52) |
| Dominican Republic | Male | 47.06(36.66-57.94) | 211.82(135.59-292.57) | 2.71(2.09-3.34) | 4.9(3.19-6.74) | 2.69(2.49-2.9) |
| Ecuador | Both | 131.6(101.42-156.35) | 577.02(414.86-770.81) | 2.67(2.06-3.18) | 4.09(2.97-5.43) | 2.1(1.76-2.44) |
| Ecuador | Female | 74.74(57.2-89.75) | 304.97(218.29-405.22) | 3.08(2.38-3.7) | 4.1(2.97-5.42) | 1.61(1.27-1.96) |
| Ecuador | Male | 56.86(43.95-67.82) | 272.05(193.03-362.93) | 2.3(1.78-2.74) | 4.06(2.93-5.38) | 2.61(2.26-2.97) |
| Egypt | Both | 551.49(428.99-646.15) | 1384.51(869.93-2004.15) | 1.97(1.53-2.3) | 2.29(1.49-3.24) | 0.52(0.41-0.63) |
| Egypt | Female | 267.71(207.44-317.02) | 581.09(354.91-864.95) | 1.99(1.55-2.37) | 2.38(1.45-3.55) | 0.73(0.64-0.82) |
| Egypt | Male | 283.77(221.54-337.76) | 803.42(492.49-1168.88) | 1.93(1.51-2.29) | 2.32(1.45-3.36) | 0.52(0.36-0.68) |
| El Salvador | Both | 48.6(38.3-57.29) | 165.06(108.81-225.85) | 1.67(1.31-1.98) | 2.7(1.77-3.7) | 1.47(1.19-1.75) |
| El Salvador | Female | 26.13(20.48-30.9) | 88.26(56.69-121.84) | 1.68(1.31-1.99) | 2.46(1.58-3.41) | 1.2(0.97-1.44) |
| El Salvador | Male | 22.47(17.67-26.76) | 76.8(50.79-104.57) | 1.66(1.3-1.98) | 3.02(1.99-4.12) | 1.81(1.45-2.17) |
| Equatorial Guinea | Both | 4.94(3.31-6.93) | 20.8(12.46-31.42) | 2.7(1.8-3.71) | 4.93(3.03-7.14) | 2.62(2.42-2.81) |
| Equatorial Guinea | Female | 2.14(1.23-3.3) | 10.1(5.93-16.24) | 2.11(1.19-3.21) | 4.13(2.52-6.39) | 2.92(2.6-3.25) |
| Equatorial Guinea | Male | 2.8(1.8-4.29) | 10.7(5.74-16.32) | 3.49(2.19-5.17) | 6.12(3.38-9.03) | 2.37(2.25-2.5) |
| Eritrea | Both | 24.43(16.92-33.96) | 94.88(68.24-127.43) | 2.59(1.83-3.6) | 3.85(2.85-5.09) | 1.12(0.84-1.4) |
| Eritrea | Female | 11.89(6.1-19.69) | 49.2(33.2-70.7) | 2.21(1.15-3.62) | 3.53(2.43-5.04) | 1.47(1.2-1.73) |
| Eritrea | Male | 12.54(7.91-17.31) | 45.68(30.32-62.85) | 3.2(2.01-4.34) | 4.33(2.86-5.77) | 0.71(0.41-1.01) |
| Estonia | Both | 121.72(83.99-152.38) | 149.72(96.23-204.64) | 5.97(4.11-7.47) | 5.25(3.41-7.15) | -0.44(-0.65--0.23) |
| Estonia | Female | 68.11(46.13-85.97) | 82.41(52.17-113.26) | 5.07(3.44-6.38) | 4.35(2.76-6.03) | -0.59(-0.77--0.41) |
| Estonia | Male | 53.61(37.04-66.71) | 67.3(43.13-92.31) | 7.77(5.38-9.68) | 7.01(4.53-9.62) | -0.28(-0.53--0.02) |
| Ethiopia | Both | 582.98(406.2-912.59) | 1116.4(774.1-1637.42) | 3.07(2.07-4.84) | 2.89(2.01-4.26) | -0.33(-0.57--0.09) |
| Ethiopia | Female | 249.03(137.3-370.39) | 489.07(325.48-744.52) | 2.68(1.52-3.93) | 2.57(1.73-3.87) | -0.38(-0.56--0.2) |
| Ethiopia | Male | 333.95(211.94-679.71) | 627.33(414.84-1011.21) | 3.42(2.16-6.77) | 3.21(2.15-5.18) | -0.27(-0.58-0.04) |
| Fiji | Both | 11.36(8.48-14.51) | 27.08(19.53-36) | 3.6(2.71-4.53) | 4.27(3.09-5.57) | 0.76(0.55-0.97) |
| Fiji | Female | 5.16(3.73-6.7) | 12.51(8.79-16.89) | 3.26(2.38-4.2) | 3.67(2.6-4.89) | 0.69(0.45-0.94) |
| Fiji | Male | 6.2(4.57-8) | 14.57(10.48-19.35) | 4.05(3-5.15) | 5.26(3.88-6.8) | 1.02(0.84-1.21) |
| Finland | Both | 314.92(222.23-393.34) | 465.91(315.51-591.96) | 4.39(3.1-5.48) | 3.56(2.44-4.52) | -0.8(-0.88--0.72) |
| Finland | Female | 173.8(121.07-218.66) | 222.81(142.63-289.52) | 3.77(2.65-4.72) | 2.89(1.93-3.72) | -1(-1.09--0.91) |
| Finland | Male | 141.11(100.49-174.51) | 243.09(169.6-307.35) | 5.43(3.88-6.69) | 4.41(3.09-5.57) | -0.78(-0.85--0.7) |
| France | Both | 7082.95(5299.54-8567.48) | 8885.79(6398.73-10933.7) | 8.24(6.17-9.94) | 5.76(4.18-7.05) | -1.38(-1.44--1.32) |
| France | Female | 3393.53(2477.38-4146.38) | 4205.01(2944.13-5355.51) | 6.28(4.61-7.66) | 4.39(3.12-5.49) | -1.37(-1.44--1.3) |
| France | Male | 3689.42(2774.46-4422.26) | 4680.78(3409.59-5801.12) | 11.22(8.47-13.46) | 7.56(5.5-9.29) | -1.52(-1.58--1.45) |
| Gabon | Both | 30.62(19.03-46.8) | 53.59(36.36-70.52) | 5.87(3.73-8.82) | 5.65(3.96-7.37) | -0.28(-0.35--0.21) |
| Gabon | Female | 13.46(8.13-21.93) | 21.55(15.21-29.42) | 4.63(2.87-7.46) | 4.19(3.01-5.6) | -0.51(-0.63--0.38) |
| Gabon | Male | 17.16(10.29-27.79) | 32.04(20.31-43.31) | 7.53(4.61-12.02) | 7.53(4.95-9.92) | -0.15(-0.24--0.05) |
| Gambia | Both | 6.29(4.66-8.21) | 22.72(15.55-31.37) | 2(1.5-2.58) | 2.58(1.78-3.53) | 0.67(0.52-0.82) |
| Gambia | Female | 2.87(2.1-3.72) | 11.18(7.29-16.28) | 1.9(1.41-2.45) | 2.42(1.59-3.51) | 0.65(0.5-0.81) |
| Gambia | Male | 3.43(2.48-4.66) | 11.54(7.49-16.52) | 2.06(1.53-2.71) | 2.73(1.81-3.91) | 0.75(0.59-0.91) |
| Germany | Both | 10116.32(7460.48-12661.83) | 11623.78(8407.53-14621.69) | 7.78(5.73-9.72) | 5.59(4.06-7) | -1.56(-2.18--0.94) |
| Germany | Female | 5626.27(4073.81-7070.32) | 5429.17(3713.47-6915.47) | 6.66(4.83-8.35) | 4.37(3.03-5.55) | -1.94(-2.61--1.26) |
| Germany | Male | 4490.04(3365.22-5567.25) | 6194.61(4582.19-7731.3) | 9.71(7.26-12.03) | 7.09(5.26-8.81) | -1.46(-2.02--0.89) |
| Ghana | Both | 140.49(106.81-177.11) | 450.08(316.91-592.86) | 2.54(1.97-3.16) | 3.15(2.25-4.1) | 0.72(0.69-0.75) |
| Ghana | Female | 69.22(52.23-88.11) | 202.47(146.73-263.92) | 2.42(1.84-3.09) | 2.57(1.89-3.33) | -0.01(-0.08-0.07) |
| Ghana | Male | 71.28(52.64-91.26) | 247.61(164.19-342.63) | 2.66(1.98-3.37) | 3.89(2.57-5.33) | 1.48(1.4-1.56) |
| Greece | Both | 711.45(516.63-864.49) | 1362.09(983.48-1653.31) | 4.74(3.44-5.74) | 5.1(3.66-6.18) | -0.21(-0.44-0.01) |
| Greece | Female | 362.68(258.37-443.44) | 621.03(426.86-769.45) | 4.37(3.13-5.33) | 4.08(2.85-5) | -0.81(-1.08--0.53) |
| Greece | Male | 348.76(253.18-425.5) | 741.05(544.84-912.53) | 5.17(3.79-6.31) | 6.32(4.67-7.75) | 0.33(0.15-0.51) |
| Greenland | Both | 3.01(2.18-3.81) | 6.19(4.24-8.11) | 9.6(6.93-12) | 9.78(6.83-12.82) | -0.38(-0.71--0.05) |
| Greenland | Female | 1.5(1.05-1.94) | 2.68(1.79-3.58) | 9.44(6.64-12.19) | 8.87(5.96-11.84) | -1.04(-1.55--0.53) |
| Greenland | Male | 1.51(1.07-1.92) | 3.51(2.35-4.68) | 9.86(7.15-12.42) | 10.6(7.24-13.89) | 0.25(0.08-0.43) |
| Grenada | Both | 3.8(2.8-4.55) | 6.47(4.48-7.92) | 4.99(3.65-6) | 6.3(4.39-7.72) | 0.89(0.76-1.02) |
| Grenada | Female | 1.96(1.41-2.4) | 3.07(2.1-3.83) | 4.25(3.02-5.2) | 5.43(3.72-6.78) | 0.99(0.66-1.32) |
| Grenada | Male | 1.84(1.37-2.25) | 3.4(2.37-4.17) | 6(4.45-7.35) | 7.27(5.2-8.88) | 0.77(0.25-1.29) |
| Grenadines | Both | 3.32(2.65-3.84) | 6.35(4.54-7.88) | 4.78(3.8-5.54) | 4.93(3.54-6.1) | -0.08(-0.34-0.18) |
| Grenadines | Female | 1.87(1.48-2.2) | 2.87(2.02-3.61) | 4.71(3.71-5.51) | 4.48(3.14-5.6) | -0.27(-0.6-0.06) |
| Grenadines | Male | 1.45(1.15-1.72) | 3.48(2.52-4.36) | 4.85(3.85-5.74) | 5.36(3.94-6.65) | 0.06(-0.16-0.29) |
| Guam | Both | 3.41(2.38-4.33) | 8.22(5.55-10.75) | 5.54(3.77-7.22) | 4.39(3-5.72) | -0.82(-1.21--0.42) |
| Guam | Female | 1.1(0.76-1.48) | 2.82(1.86-3.72) | 3.79(2.59-5.23) | 2.83(1.85-3.74) | -1.22(-1.6--0.84) |
| Guam | Male | 2.31(1.59-2.97) | 5.4(3.7-7.12) | 7.4(5.1-9.6) | 6.23(4.3-8.18) | -0.45(-0.95-0.04) |
| Guatemala | Both | 60.27(48.09-71.57) | 335.6(242.15-435.02) | 1.87(1.49-2.2) | 3.19(2.33-4.09) | 1.66(1.3-2.02) |
| Guatemala | Female | 31.4(24.22-38.12) | 173.08(124.52-222.77) | 2.05(1.6-2.47) | 3(2.18-3.86) | 1.01(0.7-1.32) |
| Guatemala | Male | 28.88(22.33-35.61) | 162.51(117.37-214.77) | 1.73(1.34-2.11) | 3.42(2.5-4.49) | 2.32(1.92-2.72) |
| Guinea | Both | 70.18(55.57-85.83) | 143.22(101.21-191.34) | 2.3(1.83-2.8) | 2.83(2.01-3.76) | 0.79(0.75-0.82) |
| Guinea | Female | 32.44(24.35-41.61) | 59.75(39.83-83.1) | 2.14(1.6-2.76) | 2.36(1.56-3.29) | 0.32(0.29-0.36) |
| Guinea | Male | 37.74(28.54-47.51) | 83.46(56.97-117.38) | 2.45(1.85-3.08) | 3.27(2.27-4.56) | 1.12(1.02-1.22) |
| Guinea-Bissau | Both | 14.05(9.58-18.63) | 24.04(17.16-31.68) | 3.73(2.63-4.88) | 3.76(2.73-4.87) | 0.04(0-0.09) |
| Guinea-Bissau | Female | 5.06(3.35-7.05) | 11.1(7.98-15.1) | 2.64(1.79-3.68) | 3.17(2.31-4.2) | 0.7(0.66-0.73) |
| Guinea-Bissau | Male | 8.99(5.72-12.36) | 12.95(8.75-18.2) | 4.86(3.2-6.58) | 4.46(3.09-6.22) | -0.34(-0.38--0.29) |
| Guyana | Both | 17.15(13.45-20.67) | 26.25(17.86-35.04) | 4.84(3.83-5.82) | 4.58(3.16-6.03) | -0.28(-0.42--0.13) |
| Guyana | Female | 8(6.11-9.84) | 12.2(8.12-16.31) | 4.39(3.34-5.38) | 4.02(2.7-5.33) | -0.36(-0.51--0.21) |
| Guyana | Male | 9.15(7.12-11.26) | 14.05(9.5-18.97) | 5.3(4.15-6.5) | 5.21(3.58-6.93) | -0.17(-0.34-0) |
| Haiti | Both | 122.14(80.31-157.45) | 269.71(179.36-375.54) | 4.11(2.73-5.23) | 4.27(2.88-5.84) | 0.26(0.19-0.33) |
| Haiti | Female | 65.99(40.53-90.97) | 149.28(94.43-222.71) | 4.22(2.67-5.71) | 4.47(2.85-6.55) | 0.31(0.23-0.39) |
| Haiti | Male | 56.15(37.82-78.87) | 120.42(77.62-173.76) | 3.97(2.73-5.58) | 4.04(2.6-5.73) | 0.22(0.09-0.35) |
| Honduras | Both | 33.13(24.96-41.23) | 135.36(85.1-201.74) | 1.66(1.23-2.06) | 2.43(1.55-3.64) | 1.37(1.23-1.52) |
| Honduras | Female | 18.29(13.71-23.19) | 74.16(47.62-114.23) | 1.81(1.33-2.33) | 2.56(1.68-3.85) | 1.18(0.97-1.39) |
| Honduras | Male | 14.84(9.96-19.67) | 61.2(32.02-96.75) | 1.51(1-1.99) | 2.3(1.18-3.64) | 1.7(1.46-1.94) |
| Hungary | Both | 1386.16(972.37-1730.16) | 1846.34(1250.59-2434.97) | 9.65(6.76-12.02) | 9.35(6.29-12.34) | -0.29(-0.51--0.07) |
| Hungary | Female | 659.1(451.74-831.65) | 801.04(535.66-1053.8) | 7.59(5.17-9.57) | 6.53(4.36-8.65) | -0.76(-0.99--0.53) |
| Hungary | Male | 727.05(515.71-903.52) | 1045.3(701.48-1381.54) | 12.86(9.17-15.95) | 13.64(9.16-18.01) | 0.08(-0.15-0.32) |
| Iceland | Both | 15.22(11.34-18.36) | 22.92(15.91-28.8) | 5.2(3.89-6.28) | 3.88(2.72-4.87) | -1.22(-1.4--1.04) |
| Iceland | Female | 7.48(5.47-9.24) | 9.12(6.22-11.76) | 4.59(3.37-5.65) | 2.71(1.89-3.48) | -2.17(-2.42--1.92) |
| Iceland | Male | 7.74(5.74-9.47) | 13.8(9.68-17.54) | 5.98(4.45-7.32) | 5.19(3.66-6.59) | -0.57(-0.76--0.37) |
| India | Both | 7349.3(5771.39-8965.98) | 24864.44(18528.76-31323.97) | 1.91(1.51-2.31) | 2.41(1.81-3.02) | 0.67(0.54-0.81) |
| India | Female | 3509.23(2559.62-4554.55) | 12681.13(9003.15-16540.35) | 1.86(1.36-2.43) | 2.36(1.68-3.06) | 0.64(0.47-0.81) |
| India | Male | 3840.07(2928.74-4899.16) | 12183.31(8737.45-15839.05) | 1.96(1.5-2.51) | 2.46(1.79-3.18) | 0.72(0.61-0.84) |
| Indonesia | Both | 3347.22(2431.88-4228.19) | 11599.06(7639.1-15678.87) | 3.66(2.7-4.57) | 6(3.9-8.09) | 1.72(1.6-1.83) |
| Indonesia | Female | 1760.63(1178.44-2350.89) | 5333.31(3197.08-7725.13) | 3.65(2.53-4.8) | 5.26(3.19-7.58) | 1.21(1.07-1.36) |
| Indonesia | Male | 1586.59(1185.41-2008.32) | 6265.74(4008.78-8657.87) | 3.67(2.74-4.64) | 6.86(4.3-9.35) | 2.22(2.13-2.31) |
| Iran | Both | 658.36(502.76-826.2) | 2408.22(1816.07-2839.76) | 2.89(2.19-3.63) | 3.51(2.65-4.14) | 0.9(0.71-1.08) |
| Iran | Female | 286.37(206.72-377.23) | 1047.82(778.56-1252.89) | 2.63(1.89-3.48) | 3.09(2.3-3.7) | 0.78(0.62-0.93) |
| Iran | Male | 371.99(274.45-477.84) | 1360.4(1030.27-1606.76) | 3.14(2.32-4.06) | 3.93(2.99-4.64) | 1.03(0.8-1.26) |
| Iraq | Both | 208.81(144.46-292.09) | 709.61(517.71-914.87) | 2.81(1.95-3.88) | 3.32(2.45-4.18) | 0.7(0.55-0.84) |
| Iraq | Female | 96.92(64.18-139.7) | 317.89(230.1-415.65) | 2.52(1.66-3.61) | 2.85(2.1-3.64) | 0.64(0.47-0.8) |
| Iraq | Male | 111.89(69.18-165.09) | 391.72(279.85-507.95) | 3.14(1.96-4.63) | 3.85(2.78-4.89) | 0.74(0.61-0.88) |
| Ireland | Both | 311.62(225.26-389.89) | 416.11(295-528.47) | 7.73(5.62-9.68) | 5.45(3.88-6.91) | -1.26(-1.3--1.21) |
| Ireland | Female | 138.35(98.88-173.33) | 171.46(117.45-223.41) | 6.1(4.41-7.64) | 4.1(2.83-5.32) | -1.4(-1.46--1.33) |
| Ireland | Male | 173.27(125.4-216.94) | 244.65(177.19-309.37) | 9.92(7.19-12.39) | 7.06(5.12-8.86) | -1.24(-1.29--1.18) |
| Israel | Both | 309.36(205.49-386.04) | 586.56(388.35-734.35) | 6.57(4.38-8.23) | 4.84(3.2-6.05) | -1.82(-2.11--1.52) |
| Israel | Female | 148.03(97.73-186.07) | 279.64(179.08-356.23) | 5.84(3.86-7.34) | 4.07(2.59-5.19) | -2.03(-2.33--1.72) |
| Israel | Male | 161.33(107.66-200.92) | 306.92(206.85-385.97) | 7.46(5.02-9.33) | 5.8(3.92-7.3) | -1.63(-1.93--1.33) |
| Italy | Both | 6314.14(4725.24-7510.45) | 8868.36(6523.58-10793.61) | 7.08(5.3-8.45) | 5.65(4.13-6.84) | -0.95(-1.05--0.85) |
| Italy | Female | 3072.25(2277.02-3689.92) | 4053.16(2930.38-5009.09) | 5.83(4.32-7) | 4.34(3.16-5.32) | -1.21(-1.28--1.13) |
| Italy | Male | 3241.89(2442-3836.45) | 4815.21(3576.06-5821.86) | 8.92(6.72-10.56) | 7.35(5.49-8.88) | -0.81(-0.94--0.68) |
| Ivory Coast | Both | 124(89.18-166.82) | 333.6(241.93-437.48) | 3.63(2.69-4.82) | 3.73(2.79-4.74) | -0.14(-0.25--0.03) |
| Ivory Coast | Female | 40.97(29.99-55.21) | 132.25(95.34-174.22) | 2.68(1.98-3.56) | 3.11(2.33-4.03) | 0.59(0.52-0.66) |
| Ivory Coast | Male | 83.03(58.16-115.21) | 201.35(140.29-267.62) | 4.46(3.18-6.17) | 4.3(3.08-5.61) | -0.53(-0.7--0.36) |
| Jamaica | Both | 62.62(44.84-76.43) | 155.91(105.43-205.13) | 3.45(2.47-4.2) | 5.14(3.46-6.77) | 1.59(1.29-1.88) |
| Jamaica | Female | 32.34(22.61-39.74) | 72.24(46.24-96.55) | 3.23(2.24-3.97) | 4.38(2.82-5.88) | 1.01(0.74-1.28) |
| Jamaica | Male | 30.28(22.11-36.42) | 83.67(57.16-111.64) | 3.68(2.69-4.43) | 5.93(4.06-7.89) | 2.06(1.64-2.48) |
| Japan | Both | 9692.88(7087.08-11880.61) | 20828.65(14658.36-26068.14) | 5.91(4.32-7.24) | 5.24(3.8-6.46) | -0.4(-0.47--0.34) |
| Japan | Female | 4417.68(3206.11-5453.39) | 10010.18(6684.9-12775.48) | 4.67(3.39-5.77) | 4(2.81-5) | -0.51(-0.56--0.45) |
| Japan | Male | 5275.19(3873.13-6410.91) | 10818.47(7803.37-13259.07) | 7.64(5.62-9.28) | 6.69(4.89-8.13) | -0.47(-0.55--0.39) |
| Jordan | Both | 59.88(44.07-76.86) | 276.92(197.15-354.47) | 4.92(3.55-6.27) | 4.86(3.48-6.12) | 0.02(-0.07-0.1) |
| Jordan | Female | 30.01(21.4-40.19) | 116.36(80.54-155.49) | 4.83(3.45-6.44) | 4.32(3.03-5.71) | -0.41(-0.53--0.29) |
| Jordan | Male | 29.87(21.43-39.66) | 160.55(112.49-215.26) | 5.04(3.59-6.58) | 5.36(3.78-7.06) | 0.34(0.15-0.53) |
| Kazakhstan | Both | 692.81(518.65-832.09) | 746.61(533.66-946.58) | 5.57(4.16-6.72) | 4.59(3.28-5.8) | -0.5(-0.63--0.37) |
| Kazakhstan | Female | 367.99(272.96-446.71) | 378.75(274.07-480.59) | 4.75(3.52-5.77) | 3.83(2.78-4.84) | -0.66(-0.79--0.53) |
| Kazakhstan | Male | 324.82(243.21-392.15) | 367.87(263.82-465.86) | 7.13(5.32-8.6) | 5.93(4.22-7.53) | -0.42(-0.57--0.27) |
| Kenya | Both | 127.48(82.17-170.75) | 482.99(323.12-659.3) | 1.69(1.1-2.28) | 2.45(1.67-3.28) | 1.55(1.38-1.72) |
| Kenya | Female | 68.92(41.53-100.39) | 249.55(154.44-375.92) | 1.77(1.07-2.56) | 2.35(1.47-3.48) | 1.31(1.11-1.52) |
| Kenya | Male | 58.56(34.28-84.12) | 233.45(155.14-314.16) | 1.61(0.95-2.31) | 2.58(1.75-3.4) | 1.83(1.61-2.05) |
| Kiribati | Both | 1.59(1.2-2) | 2.7(1.89-3.66) | 4.56(3.49-5.69) | 4.42(3.21-5.91) | -0.29(-0.36--0.23) |
| Kiribati | Female | 0.6(0.44-0.78) | 1.08(0.75-1.48) | 3.24(2.35-4.13) | 3.3(2.34-4.46) | -0.13(-0.21--0.06) |
| Kiribati | Male | 1(0.73-1.29) | 1.62(1.12-2.25) | 6.26(4.72-7.95) | 6.01(4.27-8.24) | -0.3(-0.36--0.24) |
| Kuwait | Both | 11.76(8.1-15.05) | 62.02(40.02-82.61) | 2.19(1.49-2.81) | 2.82(1.81-3.78) | 1.68(1.26-2.09) |
| Kuwait | Female | 5.43(3.52-7.25) | 19.39(12.29-27.36) | 2.51(1.64-3.35) | 2.1(1.28-2.94) | 0.38(-0.16-0.93) |
| Kuwait | Male | 6.34(4.27-8.07) | 42.64(26.91-58.45) | 1.95(1.33-2.51) | 3.3(2.1-4.53) | 2.43(2.03-2.83) |
| Kyrgyzstan | Both | 119.77(87.33-147.28) | 113.15(78.92-142.78) | 3.97(2.9-4.89) | 2.7(1.9-3.42) | -1.5(-1.71--1.28) |
| Kyrgyzstan | Female | 64.58(46.58-80.48) | 60.04(41.41-76.68) | 3.56(2.57-4.43) | 2.49(1.73-3.2) | -1.24(-1.44--1.05) |
| Kyrgyzstan | Male | 55.19(40.37-68.15) | 53.12(37.34-67.22) | 4.64(3.37-5.73) | 2.94(2.08-3.73) | -1.92(-2.2--1.63) |
| Laos | Both | 89.52(58.81-124.94) | 219.71(153.09-299.59) | 4.48(3.06-6.17) | 5.41(3.75-7.23) | 0.46(0.39-0.54) |
| Laos | Female | 44.19(27.11-66.7) | 101.18(67.36-144.98) | 4.19(2.67-6.28) | 4.8(3.26-6.74) | 0.24(0.14-0.33) |
| Laos | Male | 45.34(29.33-71.22) | 118.54(78.88-161.8) | 4.84(3.2-7.61) | 6.1(4.11-8.24) | 0.66(0.6-0.71) |
| Latvia | Both | 220.36(159.3-271.91) | 229.8(154.13-304.28) | 6.16(4.45-7.6) | 5.42(3.61-7.19) | -0.53(-0.79--0.27) |
| Latvia | Female | 123.57(86.74-153.72) | 125.63(82.82-173.71) | 5.3(3.73-6.59) | 4.46(2.95-6.28) | -0.71(-0.92--0.5) |
| Latvia | Male | 96.79(69.7-119) | 104.17(66.08-140.12) | 7.89(5.7-9.71) | 7.3(4.68-9.81) | -0.31(-0.66-0.03) |
| Lebanon | Both | 86.32(56.94-114.71) | 285.26(184.59-390.29) | 4.26(2.84-5.68) | 5.5(3.57-7.55) | 1.46(1.24-1.67) |
| Lebanon | Female | 43.09(28.21-60.29) | 138.02(86.33-205.12) | 4.1(2.7-5.65) | 4.83(3.03-7.15) | 0.93(0.76-1.09) |
| Lebanon | Male | 43.24(27.9-59.54) | 147.24(94.26-205.29) | 4.47(2.91-6.16) | 6.32(4.05-8.82) | 1.98(1.68-2.29) |
| Lesotho | Both | 22.37(16.26-32.73) | 50.22(34.68-69.43) | 2.53(1.85-3.75) | 4.43(3.12-6.06) | 2.34(2.15-2.52) |
| Lesotho | Female | 10.89(7.48-16.45) | 24.49(15.3-35.44) | 2.1(1.44-3.17) | 3.7(2.36-5.28) | 2.63(2.35-2.9) |
| Lesotho | Male | 11.48(8.15-17.51) | 25.73(17.79-37.02) | 3.1(2.23-4.7) | 5.46(3.86-7.77) | 2.1(1.92-2.29) |
| Liberia | Both | 28.57(22.04-35.57) | 50.98(33.73-74.14) | 2.8(2.18-3.48) | 2.86(1.89-4.2) | 0.5(0.18-0.83) |
| Liberia | Female | 11.17(8.6-14) | 23.72(14.88-34.7) | 2.41(1.87-3) | 2.71(1.68-3.93) | 0.9(0.59-1.2) |
| Liberia | Male | 17.41(12.81-22.74) | 27.26(16.52-43.59) | 3.13(2.33-4.06) | 2.99(1.85-4.81) | 0.25(-0.09-0.59) |
| Libya | Both | 71.86(46.31-106.3) | 211.63(134.33-293.93) | 4.01(2.59-5.88) | 4.28(2.73-5.86) | 0.3(0.19-0.42) |
| Libya | Female | 35.81(21.47-54.98) | 105.76(65.17-154.35) | 4.18(2.55-6.4) | 4.23(2.62-6.17) | 0.2(0.01-0.4) |
| Libya | Male | 36.05(19.95-55.06) | 105.88(64.88-154.19) | 3.89(2.15-5.93) | 4.32(2.68-6.21) | 0.37(0.24-0.49) |
| Lithuania | Both | 265.24(190.59-323.99) | 321.91(208.99-420.97) | 5.91(4.25-7.21) | 5.32(3.45-7) | -0.36(-0.56--0.15) |
| Lithuania | Female | 139.04(99.59-172.95) | 163.07(107.39-214.99) | 4.99(3.58-6.2) | 4.11(2.68-5.52) | -0.73(-0.9--0.56) |
| Lithuania | Male | 126.2(92.12-154.74) | 158.84(106.49-207.93) | 7.49(5.49-9.16) | 7.57(5.09-9.89) | 0.09(-0.17-0.35) |
| Luxembourg | Both | 47.55(34.45-58.52) | 53.76(38-68.74) | 8.74(6.4-10.76) | 5.07(3.62-6.47) | -2.06(-2.27--1.85) |
| Luxembourg | Female | 24.23(17.5-30.38) | 25.1(17.05-32.31) | 7.22(5.21-9) | 4.04(2.79-5.19) | -2.18(-2.37--2) |
| Luxembourg | Male | 23.33(17.15-28.78) | 28.66(20.75-36.94) | 11.15(8.15-13.71) | 6.36(4.6-8.19) | -2.07(-2.33--1.81) |
| Macedonia | Both | 89.64(63.01-109.15) | 208.17(132.51-285.53) | 5.02(3.54-6.14) | 6.77(4.33-9.21) | 1.06(0.77-1.36) |
| Macedonia | Female | 38.8(26.57-48.62) | 85.79(54.11-116.98) | 4.17(2.86-5.21) | 5.28(3.34-7.2) | 0.95(0.73-1.16) |
| Macedonia | Male | 50.83(36.22-62.54) | 122.39(77.28-166.39) | 5.96(4.27-7.31) | 8.49(5.37-11.46) | 1.19(0.81-1.57) |
| Madagascar | Both | 123.35(93.4-155.23) | 272.74(187.18-382.94) | 2.57(1.97-3.21) | 2.74(1.9-3.79) | 0.12(0.04-0.21) |
| Madagascar | Female | 55.98(39.45-75.21) | 135.92(89.96-198.68) | 2.31(1.67-3.03) | 2.58(1.75-3.76) | 0.23(0.13-0.32) |
| Madagascar | Male | 67.37(49.64-88.39) | 136.81(87.69-202.02) | 2.81(2.07-3.64) | 2.94(1.94-4.24) | 0.1(0.01-0.18) |
| Malawi | Both | 63.28(48.98-77.86) | 153.27(110.25-197.13) | 1.84(1.42-2.25) | 2.33(1.71-2.97) | 0.92(0.77-1.07) |
| Malawi | Female | 30.23(22.9-38.44) | 71.75(49.88-95.97) | 1.67(1.27-2.1) | 1.95(1.36-2.61) | 0.71(0.62-0.8) |
| Malawi | Male | 33.04(24.9-41.74) | 81.51(55.11-109.67) | 2.04(1.53-2.55) | 2.82(1.95-3.74) | 1.2(0.96-1.44) |
| Malaysia | Both | 563.92(448.05-680.76) | 1857.23(1369.56-2407.94) | 6.74(5.28-8.23) | 7.84(5.82-10.13) | -0.03(-0.25-0.19) |
| Malaysia | Female | 278.22(214.79-344.82) | 814.06(592.9-1072.38) | 6.53(4.87-8.23) | 7.01(5.11-9.23) | -0.15(-0.32-0.03) |
| Malaysia | Male | 285.7(226.22-352.06) | 1043.17(755.68-1363.88) | 6.99(5.48-8.73) | 8.67(6.33-11.31) | 0.04(-0.23-0.31) |
| Maldives | Both | 2.85(1.93-3.73) | 8.17(5.74-10.31) | 3.7(2.68-4.79) | 3.04(2.15-3.85) | -1.21(-1.41--1.01) |
| Maldives | Female | 1.38(0.83-2.03) | 3.91(2.72-5) | 4.63(3.19-6.43) | 3.31(2.34-4.25) | -1.74(-1.97--1.51) |
| Maldives | Male | 1.47(0.98-2.26) | 4.26(2.97-5.44) | 3.17(2.22-4.74) | 2.78(1.96-3.55) | -0.95(-1.14--0.77) |
| Mali | Both | 97.95(75.77-121.97) | 203.6(142.07-274.74) | 2.6(2.03-3.22) | 2.64(1.85-3.5) | -0.07(-0.17-0.02) |
| Mali | Female | 38.32(29.3-48.01) | 74.14(48.37-104.13) | 2.01(1.55-2.51) | 1.98(1.31-2.73) | -0.28(-0.38--0.19) |
| Mali | Male | 59.63(44.82-75.49) | 129.45(85.63-179.01) | 3.23(2.45-4.09) | 3.26(2.18-4.47) | -0.02(-0.15-0.11) |
| Malta | Both | 25.13(18.27-30.67) | 46.92(33.05-59.2) | 6.05(4.45-7.38) | 4.84(3.39-6.12) | -0.63(-0.75--0.51) |
| Malta | Female | 11.9(8.63-14.75) | 19.8(13.36-25.42) | 5.01(3.64-6.22) | 3.61(2.44-4.62) | -1.14(-1.27--1) |
| Malta | Male | 13.23(9.77-16.21) | 27.12(19.19-34.25) | 7.52(5.53-9.21) | 6.41(4.53-8.07) | -0.3(-0.48--0.13) |
| Marshall Islands | Both | 0.65(0.51-0.81) | 1.44(1.02-1.95) | 4.33(3.39-5.42) | 4.79(3.47-6.23) | 0.4(0.34-0.46) |
| Marshall Islands | Female | 0.3(0.22-0.39) | 0.67(0.45-0.95) | 3.97(2.92-5.11) | 4.69(3.26-6.45) | 0.51(0.44-0.59) |
| Marshall Islands | Male | 0.35(0.26-0.45) | 0.78(0.54-1.07) | 4.74(3.59-6.07) | 4.9(3.53-6.62) | 0.28(0.11-0.44) |
| Mauritania | Both | 24.86(16.2-33.61) | 47.87(30.67-66.37) | 2.7(1.77-3.62) | 2.59(1.68-3.58) | -0.06(-0.2-0.07) |
| Mauritania | Female | 11.46(7.13-16.13) | 23.53(14.31-33.48) | 2.35(1.48-3.28) | 2.59(1.6-3.64) | 0.49(0.41-0.57) |
| Mauritania | Male | 13.4(8.53-18.16) | 24.34(15.64-34.38) | 3.11(2.02-4.16) | 2.59(1.68-3.62) | -0.61(-0.83--0.39) |
| Mauritius | Both | 19.99(15.33-23.78) | 63.17(41.32-83.2) | 2.95(2.27-3.5) | 3.78(2.49-4.96) | 0.83(0.72-0.94) |
| Mauritius | Female | 9.91(7.39-11.93) | 28.2(17.83-37.29) | 2.63(1.97-3.17) | 3.03(1.92-4) | 0.52(0.35-0.69) |
| Mauritius | Male | 10.08(7.77-12.13) | 34.97(23-46.52) | 3.35(2.6-4.02) | 4.77(3.16-6.23) | 1.07(0.84-1.3) |
| Mexico | Both | 796.03(596.24-975.4) | 3111.61(2137-4057.65) | 2.02(1.51-2.47) | 2.74(1.88-3.56) | 1.15(1.02-1.28) |
| Mexico | Female | 404.51(297.67-503.36) | 1392.33(940.66-1879.64) | 2.03(1.51-2.53) | 2.29(1.55-3.1) | 0.54(0.42-0.66) |
| Mexico | Male | 391.53(300.37-476.23) | 1719.28(1188.91-2307.07) | 2.01(1.55-2.45) | 3.25(2.25-4.34) | 1.73(1.58-1.87) |
| Micronesia | Both | 1.92(1.42-2.54) | 3.15(2.08-4.34) | 4.5(3.37-5.85) | 5.12(3.53-6.81) | 0.38(0.35-0.42) |
| Micronesia | Female | 0.91(0.64-1.23) | 1.5(0.99-2.14) | 4.26(3.02-5.7) | 4.75(3.21-6.56) | 0.32(0.28-0.37) |
| Micronesia | Male | 1.01(0.72-1.39) | 1.65(1.01-2.46) | 4.73(3.52-6.33) | 5.49(3.61-7.89) | 0.43(0.4-0.45) |
| Moldova | Both | 247.8(172.8-307.23) | 312.85(218.48-404.8) | 5.63(3.92-6.99) | 5.37(3.75-6.93) | 0.52(0.02-1.04) |
| Moldova | Female | 121.19(84.15-152.54) | 131.11(89.4-168.54) | 4.65(3.23-5.87) | 3.76(2.57-4.82) | -0.06(-0.56-0.44) |
| Moldova | Male | 126.61(88.02-155.33) | 181.74(124.78-231.84) | 7.13(4.99-8.75) | 7.7(5.29-9.8) | 0.95(0.43-1.47) |
| Mongolia | Both | 44.33(34.32-54.97) | 86.39(62.69-115.37) | 4.38(3.41-5.39) | 4.17(3.09-5.46) | -0.68(-0.85--0.5) |
| Mongolia | Female | 21.36(16.37-26.85) | 39.14(27.85-52.76) | 3.83(2.94-4.8) | 3.44(2.49-4.61) | -0.79(-0.95--0.63) |
| Mongolia | Male | 22.96(17.15-29.64) | 47.25(33.77-64.01) | 5.11(3.85-6.52) | 5.22(3.8-6.89) | -0.54(-0.73--0.34) |
| Montenegro | Both | 23.53(15.13-30.83) | 45.88(27.74-62.1) | 3.9(2.52-5.11) | 4.71(2.85-6.35) | 0.89(0.83-0.96) |
| Montenegro | Female | 9.87(6.29-13.13) | 18.45(11.39-24.91) | 2.88(1.84-3.84) | 3.39(2.07-4.58) | 0.84(0.72-0.96) |
| Montenegro | Male | 13.65(8.78-18.1) | 27.43(16.57-38.29) | 5.3(3.41-7.07) | 6.44(3.9-8.98) | 0.87(0.77-0.98) |
| Morocco | Both | 327.65(245.97-401.13) | 913.25(617.65-1236.74) | 2.56(1.91-3.12) | 3.14(2.1-4.2) | 0.6(0.44-0.76) |
| Morocco | Female | 169.27(124.92-214.24) | 441.99(300.81-604.78) | 2.58(1.89-3.28) | 2.94(2.02-3.95) | 0.5(0.45-0.54) |
| Morocco | Male | 158.38(114.02-206.8) | 471.25(296.89-659.97) | 2.54(1.82-3.28) | 3.35(2.08-4.68) | 0.7(0.39-1.02) |
| Mozambique | Both | 113.15(88.42-139.33) | 336.11(238.05-449.1) | 2.2(1.73-2.7) | 3.52(2.48-4.66) | 2.02(1.87-2.16) |
| Mozambique | Female | 57.29(42.5-74.68) | 159.37(106.74-227.94) | 2.09(1.57-2.68) | 2.97(2-4.16) | 1.51(1.32-1.69) |
| Mozambique | Male | 55.86(42.77-71.51) | 176.74(120.56-243.67) | 2.32(1.8-2.93) | 4.26(2.94-5.66) | 2.59(2.43-2.76) |
| Myanmar | Both | 878.63(649.54-1234.46) | 2205.67(1558.5-2907.07) | 3.97(2.99-5.59) | 5.09(3.6-6.68) | 0.92(0.83-1.01) |
| Myanmar | Female | 444.62(324.65-584.71) | 1057.96(730.92-1448.01) | 3.75(2.79-4.84) | 4.33(3.01-5.9) | 0.5(0.36-0.63) |
| Myanmar | Male | 434.02(306.09-755.01) | 1147.71(780.96-1516.8) | 4.24(3.04-7.37) | 6.1(4.13-8) | 1.39(1.32-1.45) |
| Namibia | Both | 16.12(12.04-20.61) | 43.44(32.38-56.69) | 2.42(1.84-3.06) | 3.36(2.53-4.32) | 1.25(1.16-1.34) |
| Namibia | Female | 8.05(5.85-10.63) | 21.89(15.21-29.77) | 2.22(1.63-2.89) | 2.9(2.03-3.89) | 1.04(0.97-1.12) |
| Namibia | Male | 8.07(5.71-10.59) | 21.55(16.16-27.77) | 2.66(1.92-3.46) | 3.97(3.05-5.06) | 1.49(1.37-1.62) |
| Nepal | Both | 126.3(80.97-180.34) | 397.97(275.74-572.96) | 1.47(0.93-2.14) | 1.99(1.39-2.85) | 1.05(0.83-1.28) |
| Nepal | Female | 66.16(39.7-97.62) | 208.77(137.91-301.63) | 1.55(0.89-2.31) | 1.99(1.32-2.91) | 0.85(0.66-1.04) |
| Nepal | Male | 60.14(36.63-98.45) | 189.2(127.81-309.05) | 1.4(0.86-2.25) | 1.99(1.36-3.29) | 1.26(1-1.51) |
| Netherlands | Both | 1361.87(957.65-1704.04) | 2318.52(1627.31-2921.78) | 6.75(4.77-8.44) | 6.5(4.58-8.15) | -0.13(-0.25--0.01) |
| Netherlands | Female | 694.87(479.15-877.85) | 1091.97(748.43-1397.9) | 5.71(3.96-7.17) | 5.38(3.67-6.83) | -0.21(-0.31--0.1) |
| Netherlands | Male | 667(476.04-832.95) | 1226.55(873.09-1531.19) | 8.39(6.01-10.5) | 7.86(5.61-9.82) | -0.22(-0.36--0.08) |
| New Zealand | Both | 401.72(301.8-486.53) | 633.78(478.75-769.12) | 10.4(7.83-12.58) | 7.8(5.91-9.47) | -1.06(-1.14--0.98) |
| New Zealand | Female | 202.06(148.35-247.73) | 296.46(217.07-366.05) | 9.24(6.84-11.3) | 6.64(4.9-8.21) | -1.09(-1.17--1) |
| New Zealand | Male | 199.67(151.98-241.36) | 337.32(257.83-409.96) | 11.89(9.06-14.39) | 9.15(7.03-11.13) | -1.09(-1.18--1) |
| Nicaragua | Both | 34.01(26.04-42.06) | 143.73(106.72-180.33) | 2.37(1.78-3.02) | 3.66(2.71-4.53) | 1.52(1.27-1.77) |
| Nicaragua | Female | 17.69(12.75-23.61) | 73.15(53.96-91.35) | 2.23(1.58-3.06) | 3.37(2.51-4.19) | 1.36(0.99-1.73) |
| Nicaragua | Male | 16.33(12.65-20.28) | 70.58(51.02-92.01) | 2.58(2-3.22) | 3.99(2.92-5.09) | 1.65(1.45-1.85) |
| Niger | Both | 52.46(38.09-71.43) | 131(91.06-181.2) | 2.16(1.58-2.9) | 2.01(1.42-2.75) | -0.26(-0.4--0.13) |
| Niger | Female | 21.36(14.15-29.94) | 63.98(42.44-90.03) | 1.84(1.21-2.56) | 1.94(1.28-2.69) | 0.08(-0.01-0.18) |
| Niger | Male | 31.09(20.77-45.52) | 67.03(42.81-101.87) | 2.44(1.68-3.53) | 2.09(1.37-3.15) | -0.5(-0.68--0.33) |
| Nigeria | Both | 1006.23(710.33-1394.13) | 2424.77(1751.19-3203.51) | 2.6(1.86-3.53) | 3.3(2.44-4.31) | 1.19(1.01-1.37) |
| Nigeria | Female | 506.22(335.4-741.69) | 1262.32(864.94-1742.17) | 2.59(1.73-3.75) | 3.34(2.34-4.5) | 1.29(1.08-1.5) |
| Nigeria | Male | 500.01(306.72-746.96) | 1162.46(779.21-1676.12) | 2.57(1.62-3.75) | 3.26(2.27-4.6) | 1.19(1.02-1.37) |
| North Korea | Both | 605.02(435.05-817.74) | 1354.22(966.2-1786.3) | 3.94(2.9-5.18) | 4.28(3.01-5.61) | 0.42(0.34-0.49) |
| North Korea | Female | 321.77(222.16-441.49) | 652.27(441.12-907.31) | 3.47(2.44-4.67) | 3.46(2.34-4.83) | 0.11(0.04-0.18) |
| North Korea | Male | 283.25(198.49-399.45) | 701.94(485.04-1004) | 4.79(3.48-6.52) | 5.51(3.83-7.77) | 0.64(0.56-0.72) |
| Northern Mariana Islands | Both | 0.98(0.67-1.3) | 2.72(1.98-3.46) | 6.42(4.43-8.28) | 6.2(4.54-7.77) | -0.05(-0.2-0.1) |
| Northern Mariana Islands | Female | 0.34(0.22-0.48) | 0.79(0.56-1.04) | 5.68(3.73-7.87) | 3.81(2.69-5.04) | -1.53(-1.69--1.37) |
| Northern Mariana Islands | Male | 0.63(0.44-0.85) | 1.93(1.38-2.44) | 6.99(4.85-8.95) | 8.68(6.38-10.94) | 0.94(0.75-1.13) |
| Norway | Both | 535.56(386.19-659.61) | 677.99(491.69-839.48) | 7.55(5.47-9.3) | 6.61(4.81-8.17) | -0.43(-0.56--0.3) |
| Norway | Female | 264.36(185.45-329.75) | 331.59(235.86-416.44) | 6.31(4.47-7.89) | 5.72(4.08-7.14) | -0.32(-0.46--0.18) |
| Norway | Male | 271.2(198.2-331.94) | 346.41(254.54-424.56) | 9.31(6.82-11.42) | 7.71(5.68-9.46) | -0.63(-0.77--0.49) |
| Oman | Both | 15.17(9.46-21.61) | 37.27(23.39-51.33) | 2.67(1.68-3.76) | 2.9(1.9-3.86) | 0.64(0.51-0.78) |
| Oman | Female | 7.3(4.29-10.61) | 16.35(9.81-23.05) | 2.78(1.66-4) | 2.89(1.78-4.04) | 0.43(0.32-0.54) |
| Oman | Male | 7.87(4.68-11.77) | 20.92(12.66-30.17) | 2.59(1.56-3.74) | 3(1.91-4.04) | 0.87(0.72-1.03) |
| Pakistan | Both | 1260.21(996.46-1522.51) | 3347.98(2405-4339.24) | 2.32(1.83-2.81) | 3.29(2.35-4.23) | 1.15(0.91-1.38) |
| Pakistan | Female | 490.54(365.19-615.68) | 1376.42(965.35-1908.42) | 2(1.5-2.53) | 2.78(1.96-3.78) | 1.07(0.86-1.27) |
| Pakistan | Male | 769.66(595.27-949.19) | 1971.55(1307.53-2748.03) | 2.57(1.98-3.2) | 3.77(2.56-5.23) | 1.28(1.02-1.53) |
| Palestine | Both | 53.24(36.13-73.67) | 181.65(143.99-220.82) | 6.49(4.41-8.99) | 8.56(6.8-10.32) | 1.13(0.89-1.38) |
| Palestine | Female | 26.92(17.65-37.63) | 85.87(67.56-106.44) | 5.86(3.86-8.08) | 7.48(5.87-9.29) | 1.07(0.81-1.33) |
| Palestine | Male | 26.31(16.46-39.36) | 95.78(74.73-117.15) | 7.29(4.59-10.78) | 10.2(7.93-12.43) | 1.31(1.09-1.54) |
| Panama | Both | 50.13(40.77-58.73) | 153.07(106.21-205.9) | 3.46(2.81-4.07) | 3.66(2.54-4.94) | 0.34(0.24-0.43) |
| Panama | Female | 24.23(19.45-29.08) | 72.99(50.39-99.66) | 3.34(2.68-4.03) | 3.3(2.27-4.51) | 0.07(-0.05-0.2) |
| Panama | Male | 25.9(20.83-30.42) | 80.08(55.51-108.48) | 3.57(2.9-4.21) | 4.05(2.82-5.48) | 0.59(0.48-0.7) |
| Papua New Guinea | Both | 41.57(26.97-57.95) | 126.68(87.33-175.66) | 2.52(1.63-3.49) | 3.01(2.12-4.09) | 0.56(0.53-0.59) |
| Papua New Guinea | Female | 18.86(11.28-26.94) | 56.24(38.25-81.08) | 2.37(1.41-3.39) | 2.85(1.94-3.99) | 0.6(0.56-0.63) |
| Papua New Guinea | Male | 22.7(14.07-34.13) | 70.44(47.86-100.17) | 2.69(1.7-3.98) | 3.16(2.19-4.47) | 0.5(0.47-0.53) |
| Paraguay | Both | 50.33(39.65-61.3) | 218.46(143.65-296.58) | 2.38(1.87-2.9) | 4.08(2.7-5.52) | 2.15(1.97-2.33) |
| Paraguay | Female | 26.47(20.19-32.78) | 99.52(65.67-135.14) | 2.34(1.79-2.9) | 3.47(2.29-4.71) | 1.7(1.54-1.87) |
| Paraguay | Male | 23.85(18.59-29.26) | 118.94(79.56-161.72) | 2.4(1.87-2.95) | 4.74(3.17-6.48) | 2.58(2.37-2.8) |
| Peru | Both | 323.65(250.35-398.29) | 922.55(615.28-1273.08) | 2.87(2.22-3.54) | 2.87(1.91-3.97) | 0.03(-0.2-0.25) |
| Peru | Female | 164.03(124.65-203.48) | 480.49(310.34-659.8) | 2.84(2.15-3.53) | 2.84(1.83-3.92) | -0.21(-0.47-0.05) |
| Peru | Male | 159.62(123.02-200.28) | 442.07(291.24-630.75) | 2.88(2.24-3.61) | 2.89(1.91-4.12) | 0.3(0.09-0.51) |
| Philippines | Both | 1598.53(1295.6-1893.7) | 4671.64(3592.11-5907.61) | 5.65(4.59-6.7) | 6.24(4.84-7.83) | 0.19(-0.09-0.46) |
| Philippines | Female | 723.41(578.38-878.4) | 1981.98(1437.01-2633.25) | 5.24(4.22-6.29) | 5.06(3.7-6.68) | -0.24(-0.55-0.07) |
| Philippines | Male | 875.12(694.26-1072.52) | 2689.65(1922.96-3629.42) | 6.09(4.86-7.5) | 7.61(5.5-10.2) | 0.61(0.36-0.86) |
| Poland | Both | 2866.87(2009.95-3548.71) | 5347.68(3596.56-6923.86) | 6.7(4.7-8.27) | 7.46(5.01-9.7) | 0.26(0.09-0.42) |
| Poland | Female | 1446.03(993.47-1795.61) | 2353.3(1572.68-3170.66) | 5.58(3.84-6.92) | 5.37(3.6-7.24) | -0.34(-0.5--0.19) |
| Poland | Male | 1420.84(997.73-1742.83) | 2994.38(1941.29-4068.89) | 8.38(5.9-10.31) | 10.5(6.84-14.23) | 0.76(0.56-0.96) |
| Portugal | Both | 918.62(637.17-1143.88) | 1692.26(1188.04-2095.37) | 6.89(4.82-8.56) | 6.59(4.69-8.13) | -0.14(-0.36-0.08) |
| Portugal | Female | 444.24(305.77-564.18) | 713.85(486.04-910.79) | 5.68(3.93-7.19) | 4.65(3.24-5.85) | -0.72(-0.9--0.53) |
| Portugal | Male | 474.38(336.83-589.15) | 978.41(695.79-1208.05) | 8.57(6.06-10.61) | 9.23(6.54-11.37) | 0.28(0.02-0.53) |
| Puerto Rico | Both | 155.61(111.15-190.12) | 340.83(215.85-462.63) | 4.38(3.14-5.32) | 4.61(2.93-6.3) | 0.06(-0.13-0.27) |
| Puerto Rico | Female | 70.25(48.87-87.36) | 154.77(94.98-210.48) | 3.65(2.53-4.53) | 3.57(2.16-4.86) | -0.29(-0.46--0.11) |
| Puerto Rico | Male | 85.36(61.89-103.41) | 186.06(118.9-259.65) | 5.21(3.78-6.33) | 5.91(3.77-8.28) | 0.38(0.14-0.63) |
| Qatar | Both | 3.17(2.04-4.41) | 21.57(11.93-30.76) | 3.95(2.48-5.55) | 4.38(2.55-5.99) | 0.81(0.53-1.1) |
| Qatar | Female | 1.59(0.96-2.27) | 8.04(4.6-11.35) | 5.1(2.93-7.35) | 6.56(3.69-9.04) | 1.49(1.14-1.84) |
| Qatar | Male | 1.57(0.96-2.35) | 13.53(7.55-20.04) | 3.07(1.88-4.52) | 3.73(2.18-5.23) | 1.15(0.77-1.53) |
| Republic of Congo | Both | 47.52(30.58-65.53) | 109.14(75.52-147.72) | 4.88(3.34-6.56) | 4.7(3.38-6.15) | -0.21(-0.41--0.02) |
| Republic of Congo | Female | 21.53(13.05-32.1) | 51.3(33.95-74.37) | 4.02(2.55-5.81) | 4.19(2.89-5.89) | 0.19(0.06-0.32) |
| Republic of Congo | Male | 25.99(16.97-36.76) | 57.85(39.59-79.25) | 6.01(4.13-8.29) | 5.32(3.76-7.14) | -0.65(-0.91--0.38) |
| Romania | Both | 1023.15(689.32-1296.29) | 2107.19(1366.33-2787.98) | 3.71(2.5-4.7) | 5.64(3.68-7.47) | 1.26(1.04-1.49) |
| Romania | Female | 461.18(302.98-587.21) | 839.95(535.36-1137.14) | 3.03(2-3.86) | 3.85(2.42-5.2) | 0.62(0.46-0.79) |
| Romania | Male | 561.98(381.82-710.99) | 1267.25(832.67-1679.62) | 4.58(3.11-5.78) | 8.06(5.29-10.68) | 1.79(1.52-2.06) |
| Russia | Both | 11289.7(8472.77-13663.51) | 13572.09(9452.9-17522.32) | 6.34(4.76-7.65) | 5.76(4.02-7.42) | -0.87(-1.21--0.53) |
| Russia | Female | 6567.01(4882.3-8032.82) | 7327.16(4965.63-9860.54) | 5.54(4.12-6.77) | 4.8(3.25-6.45) | -1.05(-1.36--0.74) |
| Russia | Male | 4722.69(3545.08-5671.47) | 6244.93(4375.02-8123.13) | 8.38(6.33-10.04) | 7.52(5.29-9.76) | -0.92(-1.29--0.55) |
| Rwanda | Both | 88.42(59.83-115.72) | 165.66(117.36-219.29) | 3.25(2.28-4.22) | 3.07(2.19-3.95) | -0.63(-0.84--0.42) |
| Rwanda | Female | 42.01(24.58-60.89) | 85.31(60.28-112.58) | 2.77(1.64-3.96) | 2.71(1.94-3.52) | -0.33(-0.5--0.16) |
| Rwanda | Male | 46.41(32.9-60.2) | 80.34(53.02-113.04) | 3.88(2.79-4.94) | 3.63(2.44-4.92) | -0.81(-1.05--0.57) |
| Saint Lucia | Both | 3.73(2.81-4.47) | 8.91(6.39-11.29) | 4.53(3.41-5.42) | 4.28(3.07-5.43) | -0.5(-0.8--0.2) |
| Saint Lucia | Female | 1.98(1.42-2.42) | 4.11(2.82-5.27) | 4.18(3.01-5.07) | 3.57(2.45-4.58) | -0.96(-1.3--0.62) |
| Saint Lucia | Male | 1.74(1.32-2.1) | 4.81(3.5-6.13) | 5.06(3.84-6.06) | 5.12(3.74-6.47) | -0.15(-0.42-0.12) |
| Saint Vincent | Both | 3.32(2.65-3.84) | 6.35(4.54-7.88) | 4.78(3.8-5.54) | 4.93(3.54-6.1) | -0.08(-0.34-0.18) |
| Saint Vincent | Female | 1.87(1.48-2.2) | 2.87(2.02-3.61) | 4.71(3.71-5.51) | 4.48(3.14-5.6) | -0.27(-0.6-0.06) |
| Saint Vincent | Male | 1.45(1.15-1.72) | 3.48(2.52-4.36) | 4.85(3.85-5.74) | 5.36(3.94-6.65) | 0.06(-0.16-0.29) |
| Samoa | Both | 3.28(2.51-4.13) | 5.09(3.64-6.6) | 4.02(3.08-5.03) | 3.72(2.67-4.81) | -0.4(-0.46--0.33) |
| Samoa | Female | 1.73(1.27-2.28) | 2.85(1.92-3.88) | 4.03(2.98-5.28) | 3.99(2.68-5.38) | -0.15(-0.22--0.08) |
| Samoa | Male | 1.55(1.17-1.96) | 2.24(1.56-3.1) | 4(3.08-4.97) | 3.44(2.44-4.76) | -0.68(-0.75--0.6) |
| Sao Tome and Principe | Both | 2.33(1.76-2.85) | 5.47(3.73-7.68) | 4.01(3.07-4.87) | 6(4.06-8.41) | 1.45(1.39-1.52) |
| Sao Tome and Principe | Female | 0.7(0.53-0.86) | 1.52(0.98-2.23) | 2.27(1.73-2.79) | 3.12(2.03-4.58) | 1.04(0.96-1.11) |
| Sao Tome and Principe | Male | 1.63(1.22-2.03) | 3.95(2.56-5.79) | 6.25(4.74-7.76) | 9.32(6.03-13.76) | 1.53(1.43-1.64) |
| Saudi Arabia | Both | 117.74(74.07-168.8) | 479.1(301.36-663.63) | 2.21(1.38-3.1) | 2.88(1.83-3.85) | 0.8(0.56-1.03) |
| Saudi Arabia | Female | 52.14(30.04-77.85) | 193.36(119.48-266.62) | 2.29(1.33-3.41) | 2.81(1.75-3.84) | 0.56(0.31-0.81) |
| Saudi Arabia | Male | 65.6(40.03-97.6) | 285.74(178.21-399.69) | 2.14(1.31-3.15) | 2.92(1.87-3.98) | 0.97(0.73-1.22) |
| Senegal | Both | 93.2(70.94-115.52) | 223.59(165.22-289.55) | 3.19(2.45-3.93) | 3.36(2.52-4.29) | 0.26(0.1-0.43) |
| Senegal | Female | 36.62(26.95-46.28) | 102.54(73.77-137.5) | 2.55(1.88-3.22) | 2.97(2.17-3.95) | 0.66(0.5-0.83) |
| Senegal | Male | 56.58(41.69-73.65) | 121.04(85.18-159.95) | 3.81(2.82-4.9) | 3.76(2.72-4.95) | 0.02(-0.14-0.18) |
| Serbia | Both | 693.67(466.41-890.09) | 1213.3(778.48-1637.17) | 6.45(4.34-8.31) | 7.68(4.96-10.33) | 0.71(0.54-0.88) |
| Serbia | Female | 305.14(199.03-405.09) | 477.95(299.98-654.84) | 5.32(3.48-7.14) | 5.52(3.45-7.56) | 0.1(-0.08-0.29) |
| Serbia | Male | 388.53(260.56-511.08) | 735.35(473.35-987.37) | 7.84(5.26-10.2) | 10.39(6.71-13.88) | 1.21(1.04-1.39) |
| Seychelles | Both | 3.25(2.57-3.92) | 8.23(6.09-10.28) | 5.81(4.57-6.99) | 8.14(6.09-10.13) | 0.85(0.63-1.08) |
| Seychelles | Female | 1.66(1.27-2.02) | 3.74(2.66-4.84) | 5.04(3.86-6.13) | 6.74(4.79-8.72) | 0.91(0.74-1.08) |
| Seychelles | Male | 1.59(1.23-1.97) | 4.49(3.29-5.73) | 6.81(5.28-8.42) | 9.67(7.13-12.25) | 0.71(0.4-1.02) |
| Sierra Leone | Both | 49.35(37.14-61.91) | 94.48(67.4-125.73) | 2.75(2.1-3.43) | 2.95(2.15-3.86) | 0.44(0.3-0.59) |
| Sierra Leone | Female | 18.72(13.75-24.56) | 45.31(31.64-61.07) | 2.18(1.61-2.85) | 2.81(1.97-3.79) | 1.16(1.05-1.27) |
| Sierra Leone | Male | 30.62(22.46-39.27) | 49.17(33.73-67.95) | 3.28(2.43-4.16) | 3.08(2.16-4.24) | -0.09(-0.25-0.08) |
| Singapore | Both | 160.46(117.89-198.72) | 332.34(232.52-423.78) | 7.98(5.91-9.86) | 4.45(3.12-5.67) | -2.25(-2.36--2.14) |
| Singapore | Female | 73.65(53.39-92.44) | 155.35(106.71-198.78) | 6.64(4.85-8.34) | 3.84(2.64-4.9) | -2.09(-2.21--1.96) |
| Singapore | Male | 86.81(64.24-106.92) | 176.99(122.45-226.24) | 9.77(7.26-11.96) | 5.15(3.56-6.55) | -2.48(-2.6--2.36) |
| Slovakia | Both | 476.39(347.23-584.46) | 850.95(575.24-1131.31) | 8.03(5.87-9.84) | 9.21(6.21-12.24) | 0.49(0.3-0.68) |
| Slovakia | Female | 198.85(142.16-246.34) | 351.49(232.66-475.92) | 5.74(4.09-7.14) | 6.38(4.23-8.67) | 0.33(0.17-0.49) |
| Slovakia | Male | 277.54(205.12-341.51) | 499.47(336.37-675.91) | 11.26(8.28-13.87) | 13.46(9.09-18.06) | 0.69(0.46-0.93) |
| Slovenia | Both | 166.94(110.71-227.02) | 272.7(184.13-370.82) | 6.89(4.59-9.35) | 5.93(4.01-8.09) | -0.74(-1.04--0.44) |
| Slovenia | Female | 83.74(55.63-113.58) | 112.38(74.37-154.42) | 5.58(3.71-7.57) | 4.02(2.68-5.57) | -1.36(-1.64--1.08) |
| Slovenia | Male | 83.2(54.97-113.75) | 160.31(110.08-217.33) | 9.06(6.04-12.33) | 8.66(5.96-11.72) | -0.37(-0.72--0.02) |
| Solomon Islands | Both | 4.23(2.55-6.24) | 11.2(6.98-15.75) | 3.19(2.12-4.53) | 3.78(2.59-5.11) | 0.48(0.38-0.57) |
| Solomon Islands | Female | 1.75(0.9-3.02) | 4.98(2.82-7.4) | 2.96(1.64-4.99) | 3.52(2.17-5.02) | 0.48(0.35-0.61) |
| Solomon Islands | Male | 2.49(1.53-3.9) | 6.21(3.86-9.5) | 3.38(2.26-5.14) | 4.03(2.78-6) | 0.51(0.44-0.59) |
| Somalia | Both | 54.18(36.64-83.97) | 136.25(82.62-261.22) | 2.3(1.59-3.58) | 2.22(1.35-4.22) | 0.04(-0.03-0.1) |
| Somalia | Female | 24.02(14.72-35.61) | 67.98(38.84-124.95) | 1.9(1.18-2.78) | 1.96(1.13-3.67) | 0.37(0.26-0.47) |
| Somalia | Male | 30.16(19.95-55.97) | 68.27(40.23-140.98) | 2.78(1.85-4.98) | 2.62(1.55-5.26) | -0.13(-0.17--0.09) |
| South Africa | Both | 735.55(559.22-951.82) | 1692.82(1326.52-2069.56) | 3.78(2.85-4.96) | 4.12(3.2-5.02) | 0.3(0.04-0.55) |
| South Africa | Female | 378.49(281.58-501.49) | 814.41(622.09-1020.87) | 3.35(2.48-4.48) | 3.35(2.57-4.19) | 0.01(-0.19-0.22) |
| South Africa | Male | 357.06(271.42-488.53) | 878.4(689.34-1078.33) | 4.33(3.29-6.04) | 5.26(4.17-6.43) | 0.68(0.35-1) |
| South Korea | Both | 1025.06(774.69-1255.15) | 4078.15(3036.39-5052.23) | 3.88(2.97-4.71) | 4.74(3.52-5.88) | 0.35(0.05-0.66) |
| South Korea | Female | 490.34(366.11-608.74) | 1848.23(1351.9-2316.83) | 3.16(2.4-3.87) | 3.62(2.65-4.55) | -0.02(-0.26-0.22) |
| South Korea | Male | 534.73(409.13-657.29) | 2229.92(1649.11-2785.56) | 5.19(3.99-6.39) | 6.27(4.61-7.87) | 0.41(0.03-0.79) |
| South Sudan | Both | 80.65(45.52-135.35) | 121(75.51-187.26) | 3.67(2.12-6.09) | 3.53(2.24-5.4) | -0.12(-0.18--0.05) |
| South Sudan | Female | 32.25(16.57-60.45) | 51.3(29.89-83.53) | 3.21(1.67-5.97) | 3.09(1.89-4.93) | -0.08(-0.17-0.01) |
| South Sudan | Male | 48.4(26-85.06) | 69.7(39.67-112.63) | 4.08(2.21-7.08) | 3.91(2.27-6.3) | -0.14(-0.2--0.07) |
| Spain | Both | 3562.9(2644.22-4334.21) | 6817.32(4968.27-8519.05) | 6.57(4.89-7.98) | 6.43(4.71-8.02) | -0.05(-0.11-0.01) |
| Spain | Female | 1689.2(1236.06-2094.91) | 2886.04(2051.6-3675.61) | 5.31(3.88-6.58) | 4.52(3.21-5.65) | -0.66(-0.74--0.57) |
| Spain | Male | 1873.7(1403.63-2266.4) | 3931.28(2878.18-4911.85) | 8.42(6.31-10.21) | 8.96(6.6-11.17) | 0.32(0.23-0.41) |
| Sri Lanka | Both | 182.55(147.72-216.97) | 528.58(370.06-716.99) | 1.89(1.53-2.23) | 2.25(1.59-3) | 0.99(0.85-1.13) |
| Sri Lanka | Female | 85.48(68.21-103.2) | 279.44(194.91-379.18) | 1.79(1.43-2.16) | 2.11(1.48-2.85) | 0.86(0.73-0.98) |
| Sri Lanka | Male | 97.07(77.58-116.35) | 249.14(169.41-344.8) | 1.98(1.59-2.39) | 2.41(1.66-3.29) | 1.16(0.98-1.34) |
| Sudan | Both | 187.88(133.58-285.7) | 503.08(344.91-766.44) | 2.13(1.55-3.28) | 2.87(1.98-4.41) | 1.11(1.02-1.2) |
| Sudan | Female | 88.43(62.81-116.87) | 215.31(146.47-307.44) | 2.07(1.49-2.77) | 2.6(1.78-3.61) | 0.89(0.82-0.96) |
| Sudan | Male | 99.45(65.37-178.45) | 287.77(185.4-505.94) | 2.17(1.45-3.87) | 3.09(2.02-5.45) | 1.27(1.17-1.37) |
| Suriname | Both | 11.21(8.86-13.39) | 34.74(26.08-44.04) | 4.52(3.57-5.39) | 6.03(4.54-7.64) | 1.05(0.79-1.31) |
| Suriname | Female | 5.37(4.16-6.54) | 15.66(11.58-20.09) | 4.05(3.15-4.93) | 4.95(3.65-6.3) | 0.72(0.51-0.93) |
| Suriname | Male | 5.84(4.56-7.04) | 19.08(14.25-24.63) | 5.07(3.96-6.1) | 7.34(5.5-9.41) | 1.34(1.04-1.64) |
| Sweden | Both | 900.51(630.23-1111.41) | 1127.81(799.86-1400.23) | 5.74(4.06-7.06) | 4.98(3.53-6.19) | -0.61(-0.74--0.47) |
| Sweden | Female | 452.9(314.56-561.38) | 538.82(369.93-686.33) | 4.96(3.5-6.15) | 4.3(2.98-5.46) | -0.56(-0.67--0.45) |
| Sweden | Male | 447.6(319.17-552.77) | 588.99(419.32-733.29) | 6.85(4.88-8.44) | 5.78(4.13-7.17) | -0.76(-0.92--0.6) |
| Switzerland | Both | 495.78(360.01-603.58) | 746.55(534.23-924.01) | 4.62(3.37-5.61) | 3.98(2.88-4.9) | -0.72(-0.91--0.54) |
| Switzerland | Female | 228.11(163.82-281.99) | 330.81(233.77-415.58) | 3.49(2.52-4.29) | 3.07(2.18-3.81) | -0.61(-0.79--0.42) |
| Switzerland | Male | 267.67(195.13-324.25) | 415.74(298.45-514.51) | 6.3(4.59-7.64) | 5.13(3.7-6.34) | -0.97(-1.18--0.75) |
| Syria | Both | 97.06(67.21-127.97) | 240.7(156.27-342.4) | 1.95(1.36-2.59) | 2.24(1.49-3.1) | 0.29(0.06-0.52) |
| Syria | Female | 45.7(30.23-61.77) | 107.63(68.6-156.02) | 1.97(1.29-2.69) | 2.23(1.45-3.13) | 0.25(0.01-0.5) |
| Syria | Male | 51.37(35.59-70.14) | 133.07(85.06-190.47) | 1.94(1.33-2.64) | 2.34(1.51-3.27) | 0.45(0.23-0.68) |
| Taiwan | Both | 757.05(549.33-933.31) | 3484.76(2400.06-4805.02) | 5.07(3.68-6.23) | 8.82(6.08-12.15) | 2.12(1.87-2.37) |
| Taiwan | Female | 290.31(207.69-363.68) | 1361.09(937.87-1885.57) | 4.12(2.94-5.15) | 6.35(4.37-8.81) | 1.56(1.33-1.8) |
| Taiwan | Male | 466.74(341.79-572.2) | 2123.67(1464.44-2934.14) | 6(4.4-7.33) | 11.65(8.04-16.05) | 2.62(2.36-2.89) |
| Tajikistan | Both | 108.71(86.98-128.19) | 181.78(136.7-232.85) | 3.77(3.02-4.43) | 4.41(3.36-5.57) | 1.12(0.72-1.53) |
| Tajikistan | Female | 50.09(39.59-59.44) | 79.5(59.66-103.8) | 3.19(2.52-3.81) | 3.82(2.89-4.93) | 1.18(0.81-1.56) |
| Tajikistan | Male | 58.62(47.01-70.01) | 102.27(76.79-131.97) | 4.46(3.56-5.32) | 5.08(3.83-6.45) | 1.09(0.62-1.56) |
| Tanzania | Both | 290.72(220.55-370.85) | 778.48(560.52-1036.63) | 2.93(2.25-3.67) | 3.43(2.53-4.47) | 0.57(0.51-0.64) |
| Tanzania | Female | 132.48(99.91-167.69) | 373.81(273.67-486.88) | 2.58(1.95-3.27) | 3.1(2.29-3.97) | 0.74(0.6-0.87) |
| Tanzania | Male | 158.24(117.21-215.78) | 404.67(274.94-584.63) | 3.32(2.5-4.44) | 3.82(2.65-5.36) | 0.45(0.4-0.51) |
| Thailand | Both | 1517.11(1229.72-1800.45) | 4069.06(2856.76-5579.96) | 4.6(3.72-5.48) | 4.07(2.87-5.57) | -0.96(-1.21--0.7) |
| Thailand | Female | 685.61(549.67-826.19) | 1815.16(1276.92-2500.23) | 3.9(3.12-4.7) | 3.27(2.28-4.5) | -1.02(-1.25--0.78) |
| Thailand | Male | 831.5(659.77-1003.75) | 2253.91(1562.65-3103.03) | 5.44(4.33-6.61) | 5.03(3.48-6.89) | -0.91(-1.2--0.61) |
| Timor-Leste | Both | 7.93(5.57-10.98) | 36.72(23.34-49.82) | 3.02(2.17-4.1) | 4.9(3.16-6.62) | 2.05(1.8-2.29) |
| Timor-Leste | Female | 4.38(2.93-6.57) | 17.71(11.1-24.19) | 3.35(2.31-4.85) | 4.71(3.02-6.41) | 1.46(1.25-1.67) |
| Timor-Leste | Male | 3.54(2.13-5.32) | 19.01(11.08-26.58) | 2.71(1.65-4) | 5.1(3.08-7.12) | 2.67(2.39-2.95) |
| Tobago | Both | 40.8(29.43-49.21) | 79.31(50.89-108.18) | 5.22(3.79-6.28) | 4.39(2.83-5.98) | -0.98(-1.14--0.81) |
| Tobago | Female | 20.58(14.77-25.12) | 35.23(21.92-48.43) | 4.89(3.53-5.95) | 3.65(2.27-5.05) | -1.45(-1.63--1.27) |
| Tobago | Male | 20.22(14.67-24.24) | 44.07(28.65-60.58) | 5.55(4.05-6.63) | 5.2(3.41-7.14) | -0.54(-0.76--0.32) |
| Togo | Both | 31.85(24.63-40.13) | 97.29(66.62-131.97) | 2.89(2.26-3.59) | 3.14(2.2-4.16) | 0.23(0.19-0.27) |
| Togo | Female | 14.43(10.86-18.31) | 47.77(32.13-66.73) | 2.49(1.87-3.13) | 2.7(1.83-3.73) | 0.31(0.21-0.42) |
| Togo | Male | 17.41(13.04-22.5) | 49.52(33.32-70.79) | 3.33(2.52-4.27) | 3.76(2.56-5.27) | 0.32(0.26-0.39) |
| Tonga | Both | 1.18(0.9-1.46) | 2.04(1.48-2.67) | 2.38(1.83-2.94) | 2.64(1.92-3.45) | 0.28(0.15-0.41) |
| Tonga | Female | 0.69(0.51-0.86) | 1.2(0.83-1.62) | 2.63(1.98-3.28) | 2.75(1.92-3.73) | 0.09(-0.02-0.2) |
| Tonga | Male | 0.5(0.35-0.65) | 0.85(0.6-1.12) | 2.09(1.5-2.72) | 2.49(1.78-3.26) | 0.46(0.2-0.72) |
| Trinidad | Both | 40.8(29.43-49.21) | 79.31(50.89-108.18) | 5.22(3.79-6.28) | 4.39(2.83-5.98) | -0.98(-1.14--0.81) |
| Trinidad | Female | 20.58(14.77-25.12) | 35.23(21.92-48.43) | 4.89(3.53-5.95) | 3.65(2.27-5.05) | -1.45(-1.63--1.27) |
| Trinidad | Male | 20.22(14.67-24.24) | 44.07(28.65-60.58) | 5.55(4.05-6.63) | 5.2(3.41-7.14) | -0.54(-0.76--0.32) |
| Tunisia | Both | 124.69(88.9-158.87) | 352.44(215.48-498.28) | 2.82(1.99-3.57) | 2.98(1.84-4.21) | 0.02(-0.05-0.1) |
| Tunisia | Female | 63.08(42.5-81.75) | 174.45(106.62-249.07) | 2.91(1.95-3.81) | 2.82(1.72-3.99) | -0.24(-0.31--0.16) |
| Tunisia | Male | 61.61(43.13-80.64) | 177.99(107.19-260.43) | 2.73(1.93-3.53) | 3.18(1.96-4.61) | 0.31(0.22-0.39) |
| Turkey | Both | 1369.63(898.03-1843.94) | 3301.82(2138.53-4360.9) | 4(2.63-5.34) | 3.87(2.51-5.1) | 0.2(-0.24-0.64) |
| Turkey | Female | 624.6(400.96-871.22) | 1440.55(922.18-1935.76) | 3.48(2.24-4.81) | 3.15(2.01-4.22) | -0.14(-0.6-0.33) |
| Turkey | Male | 745.03(482.1-1034.34) | 1861.27(1196.83-2472.4) | 4.56(2.96-6.31) | 4.69(3.03-6.21) | 0.5(0.09-0.91) |
| Turkmenistan | Both | 56.08(42.33-66.97) | 96.66(69.1-128.35) | 2.87(2.15-3.43) | 2.68(1.93-3.54) | -0.53(-0.95--0.1) |
| Turkmenistan | Female | 27.48(20.48-33.04) | 47.71(34.47-63.49) | 2.48(1.85-2.99) | 2.37(1.73-3.13) | -0.59(-0.96--0.22) |
| Turkmenistan | Male | 28.59(21.87-34.2) | 48.95(34.58-64.75) | 3.42(2.6-4.07) | 3.13(2.2-4.07) | -0.42(-0.91-0.07) |
| Uganda | Both | 143.86(110-178.29) | 523.23(378.64-683.09) | 2.43(1.88-3) | 4.03(2.96-5.14) | 1.73(1.51-1.95) |
| Uganda | Female | 55.66(40.14-71.46) | 235.24(165.46-309.88) | 1.84(1.35-2.34) | 3.24(2.3-4.23) | 2.03(1.74-2.32) |
| Uganda | Male | 88.2(65.86-111.23) | 287.98(197.67-387.03) | 3.09(2.36-3.86) | 5.14(3.51-6.84) | 1.7(1.5-1.9) |
| UK | Both | 7388(5344.84-9035.26) | 7469.71(5153.33-9213.37) | 8.03(5.82-9.83) | 5.58(3.91-6.87) | -1.47(-1.66--1.27) |
| UK | Female | 3743.62(2689.75-4647.88) | 3443.11(2383.52-4285.16) | 6.68(4.79-8.26) | 4.52(3.1-5.6) | -1.51(-1.74--1.29) |
| UK | Male | 3644.38(2638.17-4470.75) | 4026.6(2817.39-4967.7) | 10.05(7.31-12.26) | 6.86(4.79-8.42) | -1.56(-1.73--1.39) |
| Ukraine | Both | 4983.42(3550.61-6145.11) | 4361.33(2921.43-5696.38) | 6.99(4.97-8.63) | 5.73(3.86-7.48) | -1.64(-2.02--1.25) |
| Ukraine | Female | 2720.08(1892.86-3387.94) | 2010.18(1254.12-2748.1) | 5.9(4.12-7.34) | 4.21(2.6-5.79) | -2.04(-2.37--1.7) |
| Ukraine | Male | 2263.34(1622.38-2778.76) | 2351.15(1587.26-3173.2) | 9.04(6.46-11.08) | 8.31(5.65-11.19) | -1.31(-1.72--0.89) |
| United Arab Emirates | Both | 16.13(9.42-23.67) | 128.14(72.59-188.26) | 5.29(3.06-7.77) | 5.11(2.87-7.71) | -0.14(-0.53-0.25) |
| United Arab Emirates | Female | 6.23(3.48-9.37) | 36.58(19.59-57.83) | 4.96(2.62-7.61) | 4.13(1.94-7.52) | -0.32(-0.98-0.34) |
| United Arab Emirates | Male | 9.9(5.25-15.48) | 91.56(52.69-135.05) | 5.78(3.16-8.73) | 5.51(3.29-7.98) | -0.41(-0.76--0.06) |
| Uruguay | Both | 423.77(322.75-502.54) | 534.68(380.21-659.11) | 10.91(8.31-12.95) | 9.19(6.57-11.28) | -0.7(-0.77--0.63) |
| Uruguay | Female | 220.37(167.41-264.06) | 278.31(198.93-348.36) | 9.72(7.38-11.66) | 7.7(5.44-9.57) | -0.93(-1.01--0.85) |
| Uruguay | Male | 203.4(156.94-240.74) | 256.37(185.22-314.3) | 12.4(9.53-14.62) | 11.25(8.15-13.78) | -0.4(-0.48--0.32) |
| USA | Both | 21198.48(15511.76-26160.97) | 26823.68(19397.05-33359.46) | 6.48(4.75-7.98) | 4.72(3.46-5.84) | -1.26(-1.36--1.15) |
| USA | Female | 10627.63(7720.22-13236.7) | 12601.39(8961.25-15771.31) | 5.39(3.91-6.69) | 3.9(2.79-4.87) | -1.24(-1.32--1.16) |
| USA | Male | 10570.85(7776-12989.73) | 14222.29(10487.67-17523.83) | 8.04(5.92-9.87) | 5.69(4.19-7) | -1.39(-1.53--1.25) |
| Uzbekistan | Both | 297.35(218.66-358.94) | 564.57(398.16-715.26) | 2.59(1.9-3.13) | 3.63(2.6-4.59) | 1.24(1.08-1.39) |
| Uzbekistan | Female | 150.36(110.53-183.51) | 275.4(196.48-354.06) | 2.3(1.7-2.8) | 3.37(2.41-4.3) | 1.5(1.34-1.67) |
| Uzbekistan | Male | 147(109.71-175.68) | 289.18(201.69-372.07) | 2.97(2.21-3.57) | 3.91(2.75-4.91) | 0.88(0.69-1.06) |
| Vanuatu | Both | 1.89(1.33-2.54) | 5.9(4.18-7.92) | 3.19(2.27-4.21) | 3.76(2.7-5.01) | 0.39(0.24-0.54) |
| Vanuatu | Female | 0.77(0.51-1.07) | 2.56(1.73-3.55) | 2.97(2.02-4.07) | 3.48(2.36-4.71) | 0.3(0.11-0.5) |
| Vanuatu | Male | 1.12(0.75-1.53) | 3.34(2.37-4.59) | 3.39(2.29-4.54) | 4.03(2.91-5.44) | 0.48(0.36-0.59) |
| Venezuela | Both | 276.06(214.85-328.75) | 1094.3(751.2-1500.97) | 3.01(2.35-3.59) | 3.88(2.69-5.29) | 0.38(0.18-0.57) |
| Venezuela | Female | 146.39(112.36-177.12) | 532.58(362.39-734.55) | 3.01(2.32-3.66) | 3.5(2.39-4.83) | 0.08(-0.12-0.27) |
| Venezuela | Male | 129.66(101.86-153.61) | 561.72(380.85-779.84) | 2.99(2.35-3.54) | 4.3(2.95-5.91) | 0.66(0.3-1.02) |
| Vietnam | Both | 1541.83(1185.11-1918.19) | 6417.45(4724.04-8208.97) | 3.99(3.07-4.94) | 7.22(5.37-9.13) | 2.33(2.2-2.45) |
| Vietnam | Female | 707.85(536.63-887.61) | 2464.95(1762.63-3177.03) | 3.11(2.36-3.9) | 4.83(3.47-6.18) | 1.77(1.66-1.89) |
| Vietnam | Male | 833.98(628.53-1063.41) | 3952.51(2917.35-5137.13) | 5.33(4.04-6.71) | 10.74(8.07-13.68) | 2.71(2.58-2.84) |
| Virgin Islands | Both | 457.22(328.62-566.11) | 654(464.96-846.49) | 6.53(4.7-8.08) | 4.59(3.26-5.97) | -1.61(-1.77--1.45) |
| Virgin Islands | Female | 229.15(160.58-286.47) | 299.53(207.05-402.59) | 5.45(3.84-6.81) | 3.71(2.56-5.04) | -1.67(-1.81--1.54) |
| Virgin Islands | Male | 228.07(165.85-279.53) | 354.47(241.86-477.54) | 8.13(5.92-9.98) | 5.63(3.85-7.56) | -1.7(-1.9--1.51) |
| Yemen | Both | 108.75(68.6-163.48) | 368.46(263.4-512.41) | 2.41(1.52-3.63) | 2.97(2.12-4.11) | 0.99(0.87-1.11) |
| Yemen | Female | 56.42(35.18-85.89) | 183.1(129.01-253.44) | 2.36(1.45-3.56) | 2.86(2.01-4.03) | 0.92(0.8-1.04) |
| Yemen | Male | 52.33(29.64-86.39) | 185.36(125.28-271.93) | 2.48(1.41-4.09) | 3.07(2.12-4.49) | 1.04(0.92-1.16) |
| Zambia | Both | 107.24(76.6-143.96) | 312.07(221.44-423.33) | 4.1(2.97-5.47) | 5.02(3.57-6.69) | 0.64(0.61-0.67) |
| Zambia | Female | 47.98(29.39-71.22) | 138.38(93.66-197.74) | 3.73(2.33-5.41) | 4.22(2.88-5.97) | 0.44(0.31-0.58) |
| Zambia | Male | 59.26(42.81-78.88) | 173.69(119.88-232.67) | 4.41(3.22-5.84) | 5.95(4.18-7.99) | 0.95(0.84-1.06) |
| Zimbabwe | Both | 142.09(113.79-172.38) | 322.08(231.13-425.44) | 3.95(3.2-4.81) | 5.12(3.72-6.7) | 0.92(0.68-1.17) |
| Zimbabwe | Female | 70.14(55-88.69) | 183.75(125.12-253.62) | 3.74(2.95-4.68) | 5.06(3.45-6.94) | 1.73(1.35-2.11) |
| Zimbabwe | Male | 71.96(55.48-88.37) | 138.33(98.09-182.89) | 4.08(3.15-4.97) | 5.14(3.69-6.59) | 0.22(-0.23-0.67) |

ASDR, age-standardized death rate; EAPC, Estimated annual percentage change; UI, uncertainty interval.
