# Supplementary material for: Dietary Risk-Related Colorectal Cancer Burden: Estimates From 1990 to 2019
Source: Front Nutr. 2021 Aug 24;8:690663. doi: 10.3389/fnut.2021.690663 (PMC8421520; doi:10.3389/fnut.2021.690663)
Supplement: Supplementary file 3 [file Data_Sheet_3.zip › Supplemental tables/Table S22.docx]

**Table S22** DALYs, ASRs and change trends of colorectal cancer attributable to dietary risks between 1990 and 2019 in countries and territories.

| **Location** | **Sex** | **DALYs (No.×1000, 95%UI)** | | **Age-standardized DALY rate (95%UI)** | | **EAPC (95%CI)** |
| --- | --- | --- | --- | --- | --- | --- |
|  |  | **1990** | **2019** | **1990** | **2019** | **1990-2019** |
| Afghanistan | Both | 6268.27(3399.51-9873.03) | 13846.21(8341.65-19937.84) | 84.57(47.27-131.48) | 89.02(56.88-124.11) | 0.29(0.16-0.42) |
| Afghanistan | Female | 3331.95(1564.96-6020.77) | 7949.71(4145.1-12474.43) | 89.74(44.93-156.97) | 96.52(54.41-144.96) | 0.35(0.21-0.49) |
| Afghanistan | Male | 2936.31(1572.61-5630.55) | 5896.5(3752.67-10613.2) | 77.78(42.68-148.17) | 82.2(53.8-144.27) | 0.33(0.2-0.45) |
| Albania | Both | 1507.23(1099.22-1844.24) | 2693.93(1735.35-3841.76) | 68.29(49.55-83.41) | 66.47(42.94-94.63) | 0.24(-0.05-0.53) |
| Albania | Female | 647.86(467.83-793.65) | 1189.87(753.79-1700.92) | 56.93(41.2-69.76) | 55.84(35.02-79.33) | 0.24(0.03-0.46) |
| Albania | Male | 859.37(625.72-1053.73) | 1504.07(967.29-2168.24) | 81.86(59.82-99.99) | 77.85(50.27-111.18) | 0.18(-0.21-0.57) |
| Algeria | Both | 7057.7(4965.28-9127.26) | 17657.83(11291.29-23908.41) | 56.49(39.89-72.26) | 51.22(32.93-69.33) | -0.49(-0.53--0.44) |
| Algeria | Female | 3511.16(2427.44-4648.04) | 8399.56(5310.51-11533.8) | 55.53(39.05-72.47) | 49.4(31.92-67.51) | -0.51(-0.55--0.47) |
| Algeria | Male | 3546.55(2492.51-4671.97) | 9258.27(5869.69-12702.11) | 57.6(40.91-75.05) | 53.11(34.09-72.61) | -0.47(-0.52--0.41) |
| American Samoa | Both | 30.76(21.87-38.73) | 62.43(44.21-80.23) | 124.92(90.02-156.22) | 128.63(92.3-164.33) | 0.15(0.01-0.29) |
| American Samoa | Female | 11.64(8.28-15.04) | 25.22(17.63-33.73) | 99.82(71.1-130.13) | 101.87(71.65-135.42) | 0.09(-0.1-0.28) |
| American Samoa | Male | 19.12(13.44-24.64) | 37.21(26.6-47.94) | 149.01(106.77-190.89) | 157.05(113.95-199.64) | 0.25(0.13-0.36) |
| Andorra | Both | 101.16(65.21-144.83) | 202.49(130.84-278.2) | 181.61(119.7-256.86) | 145.27(93.24-200.23) | -0.85(-0.89--0.8) |
| Andorra | Female | 21.74(13.66-33.3) | 49.37(30.61-69.95) | 81.82(51.74-123.65) | 70.36(43.18-99.85) | -0.55(-0.71--0.39) |
| Andorra | Male | 79.41(50.75-112.64) | 153.12(97.85-207.48) | 279.04(182.2-393.24) | 220.01(141.48-298.05) | -0.86(-0.9--0.82) |
| Angola | Both | 3475.78(2329.3-5249.64) | 10383.47(7189.53-14016.17) | 79.3(54.33-117.17) | 83.34(59.47-109.68) | 0.16(-0.01-0.33) |
| Angola | Female | 1422.56(846.05-2365.97) | 4608.02(3104.77-6458.37) | 64.44(39.22-105.4) | 68.08(47.73-93) | 0.17(0.07-0.28) |
| Angola | Male | 2053.22(1289.07-3163.42) | 5775.45(3814.87-7874.29) | 94.48(59.91-143.24) | 102.28(69.86-136.6) | 0.27(0.05-0.5) |
| Antigua | Both | 50.5(38.01-60.68) | 114.18(79.65-144.28) | 95.98(71.81-115.45) | 113.3(79.35-142.81) | 0.44(0.31-0.57) |
| Antigua | Female | 26.19(19.43-32.15) | 65.98(46-83.58) | 87.49(64.02-108.46) | 123.44(86.29-156.06) | 0.9(0.73-1.07) |
| Antigua | Male | 24.31(18.37-29.43) | 48.2(34.3-61.95) | 106.31(80.47-128.68) | 101.61(72.99-129.98) | -0.09(-0.37-0.19) |
| Argentina | Both | 58903.19(45243.98-69396.48) | 101618.05(75712.09-121487.45) | 182.35(140.04-214.98) | 192.01(142.95-229.46) | 0.19(0.1-0.28) |
| Argentina | Female | 25355.15(19149.93-30297.04) | 43297.46(31436.17-52808.68) | 142.91(107.75-170.79) | 147.4(107.48-179.49) | 0.13(0.05-0.21) |
| Argentina | Male | 33548.03(25980.58-39439.78) | 58320.58(43670.4-70365.65) | 231.08(179.1-271.34) | 246.96(185.05-297.67) | 0.24(0.13-0.35) |
| Armenia | Both | 3781(2717.95-4512.02) | 4187.8(2860.69-5484.33) | 130.65(94.19-156.16) | 101.99(69.75-132.99) | -1.2(-1.33--1.08) |
| Armenia | Female | 1917.81(1348.3-2345.48) | 2173.22(1456.69-2897.21) | 119.62(84.19-145.7) | 93.16(62.63-123.88) | -1.15(-1.27--1.02) |
| Armenia | Male | 1863.19(1358.64-2226.67) | 2014.58(1379.47-2613.25) | 145.79(107.26-172.52) | 113.82(78.34-147.7) | -1.26(-1.45--1.06) |
| Australia | Both | 35178.04(26325.44-42546.94) | 45904.54(33995.92-56433.22) | 182.69(137.03-220.49) | 116.89(87.44-143.58) | -1.9(-2.05--1.75) |
| Australia | Female | 15566.98(11621.88-18992.71) | 19811.06(14387.45-24436.65) | 150.73(112.67-183.12) | 95.36(69.61-116.92) | -1.9(-2.04--1.76) |
| Australia | Male | 19611.05(14796.04-23654.31) | 26093.47(19410.15-31949.32) | 220.56(166.21-266.07) | 140.32(104.88-171.77) | -1.95(-2.12--1.78) |
| Austria | Both | 21135.66(15420.07-26198.16) | 14291.95(10249.34-17864.51) | 187.2(136.74-231.05) | 84.29(60.66-104.75) | -3.21(-3.38--3.04) |
| Austria | Female | 9726.83(7033.35-12169.83) | 5658.35(3941.16-7198.81) | 140.72(101.97-175.54) | 60.25(42.42-76.14) | -3.24(-3.4--3.08) |
| Austria | Male | 11408.83(8366.81-14054.34) | 8633.6(6265.52-10786.21) | 252.84(185.3-311.6) | 112.68(81.77-140.94) | -3.32(-3.51--3.13) |
| Azerbaijan | Both | 6440.74(4923.87-7630.04) | 10493.15(7286.82-13797.04) | 116.3(88.98-137.62) | 101.48(70.7-132.79) | -0.34(-0.71-0.04) |
| Azerbaijan | Female | 2863.78(2178.54-3429.48) | 4558.25(3062.13-6289.2) | 94.24(71.67-112.98) | 83.13(55.91-115.05) | -0.25(-0.57-0.09) |
| Azerbaijan | Male | 3576.96(2759.06-4286.12) | 5934.9(3875.44-8124.81) | 144.8(111-173.27) | 123.33(81.84-167.42) | -0.46(-0.89--0.02) |
| Bahamas | Both | 224.05(161.11-278.21) | 568.99(397.44-765.04) | 135.45(97.61-168.33) | 141.42(99.62-188.91) | 0.35(0.26-0.44) |
| Bahamas | Female | 96.67(68.35-121.27) | 232.72(155.43-317.29) | 106.43(75-133.81) | 107.46(72.52-146.17) | 0.25(0.14-0.35) |
| Bahamas | Male | 127.38(90.86-159.12) | 336.26(235.67-453.18) | 171.77(122.08-214.87) | 181.69(128.48-243.07) | 0.39(0.26-0.52) |
| Bahrain | Both | 173.96(117.24-224.63) | 725.05(441.82-1004.05) | 84.29(56.81-109.42) | 65.86(40.95-89.43) | -1.17(-1.42--0.92) |
| Bahrain | Female | 66.13(43.08-87.16) | 242.1(145.92-340.81) | 71.35(46.49-93.58) | 56.86(34.54-78.5) | -0.9(-1.16--0.64) |
| Bahrain | Male | 107.83(73.46-140.54) | 482.95(295.24-691.69) | 97.86(66.52-127.85) | 73.89(46.06-102.52) | -1.37(-1.67--1.08) |
| Bangladesh | Both | 24237.14(16992.71-32790.37) | 60606.72(40648.36-88482.53) | 48.34(33.62-65.43) | 45.43(30.49-66.06) | -0.27(-0.39--0.16) |
| Bangladesh | Female | 10121.65(6703.03-14051.62) | 28505.7(17766.83-43379.72) | 43.65(28.4-60.89) | 43.67(27.05-66.77) | -0.01(-0.12-0.1) |
| Bangladesh | Male | 14115.49(9444.59-21090.86) | 32101.01(21359.31-49520.34) | 52.1(34.88-77.26) | 46.88(31.54-72.24) | -0.46(-0.61--0.31) |
| Barbados | Both | 341.47(242.08-423.74) | 739.55(492.68-976) | 122.17(86.06-151.99) | 154.25(102.8-203.52) | 0.73(0.56-0.89) |
| Barbados | Female | 173.28(121.44-218.51) | 361.52(233.91-485.48) | 106.82(74.87-134.53) | 136.7(88.86-183.54) | 1.17(0.94-1.39) |
| Barbados | Male | 168.19(120.77-208.07) | 378.03(260.84-496.85) | 141.56(101.37-175.75) | 174.47(120.95-228.91) | 0.26(0.03-0.5) |
| Barbuda | Both | 50.5(38.01-60.68) | 114.18(79.65-144.28) | 95.98(71.81-115.45) | 113.3(79.35-142.81) | 0.44(0.31-0.57) |
| Barbuda | Female | 26.19(19.43-32.15) | 65.98(46-83.58) | 87.49(64.02-108.46) | 123.44(86.29-156.06) | 0.9(0.73-1.07) |
| Barbuda | Male | 24.31(18.37-29.43) | 48.2(34.3-61.95) | 106.31(80.47-128.68) | 101.61(72.99-129.98) | -0.09(-0.37-0.19) |
| Belarus | Both | 20044.7(15044.66-23894.19) | 20218.84(13900.48-27667.14) | 155.44(117.13-185.78) | 129.97(88.92-177.38) | -1.49(-1.81--1.17) |
| Belarus | Female | 10433.52(7690.54-12570.74) | 9357.87(6256.37-12779.99) | 133.71(98.79-160.77) | 99.97(66.93-137.71) | -1.9(-2.24--1.55) |
| Belarus | Male | 9611.18(7242.22-11422.21) | 10860.97(7443.46-14833.92) | 191.43(143.43-228.41) | 179.07(121.72-242.5) | -1.06(-1.37--0.75) |
| Belgium | Both | 22937.2(16000.23-28640.31) | 23869.58(17022.89-29367.78) | 152.7(106.88-190.15) | 109.86(79.21-134.94) | -1.19(-1.25--1.14) |
| Belgium | Female | 11174.8(7759.18-14129.61) | 10210.17(7141.88-12839.88) | 128.28(88.66-161.75) | 84.87(59.95-105.67) | -1.48(-1.56--1.4) |
| Belgium | Male | 11762.4(8326.9-14676.77) | 13659.41(9896.02-16972.86) | 186.37(131.23-232.28) | 138.73(100.58-171.82) | -1.08(-1.15--1.02) |
| Belize | Both | 43.21(29.95-53.47) | 209.48(140.67-267.48) | 45.78(31.83-56.65) | 70.84(47.6-90.62) | 1.3(0.85-1.76) |
| Belize | Female | 21.88(14.9-27.7) | 84.81(55.7-108.69) | 46.77(31.65-59.44) | 58.57(38.72-74.94) | 0.83(0.47-1.19) |
| Belize | Male | 21.32(15.04-26.39) | 124.66(86.55-160.53) | 44.51(31.34-55.11) | 82.89(57.35-105.93) | 1.71(1.15-2.28) |
| Benin | Both | 1169.88(927.48-1437.12) | 3081.01(2219.66-4231.71) | 56.61(44.85-69.85) | 59.88(43.65-80.33) | 0.22(0.18-0.26) |
| Benin | Female | 482.9(377.19-605.08) | 1405.95(999.24-1950.95) | 44.82(35.12-56.18) | 51.38(37.11-70.3) | 0.56(0.51-0.61) |
| Benin | Male | 686.98(525.32-874.95) | 1675.06(1159.83-2336.4) | 69.25(53-88.16) | 69.46(48.91-95.51) | 0(-0.04-0.04) |
| Bermuda | Both | 103.9(71.34-132.07) | 156.98(108.74-202.98) | 165.6(113.88-210.63) | 125.23(86.89-162.34) | -0.92(-0.99--0.84) |
| Bermuda | Female | 47.48(31.95-61.76) | 63.61(41.98-85.89) | 134.61(90.73-174.58) | 89.38(58.72-120.32) | -1.82(-2--1.64) |
| Bermuda | Male | 56.42(39.38-72.65) | 93.37(65.43-121.49) | 203.64(142.68-261.75) | 167.4(118.04-217.86) | -0.23(-0.44--0.02) |
| Bhutan | Both | 117.66(65.48-174) | 304.16(176.24-442.55) | 43.62(24.37-63.28) | 52.95(31.42-76.04) | 0.62(0.59-0.66) |
| Bhutan | Female | 61.72(34.12-101) | 149.2(86.31-229.99) | 45.48(25.28-72.22) | 53.46(31.62-81.42) | 0.38(0.3-0.46) |
| Bhutan | Male | 55.94(27.88-91.74) | 154.97(82.5-238.99) | 41.76(21.54-68.04) | 52.58(28.71-80.55) | 0.89(0.83-0.96) |
| Bolivia | Both | 2767.12(1881.71-3619.78) | 9150.04(6055.74-12800.74) | 82.24(56.2-107.35) | 102.16(67.21-141.97) | 0.65(0.61-0.7) |
| Bolivia | Female | 1570.66(1011.11-2191.43) | 4996.91(3148.74-7130.29) | 88.12(57.48-122.33) | 107.26(68.09-152.83) | 0.56(0.51-0.6) |
| Bolivia | Male | 1196.46(793.93-1627.14) | 4153.13(2730.7-5817.14) | 75.44(49.67-102.23) | 96.55(62.46-135.39) | 0.79(0.7-0.88) |
| Bosnia and Herzegovina | Both | 4364.65(3076.21-5363.22) | 7232.63(4343.07-9970.2) | 102.51(72.88-125.77) | 125.07(75.06-172.41) | 0.85(0.65-1.05) |
| Bosnia and Herzegovina | Female | 2093.29(1431.78-2627.99) | 2841.87(1705.61-3959.61) | 90(62.22-112.34) | 89.71(54.2-125.31) | -0.01(-0.12-0.09) |
| Bosnia and Herzegovina | Male | 2271.36(1641.73-2774.46) | 4390.76(2604.79-6130.33) | 118.47(86.85-145.08) | 168.47(100.03-234.44) | 1.5(1.2-1.8) |
| Botswana | Both | 470.87(320.01-647.12) | 1624.05(1003.96-2387.41) | 79.22(54.64-107.16) | 109.09(69.4-156.26) | 0.75(0.36-1.14) |
| Botswana | Female | 206.76(132.78-295.95) | 704.09(410.47-1094.34) | 63.93(41.74-90.62) | 86.55(52.06-132.24) | 1.16(1-1.32) |
| Botswana | Male | 264.11(177.88-366.52) | 919.96(581.01-1312.67) | 97.76(67.74-132.53) | 138.15(89.69-191.91) | 0.55(-0.05-1.15) |
| Brazil | Both | 73821.58(55853.54-89183.83) | 210037.48(155434.98-258522.12) | 78.13(59.48-94.46) | 87.18(64.61-107.36) | 0.42(0.23-0.61) |
| Brazil | Female | 36817.78(27600.96-44740.95) | 99035.25(72068.99-123242.94) | 74.07(55.47-90.03) | 75.91(55.3-94.46) | 0.13(-0.07-0.32) |
| Brazil | Male | 37003.8(28470.91-44172.86) | 111002.23(82428.75-135871.43) | 82.58(63.7-98.66) | 100.66(74.56-123.14) | 0.73(0.54-0.93) |
| Brunei | Both | 224.51(156.75-293.34) | 623.54(427.84-794.37) | 205.07(142.51-264.92) | 195.18(134.55-247.37) | 0.43(0.1-0.76) |
| Brunei | Female | 90.65(61.68-121.2) | 253.02(172.74-334.03) | 160.73(109.41-213.08) | 151.45(101.68-198.19) | 0.26(0.01-0.51) |
| Brunei | Male | 133.86(93.66-178.84) | 370.52(253.38-490.09) | 266.31(182.93-354.96) | 261.77(183.37-338.31) | 0.7(0.24-1.17) |
| Bulgaria | Both | 19874.86(14095.83-24531.52) | 27080.17(18282.37-36920.07) | 160.6(114.34-198.59) | 202.48(135.92-277.11) | 2.03(1.54-2.52) |
| Bulgaria | Female | 8649.67(6144.83-10766.55) | 10899.3(7321.55-14846.57) | 133.42(95.07-165.85) | 148.09(98.06-202.6) | 1.46(0.99-1.93) |
| Bulgaria | Male | 11225.19(7974.57-13919.24) | 16180.87(10896.34-22101.91) | 192.54(137.21-238.24) | 271.1(183.73-369.81) | 2.51(2.01-3.02) |
| Burkina Faso | Both | 2312.8(1720.77-3057.86) | 5801.71(4261.65-7805.63) | 51.63(38.84-67.67) | 60.06(44.49-80.03) | 0.49(0.3-0.68) |
| Burkina Faso | Female | 1017.89(721.53-1403.82) | 2778.95(1973.78-3730.62) | 42.99(30.45-58.79) | 52.48(37.91-69.73) | 0.7(0.53-0.87) |
| Burkina Faso | Male | 1294.91(964.91-1747.83) | 3022.76(2193.28-4275.19) | 60.98(45.65-82.68) | 69.12(51.23-96.3) | 0.37(0.16-0.59) |
| Burundi | Both | 1780.04(1285.7-2360.98) | 3366.32(2241.67-5015.27) | 70.62(51.65-92.75) | 65.28(43.91-95.77) | -0.49(-0.58--0.39) |
| Burundi | Female | 826.22(516.14-1221.71) | 1431.21(935.53-2032.05) | 59.23(37.56-87.55) | 57.41(37.87-81.02) | -0.34(-0.43--0.25) |
| Burundi | Male | 953.82(708.66-1250.9) | 1935.12(1251.68-3103.11) | 84.53(62.45-110.09) | 72.57(47.78-115.47) | -0.74(-0.85--0.63) |
| Cambodia | Both | 5005.01(3613.05-6768.11) | 17279.91(12690.96-22001.98) | 100.03(73.72-134.57) | 135.93(99.65-171.52) | 1.04(1.01-1.08) |
| Cambodia | Female | 2581.01(1726.8-3552.31) | 8113.75(5815.9-10601.11) | 90(62.56-122.06) | 113.02(81.8-146.88) | 0.7(0.65-0.74) |
| Cambodia | Male | 2424(1705.58-3971.53) | 9166.16(6664.79-11734.49) | 113.35(80.87-184.5) | 167.68(124.43-212.14) | 1.42(1.37-1.46) |
| Cameroon | Both | 3761(2755.08-4816.03) | 10792.06(7319.9-15296.56) | 80.32(59.09-102.25) | 84.48(59.53-117.77) | 0.14(0.09-0.18) |
| Cameroon | Female | 1579.24(1170.44-2070.92) | 4403.47(2935.21-6408.76) | 65.64(48.81-85.17) | 67.19(46.03-95.43) | 0.15(0.11-0.2) |
| Cameroon | Male | 2181.76(1565.53-2866.53) | 6388.59(4144.91-9358.34) | 95.64(68.15-125.56) | 102.7(68-148.36) | 0.13(0.04-0.22) |
| Canada | Both | 45219.52(33083.74-56207.57) | 69364.12(49399.21-87483.69) | 141.36(103.74-175.68) | 106.58(75.81-134.23) | -1.01(-1.09--0.93) |
| Canada | Female | 20361.7(14705.12-25335.38) | 29658.36(20682.83-37987.31) | 116.35(84.25-144.11) | 85.63(60.1-108.79) | -1.09(-1.19--0.99) |
| Canada | Male | 24857.81(18141.01-30598.94) | 39705.76(28448.94-49814.73) | 172.25(125.71-212.32) | 129.93(93.41-163.23) | -1.02(-1.1--0.95) |
| Central African Republic | Both | 932.99(646.68-1255.72) | 1550.6(1021.82-2248.71) | 71.51(51.13-95.16) | 61.47(41.72-88.46) | -0.45(-0.51--0.4) |
| Central African Republic | Female | 384.46(249.35-557.32) | 638.5(416-938.65) | 55.49(37.48-79.7) | 48.16(31.71-69.5) | -0.39(-0.45--0.33) |
| Central African Republic | Male | 548.52(361.72-801.06) | 912.1(571.26-1460.76) | 90.17(62.92-129.47) | 77.53(51.5-122.02) | -0.48(-0.54--0.42) |
| Chad | Both | 1507.1(1114.23-1928.45) | 3986.61(2899.15-5448.34) | 52.07(38.46-66.59) | 67.56(49.52-91.46) | 1.02(0.96-1.09) |
| Chad | Female | 673.13(470.03-906.53) | 1704.94(1210.88-2334.99) | 44.81(31.27-60.66) | 61.48(44.13-83.86) | 1.34(1.24-1.43) |
| Chad | Male | 833.97(577.06-1125.11) | 2281.68(1576.46-3197.56) | 59.6(41.38-80.91) | 72.42(50.51-100.42) | 0.69(0.6-0.78) |
| Chile | Both | 10887.5(8542.32-12759.26) | 28037.7(21768.14-33386.44) | 108(84.54-126.45) | 117.9(91.55-140.38) | 0.7(0.54-0.86) |
| Chile | Female | 5459.22(4241.8-6424.72) | 13508.54(10290.32-16233.12) | 99.83(77.79-117.45) | 103.68(79.21-124.56) | 0.45(0.33-0.57) |
| Chile | Male | 5428.28(4293.47-6381.93) | 14529.16(11216.14-17306.2) | 117.55(93.53-137.75) | 134.44(103.8-159.76) | 0.93(0.72-1.14) |
| China | Both | 834715.34(646748.22-998670.34) | 2234062.97(1609962.28-2831239.7) | 91.54(71.34-109.31) | 111.16(80.19-140.81) | 1.06(0.79-1.33) |
| China | Female | 374688.61(284854.96-467577.45) | 758246.49(528517.34-998854.04) | 81.81(62.29-101.51) | 73.47(51.39-96.82) | -0.24(-0.42--0.07) |
| China | Male | 460026.72(349577.25-572556.04) | 1475816.48(1042791.46-1956517.52) | 103.16(78.74-128.24) | 152.71(108.4-200.81) | 1.92(1.59-2.26) |
| Colombia | Both | 13631.14(10370.01-16159.87) | 36624.1(24081.14-50451.82) | 73.01(55.7-86.64) | 69.65(45.86-95.9) | -0.06(-0.19-0.08) |
| Colombia | Female | 7350.15(5484.46-8776) | 18962.7(12106.98-26546.76) | 77.67(58.1-92.77) | 66.51(42.43-93.1) | -0.43(-0.56--0.29) |
| Colombia | Male | 6280.99(4892.88-7426.27) | 17661.4(11825.87-24648.02) | 67.96(53.12-80.5) | 73.31(49.23-102.16) | 0.36(0.17-0.55) |
| Comoros | Both | 165.12(85.11-235.14) | 401.86(263.17-542.26) | 71.8(39.58-100.92) | 78.87(52.15-105.78) | 0.18(0.03-0.33) |
| Comoros | Female | 79.59(40.94-120.79) | 209.09(136.31-289.72) | 66.34(35.97-99.07) | 76.43(50.33-104.63) | 0.38(0.24-0.52) |
| Comoros | Male | 85.54(43-121.4) | 192.78(119.49-281.02) | 77.66(41.84-109.26) | 82.29(53.1-118.11) | 0.02(-0.17-0.21) |
| Costa Rica | Both | 1258.48(901.34-1549.28) | 5199.45(3303.14-7222.45) | 68.88(49.38-84.78) | 100.58(64.09-139.36) | 1.51(1.35-1.66) |
| Costa Rica | Female | 632.51(446.15-793.97) | 2344.98(1458.78-3223.24) | 67.3(47.33-84.44) | 84.44(52.65-116.23) | 0.98(0.83-1.12) |
| Costa Rica | Male | 625.97(456.96-769.89) | 2854.47(1846.82-3994.83) | 70.53(51.74-86.77) | 119.06(77.18-165.99) | 2.02(1.83-2.22) |
| Croatia | Both | 9064.37(5962.26-11507.77) | 12327.08(7697.72-16972.59) | 142.56(93.77-180.59) | 146.62(90.98-202.66) | 0.21(0.04-0.39) |
| Croatia | Female | 4075.55(2678.32-5194.52) | 4555.7(2806.64-6385.02) | 110.68(72.26-140.47) | 96.23(58.73-136.44) | -0.31(-0.52--0.09) |
| Croatia | Male | 4988.83(3380.46-6252.2) | 7771.38(4828.59-10645.11) | 189.66(128.73-237.2) | 211.97(131.66-291.29) | 0.45(0.25-0.65) |
| Cuba | Both | 11042.3(7852.96-13560.4) | 19352.2(12856.52-26277.43) | 107.11(76.19-131.74) | 103.99(69.2-140.99) | -0.35(-0.54--0.16) |
| Cuba | Female | 5762.75(4034.73-7165.38) | 10084.11(6599.03-13713.25) | 110.4(77.31-137.53) | 102.04(66.58-139.88) | -0.47(-0.61--0.33) |
| Cuba | Male | 5279.56(3819.21-6408.21) | 9268.09(6198.8-12576.98) | 103.74(74.93-125.99) | 105.95(70.81-144.02) | -0.22(-0.49-0.05) |
| Cyprus | Both | 720.28(516.23-912.17) | 1778.35(1249.63-2267.79) | 90.44(65.19-114.19) | 93.46(65.1-118.22) | 0.35(0.12-0.57) |
| Cyprus | Female | 339.09(235.85-443.23) | 741.3(508.27-958.89) | 81.72(57.39-107.36) | 74.06(50.95-95.76) | -0.13(-0.31-0.04) |
| Cyprus | Male | 381.19(276.38-482.62) | 1037.05(706.91-1326.28) | 100.84(73.8-127.37) | 115.03(79.36-146.97) | 0.76(0.46-1.06) |
| Czech Republic | Both | 30174.41(21350.16-38184.66) | 26212.95(17385.01-35417.82) | 222.11(157.31-280.78) | 129.82(85.76-175.84) | -2.02(-2.2--1.84) |
| Czech Republic | Female | 12254.98(8565.67-15672.28) | 9797.02(6412.54-13484.07) | 156.07(109.65-199.04) | 88.3(58.42-121.65) | -2.17(-2.3--2.03) |
| Czech Republic | Male | 17919.43(12849.03-22510.34) | 16415.92(10691.52-22341.03) | 313.66(225.35-394.45) | 180.4(118.29-244.75) | -2.05(-2.26--1.84) |
| Democratic Republic of the Congo | Both | 10000.87(7375.58-13522.06) | 22362.54(14511.22-33539.75) | 59.06(43.71-79.65) | 57.16(37.46-85.57) | -0.25(-0.52-0.02) |
| Democratic Republic of the Congo | Female | 4627.75(3294.33-6216.27) | 10515.47(6670.1-15990.53) | 50.54(36.19-68.18) | 50.26(31.32-78.15) | -0.1(-0.34-0.14) |
| Democratic Republic of the Congo | Male | 5373.12(3812.78-8764.37) | 11847.07(7041.67-22063.44) | 68.63(48.52-113.2) | 66.04(39.62-124.86) | -0.33(-0.61--0.06) |
| Denmark | Both | 12701.03(9263.23-15793.56) | 14951.31(10508.77-18860.18) | 164.15(120.53-203.79) | 135.3(96.23-169.71) | -1.08(-1.3--0.86) |
| Denmark | Female | 6242.32(4498.63-7771.35) | 6650.55(4575.55-8504.46) | 144.65(104.81-179.48) | 113.46(77.94-144.75) | -1.22(-1.42--1.01) |
| Denmark | Male | 6458.71(4777.2-7990.01) | 8300.76(5930.62-10465.91) | 188.8(139.93-233.05) | 159.83(114.71-201.35) | -1.03(-1.27--0.8) |
| Djibouti | Both | 144.37(93.59-206.77) | 672.86(431.87-1002.79) | 88.46(59.7-123.35) | 98.75(66.4-141.18) | 0.32(0.27-0.37) |
| Djibouti | Female | 65.15(42.42-98.33) | 273.1(167.86-409.54) | 81.98(55.03-119.85) | 86.67(57.5-123.97) | 0.15(0.09-0.2) |
| Djibouti | Male | 79.22(46.83-119.27) | 399.76(249.63-604.2) | 95.19(57.53-138.07) | 109.26(70.19-159.44) | 0.4(0.34-0.45) |
| Dominica | Both | 56.06(37.61-71.32) | 81.17(49.92-109.49) | 81.13(54.23-103) | 91.75(56.33-123.56) | 0.57(0.5-0.65) |
| Dominica | Female | 29.14(19.43-37.14) | 36.17(22.7-48.16) | 72.43(48.16-92.63) | 79.76(49.93-106.88) | 0.57(0.48-0.66) |
| Dominica | Male | 26.92(18.47-34.48) | 45(27.74-62.71) | 92.68(62.93-118.68) | 103.27(63.83-142.51) | 0.41(0.31-0.52) |
| Dominican Republic | Both | 2449.17(1913.28-2955.06) | 9173.24(6097.34-12863.68) | 61.88(48.44-74.53) | 95.95(63.99-133.64) | 1.98(1.83-2.14) |
| Dominican Republic | Female | 1210.24(930.65-1494.45) | 3801.09(2518.37-5274.04) | 61.65(47.59-75.73) | 77.88(51.71-107.86) | 1.18(1-1.35) |
| Dominican Republic | Male | 1238.93(957.14-1540.27) | 5372.16(3389.69-7695.84) | 62.06(48.24-76.94) | 114.48(72.09-162.21) | 2.66(2.49-2.83) |
| Ecuador | Both | 3079.92(2363.92-3658.24) | 12986.12(9213.73-17609.19) | 55.24(42.38-65.86) | 84.94(60.42-114.65) | 2(1.69-2.32) |
| Ecuador | Female | 1673.83(1269.32-2017.45) | 6522.21(4581.95-8791.8) | 60.25(45.51-72.29) | 82.35(58.07-110.76) | 1.61(1.29-1.93) |
| Ecuador | Male | 1406.09(1088.88-1674.93) | 6463.91(4534.18-8749.24) | 50.32(39-60) | 87.44(61.14-117.83) | 2.41(2.08-2.74) |
| Egypt | Both | 16122.65(12639.44-19027.87) | 39756.03(24424.58-57613.58) | 48.84(38.04-57.61) | 55.74(34.92-80.13) | 0.42(0.28-0.56) |
| Egypt | Female | 7346.46(5659.43-8793.48) | 15815.27(9685.58-23298.78) | 46.24(35.61-55.15) | 51.27(31.33-75.65) | 0.43(0.31-0.56) |
| Egypt | Male | 8776.19(6831.76-10506.18) | 23940.77(14807.42-35311.56) | 51.2(40.02-60.99) | 60.83(37.67-89.12) | 0.48(0.3-0.65) |
| El Salvador | Both | 1232.28(977.64-1449.54) | 3687.59(2364.43-5133.43) | 39.5(31.26-46.51) | 62.33(39.73-86.7) | 1.36(1.06-1.66) |
| El Salvador | Female | 627.62(496.74-745.24) | 1800.92(1132.89-2528.12) | 38.17(30.23-45.41) | 52.73(33.04-74.26) | 0.99(0.75-1.23) |
| El Salvador | Male | 604.67(476.78-716.45) | 1886.66(1216.78-2672.09) | 40.9(32.16-48.41) | 75(48.2-105.93) | 1.79(1.41-2.17) |
| Equatorial Guinea | Both | 139.18(91.89-199.41) | 553.53(317.84-859.04) | 65.06(43.52-92.06) | 104.83(62.15-159.69) | 2.15(1.99-2.32) |
| Equatorial Guinea | Female | 59.58(34.04-94.61) | 261.62(142.58-444.77) | 50.17(28.84-78.25) | 87.44(50.4-144.05) | 2.52(2.22-2.83) |
| Equatorial Guinea | Male | 79.6(50.77-120.53) | 291.91(151.63-461.14) | 83.96(53.58-127.62) | 129.35(69.93-198.37) | 1.86(1.71-2.01) |
| Eritrea | Both | 774.66(527.26-1077.94) | 2885.44(2029.27-4006.11) | 65.1(45.56-90.44) | 91.79(66.19-123.15) | 0.96(0.7-1.22) |
| Eritrea | Female | 368.03(187.17-611) | 1422.51(956.05-2039.87) | 55.11(28.42-91.36) | 83.39(56.2-119.59) | 1.33(1.1-1.56) |
| Eritrea | Male | 406.63(259.41-570.73) | 1462.93(966.82-2061.51) | 79.44(49.56-108.58) | 103.2(68.99-141.8) | 0.57(0.26-0.88) |
| Estonia | Both | 2826.14(1935.65-3555.01) | 2741.35(1756.71-3758.16) | 138.82(94.99-174.81) | 108.98(69.03-150.5) | -1.03(-1.23--0.83) |
| Estonia | Female | 1478.25(1009.62-1873.79) | 1404.53(879.87-1971.37) | 116.31(79.88-147.31) | 90.35(56.83-127.51) | -1.19(-1.38--1) |
| Estonia | Male | 1347.89(920.7-1688.46) | 1336.81(838.1-1844.52) | 176.73(121.26-221.28) | 139.22(87.58-191.68) | -0.9(-1.16--0.63) |
| Ethiopia | Both | 17780.49(12274.05-27052.66) | 30352.67(21056.02-44240.16) | 77.51(53.55-120.04) | 65.88(45.61-96.68) | -0.75(-1.01--0.48) |
| Ethiopia | Female | 7953.86(4195.6-12058.82) | 13667.87(8928.61-21067.95) | 69.17(38.26-102.88) | 59.25(39.09-91.11) | -0.84(-1.05--0.63) |
| Ethiopia | Male | 9826.63(6250.69-20203.77) | 16684.8(10912-26466.03) | 84.75(53.83-171.99) | 72.18(47.42-115.53) | -0.64(-0.97--0.3) |
| Fiji | Both | 331.61(247.85-428.29) | 716.8(506.99-963.43) | 81.67(60.99-104.29) | 92.9(66.55-123.33) | 0.65(0.41-0.89) |
| Fiji | Female | 147.03(105.73-195.13) | 315.7(218.02-433.83) | 72.79(52.39-94.84) | 79.22(55.52-108.41) | 0.58(0.32-0.84) |
| Fiji | Male | 184.57(135.15-238.05) | 401.11(281.03-538.01) | 91.18(67.31-117.18) | 109.83(79.51-144.55) | 0.79(0.56-1.01) |
| Finland | Both | 6462.3(4606.74-8032.02) | 8316.55(5599.47-10520.12) | 92.62(65.89-114.84) | 73.2(50.16-92.58) | -0.86(-0.92--0.8) |
| Finland | Female | 3260.04(2295.46-4082.65) | 3680.56(2422.7-4731.57) | 77.78(55.21-97.21) | 59.32(40.06-76.06) | -0.99(-1.06--0.93) |
| Finland | Male | 3202.27(2318.8-3954.12) | 4635.99(3224.77-5854.48) | 113.88(82.26-140.52) | 89.51(62.31-112.81) | -0.87(-0.94--0.81) |
| France | Both | 134306.21(100681.39-160376.34) | 148884.42(107494.49-182430.32) | 167.27(125.18-199.53) | 116.56(85.07-142.31) | -1.37(-1.42--1.31) |
| France | Female | 58155.86(43054.64-70759.7) | 63328.52(45392.63-79332.12) | 124.17(92.03-150.39) | 87.65(63.1-108.71) | -1.3(-1.36--1.24) |
| France | Male | 76150.34(57025.69-90716.23) | 85555.9(62761.94-104955.03) | 224.62(168.06-267.9) | 151.2(111.62-185.19) | -1.5(-1.56--1.44) |
| Gabon | Both | 797.31(490.81-1225.07) | 1398.39(909.11-1882.42) | 137.05(85.41-210.11) | 125.22(83.3-165.22) | -0.45(-0.52--0.38) |
| Gabon | Female | 329(189.43-547.51) | 520.98(339.03-741.4) | 104.85(61.49-172.71) | 89.04(60.35-124.29) | -0.72(-0.88--0.57) |
| Gabon | Male | 468.31(277.8-763.67) | 877.41(534.99-1201.19) | 176.28(105.65-285.74) | 166.46(105.14-224.93) | -0.34(-0.44--0.24) |
| Gambia | Both | 165.03(119.46-222.49) | 558.06(369.27-773.51) | 43.79(32.18-57.84) | 55.45(37.28-77.05) | 0.59(0.4-0.78) |
| Gambia | Female | 70.82(49.83-94.85) | 262.19(165.42-380.72) | 40.51(29.02-53.38) | 51.02(32.47-74.29) | 0.58(0.38-0.78) |
| Gambia | Male | 94.21(66.72-130.18) | 295.87(189.22-430.35) | 46.31(33.57-62.89) | 59.93(38.34-86.06) | 0.65(0.47-0.84) |
| Germany | Both | 200358.26(148083.26-249153.02) | 201932.5(145845.05-251958.06) | 162.16(119.87-201.78) | 112.87(81.35-140.5) | -1.64(-2.09--1.18) |
| Germany | Female | 101929.65(74032.74-127404.47) | 85291.59(59630.41-108059.08) | 135.97(98.84-169.08) | 85.35(59.93-107.76) | -2.05(-2.5--1.6) |
| Germany | Male | 98428.62(74013.67-121558) | 116640.91(86112.97-144807.18) | 201.02(150.49-247.62) | 143.92(106.15-178.71) | -1.51(-1.96--1.06) |
| Ghana | Both | 3847.59(2913.78-4892.67) | 11869.56(8206.05-15890.77) | 56.73(43.06-71.66) | 68.32(48.03-90.2) | 0.64(0.61-0.68) |
| Ghana | Female | 1867.17(1374.43-2419.21) | 5020.61(3526.33-6657.69) | 53.27(39.96-68.05) | 52.95(37.85-69.42) | -0.24(-0.34--0.15) |
| Ghana | Male | 1980.42(1431.12-2564.84) | 6848.95(4513.94-9600.49) | 60.37(44.23-77.33) | 87.34(57.99-121.05) | 1.47(1.39-1.56) |
| Greece | Both | 14228.31(10327.33-17265.59) | 22839.2(16434.42-27770.05) | 95.07(69.35-115.28) | 103.04(75.78-124.73) | -0.05(-0.19-0.08) |
| Greece | Female | 7023.47(5012.35-8583.62) | 9853.68(6871.06-12139.69) | 87.22(62.62-106.18) | 81.26(57.63-99.86) | -0.65(-0.8--0.49) |
| Greece | Male | 7204.84(5264.63-8794.99) | 12985.52(9607.91-15814.57) | 104.34(76.86-126.99) | 128.24(94.43-156.4) | 0.44(0.31-0.57) |
| Greenland | Both | 85.42(61.41-109.03) | 151.55(102.77-199.09) | 222.77(160.9-282.65) | 213.43(145.89-281.33) | -0.51(-0.8--0.23) |
| Greenland | Female | 40.63(28.12-52.83) | 63.51(42.78-85.68) | 221.94(154.21-287.52) | 194.42(130.93-262.19) | -1.17(-1.61--0.73) |
| Greenland | Male | 44.79(31.17-58.2) | 88.04(58.57-116.42) | 225.94(160.88-286.68) | 229.56(155.52-301.95) | 0.06(-0.11-0.24) |
| Grenada | Both | 76.93(55.51-92.81) | 148.44(102.67-184.71) | 110.67(79.79-133.64) | 132.69(91.92-164.79) | 0.74(0.61-0.87) |
| Grenada | Female | 36.99(26.24-45.57) | 63.97(41.99-80.68) | 92.63(64.43-114.43) | 110.5(72.79-138.93) | 0.76(0.44-1.09) |
| Grenada | Male | 39.94(29.2-49.04) | 84.47(58.92-104.55) | 133.82(98.09-164.11) | 155.63(108.69-191.52) | 0.64(0.34-0.94) |
| Grenadines | Both | 72.87(57.48-85.28) | 141.25(100.18-177.15) | 101.36(80.46-118.76) | 105.14(74.55-131.82) | -0.17(-0.42-0.07) |
| Grenadines | Female | 38.45(29.99-45.43) | 60.09(41.86-75.72) | 97.62(76.06-115.46) | 91.97(64.15-115.78) | -0.48(-0.77--0.19) |
| Grenadines | Male | 34.42(27.18-40.91) | 81.16(58.22-102.46) | 105.39(83.28-124.61) | 117.37(84.4-147.22) | 0.04(-0.19-0.27) |
| Guam | Both | 93.13(65.73-117.78) | 197.24(132.38-258.85) | 114.78(80.33-146.42) | 104.39(69.85-136.09) | -0.31(-0.67-0.06) |
| Guam | Female | 27.64(19.27-36.13) | 61.07(39.71-81.79) | 73.53(50.57-97.8) | 63.73(41.16-85.51) | -0.62(-0.91--0.33) |
| Guam | Male | 65.49(45.69-84.03) | 136.17(92.1-181.74) | 155.13(107.02-200.11) | 145.95(99.47-193.48) | -0.09(-0.53-0.36) |
| Guatemala | Both | 1626.54(1280.91-1948.25) | 8231.83(5886.54-10821.25) | 41.55(33.06-49.44) | 69.69(50-91.41) | 1.78(1.47-2.08) |
| Guatemala | Female | 833.79(638.82-1024.38) | 3993.96(2826.53-5236.45) | 43.15(33.27-52.54) | 63.18(44.74-82.33) | 1.24(1.01-1.48) |
| Guatemala | Male | 792.74(607.33-972.6) | 4237.87(3043.73-5625.86) | 40.21(31.19-49.52) | 77.57(55.66-102.52) | 2.33(1.95-2.71) |
| Guinea | Both | 1709.52(1331.06-2120.46) | 3558.93(2497.6-4810.79) | 50.41(39.65-62.46) | 61.75(43.6-83.02) | 0.8(0.77-0.83) |
| Guinea | Female | 802.6(609.73-1030.45) | 1478.85(975.79-2061.58) | 47.03(35.59-60.23) | 50.81(34.01-70.29) | 0.27(0.22-0.31) |
| Guinea | Male | 906.92(682.79-1145.48) | 2080.08(1417-2945.56) | 53.73(40.43-67.42) | 72.23(49.47-101.71) | 1.17(1.07-1.26) |
| Guinea-Bissau | Both | 388.97(259.03-524.06) | 679.99(478.31-910.06) | 88.64(59.91-117.92) | 84.6(59.95-111.59) | -0.14(-0.18--0.1) |
| Guinea-Bissau | Female | 144.02(93.82-204.74) | 308.22(214.49-430.54) | 61.98(41.09-86.87) | 70.05(49.84-96) | 0.5(0.45-0.54) |
| Guinea-Bissau | Male | 244.95(151.39-339.95) | 371.78(250.88-528.56) | 116.62(73.45-161.8) | 101.68(68.82-142.89) | -0.51(-0.56--0.47) |
| Guyana | Both | 448.94(355.46-543.06) | 691.26(462.44-931.28) | 110.49(86.94-133.56) | 105.07(71.02-140.66) | -0.2(-0.35--0.05) |
| Guyana | Female | 199.25(151.06-248.66) | 302.71(194.81-415.54) | 96.63(73.19-119.31) | 88.52(57.79-120.46) | -0.32(-0.46--0.18) |
| Guyana | Male | 249.69(192.14-307.99) | 388.55(256.51-526.76) | 124.92(96.54-154.16) | 123.18(82.9-167.03) | -0.09(-0.26-0.08) |
| Haiti | Both | 3376.6(2203.73-4393.86) | 7239.14(4834.89-10345.79) | 96.31(63.49-124.43) | 95.91(63.66-134.51) | 0.15(0.06-0.24) |
| Haiti | Female | 1830.22(1114.14-2567.01) | 4035.94(2567.46-6141.48) | 100.35(61.86-138.66) | 100.45(63.75-150.48) | 0.15(0.06-0.23) |
| Haiti | Male | 1546.38(1021.7-2175.86) | 3203.2(2078.9-4629.03) | 91.83(62.08-128.58) | 90.81(58.91-131.35) | 0.15(-0.02-0.32) |
| Honduras | Both | 901.41(675.72-1127.63) | 3273.84(2082.32-4874.57) | 39.68(29.77-49.32) | 52.29(32.95-78.15) | 1.02(0.9-1.13) |
| Honduras | Female | 492.79(372.43-626.73) | 1800.72(1106.5-2831.29) | 42.54(31.99-53.82) | 54.18(34.28-84.34) | 0.82(0.67-0.97) |
| Honduras | Male | 408.62(282.34-545.1) | 1473.12(814.08-2314.81) | 36.67(24.87-48.84) | 50.19(27.01-79.14) | 1.27(1.08-1.46) |
| Hungary | Both | 29989.03(21112.23-37470.13) | 37744.3(25314.12-50041.08) | 207.69(146.46-258.67) | 206.9(140.01-275.98) | -0.19(-0.41-0.04) |
| Hungary | Female | 13416.29(9255.86-16883.59) | 15054.62(10051.55-19977.74) | 160.29(110.28-201.08) | 141.78(94.48-189.91) | -0.64(-0.87--0.41) |
| Hungary | Male | 16572.73(11801.65-20621.17) | 22689.68(15316.98-30276.03) | 273.9(194.04-340.31) | 295.39(200.02-394.51) | 0.12(-0.12-0.37) |
| Iceland | Both | 307.92(230.3-370.33) | 424.87(300.83-533.48) | 110.16(82.41-132.54) | 79.41(56.3-99.53) | -1.33(-1.47--1.18) |
| Iceland | Female | 146.66(108.19-180.53) | 152.77(106.79-194.96) | 98.72(72.8-121.86) | 54.32(38.28-69) | -2.44(-2.68--2.2) |
| Iceland | Male | 161.26(119.78-196.05) | 272.09(193.33-343.13) | 123.97(92.29-150.59) | 105.99(75.37-133.45) | -0.6(-0.73--0.47) |
| India | Both | 206161.1(159883.77-251945.74) | 600576.44(437433.89-761657.48) | 42.72(33.48-52.09) | 51.65(37.79-65.43) | 0.55(0.43-0.67) |
| India | Female | 100460.39(73642.22-130720.57) | 304767.62(210332.55-402900.82) | 42.55(31.22-55.39) | 51.27(35.55-67.56) | 0.49(0.31-0.66) |
| India | Male | 105700.71(79490.61-134563.4) | 295808.82(210258.95-386837.8) | 43.01(32.77-54.81) | 52.21(37.38-67.68) | 0.62(0.52-0.71) |
| Indonesia | Both | 96831.14(69567.04-123199.56) | 306136.74(200990.04-413620.21) | 87.77(63.78-111.1) | 133.34(87.75-180.89) | 1.43(1.29-1.57) |
| Indonesia | Female | 51056.95(33038-69520.03) | 136302.64(81681.06-202330.88) | 88.95(58.94-119.31) | 115.48(69.65-169.86) | 0.82(0.65-0.99) |
| Indonesia | Male | 45774.19(34035.23-57706.1) | 169834.1(111785.04-235464.2) | 86.68(64.68-109.58) | 152.8(98.88-210.55) | 2.01(1.9-2.13) |
| Iran | Both | 18544.65(14213.31-23269.79) | 58828.05(44254.91-69661.17) | 65.67(50.26-82.56) | 76.29(57.5-90.45) | 0.74(0.54-0.94) |
| Iran | Female | 8061.58(5912.91-10666.49) | 25212.21(18692.2-29864.65) | 59.6(43.32-78.61) | 65.64(48.69-78.1) | 0.55(0.39-0.71) |
| Iran | Male | 10483.07(7672.64-13452.62) | 33615.85(25464.77-39754.25) | 71.18(52.63-91.36) | 87.09(66.27-102.71) | 0.94(0.71-1.18) |
| Iraq | Both | 5510.13(3773.25-7794.43) | 19770.68(14096.64-26708.03) | 66.08(45.51-93.12) | 76.73(55.72-100.59) | 0.62(0.49-0.75) |
| Iraq | Female | 2593.89(1696.41-3855.18) | 8949.89(6209.46-12210.43) | 60.83(39.9-90.01) | 67.36(47.83-89.39) | 0.53(0.38-0.68) |
| Iraq | Male | 2916.25(1798.36-4298.28) | 10820.78(7607.84-14490.45) | 71.94(44.46-105.09) | 86.89(62.14-113.62) | 0.69(0.56-0.81) |
| Ireland | Both | 6592.27(4763.23-8220.12) | 8122.02(5863.56-10281.14) | 165.81(120.21-206.27) | 111.03(80.15-140.39) | -1.46(-1.52--1.41) |
| Ireland | Female | 2793.86(1995.12-3509.63) | 3202.13(2236.1-4132.95) | 130.94(94.35-164.28) | 83.61(58.85-107.37) | -1.59(-1.67--1.52) |
| Ireland | Male | 3798.42(2747.82-4753.13) | 4919.9(3566.2-6266.96) | 207.93(150.17-259.71) | 141.29(102.64-179.56) | -1.44(-1.51--1.37) |
| Israel | Both | 6225.16(4184.64-7736.28) | 10664.22(7017.41-13352.5) | 130.03(87.07-161.7) | 94.76(62.32-118.76) | -1.81(-2.09--1.52) |
| Israel | Female | 3017.31(2005.03-3762.12) | 4807.76(3096.48-6051.95) | 117.96(78.22-147.13) | 78.84(50.63-99.18) | -2.12(-2.41--1.82) |
| Israel | Male | 3207.85(2151.64-4006.32) | 5856.46(3933.97-7328.8) | 145.03(97.13-180.01) | 113.32(76.38-141.69) | -1.57(-1.86--1.27) |
| Italy | Both | 131345.65(98526.3-156537.49) | 156140.22(115435.59-188593.15) | 152.58(114.91-181.91) | 118.38(87.81-142.76) | -1.07(-1.18--0.96) |
| Italy | Female | 60577.66(44977.5-72773.02) | 66304.08(47762.69-81037.92) | 125.49(93.49-150.35) | 90.87(65.79-110.38) | -1.32(-1.41--1.23) |
| Italy | Male | 70767.99(53311.61-84093.67) | 89836.15(66619.36-108213.85) | 188.47(142.08-223.89) | 151.07(111.83-181.45) | -0.94(-1.08--0.8) |
| Ivory Coast | Both | 3544.8(2540.74-4820.84) | 8971.82(6345.67-11952.17) | 79.99(57.84-107.5) | 78.24(56.52-102.66) | -0.38(-0.52--0.24) |
| Ivory Coast | Female | 1154.99(825.48-1570.52) | 3432.49(2353.66-4593.03) | 56.76(41.72-76.35) | 63.1(45.17-83.37) | 0.42(0.34-0.5) |
| Ivory Coast | Male | 2389.81(1673.2-3326.73) | 5539.33(3856.87-7483.13) | 99.9(70.99-138.51) | 91.9(64.36-121.82) | -0.76(-0.97--0.55) |
| Jamaica | Both | 1257.91(882.69-1542.66) | 3433.64(2274.42-4605.55) | 71.18(50.02-87.16) | 115.56(76.48-154.93) | 1.88(1.54-2.23) |
| Jamaica | Female | 626.11(433.97-775.47) | 1507.33(962.03-2053.33) | 66.28(45.78-81.96) | 97.57(61.91-132.97) | 1.35(1.07-1.64) |
| Jamaica | Male | 631.8(454.12-766.9) | 1926.32(1283.78-2606.93) | 76.65(55.15-93.04) | 134.2(90-181.76) | 2.29(1.82-2.77) |
| Japan | Both | 222950.68(162245.43-275497.9) | 348966.36(254890.04-426399.62) | 131.97(96.2-162.77) | 114.75(84.64-139.54) | -0.5(-0.56--0.43) |
| Japan | Female | 95081.97(68660.99-118412.8) | 146501.68(102629.12-183083.9) | 102.44(74-127.51) | 85.52(63.6-104.93) | -0.62(-0.68--0.57) |
| Japan | Male | 127868.72(93626.21-157027.17) | 202464.69(147620.71-246230.79) | 169.17(123.69-207.36) | 147.17(108.12-178.29) | -0.51(-0.59--0.43) |
| Jordan | Both | 1699.79(1273.06-2193) | 7374.82(5201.87-9472.78) | 113.07(83.46-145.11) | 103.39(73.29-132.68) | -0.33(-0.4--0.26) |
| Jordan | Female | 877.19(629.51-1175.22) | 3102.25(2126.25-4225.48) | 116.42(82.96-156.02) | 91.88(64.11-122.94) | -0.94(-1.08--0.8) |
| Jordan | Male | 822.6(588.77-1106.6) | 4272.57(2964.94-5795.43) | 110.76(79.45-146.95) | 114.02(79.31-153.15) | 0.18(0.03-0.33) |
| Kazakhstan | Both | 18917.79(14263.29-22725.62) | 18809.41(13468.11-23733.27) | 141.56(106.63-170.19) | 105.03(75.01-132.6) | -0.94(-1.06--0.82) |
| Kazakhstan | Female | 9528.09(7149.41-11516.79) | 9236.22(6612.05-11709.94) | 121.08(90.41-146.28) | 88.93(64.03-113.06) | -1.1(-1.22--0.98) |
| Kazakhstan | Male | 9389.7(7072.13-11314.74) | 9573.19(6817.03-12160.64) | 175.75(131.26-212.48) | 130.82(93.62-165.53) | -0.82(-0.96--0.68) |
| Kenya | Both | 3362.5(2156.47-4498.52) | 13216.17(8628.91-18289.01) | 37.97(24.31-51) | 54.46(36.28-74.35) | 1.52(1.34-1.69) |
| Kenya | Female | 1851.22(1100.87-2714.11) | 6694.39(4073.46-10326.17) | 40.54(24.3-59.36) | 52.53(32.19-79.97) | 1.19(1.01-1.36) |
| Kenya | Male | 1511.28(889.38-2168.56) | 6521.77(4271.9-8945.51) | 35.31(20.61-50.69) | 56.76(37.9-76.43) | 1.87(1.61-2.14) |
| Kiribati | Both | 49.44(36.9-62.4) | 82.98(57.64-113.84) | 116.5(87.75-146.68) | 106.46(74.55-144.41) | -0.52(-0.59--0.45) |
| Kiribati | Female | 17.15(12.84-22.32) | 29.84(20.18-41.29) | 77.76(57.2-100.8) | 73.46(50.85-100.69) | -0.41(-0.49--0.33) |
| Kiribati | Male | 32.29(23.77-41.78) | 53.15(36.44-73.86) | 162.15(119.79-208.51) | 148.09(103.48-203.89) | -0.5(-0.57--0.43) |
| Kuwait | Both | 341.79(232.73-440.96) | 1570.26(1015.72-2111.93) | 46.7(31.47-60.3) | 55.65(35.72-73.83) | 1.32(0.88-1.77) |
| Kuwait | Female | 150.23(95.95-201.54) | 511.03(318.04-724.3) | 54.63(34.89-73.1) | 41.21(25.4-57.53) | -0.11(-0.7-0.49) |
| Kuwait | Male | 191.56(127.35-243.82) | 1059.23(661.94-1479.04) | 41.82(28.09-53.53) | 65.41(41.01-90.57) | 2.14(1.72-2.56) |
| Kyrgyzstan | Both | 3349.06(2462.37-4114.85) | 2862.48(1988.93-3629.6) | 105.84(77.65-129.89) | 59.37(41.37-74.68) | -2.34(-2.58--2.1) |
| Kyrgyzstan | Female | 1690.63(1222.28-2109.68) | 1389.91(962.49-1787.66) | 93.55(67.52-116.62) | 52.46(35.92-66.99) | -2.22(-2.43--2.01) |
| Kyrgyzstan | Male | 1658.43(1222.2-2036.65) | 1472.57(1034.74-1880.85) | 122.98(89.9-151.68) | 67.52(47.34-85.04) | -2.53(-2.83--2.23) |
| Laos | Both | 2611.19(1668.12-3656.61) | 6107.54(4148.77-8366.23) | 113.65(73.91-157.84) | 126.25(87.56-171.49) | 0.17(0.09-0.24) |
| Laos | Female | 1292.96(768.11-1973.67) | 2757.44(1777.91-4046.23) | 107.23(65.18-163.3) | 111.2(73.07-160.89) | -0.12(-0.21--0.02) |
| Laos | Male | 1318.23(841.9-2080.83) | 3350.1(2193.7-4645.75) | 120.89(78.16-190.98) | 142.3(94.58-195.58) | 0.41(0.36-0.47) |
| Latvia | Both | 5145.8(3682.53-6353.59) | 4343.4(2861.27-5771.45) | 145.48(104.15-179.22) | 115.47(75.53-153.89) | -1.01(-1.29--0.72) |
| Latvia | Female | 2754.96(1931.81-3438.82) | 2227.02(1459.42-3138.25) | 125.76(88.44-157.59) | 95.37(61.09-136.54) | -1.17(-1.42--0.93) |
| Latvia | Male | 2390.84(1708.33-2943.41) | 2116.38(1337.25-2870.08) | 180.58(129.83-222.78) | 149.34(94.89-201.63) | -0.83(-1.18--0.48) |
| Lebanon | Both | 2099.71(1373.22-2863.23) | 6016.21(3811.34-8159.58) | 91.41(60.27-123.39) | 114.73(72.65-155.79) | 1.35(1.13-1.56) |
| Lebanon | Female | 1066.55(685.68-1499.52) | 2933.34(1823.11-4226.91) | 90.46(58.35-126.63) | 102.34(63.49-146.87) | 0.73(0.61-0.86) |
| Lebanon | Male | 1033.15(656.34-1439.52) | 3082.88(1935.8-4330.12) | 92.56(59.5-128.12) | 129.84(81.43-182.56) | 1.98(1.64-2.32) |
| Lesotho | Both | 547.46(392.93-803.2) | 1354.47(904.65-1913.9) | 54.81(39.51-80.7) | 100.79(68.38-139.75) | 2.6(2.37-2.83) |
| Lesotho | Female | 240.89(162.21-364.81) | 597.22(356.74-876.96) | 43.34(29.35-65.41) | 79.12(48.32-116.15) | 2.91(2.57-3.25) |
| Lesotho | Male | 306.57(215.19-458.8) | 757.24(509.51-1097.76) | 68.95(49.22-104.18) | 129.67(89.88-186) | 2.44(2.2-2.68) |
| Liberia | Both | 691.68(528.01-882.44) | 1319.61(844.9-1943.99) | 60.87(46.66-77.33) | 59.77(39.09-87.36) | 0.33(0-0.67) |
| Liberia | Female | 274.46(208.61-353.58) | 606.49(374.61-911.6) | 52.1(39.64-66.41) | 56.13(34.94-82.75) | 0.7(0.39-1) |
| Liberia | Male | 417.22(305.88-551.2) | 713.12(416.63-1151.7) | 68.39(50.39-90.15) | 63.26(38.14-101.9) | 0.1(-0.26-0.46) |
| Libya | Both | 1924.36(1218.7-2867.6) | 5757.4(3678-8096.23) | 95.01(60.1-140.94) | 100.48(63.44-140.23) | 0.24(0.12-0.35) |
| Libya | Female | 978.97(579.58-1517.02) | 2961.53(1843.74-4387.11) | 103.95(61.94-161.38) | 102.91(63.52-150.65) | 0.07(-0.13-0.28) |
| Libya | Male | 945.39(523.8-1457.86) | 2795.86(1704.06-4138.61) | 88.53(48.67-136.46) | 98.36(60.12-143.84) | 0.34(0.22-0.47) |
| Lithuania | Both | 6277.48(4519.01-7658.28) | 6190.01(3994.53-8234.53) | 140.62(101.11-171.87) | 115.16(74.54-154.89) | -0.73(-0.97--0.5) |
| Lithuania | Female | 3172.49(2258.76-3921.9) | 2944.24(1904.26-3992.02) | 119.43(85.13-147.63) | 90.29(57.6-124.08) | -1.11(-1.31--0.9) |
| Lithuania | Male | 3104.99(2274.28-3809.03) | 3245.76(2116.82-4330.49) | 174.77(128.11-214.09) | 156.55(101.82-207.98) | -0.35(-0.63--0.06) |
| Luxembourg | Both | 962.81(696.32-1179.99) | 984.11(702.51-1261.14) | 179.59(130.82-220.43) | 100.34(71.7-128.61) | -2.18(-2.39--1.97) |
| Luxembourg | Female | 452.12(323.63-563.79) | 418.56(290.76-537.57) | 145.67(105.19-181.08) | 79.14(54.88-102.24) | -2.24(-2.41--2.06) |
| Luxembourg | Male | 510.7(372.96-631.6) | 565.56(407.71-727.93) | 227.04(166.5-280.32) | 124.13(89.65-159.48) | -2.27(-2.52--2.01) |
| Macedonia | Both | 2268.67(1604.44-2788.81) | 4766.82(3008.82-6563.83) | 117.31(82.4-144.21) | 148.09(93.98-203.99) | 0.77(0.51-1.03) |
| Macedonia | Female | 978.7(670.12-1236.24) | 1900.61(1185.53-2622.88) | 97.73(66.43-123.36) | 114.66(71.71-158.69) | 0.59(0.4-0.79) |
| Macedonia | Male | 1289.97(922.18-1596.31) | 2866.21(1784.36-3948.78) | 138.66(99.4-170.24) | 184.61(115.84-253.21) | 0.9(0.56-1.23) |
| Madagascar | Both | 3438.99(2550.1-4416.7) | 8119.76(5565.92-11526.19) | 60.92(45.69-77.19) | 64.09(44.1-90.02) | 0.1(0.03-0.17) |
| Madagascar | Female | 1652.08(1120.16-2284.13) | 4117.39(2663.59-6039.88) | 57.09(39.83-77.43) | 61.85(40.92-90.16) | 0.15(0.08-0.23) |
| Madagascar | Male | 1786.9(1317.01-2325.72) | 4002.37(2534.54-5976.99) | 64.41(47.27-84.31) | 66.66(42.99-98.13) | 0.09(0.01-0.16) |
| Malawi | Both | 1713.97(1317.48-2132.7) | 4019.36(2789.64-5374.42) | 41.5(32.09-51.18) | 51.53(36.6-66.96) | 0.81(0.64-0.99) |
| Malawi | Female | 812.74(614.75-1050.27) | 1746.8(1179.53-2392.47) | 37.23(28.26-47.65) | 41.55(28.57-56.24) | 0.49(0.41-0.58) |
| Malawi | Male | 901.23(676.36-1157.68) | 2272.55(1498.75-3147.11) | 46.37(34.99-58.75) | 63.62(42.93-85.37) | 1.13(0.86-1.4) |
| Malaysia | Both | 14122.98(11372.62-16968.61) | 43433.89(31587.32-57082.7) | 145.87(116.96-174.38) | 160.97(117.58-210.77) | -0.11(-0.32-0.09) |
| Malaysia | Female | 6880.1(5426.09-8396.36) | 18337.5(13238.43-24454.84) | 138.85(108.03-170.98) | 138.35(100.51-183.16) | -0.38(-0.57--0.2) |
| Malaysia | Male | 7242.88(5776.34-8794.84) | 25096.39(18131.08-33040.65) | 153.75(121.75-187.5) | 183.46(132.78-240.8) | 0.08(-0.15-0.32) |
| Maldives | Both | 80.17(51.94-107.08) | 192.68(135.25-243.45) | 83.19(56.71-109.23) | 59.33(41.22-75.16) | -1.75(-1.99--1.5) |
| Maldives | Female | 39.14(21.66-59.05) | 80.58(55.08-103.5) | 98.94(61.31-143.35) | 60.11(41.41-77.33) | -2.31(-2.57--2.05) |
| Maldives | Male | 41.04(26.32-63.43) | 112.09(77.68-143.46) | 73.4(48.96-112.72) | 57.71(39.96-73.88) | -1.4(-1.63--1.17) |
| Mali | Both | 2606.87(2000.68-3231.38) | 5250.77(3584.95-7326.85) | 59.74(46.23-74.12) | 57.88(40.16-78.93) | -0.27(-0.4--0.13) |
| Mali | Female | 1046.72(795.27-1317.99) | 1953.68(1236.92-2819.94) | 46.88(35.69-59.03) | 43.67(28.01-61.77) | -0.53(-0.65--0.4) |
| Mali | Male | 1560.15(1170.92-1995.66) | 3297.09(2151.51-4609.49) | 72.79(54.75-92.48) | 71.23(46.93-99.1) | -0.16(-0.33-0.02) |
| Malta | Both | 533.31(391.9-654.35) | 874.72(601.58-1106) | 124.76(91.39-153.27) | 99.41(68.45-125.64) | -0.65(-0.77--0.53) |
| Malta | Female | 247.91(179.99-308.12) | 345.73(232.1-445.31) | 104.88(75.81-130.34) | 74.11(50.26-95.07) | -1.18(-1.34--1.02) |
| Malta | Male | 285.4(210.94-350.48) | 528.99(372.74-662.52) | 150.93(110.98-185.33) | 128.26(90.93-161.42) | -0.34(-0.49--0.19) |
| Marshall Islands | Both | 18.67(14.35-23.26) | 44.45(30.69-60.49) | 102.07(79.51-126.73) | 112.99(80.23-151.64) | 0.37(0.33-0.41) |
| Marshall Islands | Female | 8.38(6.21-10.79) | 20.24(13.27-29.28) | 92.61(68.92-119.28) | 107.76(72.89-152.56) | 0.43(0.33-0.53) |
| Marshall Islands | Male | 10.29(7.64-13.2) | 24.21(16.52-33.95) | 112.02(82.98-143.86) | 118.09(81.58-163.35) | 0.3(0.18-0.43) |
| Mauritania | Both | 611.32(389.92-839.51) | 1077.22(667.2-1550.99) | 59.31(38.19-81.11) | 51.35(32.14-72.26) | -0.37(-0.53--0.21) |
| Mauritania | Female | 275.35(170.3-392.99) | 537.18(324.87-777.08) | 51.27(31.7-73.12) | 51.09(30.74-73.1) | 0.19(0.09-0.29) |
| Mauritania | Male | 335.97(209.36-454.85) | 540.04(337.02-791.16) | 68.17(42.97-92.18) | 51.5(32.3-73.85) | -0.91(-1.15--0.67) |
| Mauritius | Both | 519.4(395.69-620.49) | 1470.62(938.31-1955.44) | 67.05(51.1-80.12) | 84.14(54.04-111.51) | 0.76(0.65-0.87) |
| Mauritius | Female | 242.97(179.31-291.83) | 620.58(387.31-828.4) | 58.55(43.47-70.53) | 66.56(41.63-88.51) | 0.41(0.24-0.58) |
| Mauritius | Male | 276.43(211.66-334.03) | 850.05(553.95-1140.61) | 77(58.94-92.8) | 104.9(68.96-139.77) | 1.01(0.81-1.22) |
| Mexico | Both | 19881.3(14811.98-24324.51) | 76308.19(51999.62-99470.43) | 43.38(32.27-53.12) | 63.2(43.22-82.33) | 1.4(1.28-1.52) |
| Mexico | Female | 9681.27(7116.52-12038.22) | 32544.34(22128.43-44275.21) | 41.5(30.63-51.59) | 50.89(34.64-69.04) | 0.84(0.73-0.94) |
| Mexico | Male | 10200.03(7753.11-12342.34) | 43763.85(30071.68-58926.26) | 45.48(34.65-55.23) | 77.11(53-103.84) | 1.91(1.77-2.04) |
| Micronesia | Both | 56.23(39.78-74.84) | 92.27(58.06-130.46) | 109.29(79.54-144.54) | 120.02(78.06-166.28) | 0.26(0.22-0.3) |
| Micronesia | Female | 25.16(17.42-34.29) | 41.05(25.26-60.03) | 100.73(70.49-136.83) | 106.58(67.86-153.09) | 0.14(0.08-0.21) |
| Micronesia | Male | 31.07(21.07-42.61) | 51.22(30.11-77.97) | 117.32(83.21-159.74) | 133.27(82.63-196.13) | 0.36(0.33-0.39) |
| Moldova | Both | 6666.4(4665.7-8254.03) | 7565.89(5230.42-9810.89) | 144.7(101.29-178.57) | 131.84(91.05-170.72) | 0.36(-0.12-0.85) |
| Moldova | Female | 3157.02(2193.58-3981.59) | 2964.87(1994.99-3838.24) | 118.81(82.37-149.75) | 89.58(60.8-115.98) | -0.32(-0.79-0.16) |
| Moldova | Male | 3509.38(2436.03-4332.24) | 4601.01(3141.06-5922.13) | 181.09(126.53-222.69) | 189.2(129.12-242.72) | 0.85(0.35-1.35) |
| Mongolia | Both | 1224.54(943.52-1531.68) | 2529.57(1808.99-3448.77) | 108.78(83.71-135.8) | 96(70.06-128.6) | -1.04(-1.26--0.83) |
| Mongolia | Female | 555.98(416.82-708.22) | 1066.73(742.33-1467.33) | 93.84(70.59-119.44) | 76.17(53.94-102.82) | -1.25(-1.45--1.05) |
| Mongolia | Male | 668.56(496.22-865.33) | 1462.84(1041.98-2014.41) | 125.84(94.35-162.28) | 121.16(87.24-163.56) | -0.81(-1.04--0.58) |
| Montenegro | Both | 562.07(364.77-734.43) | 1008.2(620.36-1372.5) | 88.81(57.7-116.16) | 103.9(64.06-141.28) | 0.71(0.63-0.8) |
| Montenegro | Female | 226.58(146.38-300.56) | 390.2(242.92-527.16) | 65.51(42.52-86.7) | 75.06(46.68-101.42) | 0.7(0.56-0.83) |
| Montenegro | Male | 335.49(214.87-444.4) | 618(376.19-865.02) | 118.33(75.95-157.05) | 138.83(84.99-194.07) | 0.68(0.59-0.78) |
| Morocco | Both | 8691.05(6604.31-10709.92) | 23138.7(15711.18-31755.28) | 59.83(45.01-73.38) | 70.72(47.99-96.24) | 0.47(0.36-0.58) |
| Morocco | Female | 4559.99(3287.74-5753.4) | 11533.28(7875.17-16360.27) | 61.59(45.08-77.71) | 68.8(47.61-96.17) | 0.38(0.35-0.41) |
| Morocco | Male | 4131.06(3033.4-5327.15) | 11605.42(7428.63-16402.06) | 58.03(41.91-74.59) | 72.78(46.45-101.39) | 0.57(0.34-0.8) |
| Mozambique | Both | 2929.92(2248.3-3682.49) | 8917.66(6130.16-12166.3) | 47.23(36.9-58.37) | 76.51(53.77-102.4) | 2.12(1.95-2.29) |
| Mozambique | Female | 1484.84(1065-1967.48) | 3985.17(2581.29-5729.34) | 44.98(33.06-59) | 62.55(41.22-90.06) | 1.45(1.23-1.68) |
| Mozambique | Male | 1445.08(1093.55-1853.4) | 4932.49(3332.86-6874.68) | 49.66(38.15-63.34) | 93.91(63.94-128.88) | 2.77(2.58-2.97) |
| Myanmar | Both | 25496.6(18285.6-35481.35) | 57679.29(39836.94-77622.68) | 99.84(72.95-139.05) | 118.22(82.46-157.87) | 0.61(0.5-0.71) |
| Myanmar | Female | 12942.57(8948-17499.66) | 26443.31(17812.27-36665.42) | 95.61(68.14-127.38) | 98.15(67.04-135.3) | 0.03(-0.13-0.19) |
| Myanmar | Male | 12554.04(8674-21844.31) | 31235.97(21473.16-42069.42) | 104.91(73.77-181.98) | 143.6(98.16-190.01) | 1.18(1.11-1.26) |
| Namibia | Both | 408.44(300.06-528.38) | 1052.75(754.38-1440.9) | 54.81(40.51-70.34) | 72.04(52.71-96.13) | 0.98(0.83-1.13) |
| Namibia | Female | 199.43(140.74-270.91) | 481.17(320.04-685.07) | 49.84(35.73-67.12) | 58.7(39.48-82.56) | 0.59(0.48-0.7) |
| Namibia | Male | 209.01(143.15-279.95) | 571.58(414.08-771.04) | 60.67(42.15-80.46) | 88.98(65.77-115.77) | 1.37(1.16-1.58) |
| Nepal | Both | 3597.84(2306.68-5120.21) | 9554.33(6498.59-13796.05) | 34.88(22.35-49.45) | 42.28(29.02-60.81) | 0.66(0.4-0.92) |
| Nepal | Female | 1937.24(1201.89-2920.15) | 5119.75(3316.83-7397.82) | 37.64(22.85-55.42) | 42.75(27.65-61.27) | 0.39(0.16-0.62) |
| Nepal | Male | 1660.6(1006.28-2746.7) | 4434.57(2962.6-7210.03) | 32.19(19.66-52.83) | 41.61(27.93-67.52) | 0.93(0.64-1.22) |
| Netherlands | Both | 27543.77(19621-34271.46) | 42785.72(30115.62-53672.67) | 141.46(100.92-175.31) | 131.23(92.93-164.38) | -0.33(-0.42--0.24) |
| Netherlands | Female | 13144.77(9235.38-16438.46) | 18754.63(12934.98-23838.62) | 119.55(84.41-149) | 108.36(75.1-137.07) | -0.43(-0.5--0.35) |
| Netherlands | Male | 14399(10426.57-17822.86) | 24031.09(17176.18-30154.94) | 171.25(123.81-211.58) | 156.78(112.07-196.12) | -0.37(-0.49--0.25) |
| New Zealand | Both | 8990.54(6726.38-10829) | 12218.43(9239.95-14782.53) | 235.53(176.74-283.7) | 163.61(124.48-197.78) | -1.37(-1.45--1.29) |
| New Zealand | Female | 4313.64(3180.1-5247.67) | 5497.28(4087.2-6741.93) | 210.43(155.51-257.56) | 138.83(104.12-169.91) | -1.42(-1.5--1.33) |
| New Zealand | Male | 4676.9(3592.38-5638.66) | 6721.15(5184.33-8089.94) | 265.96(204.05-321.1) | 191.25(147.55-230.36) | -1.37(-1.47--1.26) |
| Nicaragua | Both | 872.97(686.54-1052.06) | 3324.1(2421.73-4272.71) | 52.6(40.53-63.94) | 73.83(54.34-93.83) | 1.21(1.05-1.38) |
| Nicaragua | Female | 443.99(338.56-551) | 1593.12(1168.64-2047.11) | 50.06(37.31-64.22) | 65.18(47.9-83) | 0.87(0.65-1.08) |
| Nicaragua | Male | 428.98(337.7-532.71) | 1730.98(1229.1-2273.81) | 55.71(43.49-69.51) | 83.98(60.22-109.42) | 1.56(1.35-1.77) |
| Niger | Both | 1447.4(1032.98-1991.54) | 3414.23(2326.18-4817.93) | 48.03(34.81-65.3) | 42.21(29.1-58.13) | -0.54(-0.71--0.37) |
| Niger | Female | 592.99(394.54-843.83) | 1672.76(1077-2400.46) | 40.67(26.96-56.98) | 40.23(26.68-57.07) | -0.23(-0.36--0.09) |
| Niger | Male | 854.41(565.68-1269.53) | 1741.47(1089.62-2675.63) | 54.49(36.56-80.04) | 44.18(28.22-67.41) | -0.74(-0.94--0.54) |
| Nigeria | Both | 24253.11(16845.77-34381.01) | 58768.72(41236.28-79337.08) | 54.42(38.08-76.53) | 66.35(47.78-88.23) | 1.03(0.86-1.2) |
| Nigeria | Female | 11227.82(7210.56-17130.92) | 30392.78(20273.51-43295.89) | 53.12(34.42-80.17) | 65.86(44.72-91.44) | 1.12(0.92-1.33) |
| Nigeria | Male | 13025.29(7904.01-19727.34) | 28375.94(18767.35-42059.08) | 55.01(33.85-82.07) | 67.08(45.03-98.27) | 1.02(0.87-1.18) |
| North Korea | Both | 18018.62(12620.12-24922.49) | 36065.28(25115.13-49352.44) | 101.59(72.42-137.4) | 109.95(76.71-150.51) | 0.4(0.33-0.46) |
| North Korea | Female | 8927.32(5853.35-12562.38) | 15691.44(10377.89-22170.31) | 87.92(58.78-121.71) | 86.61(57.82-123.89) | 0.06(0-0.12) |
| North Korea | Male | 9091.31(6114.94-13196.29) | 20373.84(13546.44-30015.95) | 121.92(86.22-171.57) | 139.18(95.13-201.32) | 0.61(0.53-0.68) |
| Northern Mariana Islands | Both | 32.59(21.58-44.26) | 73(52.12-94.5) | 142.44(97.59-187.03) | 134.78(97.12-171.85) | -0.15(-0.29-0) |
| Northern Mariana Islands | Female | 10.84(6.92-15.52) | 20.02(14.14-26.87) | 122.66(80.04-171.34) | 78.65(55.66-105.73) | -1.7(-1.89--1.52) |
| Northern Mariana Islands | Male | 21.75(14.46-30.04) | 52.98(37.71-68.31) | 156.28(106.33-204.86) | 189.4(136.84-240.64) | 0.82(0.65-0.98) |
| Norway | Both | 10263.99(7438.63-12597.05) | 12160.44(8893.63-15001.11) | 159.37(115.49-195.88) | 131.56(96.24-162.23) | -0.64(-0.78--0.5) |
| Norway | Female | 4798.89(3378.92-5978.08) | 5567.12(3978.5-6959.79) | 134.23(95.56-167.09) | 113.89(81.51-142.27) | -0.57(-0.73--0.41) |
| Norway | Male | 5465.1(3986.16-6671.75) | 6593.32(4832.97-8092.87) | 191.6(140.04-233.35) | 151.14(111.13-185.49) | -0.79(-0.93--0.65) |
| Oman | Both | 427.79(262.71-614.27) | 1049.4(645.31-1494.54) | 57.89(36.03-82.26) | 55.64(34.96-75.86) | 0.14(-0.07-0.35) |
| Oman | Female | 187.46(108.08-277.99) | 422.25(248.83-614.73) | 60.77(35.62-88.84) | 56.99(34.34-80.24) | 0.05(-0.11-0.21) |
| Oman | Male | 240.33(140.22-360.27) | 627.15(370.03-959.49) | 55.61(33.14-82.5) | 55.49(34.2-76.59) | 0.28(0.03-0.53) |
| Pakistan | Both | 32459.53(25477.27-39300.74) | 95123.57(68324.5-124661.26) | 54.03(42.74-65.21) | 77.01(55.71-99.81) | 1.15(0.88-1.41) |
| Pakistan | Female | 12985.18(9643.08-16332.82) | 39340.73(27130.01-55154.81) | 46.77(34.76-58.58) | 64.94(45.31-90.16) | 1.03(0.78-1.27) |
| Pakistan | Male | 19474.35(15107.9-24021.13) | 55782.84(36775.72-78635.5) | 59.87(46.43-73.95) | 88.31(58.86-122.95) | 1.29(1-1.57) |
| Palestine | Both | 1369.58(919.05-1929.68) | 4787.77(3781.35-5836.04) | 151.01(101.39-213.2) | 187.93(148.7-228.82) | 0.92(0.68-1.17) |
| Palestine | Female | 720.27(467.86-1027.42) | 2265.95(1769.3-2844.17) | 143.26(93.79-201.33) | 170.43(133.28-213.3) | 0.83(0.57-1.1) |
| Palestine | Male | 649.31(400.09-978.45) | 2521.83(1943.15-3091.47) | 160.67(99.45-240.68) | 210.81(164.03-258.16) | 1.05(0.83-1.28) |
| Panama | Both | 1182.7(954.62-1390.33) | 3279.41(2219.52-4482.56) | 76.58(61.83-90.02) | 78.9(53.39-107.76) | 0.29(0.21-0.37) |
| Panama | Female | 567.12(452.34-672.87) | 1505.6(1015.28-2089.17) | 73.81(59.08-87.95) | 70.49(47.4-97.84) | 0.01(-0.1-0.12) |
| Panama | Male | 615.58(492.85-723.15) | 1773.81(1197.15-2455.6) | 79.15(64-93.02) | 87.63(59.44-121.02) | 0.55(0.45-0.66) |
| Papua New Guinea | Both | 1234.34(792.13-1718.25) | 3806.18(2590.26-5366.59) | 59.3(38.36-82.56) | 69.78(48.15-96.79) | 0.52(0.5-0.55) |
| Papua New Guinea | Female | 551.18(325.76-788.16) | 1662.01(1104.79-2425.05) | 55.48(33.11-79.07) | 65.05(44.36-93.31) | 0.52(0.5-0.55) |
| Papua New Guinea | Male | 683.16(416.88-1041.14) | 2144.17(1451.11-3116.35) | 63.04(39.31-94) | 74.13(50.56-105.11) | 0.5(0.48-0.53) |
| Paraguay | Both | 1198.04(932.43-1453.98) | 5110.37(3338.85-7058.99) | 51.58(40.32-62.78) | 89.43(58.37-122.76) | 2.14(1.97-2.31) |
| Paraguay | Female | 606.95(459.27-750.46) | 2160.27(1388.13-3030.47) | 50.28(38.14-62.38) | 73.28(46.99-102.32) | 1.63(1.48-1.78) |
| Paraguay | Male | 591.09(457.18-729.17) | 2950.1(1948.17-4072.84) | 52.88(40.77-65.23) | 106.35(70.72-145.88) | 2.57(2.36-2.79) |
| Peru | Both | 7852.27(6057.61-9743.74) | 19522.18(12575.68-27168.03) | 62.9(48.54-77.93) | 60.33(38.85-84.23) | -0.11(-0.32-0.11) |
| Peru | Female | 3830.95(2925.26-4792.38) | 9582.65(6043.71-13304.06) | 60.33(45.96-74.87) | 57.15(36.24-79.81) | -0.37(-0.63--0.12) |
| Peru | Male | 4021.32(3091.46-5039.84) | 9939.53(6406-14346.42) | 65.49(50.5-82.47) | 63.6(41.09-91.93) | 0.18(-0.03-0.38) |
| Philippines | Both | 46817.93(38273.15-55686.75) | 132088.29(100802.02-168217.92) | 136.22(110.8-161.33) | 153.66(117.88-194.97) | 0.24(-0.02-0.49) |
| Philippines | Female | 19709.96(15730.06-23874.25) | 52106.95(37222.04-70090.73) | 116.55(93.38-141.33) | 118.11(85.07-157.7) | -0.07(-0.36-0.22) |
| Philippines | Male | 27107.97(21529.14-32959.42) | 79981.33(57191.53-107925.09) | 156.5(123.85-191.36) | 192.65(138.42-259.5) | 0.51(0.27-0.75) |
| Poland | Both | 65361.39(46216.6-80381.5) | 106433.35(71720.73-138838.36) | 149.78(106.13-184.22) | 157.53(105.97-205.57) | 0.11(-0.04-0.26) |
| Poland | Female | 30754.94(21303.25-38186.66) | 42643.73(28643.29-57996.21) | 122.33(84.92-151.73) | 110.65(74.68-150.92) | -0.47(-0.59--0.35) |
| Poland | Male | 34606.45(24545.56-42391.72) | 63789.62(41068.44-87301.2) | 187.28(132.93-229.91) | 218.42(141.08-298.16) | 0.52(0.33-0.71) |
| Portugal | Both | 19080.89(13324.94-23837.57) | 30524.62(21689.35-37631.84) | 141.66(99.34-176.06) | 139.59(99.92-171.01) | -0.06(-0.31-0.2) |
| Portugal | Female | 8621.06(5954.57-10918.4) | 11971.21(8359.88-15086.23) | 114.3(79.2-144.19) | 98(69.93-121.66) | -0.57(-0.78--0.35) |
| Portugal | Male | 10459.83(7340.76-13069.66) | 18553.41(13117.83-22954.53) | 176.9(124.94-220.41) | 191.32(135.57-236.38) | 0.28(-0.02-0.57) |
| Puerto Rico | Both | 3449.09(2459.49-4202.64) | 6662.79(4190.06-9221.36) | 95.58(68.18-116.33) | 104.56(65.08-144.87) | 0.21(0.02-0.39) |
| Puerto Rico | Female | 1470.88(1015.32-1836.63) | 2787.37(1688.47-3813.38) | 75.74(52.3-94.21) | 77.96(47.18-106.73) | -0.1(-0.29-0.09) |
| Puerto Rico | Male | 1978.21(1411.82-2406.74) | 3875.42(2469.97-5487.42) | 118.32(84.68-143.73) | 136.35(86.9-192.58) | 0.45(0.24-0.66) |
| Qatar | Both | 98.08(62.45-138.15) | 683.34(385.99-984.08) | 75.85(48.17-105.97) | 74.15(42.76-103.39) | 0.27(-0.01-0.55) |
| Qatar | Female | 46.44(28.53-67.39) | 245.59(140.49-350.82) | 106.47(62.87-152.21) | 111.81(63.7-152.47) | 0.62(0.31-0.93) |
| Qatar | Male | 51.64(31.09-77.25) | 437.75(241.24-658.27) | 59.62(36.37-87.48) | 62.52(35.92-89.39) | 0.47(0.16-0.79) |
| Republic of Congo | Both | 1346.04(834.83-1884.61) | 3067.33(2063.2-4283.69) | 116.43(74.8-161.23) | 105.36(72.76-142.98) | -0.46(-0.66--0.25) |
| Republic of Congo | Female | 597.87(345.49-911.08) | 1434.87(896.92-2149.63) | 94.6(56.28-141.95) | 95.19(62.2-138.8) | 0.07(-0.06-0.2) |
| Republic of Congo | Male | 748.17(469.13-1062.32) | 1632.46(1081.47-2291.04) | 143.98(94.2-202.86) | 117.07(79.88-159.82) | -0.98(-1.27--0.69) |
| Romania | Both | 26239.41(17574.41-33223.86) | 46116.46(29968.33-61183.57) | 93.16(62.71-118.03) | 134.49(87.43-179.19) | 1.07(0.84-1.31) |
| Romania | Female | 11452.12(7499.7-14538.51) | 17315.36(10853.9-23419.17) | 75.55(49.69-95.83) | 91.08(57.62-123.1) | 0.44(0.26-0.62) |
| Romania | Male | 14787.29(10066.48-18682.81) | 28801.1(18714.18-38241.31) | 114.38(77.92-144.41) | 187.88(122.31-250.8) | 1.53(1.26-1.81) |
| Russia | Both | 276233.84(205016.16-334252) | 295968.06(207322.13-383462.77) | 151.14(112.12-182.73) | 128.65(90.34-166.63) | -1.17(-1.55--0.8) |
| Russia | Female | 152830.56(112297.47-187498.08) | 148910.69(100556.06-200413.66) | 133.86(98.46-164.72) | 106.4(71.69-143.22) | -1.44(-1.78--1.09) |
| Russia | Male | 123403.28(92917.85-148780.35) | 147057.37(102521.55-192390.64) | 188.85(142.34-227.14) | 164.37(114.64-214.32) | -1.06(-1.46--0.66) |
| Rwanda | Both | 2515.23(1673.05-3326.12) | 4519.75(3101.1-6114.62) | 79.01(52.82-104.05) | 68.63(48.3-91.02) | -1.07(-1.33--0.8) |
| Rwanda | Female | 1226.16(685.49-1793.9) | 2261.36(1559.62-3028.51) | 68.72(39.04-100.55) | 60.64(42.89-80.58) | -0.84(-1.07--0.61) |
| Rwanda | Male | 1289.07(898.11-1686.69) | 2258.38(1460.9-3277.86) | 92.14(64.77-119.73) | 79.96(53.08-112.7) | -1.23(-1.53--0.93) |
| Saint Lucia | Both | 83.79(62.74-101.42) | 193.81(136.7-247.88) | 95.83(72.15-115.36) | 90.07(63.72-115.39) | -0.41(-0.68--0.14) |
| Saint Lucia | Female | 42.56(30.66-52.3) | 82.75(56.56-107.78) | 87.9(63.66-107.93) | 72.91(49.7-94.74) | -0.98(-1.31--0.65) |
| Saint Lucia | Male | 41.23(31.25-49.66) | 111.06(80.29-142.57) | 106.3(80.7-127.84) | 108.75(78.87-139.59) | 0.01(-0.22-0.24) |
| Saint Vincent | Both | 72.87(57.48-85.28) | 141.25(100.18-177.15) | 101.36(80.46-118.76) | 105.14(74.55-131.82) | -0.17(-0.42-0.07) |
| Saint Vincent | Female | 38.45(29.99-45.43) | 60.09(41.86-75.72) | 97.62(76.06-115.46) | 91.97(64.15-115.78) | -0.48(-0.77--0.19) |
| Saint Vincent | Male | 34.42(27.18-40.91) | 81.16(58.22-102.46) | 105.39(83.28-124.61) | 117.37(84.4-147.22) | 0.04(-0.19-0.27) |
| Samoa | Both | 83.9(61.53-108.28) | 128.82(90.04-169.8) | 92.74(69-118.72) | 85.14(60.28-111.49) | -0.43(-0.49--0.37) |
| Samoa | Female | 43.63(30.99-59.29) | 71.67(47.27-98.61) | 94.88(67.92-128.06) | 93.82(62.47-128.31) | -0.15(-0.22--0.08) |
| Samoa | Male | 40.28(30.02-51.69) | 57.15(39.52-80.37) | 90.66(68.18-114.5) | 76.81(54.01-106.49) | -0.74(-0.82--0.67) |
| Sao Tome and Principe | Both | 53.87(39.46-66.47) | 132.57(87.4-187.64) | 83.56(61.78-102.56) | 122.6(82.09-173.2) | 1.33(1.26-1.4) |
| Sao Tome and Principe | Female | 16.31(11.82-20.62) | 37.7(24.1-54.83) | 48.47(35.4-60.97) | 64.8(41.43-94.34) | 0.86(0.74-0.97) |
| Sao Tome and Principe | Male | 37.56(27.43-47.21) | 94.87(61.1-139.48) | 124.19(92.29-155.29) | 186.32(120.51-274.18) | 1.5(1.42-1.58) |
| Saudi Arabia | Both | 3257.9(2009.19-4751.74) | 15722.37(9756.81-22139.35) | 48.56(30.13-70.17) | 65.58(41.41-89.52) | 0.98(0.78-1.18) |
| Saudi Arabia | Female | 1394.9(800.98-2100.7) | 6353.93(3929.55-8947.82) | 51.04(29.66-76.5) | 64.97(39.94-89.58) | 0.79(0.54-1.04) |
| Saudi Arabia | Male | 1862.99(1117.56-2807.7) | 9368.44(5914.5-13444.62) | 46.97(28.63-69.72) | 65.93(41.6-92.44) | 1.1(0.91-1.3) |
| Senegal | Both | 2344.38(1729.68-2923.67) | 5390.23(3763.82-7155.05) | 69.76(52.5-86.59) | 69.83(50.17-91.38) | 0.09(-0.11-0.29) |
| Senegal | Female | 929.67(672.62-1209.87) | 2419.37(1698.66-3341.37) | 55.28(40.15-71.03) | 60.52(43.18-82.45) | 0.44(0.23-0.64) |
| Senegal | Male | 1414.71(1031.86-1870.47) | 2970.86(2003.24-4010.81) | 83.65(61.76-109.68) | 79.66(54.9-106.06) | -0.11(-0.31-0.09) |
| Serbia | Both | 16986.49(11403.57-21909.63) | 25365.08(16042.49-34701.36) | 148.39(99.83-191.11) | 166.44(105.38-227.68) | 0.41(0.24-0.58) |
| Serbia | Female | 7236.4(4758.6-9593.83) | 9615.68(5937.06-13311.37) | 121.11(80.04-161.55) | 119.27(73.71-165.46) | -0.16(-0.33-0.01) |
| Serbia | Male | 9750.09(6501.35-12904.21) | 15749.4(9817.47-21199.96) | 180.14(120.74-236.61) | 222.13(140.29-298.3) | 0.84(0.65-1.03) |
| Seychelles | Both | 74.55(58.66-90.1) | 195.89(143.43-246.99) | 131.92(103.73-159.28) | 174.19(128.81-218.18) | 0.61(0.39-0.82) |
| Seychelles | Female | 35.31(27.18-43.16) | 78.96(55.24-103.23) | 113.62(86.71-138.93) | 137.66(96.63-179.71) | 0.61(0.48-0.74) |
| Seychelles | Male | 39.23(30.73-48.78) | 116.93(84.96-151.21) | 154.75(121.14-192.34) | 211.46(154.74-269.31) | 0.49(0.19-0.8) |
| Sierra Leone | Both | 1169.1(857.79-1493.27) | 2368.59(1631.1-3191.22) | 59.47(44.34-75.61) | 62.38(43.73-83.39) | 0.35(0.19-0.51) |
| Sierra Leone | Female | 441.53(316.88-589.68) | 1113.46(775.24-1517.03) | 46.04(33.11-60.82) | 58.63(41.02-79.77) | 1.1(0.99-1.22) |
| Sierra Leone | Male | 727.57(527.57-946.46) | 1255.13(834.66-1744.63) | 72.06(52.56-93.79) | 65.94(44.64-91.62) | -0.19(-0.37--0.01) |
| Singapore | Both | 4008.28(2946.13-4977.06) | 7138.8(4954.06-9049.11) | 172.98(127.04-215) | 90.51(63.13-114.61) | -2.48(-2.57--2.39) |
| Singapore | Female | 1765.3(1277.68-2219.09) | 3099.28(2158.18-3984.65) | 142.29(102.99-179.01) | 77.05(53.73-99.14) | -2.32(-2.42--2.21) |
| Singapore | Male | 2242.98(1660.45-2765.57) | 4039.52(2831.47-5145.09) | 210.2(155.16-259.55) | 104.75(72.97-133.55) | -2.67(-2.77--2.57) |
| Slovakia | Both | 11189.87(8135.04-13779.37) | 18180.01(12144.24-24482.81) | 188.01(136.49-231.51) | 200.45(133.68-271.34) | 0.21(0.04-0.39) |
| Slovakia | Female | 4447.01(3165.32-5538.92) | 6999.07(4646.11-9602.92) | 132.43(94.07-165.02) | 137.96(92.35-190.09) | 0.09(-0.05-0.23) |
| Slovakia | Male | 6742.86(4934.02-8296.65) | 11180.94(7487.94-15193.2) | 260.7(191.23-321.53) | 283.98(190.07-385.01) | 0.33(0.11-0.55) |
| Slovenia | Both | 3666.38(2386.53-5015.24) | 5033.87(3348.9-6907.55) | 150.59(98.3-205.25) | 122.42(81.49-169.02) | -1(-1.31--0.68) |
| Slovenia | Female | 1728.13(1121.65-2391.77) | 1869.24(1234.12-2605.18) | 120.43(77.59-166.78) | 82.04(53.43-114.65) | -1.6(-1.88--1.32) |
| Slovenia | Male | 1938.24(1260.19-2642.99) | 3164.63(2152.46-4350.41) | 195.82(127.87-266.83) | 171.55(116.27-236.15) | -0.76(-1.12--0.39) |
| Solomon Islands | Both | 138.78(80.27-205.6) | 369.72(220.22-531.6) | 84.74(51.69-124.04) | 97.26(61.15-136.24) | 0.39(0.3-0.48) |
| Solomon Islands | Female | 58.68(29.41-101.82) | 162.35(86.46-249.04) | 78.19(40.86-134.66) | 88.12(50.38-130.52) | 0.32(0.19-0.45) |
| Solomon Islands | Male | 80.09(46.14-126.63) | 207.37(122.99-325.27) | 90.13(55.63-140.05) | 106.14(66.64-162.05) | 0.49(0.42-0.55) |
| Somalia | Both | 1651.51(1092.53-2548.48) | 4136.47(2494.51-7961.35) | 55.87(37.84-86.42) | 53.43(32.55-102.36) | -0.01(-0.07-0.05) |
| Somalia | Female | 743.09(445.33-1097.1) | 2003.2(1135.17-3663.07) | 46.71(28.75-69.38) | 47.02(26.92-86.94) | 0.26(0.16-0.35) |
| Somalia | Male | 908.42(569.84-1696) | 2133.26(1239.85-4442.14) | 66.38(43.57-122.87) | 62.36(36.68-127.61) | -0.15(-0.19--0.1) |
| South Africa | Both | 18509.96(14490.08-23017.23) | 41050.64(32253.52-50556.25) | 82.8(63.92-105.38) | 88.42(69.39-109.01) | 0.3(0.06-0.55) |
| South Africa | Female | 8764.85(6839.28-11010.86) | 18037.15(13666.23-22878.51) | 70.1(53.92-89.73) | 68.95(52.26-87.62) | 0.11(-0.08-0.29) |
| South Africa | Male | 9745.11(7461.79-12794.35) | 23013.49(17957.1-28301.51) | 98.81(75.11-134.16) | 114.4(89.64-140.99) | 0.52(0.2-0.85) |
| South Korea | Both | 28024.37(21149.16-34666.23) | 82092.55(60027.68-102755.64) | 86.69(65.41-106.24) | 93.68(68.58-116.26) | -0.05(-0.41-0.31) |
| South Korea | Female | 12804.43(9559.74-16033.15) | 32667.96(23671.47-41524.8) | 70.82(52.87-88.15) | 68.43(49.45-86.43) | -0.61(-0.88--0.34) |
| South Korea | Male | 15219.94(11599.44-18618.94) | 49424.59(36364.93-61697.51) | 111.24(85.12-136.42) | 123.77(90.8-153.96) | 0.17(-0.27-0.61) |
| South Sudan | Both | 2091.15(1163.63-3591.03) | 3258.31(1987.18-5076.29) | 82.95(46.35-139.67) | 78.04(48.47-120.24) | -0.2(-0.29--0.11) |
| South Sudan | Female | 834.63(419.38-1591.4) | 1434.61(810.55-2359.51) | 73.42(37.37-139.52) | 69.45(40.16-113.03) | -0.14(-0.26--0.02) |
| South Sudan | Male | 1256.52(650.29-2223.09) | 1823.71(1019.03-2985.62) | 90.44(47.75-158.78) | 85.47(48.49-138.71) | -0.2(-0.29--0.12) |
| Spain | Both | 74105.68(55193.09-90014.72) | 119895.38(86687.32-149964.76) | 140.8(105.07-170.76) | 132.72(96.51-165.55) | -0.22(-0.28--0.16) |
| Spain | Female | 33402.31(24520.46-40987.27) | 46764.6(33385.82-58626.95) | 114.35(84.1-139.66) | 93.25(66.9-115.93) | -0.83(-0.92--0.74) |
| Spain | Male | 40703.36(30513.29-49260.41) | 73130.79(53432.17-91835.48) | 175.66(132.29-212.98) | 180.04(131.58-224.67) | 0.13(0.04-0.22) |
| Sri Lanka | Both | 4742.33(3843.5-5637.24) | 12023.18(8280.16-16571.91) | 41.28(33.5-49.02) | 47.38(32.92-65.26) | 0.73(0.6-0.85) |
| Sri Lanka | Female | 2187.47(1730.48-2652.72) | 5978.58(4112.01-8366.63) | 38.44(30.36-46.65) | 42.88(29.55-59.13) | 0.51(0.42-0.61) |
| Sri Lanka | Male | 2554.86(2019.33-3095.66) | 6044.6(4045.25-8530.92) | 44.08(34.86-53.13) | 52.56(35.47-73.41) | 0.97(0.81-1.14) |
| Sudan | Both | 5121.13(3582.86-7684.57) | 13592.74(8969.74-20635.37) | 50.9(35.96-77.25) | 65.61(44.31-99.61) | 0.95(0.86-1.03) |
| Sudan | Female | 2478.13(1697.77-3341.61) | 6084.14(3974.67-9077.71) | 50.49(35.33-67.34) | 60.65(40.78-87.65) | 0.73(0.66-0.8) |
| Sudan | Male | 2643(1717.49-4829.36) | 7508.61(4696.57-13382.25) | 51.02(33.43-92.47) | 69.55(44.55-123.75) | 1.11(1.02-1.21) |
| Suriname | Both | 268.73(209.74-321.05) | 819.51(606.86-1040.68) | 100.54(79.03-120.31) | 134.74(99.97-171.37) | 1(0.77-1.24) |
| Suriname | Female | 121.81(94.84-148.56) | 346.6(253.33-442.95) | 88.32(69.07-107.95) | 107.1(78.31-136.61) | 0.61(0.42-0.8) |
| Suriname | Male | 146.93(113.68-177.95) | 472.91(349.74-614.01) | 113.67(88.55-137.7) | 166.15(123.95-215.2) | 1.34(1.06-1.62) |
| Sweden | Both | 16972.49(12107.85-20929.88) | 19811.98(14106.65-24560.41) | 119.81(86.24-147.59) | 101.36(72.43-125.31) | -0.66(-0.78--0.53) |
| Sweden | Female | 8125.27(5748.79-10119.62) | 8972.8(6210.78-11317.77) | 104.66(74.19-129.32) | 87.38(61.01-109.63) | -0.65(-0.77--0.54) |
| Sweden | Male | 8847.22(6311.45-10890.77) | 10839.18(7756.91-13392.28) | 139.25(100.35-171.18) | 116.52(84.58-143.38) | -0.75(-0.89--0.61) |
| Switzerland | Both | 9961.22(7303.01-12112.66) | 13598.78(9763.13-16624.91) | 99.72(73.37-120.82) | 82.82(59.61-100.76) | -0.87(-1.06--0.68) |
| Switzerland | Female | 4277.44(3090.45-5257.76) | 5724.26(4075.76-7104.11) | 74.78(54.35-91.62) | 64.92(45.86-80.08) | -0.69(-0.87--0.5) |
| Switzerland | Male | 5683.78(4180.38-6916.99) | 7874.52(5688.37-9678.25) | 132.16(96.86-160.59) | 103.3(75.05-126.72) | -1.12(-1.33--0.9) |
| Syria | Both | 2738.81(1920.91-3649.98) | 6128.42(3891.34-8856.13) | 46.54(32.23-61.37) | 48.58(31.13-69.13) | -0.11(-0.38-0.17) |
| Syria | Female | 1267.59(831.14-1730.17) | 2741.35(1724.3-4081.91) | 45.24(30-61.17) | 45.22(28.84-65.94) | -0.21(-0.5-0.07) |
| Syria | Male | 1471.22(1004.85-2044.34) | 3387.07(2112.71-4977.02) | 47.73(32.66-66.18) | 52.5(33.12-75.55) | 0.04(-0.23-0.31) |
| Taiwan | Both | 20623.6(14981.4-25384.42) | 76257.51(52148.16-105982.05) | 120.84(87.86-148.42) | 198.77(135.94-276.23) | 1.96(1.73-2.2) |
| Taiwan | Female | 7758.95(5527.93-9704.85) | 27401.48(18578.58-38496.4) | 97.07(69.34-121.52) | 135.22(91.87-189.97) | 1.26(1.06-1.46) |
| Taiwan | Male | 12864.65(9422.46-15739.89) | 48856.03(33469.12-68108.3) | 142.68(105.14-173.85) | 268.76(184.43-373.54) | 2.54(2.26-2.82) |
| Tajikistan | Both | 3349.08(2667.08-3965.42) | 5287.42(3950.21-6819.75) | 107.86(86.45-127.62) | 96.32(72.53-123) | -0.08(-0.48-0.31) |
| Tajikistan | Female | 1430.81(1131.13-1715.74) | 2157.63(1599.19-2879.81) | 88.08(69.72-106.15) | 79.85(59.83-104.65) | 0(-0.37-0.38) |
| Tajikistan | Male | 1918.26(1526.94-2314.65) | 3129.79(2345.83-4076.97) | 129.94(103.54-156.47) | 113.88(86.01-145.99) | -0.14(-0.57-0.28) |
| Tanzania | Both | 7755.3(5813.84-10001.67) | 20707.8(14756.31-28356.11) | 66.81(50.53-85.47) | 77.42(55.89-104.39) | 0.55(0.48-0.62) |
| Tanzania | Female | 3553.37(2647.76-4553.45) | 9820.5(7070.29-12939.22) | 59.01(44.26-74.88) | 70.09(50.86-90.82) | 0.72(0.57-0.88) |
| Tanzania | Male | 4201.93(3051.14-5940.06) | 10887.3(7237.75-16210.82) | 75.11(55.38-104.05) | 85.52(57.68-124.68) | 0.41(0.36-0.47) |
| Thailand | Both | 41671.59(33800.07-49483.69) | 93427.04(64211.45-128666.31) | 106.61(86.69-126.36) | 92.38(63.77-126.98) | -1.14(-1.46--0.82) |
| Thailand | Female | 17734.77(14135.63-21495) | 38806.95(26667.69-53924.02) | 87.22(69.52-105.46) | 70.95(48.69-98.1) | -1.16(-1.43--0.88) |
| Thailand | Male | 23936.82(18969.34-29119.82) | 54620.09(37414.85-76315.7) | 128.35(101.66-155.73) | 116.74(80.39-162.47) | -1.12(-1.49--0.75) |
| Timor-Leste | Both | 243.84(168.12-345.19) | 927.07(588.81-1276.22) | 71.65(50.52-99.07) | 110.48(70.92-150.71) | 1.83(1.58-2.09) |
| Timor-Leste | Female | 134.12(87.11-208.92) | 445.82(283.72-614.96) | 80.43(54.02-120.53) | 106.47(67.5-146.04) | 1.2(0.97-1.43) |
| Timor-Leste | Male | 109.71(64.53-166.18) | 481.24(275.46-669.55) | 63.32(38.41-94.59) | 114.86(66.07-159.61) | 2.52(2.22-2.81) |
| Tobago | Both | 945.99(669.72-1142.98) | 1794.78(1119.75-2470.78) | 109.95(78-133.17) | 97.34(61.01-133.38) | -0.84(-1.04--0.65) |
| Tobago | Female | 449.5(314.07-550.29) | 740.2(449.23-1041.37) | 99.96(69.74-122.21) | 77.83(47.25-109.65) | -1.34(-1.53--1.16) |
| Tobago | Male | 496.49(359.31-597.96) | 1054.58(672.27-1475.08) | 120.21(86.86-144.75) | 117.67(75.96-164.34) | -0.45(-0.71--0.18) |
| Togo | Both | 855.08(652.97-1091.65) | 2614.76(1760.34-3611.83) | 63.69(49.05-80.87) | 66.89(45.7-90.93) | 0.09(0.04-0.15) |
| Togo | Female | 387.72(289.14-499.04) | 1216.7(795.25-1720.2) | 54.12(40.72-68.6) | 55.8(37.09-78.66) | 0.11(-0.02-0.24) |
| Togo | Male | 467.36(346.51-615.23) | 1398.06(911.82-2020.4) | 74.09(55.45-96.27) | 81.79(55.26-115.71) | 0.23(0.14-0.32) |
| Tonga | Both | 29.47(22.27-36.83) | 45.14(32.39-60.34) | 51.9(39.47-64.58) | 56.46(40.72-75.12) | 0.18(0.06-0.31) |
| Tonga | Female | 16.81(12.4-21.06) | 24.64(17.03-33.68) | 57.22(42.65-71.57) | 57.48(39.82-78.54) | -0.1(-0.22-0.02) |
| Tonga | Male | 12.66(9-16.66) | 20.51(14.47-27.51) | 45.98(33.04-60.17) | 54.85(39.17-73.42) | 0.5(0.26-0.73) |
| Trinidad | Both | 945.99(669.72-1142.98) | 1794.78(1119.75-2470.78) | 109.95(78-133.17) | 97.34(61.01-133.38) | -0.84(-1.04--0.65) |
| Trinidad | Female | 449.5(314.07-550.29) | 740.2(449.23-1041.37) | 99.96(69.74-122.21) | 77.83(47.25-109.65) | -1.34(-1.53--1.16) |
| Trinidad | Male | 496.49(359.31-597.96) | 1054.58(672.27-1475.08) | 120.21(86.86-144.75) | 117.67(75.96-164.34) | -0.45(-0.71--0.18) |
| Tunisia | Both | 3033.41(2122.56-3881.46) | 7958.09(4751.69-11396.48) | 59.22(41.63-75.73) | 62.56(37.84-89.43) | 0.02(-0.05-0.09) |
| Tunisia | Female | 1592.58(1079.41-2064.92) | 3958.02(2401.82-5777.58) | 63.12(42.74-82.03) | 60.37(36.57-87.8) | -0.24(-0.28--0.19) |
| Tunisia | Male | 1440.82(1010.15-1888.46) | 4000.07(2346.8-5873.15) | 55.34(38.96-72.35) | 64.98(38.8-94.86) | 0.3(0.21-0.4) |
| Turkey | Both | 36539.94(23837.31-49976.88) | 75429.71(48718.31-100549.68) | 94.81(61.85-128.57) | 84.16(54.32-111.98) | -0.25(-0.69-0.19) |
| Turkey | Female | 16045.35(10249.23-22549.89) | 30193.88(19314.89-41109.01) | 81.09(52.11-113.3) | 64.58(41.26-87.88) | -0.72(-1.12--0.32) |
| Turkey | Male | 20494.59(12956.49-28911.9) | 45235.83(29047.3-60672.65) | 109.44(69.74-153.94) | 105.43(67.79-140.36) | 0.12(-0.33-0.58) |
| Turkmenistan | Both | 1733.83(1312.11-2070.6) | 2639.53(1883.17-3522.38) | 78.99(59.81-94.49) | 63.08(44.99-83.61) | -1.11(-1.66--0.56) |
| Turkmenistan | Female | 802.35(606.92-961.64) | 1217.56(866.27-1651.44) | 67(50.31-80.41) | 54.74(39.17-73.8) | -1.2(-1.7--0.7) |
| Turkmenistan | Male | 931.48(714.4-1110.12) | 1421.97(1000.05-1895.28) | 94.37(72.12-112.85) | 73.21(51.82-96.4) | -1.04(-1.65--0.42) |
| Uganda | Both | 3757.12(2815.56-4722.73) | 14518.25(10219.13-19197.38) | 54.85(41.69-68.55) | 92.28(66.09-120.81) | 1.72(1.46-1.98) |
| Uganda | Female | 1432.7(1004.96-1905.51) | 6216.32(4261.16-8370.65) | 40.62(28.99-52.75) | 72.19(50.48-96.16) | 1.98(1.66-2.3) |
| Uganda | Male | 2324.42(1706.78-2970.11) | 8301.94(5544-11158.58) | 69.81(51.92-88.28) | 117.69(80.34-158.3) | 1.69(1.45-1.93) |
| UK | Both | 146002.9(105733.69-178945.19) | 133811.97(92634.13-164360.63) | 168.45(121.96-206.05) | 112.74(78.31-138.12) | -1.62(-1.81--1.43) |
| UK | Female | 68391.09(48548.91-84211) | 57524.85(39779.5-71407.39) | 138.32(98.23-169.76) | 90.21(62.01-111.17) | -1.64(-1.87--1.4) |
| UK | Male | 77611.81(56587.88-95086.91) | 76287.13(53142.05-94072.57) | 207.75(151.31-254.37) | 137.95(96.45-169.7) | -1.7(-1.86--1.53) |
| Ukraine | Both | 125801.68(90021.44-155227.44) | 104257.79(69747-136942.47) | 177.92(127.73-218.85) | 143.65(96.24-189.19) | -1.79(-2.24--1.35) |
| Ukraine | Female | 64473.08(44585.44-79848.93) | 45671.25(27929.95-63127.66) | 148.29(103.01-183.4) | 105.23(64.57-146.2) | -2.11(-2.48--1.75) |
| Ukraine | Male | 61328.6(44399.26-75406.09) | 58586.54(39044.67-79275.53) | 225.65(163.19-277.11) | 202.17(135.62-273.04) | -1.5(-2--1.01) |
| United Arab Emirates | Both | 524.98(310.55-773.19) | 4513.45(2649.86-6582.63) | 103.13(59.45-151.5) | 94.05(53.45-143.53) | -0.34(-0.66--0.01) |
| United Arab Emirates | Female | 199.3(113.3-307.07) | 1303.28(725.62-1995.37) | 112.04(60.93-170) | 90.59(44.86-158.25) | -0.48(-0.98-0.02) |
| United Arab Emirates | Male | 325.68(172.19-512.28) | 3210.17(1841.51-4779.83) | 104.73(57.69-160.59) | 97.02(55.86-142.9) | -0.51(-0.81--0.2) |
| Uruguay | Both | 8811.64(6721.27-10482.04) | 9705.01(6949.28-11891.98) | 230.92(175.88-275.31) | 187.77(135.4-229.42) | -0.82(-0.88--0.75) |
| Uruguay | Female | 4283.78(3210.77-5139.7) | 4630.6(3263.43-5705.02) | 200.89(151.37-241.14) | 154.2(111.05-188.75) | -1.03(-1.11--0.96) |
| Uruguay | Male | 4527.86(3474.51-5349.41) | 5074.41(3666.2-6227.63) | 267.1(205.71-314.93) | 230.83(167.22-282.56) | -0.58(-0.66--0.51) |
| USA | Both | 442766.98(324818.22-543529.25) | 565375.9(407667.17-696827.79) | 142.73(105.34-174.82) | 109.02(78.46-133.96) | -1.04(-1.14--0.93) |
| USA | Female | 206709.71(149378.28-255362.12) | 245766.41(175861.19-306778.85) | 117.05(85.77-144.08) | 88.24(62.79-109.53) | -1.06(-1.14--0.98) |
| USA | Male | 236057.27(174837.86-288809.64) | 319609.5(233342.64-390605.95) | 175.73(129.86-215.13) | 132.28(96.67-161.4) | -1.11(-1.25--0.98) |
| Uzbekistan | Both | 8921.9(6576.77-10759.85) | 16728.77(11703.41-21436.93) | 71.07(52.38-85.88) | 76.07(54.05-96.42) | -0.1(-0.31-0.1) |
| Uzbekistan | Female | 4165.36(3063.79-5100.93) | 7690.94(5378.28-9985.12) | 61.18(45.08-74.86) | 67.34(48.03-86.29) | 0.04(-0.14-0.22) |
| Uzbekistan | Male | 4756.55(3525.36-5693.23) | 9037.83(6331.12-11658.62) | 83.02(61.38-99.77) | 86.03(60.87-109.27) | -0.26(-0.5--0.02) |
| Vanuatu | Both | 54.21(36.71-75.13) | 164.35(114.74-225.4) | 74.34(51.55-100.93) | 88.06(62-119.11) | 0.36(0.18-0.54) |
| Vanuatu | Female | 21.8(13.99-31.16) | 69.36(45.25-98.4) | 67.25(44.7-94.1) | 78.8(53.24-109.78) | 0.24(0-0.48) |
| Vanuatu | Male | 32.41(20.99-45.72) | 94.99(64.93-132.25) | 80.6(52.83-111.05) | 96.88(67.11-134.41) | 0.48(0.35-0.61) |
| Venezuela | Both | 7040.86(5428.61-8363.48) | 26092.63(17616.2-36467.64) | 67.58(52.14-80.56) | 88.21(59.66-122.42) | 0.41(0.21-0.6) |
| Venezuela | Female | 3588.78(2730.02-4365.76) | 12018.94(7996.51-16711.95) | 66.09(50.37-80.44) | 77.32(51.64-107.13) | 0.07(-0.15-0.29) |
| Venezuela | Male | 3452.09(2701.21-4085.55) | 14073.69(9377.91-19974.1) | 69(54.14-81.55) | 100.03(67.51-140.92) | 0.72(0.38-1.05) |
| Vietnam | Both | 38784.18(29235.71-49331.45) | 165892.32(119485.83-219269.04) | 93.79(71.16-119) | 168.07(122.13-218.61) | 2.35(2.21-2.5) |
| Vietnam | Female | 17114.08(12714.86-22145.66) | 58574.7(40929.94-77858.94) | 72.71(53.99-93.9) | 108.47(76.35-142.72) | 1.68(1.55-1.82) |
| Vietnam | Male | 21670.1(16352.8-28158.82) | 107317.61(76602.11-140907.28) | 122.79(92.75-158.52) | 245.01(178.63-318.39) | 2.74(2.58-2.89) |
| Virgin Islands | Both | 10172.63(7365.1-12562.44) | 14122.77(10081.43-18399.86) | 145.49(105.37-179.98) | 104.86(75.05-136.55) | -1.47(-1.63--1.31) |
| Virgin Islands | Female | 4740.21(3375.25-5931.23) | 5980.37(4113.82-8029.08) | 119.97(85.48-150.56) | 82.94(56.99-111.64) | -1.58(-1.71--1.44) |
| Virgin Islands | Male | 5432.42(4010.94-6670.4) | 8142.4(5449.7-10909.97) | 178.96(131.62-220.7) | 129.67(87.14-173.66) | -1.48(-1.68--1.29) |
| Yemen | Both | 3095.04(1915.18-4672.42) | 10339.91(7233.96-14476.97) | 57.11(35.96-85.39) | 68.96(49.19-96.57) | 0.91(0.79-1.03) |
| Yemen | Female | 1571.79(948.64-2428.15) | 5235.22(3635.52-7366.98) | 56.82(35.1-86.59) | 67.88(48-94.19) | 0.86(0.74-0.98) |
| Yemen | Male | 1523.25(851.02-2588.95) | 5104.69(3358.67-7608.81) | 57.79(32.93-95.6) | 70.09(47.09-103.67) | 0.94(0.83-1.05) |
| Zambia | Both | 3016.25(2119.5-4103.27) | 9074.39(6322.85-12450.64) | 95.73(68.42-128.94) | 117.39(82.77-159.88) | 0.6(0.55-0.65) |
| Zambia | Female | 1437.53(850.63-2171.19) | 3995.67(2642.45-5733.09) | 90.09(54.83-134.14) | 99.28(66.58-143.09) | 0.31(0.17-0.44) |
| Zambia | Male | 1578.72(1134.64-2101.37) | 5078.72(3451.85-6958.48) | 100.14(72.18-133.28) | 137.44(95.2-184.55) | 0.95(0.8-1.11) |
| Zimbabwe | Both | 3666.78(2912.63-4471.28) | 8874.6(6212.02-11898.07) | 86.03(68.13-104.58) | 116.25(83.1-153.79) | 1.16(0.89-1.44) |
| Zimbabwe | Female | 1713.18(1320.38-2164.19) | 4860.56(3255.03-6763.69) | 78.49(61.04-99.68) | 112.62(76.54-155.3) | 2.22(1.73-2.72) |
| Zimbabwe | Male | 1953.6(1499.17-2405.08) | 4014.03(2734.55-5405.44) | 92.55(71.16-113.21) | 120.7(85.39-158.25) | 0.38(-0.11-0.87) |

DALYs, disability-adjusted life years; EAPC, Estimated annual percentage change; UI, uncertainty interval.
