# Supplementary material for: Dietary Risk-Related Colorectal Cancer Burden: Estimates From 1990 to 2019
Source: Front Nutr. 2021 Aug 24;8:690663. doi: 10.3389/fnut.2021.690663 (PMC8421520; doi:10.3389/fnut.2021.690663)
Supplement: Supplementary file 3 [file Data_Sheet_3.zip › Supplemental tables/Table S23.docx]

**Table S23** Countries or territories among 21 regions.

| **Region** | **Countries or territories** |
| --- | --- |
| **Central Asia** | Armenia, Azerbaijan, Georgia, Kazakhstan, Kyrgyzstan, Mongolia, Tajikistan, Turkmenistan, Uzbekistan |
| **Central Europe** | Albania, Bosnia and Herzegovina, Bulgaria, Croatia, Czech Republic, Hungary, Montenegro, North Macedonia, Poland, Romania, Serbia, Slovakia, Slovenia |
| **Eastern Europe** | Belarus, Estonia, Latvia, Lithuania, Moldova, Russia, Ukraine |
| **Australasia** | Australia**,** New Zealand |
| **High-income Asia Pacific** | Brunei, Japan, South Korea, Singapore |
| **High-income North America** | Canada, Greenland, USA |
| **Southern Latin America** | Argentina, Chile, Uruguay |
| **Western Europe** | Andorra, Austria, Belgium, Cyprus, Denmark, Finland, France, Germany, Greece, Iceland, Ireland, Israel, Italy, Luxembourg, Malta, Monaco, Netherlands, Norway, Portugal, San Marino, Spain, Sweden, Switzerland, UK |
| **Andean Latin America** | Bolivia, Ecuador, Peru |
| **Caribbean** | Antigua and Barbuda, The Bahamas, Barbados, Belize, Bermuda, Cuba, Dominica, Dominican Republic, Grenada, Guyana, Haiti, Jamaica, Puerto Rico, Saint Kitts and Nevis, Saint Lucia, Saint Vincent and the Grenadines, Suriname, Trinidad and Tobago, Virgin Islands |
| **Central Latin America** | Colombia, Costa Rica, El Salvador, Guatemala, Honduras, Mexico, Nicaragua, Panama, Venezuela |
| **Tropical Latin America** | Brazil, Paraguay |
| **North Africa and Middle East** | Afghanistan, Algeria, Bahrain, Egypt, Iran, Iraq, Jordan, Kuwait, Lebanon, Libya, Morocco, Oman, Palestine, Qatar, Saudi Arabia, Sudan, Syria, Tunisia, Turkey, United Arab Emirates, Yemen |
| **South Asia** | Bangladesh, Bhutan, India, Nepal, Pakistan |
| **East Asia** | China, North Korea, Taiwan (province of China) |
| **Oceania** | American Samoa, Cook Islands, Fiji, Guam, Kiribati, Marshall Islands, Federated States of Micronesia, Nauru, Niue, Northern Mariana Islands, Palau, Papua New Guinea, Samoa, Solomon Islands, Tokelau, Tonga, Tuvalu, Vanuatu |
| **Southeast Asia** | Cambodia, Indonesia, Laos, Malaysia, Maldives, Mauritius, Myanmar, Philippines, Seychelles, Sri Lanka, Thailand, Timor-Leste, Vietnam |
| **Central sub-Saharan Africa** | Angola, Central African Republic, Congo (Brazzaville), DR Congo, Equatorial Guinea, Gabon |
| **Eastern sub-Saharan Africa** | Burundi, Comoros, Djibouti, Eritrea, Ethiopia, Kenya, Madagascar, Malawi, Mozambique, Rwanda, Somalia, South Sudan, Uganda, Tanzania, Zambia |
| **Southern sub-Saharan Africa** | Botswana, eSwatini, Lesotho, Namibia, South Africa, Zimbabwe |
| **Western sub-Saharan Africa** | Benin, Burkina Faso, Cape Verde, Cameroon, Chad, Côte d'Ivoire, The Gambia, Ghana, Guinea, Guinea-Bissau, Liberia, Mali, Mauritania, Niger, Nigeria, São Tomé and PrÍncipe, Senegal, Sierra Leone, Togo |
|  |  |
