# Supplementary material for: Dietary Risk-Related Colorectal Cancer Burden: Estimates From 1990 to 2019
Source: Front Nutr. 2021 Aug 24;8:690663. doi: 10.3389/fnut.2021.690663 (PMC8421520; doi:10.3389/fnut.2021.690663)
Supplement: Supplementary file 3 [file Data_Sheet_3.zip › Supplemental tables/Table S3.docx]

**Table S3** Age-standardized summary exposure value of diet low in milk attributable to colorectal cancer and annualized rate of changes.

| **Location** | **Sex** | **Age-standardized summary exposure value (SEV) rate (per 100,000) (95% UI)** | | **Annualized rate of change (ARC, %) (95% UI)** | | |
| --- | --- | --- | --- | --- | --- | --- |
|  |  | **1990** | **2019** | **1990-2010** | **2010-2019** | **1990-2019** |
| Global | Both | 80.09(68.47-89.1) | 82.54(71.88-91.12) | 0.01(0-0.02) | 0.02(0.01-0.03) | 0.03(0.01-0.05) |
| Global | Female | 79.35(67.51-88.57) | 82.25(71.41-90.88) | 0.01(0-0.03) | 0.02(0.02-0.03) | 0.04(0.02-0.06) |
| Global | Male | 80.93(69.49-89.69) | 82.85(72.34-91.35) | 0(-0.01-0.02) | 0.02(0.01-0.03) | 0.02(0.01-0.04) |
| **Sociodemographic Index** | | | | | | |
| High SDI | Both | 62.43(42.4-78.72) | 58.79(41.06-73.71) | -0.06(-0.11--0.02) | 0.01(-0.01-0.02) | -0.06(-0.1--0.01) |
| High SDI | Female | 62.22(41.64-78.81) | 58.39(40.8-73.6) | -0.07(-0.12--0.01) | 0.01(-0.01-0.03) | -0.06(-0.11-0) |
| High SDI | Male | 62.63(43.36-78.81) | 59.17(41.47-74.7) | -0.06(-0.1--0.02) | 0.01(-0.02-0.03) | -0.06(-0.1--0.01) |
| High-middle SDI | Both | 69.21(55.63-80.38) | 76.07(63.27-86.27) | 0.04(0.01-0.09) | 0.06(0.04-0.07) | 0.1(0.06-0.16) |
| High-middle SDI | Female | 68.89(54.98-80.19) | 75.56(62.2-85.93) | 0.04(0.01-0.08) | 0.06(0.04-0.07) | 0.1(0.06-0.15) |
| High-middle SDI | Male | 69.7(56.38-80.89) | 76.61(64.36-86.64) | 0.04(0.01-0.09) | 0.06(0.04-0.07) | 0.1(0.06-0.16) |
| Low SDI | Both | 93.1(84.06-99.64) | 91.45(81.1-99.09) | -0.02(-0.04--0.01) | 0(0-0.01) | -0.02(-0.04-0) |
| Low SDI | Female | 92.69(83.29-99.57) | 90.88(80.16-98.95) | -0.02(-0.05--0.01) | 0(0-0.01) | -0.02(-0.04--0.01) |
| Low SDI | Male | 93.5(84.87-99.69) | 92.03(82.15-99.27) | -0.02(-0.03--0.01) | 0(0-0.01) | -0.02(-0.03-0) |
| Low-middle SDI | Both | 93.15(85-99.09) | 91.25(81.81-98.26) | -0.02(-0.04--0.01) | 0(-0.01-0) | -0.02(-0.04--0.01) |
| Low-middle SDI | Female | 92.99(84.8-98.97) | 90.97(81.45-98.08) | -0.02(-0.04--0.01) | 0(-0.01-0.01) | -0.02(-0.04--0.01) |
| Low-middle SDI | Male | 93.3(85.19-99.19) | 91.52(82.21-98.47) | -0.02(-0.03--0.01) | 0(-0.01-0) | -0.02(-0.04--0.01) |
| Middle SDI | Both | 92.68(85.02-98.59) | 91.2(82.39-97.9) | -0.02(-0.03--0.01) | 0(0-0) | -0.02(-0.03--0.01) |
| Middle SDI | Female | 92.59(84.91-98.54) | 91.02(82.13-97.8) | -0.02(-0.03--0.01) | 0(0-0.01) | -0.02(-0.03--0.01) |
| Middle SDI | Male | 92.75(85.01-98.62) | 91.37(82.56-98.05) | -0.01(-0.03--0.01) | 0(-0.01-0) | -0.01(-0.03--0.01) |
| **Region** | | | | | | |
| Andean Latin America | Both | 87.86(75.08-97.82) | 88.38(75.11-98.58) | 0(-0.01-0.02) | 0(0-0.01) | 0.01(-0.01-0.02) |
| Andean Latin America | Female | 87(73.4-97.54) | 87.69(73.58-98.4) | 0.01(-0.01-0.02) | 0(0-0.01) | 0.01(0-0.02) |
| Andean Latin America | Male | 88.75(76.81-98.13) | 89.12(76.22-98.77) | 0(-0.01-0.02) | 0(0-0.01) | 0(-0.01-0.02) |
| Australasia | Both | 40.02(18.03-59.09) | 21.03(7.33-39.03) | -0.5(-0.73--0.21) | 0.05(-0.06-0.27) | -0.47(-0.7--0.22) |
| Australasia | Female | 39.48(17.47-58.81) | 20.49(7.34-38.79) | -0.51(-0.74--0.21) | 0.05(-0.09-0.32) | -0.48(-0.71--0.22) |
| Australasia | Male | 40.59(17.81-59.77) | 21.61(7.35-40.03) | -0.49(-0.73--0.2) | 0.04(-0.1-0.29) | -0.47(-0.72--0.21) |
| Caribbean | Both | 87.55(71.6-99.7) | 89.41(75.28-99.82) | 0.02(0-0.05) | 0(0-0.01) | 0.02(0-0.06) |
| Caribbean | Female | 87.51(71.54-99.68) | 89.44(75.31-99.82) | 0.02(0-0.06) | 0(0-0.01) | 0.02(0-0.06) |
| Caribbean | Male | 87.59(71.91-99.71) | 89.38(75.08-99.83) | 0.02(0-0.05) | 0(0-0.01) | 0.02(0-0.06) |
| Central Asia | Both | 47.86(28.47-65.83) | 47.97(29.83-63.81) | -0.09(-0.13--0.03) | 0.1(0.04-0.19) | 0(-0.08-0.15) |
| Central Asia | Female | 47.31(27.85-65.16) | 47.38(29.45-63.36) | -0.09(-0.14--0.03) | 0.1(0.05-0.2) | 0(-0.08-0.14) |
| Central Asia | Male | 48.59(28.98-66.34) | 48.68(29.84-64.67) | -0.08(-0.13--0.01) | 0.09(0.04-0.18) | 0(-0.08-0.15) |
| Central Europe | Both | 58.16(33.87-78.01) | 61.16(37.23-79.73) | 0.05(-0.05-0.27) | 0(-0.04-0.02) | 0.05(-0.03-0.22) |
| Central Europe | Female | 59.83(34.35-80.62) | 64.42(39.38-84.54) | 0.1(0.01-0.32) | -0.03(-0.06--0.01) | 0.08(0-0.24) |
| Central Europe | Male | 56.33(33.13-75.35) | 57.73(35.45-75.34) | 0(-0.1-0.21) | 0.03(-0.01-0.06) | 0.02(-0.06-0.2) |
| Central Latin America | Both | 81.27(61.06-97.54) | 75.78(53.7-93.58) | -0.07(-0.15--0.02) | 0(-0.01-0.01) | -0.07(-0.15--0.02) |
| Central Latin America | Female | 81.14(60.58-97.48) | 75.58(53.32-93.51) | -0.07(-0.15--0.02) | 0(-0.01-0.01) | -0.07(-0.15--0.02) |
| Central Latin America | Male | 81.41(61.22-97.65) | 76(53.38-93.91) | -0.07(-0.15--0.01) | 0(-0.01-0.01) | -0.07(-0.15--0.02) |
| Central Sub-Saharan Africa | Both | 98.98(97.18-100) | 99.02(97.25-100) | 0(0-0) | 0(0-0) | 0(0-0) |
| Central Sub-Saharan Africa | Female | 99.01(97.25-100) | 98.98(97.16-100) | 0(0-0) | 0(0-0) | 0(0-0) |
| Central Sub-Saharan Africa | Male | 98.96(97.1-100) | 99.06(97.34-100) | 0(0-0) | 0(0-0) | 0(0-0) |
| East Asia | Both | 94.66(88.41-99.18) | 95.79(88.49-100) | 0(-0.01-0.02) | 0.01(0-0.02) | 0.01(-0.01-0.04) |
| East Asia | Female | 94.74(88.5-99.3) | 95.61(88.01-100) | 0(-0.02-0.02) | 0.01(0-0.02) | 0.01(-0.02-0.04) |
| East Asia | Male | 94.6(88.43-99.08) | 95.96(88.85-100) | 0.01(-0.01-0.03) | 0.01(0-0.02) | 0.01(-0.01-0.04) |
| Eastern Europe | Both | 34.42(14.16-52.66) | 43.3(21.83-61.65) | -0.1(-0.18--0.03) | 0.4(0.18-0.78) | 0.26(0.08-0.64) |
| Eastern Europe | Female | 33.52(13.32-51.37) | 42.2(21.08-61.32) | -0.13(-0.24--0.04) | 0.45(0.2-0.92) | 0.26(0.07-0.71) |
| Eastern Europe | Male | 35.52(15.11-54.15) | 44.62(22.41-63.31) | -0.07(-0.16-0.03) | 0.35(0.15-0.68) | 0.26(0.07-0.67) |
| Eastern Sub-Saharan Africa | Both | 91.66(81.1-99.27) | 89.5(76.02-99.5) | -0.02(-0.06-0) | 0(-0.01-0.01) | -0.02(-0.07-0) |
| Eastern Sub-Saharan Africa | Female | 91.66(81.06-99.26) | 89.44(75.93-99.51) | -0.02(-0.06-0) | 0(-0.01-0.01) | -0.02(-0.07-0) |
| Eastern Sub-Saharan Africa | Male | 91.66(81.05-99.26) | 89.56(76.1-99.52) | -0.02(-0.06-0) | 0(-0.01-0.01) | -0.02(-0.06-0) |
| High-income Asia Pacific | Both | 84.96(65.85-99.66) | 84.49(64.61-99.7) | -0.01(-0.03-0) | 0(0-0.01) | -0.01(-0.02-0) |
| High-income Asia Pacific | Female | 83.72(63.32-99.58) | 83.92(63.1-99.63) | 0(-0.01-0.01) | 0(-0.01-0.01) | 0(-0.01-0.02) |
| High-income Asia Pacific | Male | 86.22(68.64-99.73) | 85.03(65.97-99.77) | -0.02(-0.06-0) | 0(0-0.02) | -0.01(-0.05-0) |
| High-income North America | Both | 49.58(27.03-69.48) | 42.55(20.19-61.69) | -0.14(-0.3--0.05) | 0(-0.05-0.06) | -0.14(-0.29--0.05) |
| High-income North America | Female | 49.56(26.66-69.3) | 42.73(20.3-61.77) | -0.14(-0.3--0.05) | 0(-0.07-0.08) | -0.14(-0.29--0.04) |
| High-income North America | Male | 49.61(27.73-69.51) | 42.35(21.01-61.97) | -0.14(-0.3--0.05) | -0.01(-0.08-0.07) | -0.15(-0.32--0.05) |
| North Africa and Middle East | Both | 82.74(70.06-93.01) | 81.32(64.46-94.76) | -0.03(-0.1-0.01) | 0.01(0-0.02) | -0.02(-0.09-0.02) |
| North Africa and Middle East | Female | 82.37(69.47-92.69) | 80.92(64.21-94.45) | -0.03(-0.1-0.01) | 0.01(0-0.02) | -0.02(-0.09-0.03) |
| North Africa and Middle East | Male | 83.1(70.38-93.24) | 81.69(64.95-95.13) | -0.03(-0.1-0.01) | 0.01(0-0.02) | -0.02(-0.09-0.03) |
| Oceania | Both | 96.23(89.55-100) | 96.82(90.9-100) | 0.01(0-0.02) | 0(0-0) | 0.01(0-0.02) |
| Oceania | Female | 96.18(89.43-100) | 96.77(90.77-100) | 0.01(0-0.02) | 0(0-0) | 0.01(0-0.02) |
| Oceania | Male | 96.28(89.72-100) | 96.86(91.11-100) | 0.01(0-0.02) | 0(0-0) | 0.01(0-0.02) |
| South Asia | Both | 94.14(86.06-99.76) | 93.05(84.18-99.45) | -0.01(-0.02-0) | 0(-0.01-0.01) | -0.01(-0.02-0) |
| South Asia | Female | 94.18(86.16-99.76) | 93.09(84.23-99.45) | -0.01(-0.02-0) | 0(-0.01-0.01) | -0.01(-0.02-0) |
| South Asia | Male | 94.09(85.95-99.75) | 92.98(83.97-99.47) | -0.01(-0.02-0) | 0(-0.01-0.01) | -0.01(-0.03-0) |
| Southeast Asia | Both | 99.55(98.53-100) | 98.73(96.17-100) | -0.01(-0.02-0) | 0(0-0) | -0.01(-0.02-0) |
| Southeast Asia | Female | 99.54(98.51-100) | 98.69(96.02-100) | -0.01(-0.02-0) | 0(0-0) | -0.01(-0.03-0) |
| Southeast Asia | Male | 99.55(98.54-100) | 98.78(96.31-100) | -0.01(-0.02-0) | 0(0-0) | -0.01(-0.02-0) |
| Southern Latin America | Both | 76.37(54.6-93.74) | 76.73(55.11-94.25) | 0.02(0.01-0.04) | -0.02(-0.04--0.01) | 0(0-0.02) |
| Southern Latin America | Female | 76.21(54.4-93.69) | 76.49(54.87-94.05) | 0.02(0.01-0.04) | -0.02(-0.04--0.01) | 0(-0.01-0.02) |
| Southern Latin America | Male | 76.56(54.84-93.92) | 77(55.57-94.31) | 0.02(0.01-0.05) | -0.02(-0.04--0.01) | 0.01(-0.01-0.02) |
| Southern Sub-Saharan Africa | Both | 88.68(72.87-99.98) | 85.49(66.99-99.75) | -0.03(-0.09-0) | 0(-0.01-0) | -0.04(-0.1-0) |
| Southern Sub-Saharan Africa | Female | 88.59(72.7-99.98) | 85.44(66.93-99.72) | -0.03(-0.09-0) | 0(-0.02-0) | -0.04(-0.1-0) |
| Southern Sub-Saharan Africa | Male | 88.79(73.06-99.98) | 85.55(67.05-99.78) | -0.03(-0.09-0) | 0(-0.02-0) | -0.04(-0.1-0) |
| Tropical Latin America | Both | 74.18(48.09-94.79) | 63.44(36.93-83.79) | -0.14(-0.29--0.03) | -0.01(-0.03-0.01) | -0.14(-0.3--0.04) |
| Tropical Latin America | Female | 73.99(47.68-94.75) | 63.21(36.58-83.8) | -0.14(-0.29--0.03) | -0.01(-0.04-0.02) | -0.15(-0.3--0.04) |
| Tropical Latin America | Male | 74.38(48.49-94.92) | 63.69(37.28-84.43) | -0.14(-0.28--0.04) | -0.01(-0.04-0.02) | -0.14(-0.3--0.04) |
| Western Europe | Both | 57.01(33.53-75.89) | 50.52(28.81-69.66) | -0.13(-0.29--0.04) | 0.02(0-0.05) | -0.11(-0.25--0.04) |
| Western Europe | Female | 56.99(33.59-75.7) | 50.58(28.97-69.91) | -0.13(-0.28--0.04) | 0.02(0-0.05) | -0.11(-0.25--0.03) |
| Western Europe | Male | 57.04(33.47-75.95) | 50.48(28.68-69.51) | -0.13(-0.29--0.04) | 0.02(0-0.05) | -0.12(-0.26--0.03) |
| Western Sub-Saharan Africa | Both | 97.59(94.42-99.75) | 97.17(93.57-99.74) | 0(-0.01-0) | 0(0-0) | 0(-0.01-0) |
| Western Sub-Saharan Africa | Female | 97.4(93.99-99.74) | 97.2(93.6-99.74) | 0(-0.01-0) | 0(0-0) | 0(-0.01-0) |
| Western Sub-Saharan Africa | Male | 97.75(94.78-99.77) | 97.14(93.51-99.75) | -0.01(-0.01-0) | 0(0-0) | -0.01(-0.01-0) |

SDI, socio-demographic index; UI, uncertainty interval.
