# Supplementary material for: Dietary Risk-Related Colorectal Cancer Burden: Estimates From 1990 to 2019
Source: Front Nutr. 2021 Aug 24;8:690663. doi: 10.3389/fnut.2021.690663 (PMC8421520; doi:10.3389/fnut.2021.690663)
Supplement: Supplementary file 3 [file Data_Sheet_3.zip › Supplemental tables/Table S4.docx]

**Table S4** Age-standardized summary exposure value of diet low in calcium attributable to colorectal cancer and annualized rate of changes.

| **Location** | **Sex** | **Age-standardized summary exposure value (SEV) rate (per 100,000) (95% UI)** | | **Annualized rate of change (ARC, %) (95% UI)** | | |
| --- | --- | --- | --- | --- | --- | --- |
|  |  | **1990** | **2019** | **1990-2010** | **2010-2019** | **1990-2019** |
| Global | Both | 52.64(64.79-43.62) | 46.02(60.32-35.93) | -0.08(-0.04--0.11) | -0.05(-0.03--0.08) | -0.13(-0.06--0.18) |
| Global | Female | 49.62(61.85-40.62) | 43.68(58.14-33.42) | -0.07(-0.03--0.11) | -0.05(-0.03--0.08) | -0.12(-0.06--0.18) |
| Global | Male | 55.87(67.87-46.65) | 48.47(62.73-38.22) | -0.08(-0.04--0.12) | -0.06(-0.03--0.08) | -0.13(-0.07--0.19) |
| **Sociodemographic Index** | | | | | | |
| High SDI | Both | 20.79(34.41-12.41) | 18.65(31.38-10.86) | -0.05(-0.02--0.07) | -0.06(-0.03--0.09) | -0.1(-0.08--0.13) |
| High SDI | Female | 17.98(30.44-10.22) | 16.5(28-9.41) | -0.02(0.04--0.06) | -0.07(-0.03--0.1) | -0.08(-0.05--0.12) |
| High SDI | Male | 23.78(38.68-14.56) | 20.77(34.88-12.28) | -0.07(-0.05--0.1) | -0.06(-0.03--0.09) | -0.13(-0.1--0.17) |
| High-middle SDI | Both | 41.13(56.64-30.53) | 32.58(50.07-21.33) | -0.13(-0.07--0.19) | -0.09(-0.05--0.14) | -0.21(-0.12--0.3) |
| High-middle SDI | Female | 37.55(53-27.09) | 29.97(46.69-19.19) | -0.12(-0.06--0.19) | -0.09(-0.05--0.14) | -0.2(-0.11--0.3) |
| High-middle SDI | Male | 45.07(60.7-34.06) | 35.32(53.45-23.7) | -0.13(-0.07--0.2) | -0.1(-0.05--0.15) | -0.22(-0.12--0.31) |
| Low SDI | Both | 79.41(90.15-71.03) | 72.94(85.8-63.31) | -0.05(-0.03--0.06) | -0.04(-0.02--0.05) | -0.08(-0.04--0.11) |
| Low SDI | Female | 77.42(89.16-68.37) | 70.41(83.97-60.21) | -0.05(-0.03--0.07) | -0.04(-0.02--0.06) | -0.09(-0.05--0.12) |
| Low SDI | Male | 81.39(91.21-73.62) | 75.54(87.36-66.5) | -0.04(-0.02--0.06) | -0.03(-0.02--0.05) | -0.07(-0.04--0.1) |
| Low-middle SDI | Both | 67.4(80.21-58.06) | 56.66(71.32-45.92) | -0.1(-0.06--0.13) | -0.07(-0.04--0.09) | -0.16(-0.1--0.21) |
| Low-middle SDI | Female | 65.79(79.12-56.03) | 54.52(69.74-43.59) | -0.11(-0.06--0.15) | -0.07(-0.04--0.1) | -0.17(-0.1--0.23) |
| Low-middle SDI | Male | 68.98(81.29-59.98) | 58.9(72.97-48.33) | -0.09(-0.05--0.12) | -0.07(-0.04--0.09) | -0.15(-0.09--0.2) |
| Middle SDI | Both | 70.5(83.52-61.09) | 54.59(70.29-43.25) | -0.15(-0.09--0.21) | -0.08(-0.05--0.12) | -0.23(-0.13--0.3) |
| Middle SDI | Female | 67.97(81.45-58.16) | 51.98(68.07-40.29) | -0.16(-0.1--0.22) | -0.09(-0.05--0.12) | -0.24(-0.14--0.31) |
| Middle SDI | Male | 73.01(85.42-63.75) | 57.3(72.68-46.21) | -0.15(-0.08--0.2) | -0.08(-0.04--0.11) | -0.22(-0.12--0.29) |
| **Region** | | | | | | |
| Andean Latin America | Both | 64.75(81.27-53.13) | 50.87(69.1-38) | -0.16(-0.08--0.22) | -0.06(-0.03--0.09) | -0.21(-0.11--0.29) |
| Andean Latin America | Female | 61.18(78.36-49.18) | 46.76(65.68-33.66) | -0.18(-0.09--0.25) | -0.07(-0.03--0.11) | -0.24(-0.12--0.33) |
| Andean Latin America | Male | 68.48(84.23-57.21) | 55.19(73.29-42.61) | -0.15(-0.07--0.2) | -0.06(-0.03--0.09) | -0.19(-0.1--0.27) |
| Australasia | Both | 17.09(32.08-7.9) | 17.29(31.52-8.58) | 0.15(0.34-0.06) | -0.12(-0.08--0.18) | 0.01(0.13--0.06) |
| Australasia | Female | 14.33(27.42-6.01) | 14.53(26.82-6.86) | 0.17(0.5-0.06) | -0.14(-0.09--0.21) | 0.01(0.22--0.09) |
| Australasia | Male | 20(37.18-9.85) | 20.21(36.36-10.51) | 0.14(0.29-0.06) | -0.11(-0.07--0.18) | 0.01(0.1--0.05) |
| Caribbean | Both | 51.62(68.25-40.1) | 48.87(65.24-37.77) | -0.04(-0.02--0.06) | -0.01(0--0.02) | -0.05(-0.03--0.08) |
| Caribbean | Female | 47.94(65.32-36.24) | 45.63(62.5-34.43) | -0.04(-0.01--0.07) | -0.01(0--0.02) | -0.05(-0.02--0.08) |
| Caribbean | Male | 55.52(71.85-44.21) | 52.34(68.23-41.11) | -0.04(-0.03--0.06) | -0.01(-0.01--0.02) | -0.06(-0.03--0.08) |
| Central Asia | Both | 31.64(50.83-19.93) | 24.04(39.88-14.44) | -0.11(-0.09--0.13) | -0.15(-0.08--0.22) | -0.24(-0.18--0.31) |
| Central Asia | Female | 28.21(47.12-16.88) | 20.75(35.76-11.72) | -0.13(-0.11--0.16) | -0.16(-0.08--0.24) | -0.26(-0.2--0.35) |
| Central Asia | Male | 35.71(55.17-23.52) | 27.75(44.66-17.43) | -0.09(-0.07--0.11) | -0.14(-0.08--0.21) | -0.22(-0.16--0.29) |
| Central Europe | Both | 20.49(37.65-10.33) | 16(29.54-7.78) | -0.09(-0.06--0.1) | -0.15(-0.09--0.23) | -0.22(-0.18--0.28) |
| Central Europe | Female | 17.51(33.21-8.06) | 13.23(25.07-5.91) | -0.1(-0.06--0.12) | -0.16(-0.09--0.26) | -0.24(-0.2--0.32) |
| Central Europe | Male | 23.76(42.43-13.07) | 18.91(34.45-9.87) | -0.08(-0.06--0.09) | -0.14(-0.08--0.21) | -0.2(-0.16--0.27) |
| Central Latin America | Both | 53.8(72.24-41.07) | 42.96(62.56-30.04) | -0.18(-0.09--0.26) | -0.02(-0.01--0.04) | -0.2(-0.1--0.28) |
| Central Latin America | Female | 49.69(68.77-36.75) | 38.9(59.61-25.91) | -0.2(-0.1--0.29) | -0.02(-0.01--0.04) | -0.22(-0.11--0.31) |
| Central Latin America | Male | 58.16(76.28-45.69) | 47.46(66.61-34.25) | -0.17(-0.08--0.24) | -0.02(-0.01--0.04) | -0.18(-0.09--0.26) |
| Central Sub-Saharan Africa | Both | 86.19(94.58-78.65) | 88.12(94.57-82.33) | 0.04(0.08-0) | -0.02(0--0.03) | 0.02(0.05-0) |
| Central Sub-Saharan Africa | Female | 84.2(93.75-75.89) | 86.24(93.87-79.68) | 0.04(0.09-0.01) | -0.02(0--0.03) | 0.02(0.05-0) |
| Central Sub-Saharan Africa | Male | 88.38(95.49-81.74) | 90.13(95.33-85.21) | 0.03(0.07-0) | -0.01(0--0.03) | 0.02(0.05-0) |
| East Asia | Both | 67.8(83.33-56.44) | 46.7(66.07-33.42) | -0.2(-0.1--0.28) | -0.14(-0.07--0.21) | -0.31(-0.16--0.43) |
| East Asia | Female | 64.91(81.37-53.01) | 45.01(64.81-31.59) | -0.19(-0.09--0.28) | -0.14(-0.07--0.21) | -0.31(-0.16--0.42) |
| East Asia | Male | 70.62(85.28-59.2) | 48.46(67.71-34.92) | -0.2(-0.1--0.28) | -0.15(-0.07--0.22) | -0.31(-0.16--0.43) |
| Eastern Europe | Both | 24.5(43.81-13.31) | 18.45(34.23-8.89) | -0.19(-0.1--0.31) | -0.07(-0.04--0.12) | -0.25(-0.14--0.39) |
| Eastern Europe | Female | 21.52(40.12-10.83) | 15.71(29.92-6.87) | -0.21(-0.1--0.36) | -0.08(-0.04--0.15) | -0.27(-0.14--0.45) |
| Eastern Europe | Male | 28.2(48.64-16.21) | 21.71(39.56-11.49) | -0.17(-0.09--0.27) | -0.07(-0.03--0.12) | -0.23(-0.12--0.35) |
| Eastern Sub-Saharan Africa | Both | 82.25(90.95-75.44) | 74.65(86.36-65.55) | -0.06(-0.03--0.09) | -0.03(-0.02--0.04) | -0.09(-0.05--0.13) |
| Eastern Sub-Saharan Africa | Female | 80.15(89.68-72.71) | 71.89(84.66-62.26) | -0.07(-0.04--0.1) | -0.03(-0.02--0.05) | -0.1(-0.06--0.15) |
| Eastern Sub-Saharan Africa | Male | 84.44(92.19-78.24) | 77.6(88.4-69.22) | -0.06(-0.03--0.08) | -0.03(-0.01--0.04) | -0.08(-0.04--0.12) |
| High-income Asia Pacific | Both | 40.28(60.98-26.79) | 37.52(58.51-24.05) | 0(0.02--0.02) | -0.07(-0.03--0.11) | -0.07(-0.03--0.11) |
| High-income Asia Pacific | Female | 37.14(58.49-23.55) | 36.75(58.09-23.43) | 0.05(0.1-0.02) | -0.06(-0.03--0.11) | -0.01(0.02--0.05) |
| High-income Asia Pacific | Male | 43.56(63.61-30.17) | 38.24(58.99-24.9) | -0.05(-0.02--0.1) | -0.07(-0.03--0.11) | -0.12(-0.06--0.19) |
| High-income North America | Both | 13.77(25.65-6.38) | 11.67(21.48-5.45) | -0.12(-0.07--0.18) | -0.04(0--0.09) | -0.15(-0.09--0.22) |
| High-income North America | Female | 11.55(22.08-5.05) | 10.27(19.06-4.54) | -0.09(0.01--0.18) | -0.03(0.03--0.09) | -0.11(-0.01--0.22) |
| High-income North America | Male | 16.18(29.53-7.79) | 13.14(23.93-6.55) | -0.15(-0.08--0.22) | -0.04(0.01--0.11) | -0.19(-0.11--0.27) |
| North Africa and Middle East | Both | 53.87(68.7-42.57) | 42.87(59.48-31.54) | -0.18(-0.09--0.25) | -0.03(-0.02--0.04) | -0.2(-0.12--0.27) |
| North Africa and Middle East | Female | 50.43(65.61-39.08) | 39.44(56.32-28.11) | -0.19(-0.1--0.27) | -0.03(-0.02--0.04) | -0.22(-0.13--0.29) |
| North Africa and Middle East | Male | 57.17(72.03-46.1) | 46.05(62.44-34.56) | -0.17(-0.09--0.23) | -0.03(-0.02--0.04) | -0.19(-0.11--0.26) |
| Oceania | Both | 77.7(89.66-68.17) | 79.86(90.58-70.98) | 0.04(0.07-0.01) | -0.01(0--0.02) | 0.03(0.05-0.01) |
| Oceania | Female | 74.85(87.98-64.63) | 77.11(89.1-67.51) | 0.05(0.07-0.02) | -0.01(0--0.03) | 0.03(0.05-0.01) |
| Oceania | Male | 80.39(91.19-71.41) | 82.52(92.22-74.28) | 0.04(0.06-0.01) | -0.01(0--0.02) | 0.03(0.05-0.01) |
| South Asia | Both | 62.77(77.02-52.35) | 52.42(67.63-41.38) | -0.07(-0.05--0.1) | -0.1(-0.06--0.13) | -0.16(-0.1--0.22) |
| South Asia | Female | 61.17(76.05-50.43) | 50.33(66.28-38.89) | -0.08(-0.05--0.11) | -0.1(-0.06--0.14) | -0.18(-0.11--0.23) |
| South Asia | Male | 64.24(77.95-54.15) | 54.5(69.15-43.55) | -0.06(-0.04--0.09) | -0.09(-0.06--0.12) | -0.15(-0.1--0.2) |
| Southeast Asia | Both | 94.09(96.36-91.75) | 83.38(92.74-75.28) | -0.08(-0.02--0.14) | -0.03(-0.01--0.05) | -0.11(-0.03--0.18) |
| Southeast Asia | Female | 93.3(96.24-90.46) | 81.15(91.6-72.31) | -0.1(-0.03--0.15) | -0.04(-0.02--0.06) | -0.13(-0.04--0.2) |
| Southeast Asia | Male | 94.94(96.49-93.14) | 85.72(93.99-78.5) | -0.07(-0.01--0.12) | -0.03(-0.01--0.05) | -0.1(-0.03--0.16) |
| Southern Latin America | Both | 36.65(57-23.99) | 30.68(50.79-18.49) | -0.09(-0.05--0.14) | -0.08(-0.04--0.12) | -0.16(-0.09--0.25) |
| Southern Latin America | Female | 32.77(53.59-20.21) | 27.04(47.03-15.13) | -0.1(-0.05--0.16) | -0.08(-0.04--0.14) | -0.18(-0.1--0.27) |
| Southern Latin America | Male | 40.92(60.92-27.9) | 34.67(54.95-22.14) | -0.09(-0.05--0.13) | -0.07(-0.04--0.12) | -0.15(-0.09--0.23) |
| Southern Sub-Saharan Africa | Both | 80(89.49-72.31) | 76.83(87.87-68.25) | -0.02(-0.01--0.04) | -0.02(-0.01--0.03) | -0.04(-0.02--0.06) |
| Southern Sub-Saharan Africa | Female | 77.73(88.28-69.31) | 74.5(86.6-65.29) | -0.03(-0.01--0.04) | -0.02(-0.01--0.03) | -0.04(-0.02--0.07) |
| Southern Sub-Saharan Africa | Male | 82.6(90.9-75.71) | 79.52(89.29-71.68) | -0.02(-0.01--0.04) | -0.01(-0.01--0.03) | -0.04(-0.02--0.06) |
| Tropical Latin America | Both | 48.4(67.3-35.44) | 29.14(49.13-17.07) | -0.39(-0.24--0.53) | -0.01(0.01--0.04) | -0.4(-0.25--0.53) |
| Tropical Latin America | Female | 44.15(63.87-31.07) | 25.42(45.13-13.81) | -0.42(-0.26--0.57) | -0.01(0.02--0.05) | -0.42(-0.27--0.58) |
| Tropical Latin America | Male | 52.96(71.4-40.07) | 33.24(53.26-20.65) | -0.37(-0.22--0.49) | -0.01(0.02--0.04) | -0.37(-0.22--0.5) |
| Western Europe | Both | 14.37(26.57-6.67) | 11.73(21.57-5.58) | -0.14(-0.1--0.18) | -0.05(-0.03--0.08) | -0.18(-0.13--0.24) |
| Western Europe | Female | 11.69(21.85-5.09) | 9.04(16.45-3.84) | -0.18(-0.12--0.24) | -0.06(-0.03--0.1) | -0.23(-0.16--0.31) |
| Western Europe | Male | 17.28(31.82-8.51) | 14.52(26.82-6.93) | -0.12(-0.08--0.17) | -0.05(-0.03--0.08) | -0.16(-0.11--0.23) |
| Western Sub-Saharan Africa | Both | 85.28(93.71-77.89) | 72.64(86.42-62.12) | -0.13(-0.06--0.18) | -0.02(-0.01--0.04) | -0.15(-0.07--0.21) |
| Western Sub-Saharan Africa | Female | 82.97(92.59-74.75) | 69.62(84.68-58.57) | -0.14(-0.07--0.19) | -0.02(-0.01--0.04) | -0.16(-0.08--0.22) |
| Western Sub-Saharan Africa | Male | 87.47(94.67-80.95) | 75.96(88.56-66.17) | -0.11(-0.05--0.16) | -0.02(-0.01--0.03) | -0.13(-0.06--0.19) |

SDI, socio-demographic index; UI, uncertainty interval.
