# Supplementary material for: Dietary Risk-Related Colorectal Cancer Burden: Estimates From 1990 to 2019
Source: Front Nutr. 2021 Aug 24;8:690663. doi: 10.3389/fnut.2021.690663 (PMC8421520; doi:10.3389/fnut.2021.690663)
Supplement: Supplementary file 3 [file Data_Sheet_3.zip › Supplemental tables/Table S5.docx]

**Table S5** Age-standardized summary exposure value of diet low in whole grains attributable to colorectal cancer and annualized rate of changes.

| **Location** | **Sex** | **Age-standardized summary exposure value (SEV) rate (per 100,000) (95% UI)** | | **Annualized rate of change (ARC, %) (95% UI)** | | |
| --- | --- | --- | --- | --- | --- | --- |
|  |  | **1990** | **2019** | **1990-2010** | **2010-2019** | **1990-2019** |
| Global | Both | 79.92(72.52-87.44) | 78.81(71.06-86.78) | 0(-0.01-0) | -0.01(-0.01--0.01) | -0.01(-0.02--0.01) |
| Global | Female | 79.15(71.57-87) | 77.68(69.61-86.02) | -0.01(-0.01-0) | -0.01(-0.02--0.01) | -0.02(-0.03--0.01) |
| Global | Male | 80.68(73.44-87.9) | 79.97(72.46-87.48) | 0(0-0) | -0.01(-0.01-0) | -0.01(-0.01-0) |
| **Sociodemographic Index** | | | | | | |
| High SDI | Both | 75.33(65.93-84.9) | 80.16(72.03-88.15) | 0.06(0.03-0.09) | 0(0-0.01) | 0.06(0.04-0.1) |
| High SDI | Female | 72.71(62.3-83.08) | 77.93(68.86-86.62) | 0.07(0.04-0.1) | 0(0-0.01) | 0.07(0.04-0.11) |
| High SDI | Male | 78.16(69.67-86.84) | 82.41(75.04-89.75) | 0.05(0.03-0.08) | 0(0-0.01) | 0.05(0.03-0.08) |
| High-middle SDI | Both | 90.12(86.05-94.32) | 86.1(80.43-91.92) | -0.03(-0.05--0.02) | -0.01(-0.02--0.01) | -0.04(-0.07--0.02) |
| High-middle SDI | Female | 91.21(87.5-94.82) | 86.63(81.01-92.05) | -0.04(-0.06--0.02) | -0.01(-0.02--0.01) | -0.05(-0.07--0.03) |
| High-middle SDI | Male | 88.92(84.26-93.74) | 85.53(79.41-91.86) | -0.03(-0.04--0.01) | -0.01(-0.02-0) | -0.04(-0.06--0.02) |
| Low SDI | Both | 81.69(74.52-88.79) | 81.34(73.71-88.68) | 0(0-0.01) | -0.01(-0.01-0) | 0(-0.01-0) |
| Low SDI | Female | 80.35(72.86-87.98) | 79.98(71.87-87.8) | 0(0-0.01) | -0.01(-0.01-0) | 0(-0.01-0) |
| Low SDI | Male | 83.03(75.98-89.73) | 82.74(75.52-89.66) | 0(0-0.01) | -0.01(-0.01-0) | 0(-0.01-0) |
| Low-middle SDI | Both | 74.31(65.72-83.41) | 73(64.39-82.37) | -0.01(-0.02-0) | -0.01(-0.01-0) | -0.02(-0.03--0.01) |
| Low-middle SDI | Female | 72.78(64.06-82.2) | 71.33(62.51-81.15) | -0.01(-0.02-0) | -0.01(-0.02-0) | -0.02(-0.03--0.01) |
| Low-middle SDI | Male | 75.8(67.4-84.4) | 74.75(66.26-83.48) | -0.01(-0.01-0) | -0.01(-0.01-0) | -0.01(-0.02--0.01) |
| Middle SDI | Both | 76.92(68.29-85.56) | 76.33(67.74-85.24) | 0(0-0.01) | -0.01(-0.02-0) | -0.01(-0.01-0) |
| Middle SDI | Female | 75.83(67.07-84.8) | 75.3(66.49-84.18) | 0(0-0.01) | -0.01(-0.02-0) | -0.01(-0.02-0) |
| Middle SDI | Male | 78.01(69.72-86.29) | 77.42(69.11-86.02) | 0(0-0.01) | -0.01(-0.02-0) | -0.01(-0.02-0) |
| **Region** | | | | | | |
| Andean Latin America | Both | 82.82(75.1-90.35) | 77.66(68.28-86.83) | -0.05(-0.08--0.03) | -0.01(-0.02--0.01) | -0.06(-0.1--0.03) |
| Andean Latin America | Female | 81.77(73.69-89.73) | 76.32(66.45-85.94) | -0.05(-0.08--0.03) | -0.02(-0.03--0.01) | -0.07(-0.1--0.04) |
| Andean Latin America | Male | 83.91(76.57-91.08) | 79.05(69.95-87.88) | -0.05(-0.07--0.02) | -0.01(-0.02-0) | -0.06(-0.09--0.03) |
| Australasia | Both | 74.62(64.02-84.9) | 79.31(70.47-87.89) | 0.07(0.04-0.11) | -0.01(-0.02-0) | 0.06(0.03-0.1) |
| Australasia | Female | 73.15(61.98-83.85) | 78.14(69.07-87.09) | 0.07(0.04-0.12) | -0.01(-0.02-0) | 0.07(0.04-0.11) |
| Australasia | Male | 76.17(66.17-85.91) | 80.54(72.1-88.71) | 0.06(0.03-0.1) | -0.01(-0.02-0) | 0.06(0.03-0.1) |
| Caribbean | Both | 86.04(79.71-92.19) | 81.93(74.11-89.56) | -0.05(-0.07--0.03) | 0(0-0) | -0.05(-0.07--0.03) |
| Caribbean | Female | 85(78.31-91.53) | 80.82(72.63-88.75) | -0.05(-0.07--0.03) | 0(-0.01-0.01) | -0.05(-0.08--0.03) |
| Caribbean | Male | 87.13(81.16-92.9) | 83.12(75.81-90.31) | -0.05(-0.07--0.03) | 0(-0.01-0.01) | -0.05(-0.07--0.03) |
| Central Asia | Both | 99.92(100-100) | 99.92(100-100) | 0(0-0) | 0(0-0) | 0(0-0) |
| Central Asia | Female | 99.92(100-100) | 99.92(100-100) | 0(0-0) | 0(0-0) | 0(0-0) |
| Central Asia | Male | 99.92(100-100) | 99.92(100-100) | 0(0-0) | 0(0-0) | 0(0-0) |
| Central Europe | Both | 89.09(84.23-93.97) | 84.58(78.05-91.4) | -0.05(-0.08--0.03) | 0(0-0.01) | -0.05(-0.08--0.03) |
| Central Europe | Female | 87.65(81.96-93.12) | 84.72(78.15-91.02) | -0.04(-0.06--0.02) | 0.01(0-0.02) | -0.03(-0.05--0.02) |
| Central Europe | Male | 90.69(86.38-95.06) | 84.46(77.37-91.81) | -0.06(-0.09--0.03) | -0.01(-0.02-0) | -0.07(-0.11--0.03) |
| Central Latin America | Both | 67.49(56.96-79.02) | 67.73(57.48-78.87) | 0(-0.01-0.01) | 0.01(0-0.02) | 0(-0.01-0.02) |
| Central Latin America | Female | 65.52(54.61-77.56) | 66.04(55.63-77.36) | 0(-0.01-0.02) | 0.01(-0.01-0.02) | 0.01(-0.01-0.03) |
| Central Latin America | Male | 69.6(59.5-80.73) | 69.61(59.59-80.61) | -0.01(-0.01-0) | 0.01(-0.01-0.02) | 0(-0.01-0.01) |
| Central Sub-Saharan Africa | Both | 73.45(62.71-83.76) | 76.23(66.55-85.86) | 0.05(0.03-0.08) | -0.01(-0.03--0.01) | 0.04(0.02-0.06) |
| Central Sub-Saharan Africa | Female | 71.85(60.88-82.88) | 74.91(64.76-85.05) | 0.06(0.03-0.09) | -0.02(-0.03-0) | 0.04(0.02-0.07) |
| Central Sub-Saharan Africa | Male | 75.2(65.06-85.05) | 77.68(68.22-86.7) | 0.05(0.02-0.08) | -0.01(-0.03-0) | 0.03(0.02-0.06) |
| East Asia | Both | 81.44(73.5-89.16) | 83.02(75.58-90.33) | 0.03(0.02-0.05) | -0.01(-0.02-0) | 0.02(0.01-0.04) |
| East Asia | Female | 80.52(72.19-88.36) | 82.16(74.15-89.81) | 0.03(0.02-0.06) | -0.01(-0.03-0) | 0.02(0.01-0.04) |
| East Asia | Male | 82.34(75-89.97) | 83.89(76.91-90.97) | 0.03(0.01-0.05) | -0.01(-0.02-0) | 0.02(0.01-0.04) |
| Eastern Europe | Both | 97.01(95.4-98.81) | 90.39(85.75-94.8) | -0.06(-0.09--0.03) | -0.01(-0.02-0) | -0.07(-0.1--0.04) |
| Eastern Europe | Female | 96.61(94.79-98.6) | 89.48(84.43-94.35) | -0.06(-0.1--0.03) | -0.01(-0.02-0) | -0.07(-0.11--0.04) |
| Eastern Europe | Male | 97.52(96.09-99.1) | 91.48(87.2-95.55) | -0.05(-0.08--0.03) | -0.01(-0.02-0) | -0.06(-0.1--0.03) |
| Eastern Sub-Saharan Africa | Both | 76.57(67.85-85.23) | 76.2(66.72-85.29) | 0.01(0-0.01) | -0.01(-0.02--0.01) | 0(-0.02-0.01) |
| Eastern Sub-Saharan Africa | Female | 75.21(66.28-84.37) | 74.67(64.8-84.3) | 0(-0.01-0.01) | -0.01(-0.02-0) | -0.01(-0.03-0.01) |
| Eastern Sub-Saharan Africa | Male | 77.99(69.44-86.34) | 77.84(68.92-86.44) | 0.01(0-0.02) | -0.01(-0.02-0) | 0(-0.01-0.01) |
| High-income Asia Pacific | Both | 64.72(52.42-77.75) | 73.14(62.63-83.47) | 0.14(0.08-0.21) | -0.01(-0.02-0) | 0.13(0.07-0.2) |
| High-income Asia Pacific | Female | 62.77(50.19-76.4) | 71.69(60.71-82.65) | 0.15(0.08-0.23) | 0(-0.02-0.01) | 0.14(0.08-0.22) |
| High-income Asia Pacific | Male | 66.81(54.76-79.39) | 74.58(64.21-84.66) | 0.12(0.07-0.2) | -0.01(-0.02-0.01) | 0.12(0.06-0.19) |
| High-income North America | Both | 78.14(68.93-87.04) | 80.14(71.63-88.52) | 0.03(0.01-0.05) | 0(-0.01-0.01) | 0.03(0.01-0.05) |
| High-income North America | Female | 76.94(67.08-86.24) | 79.24(70.29-87.83) | 0.03(0.01-0.06) | 0(-0.02-0.02) | 0.03(0.01-0.06) |
| High-income North America | Male | 79.46(70.86-88.06) | 81.09(72.97-89.07) | 0.02(0.01-0.04) | 0(-0.02-0.01) | 0.02(0-0.04) |
| North Africa and Middle East | Both | 96.73(95.31-98.21) | 96(94.29-97.76) | -0.01(-0.01--0.01) | 0(0-0) | -0.01(-0.01-0) |
| North Africa and Middle East | Female | 96.61(95.18-98.09) | 95.98(94.27-97.7) | -0.01(-0.01-0) | 0(0-0) | -0.01(-0.01-0) |
| North Africa and Middle East | Male | 96.83(95.47-98.32) | 96.02(94.28-97.83) | -0.01(-0.01--0.01) | 0(0-0) | -0.01(-0.01-0) |
| Oceania | Both | 91.42(87.13-95.57) | 90.47(85.74-95.01) | -0.01(-0.03-0) | 0(0-0.01) | -0.01(-0.03-0) |
| Oceania | Female | 91.15(86.75-95.42) | 90.16(85.33-94.76) | -0.01(-0.03-0) | 0(0-0.01) | -0.01(-0.03-0) |
| Oceania | Male | 91.68(87.48-95.73) | 90.77(86.03-95.18) | -0.01(-0.03-0) | 0(0-0.01) | -0.01(-0.03-0) |
| South Asia | Both | 72.55(63.72-81.95) | 71.79(62.73-81.44) | 0(-0.01-0) | -0.01(-0.01-0) | -0.01(-0.02-0) |
| South Asia | Female | 70.8(61.73-80.93) | 70(60.74-80.28) | 0(-0.01-0.01) | -0.01(-0.02-0) | -0.01(-0.03-0) |
| South Asia | Male | 74.15(65.57-82.82) | 73.58(64.68-82.59) | 0(-0.01-0.01) | -0.01(-0.01-0) | -0.01(-0.02-0) |
| Southeast Asia | Both | 62.15(49.79-76.45) | 60.2(47.3-75.16) | -0.02(-0.03-0) | -0.02(-0.03--0.01) | -0.03(-0.06--0.01) |
| Southeast Asia | Female | 59.17(46.05-74.48) | 57.14(43.43-73.06) | -0.02(-0.04-0) | -0.02(-0.04-0) | -0.03(-0.07--0.01) |
| Southeast Asia | Male | 65.38(53.04-78.76) | 63.42(50.63-77.5) | -0.02(-0.03--0.01) | -0.01(-0.03-0) | -0.03(-0.05--0.01) |
| Southern Latin America | Both | 99.02(98.46-99.72) | 89.86(84.5-94.98) | -0.08(-0.12--0.04) | -0.01(-0.02--0.01) | -0.09(-0.14--0.05) |
| Southern Latin America | Female | 98.55(97.67-99.55) | 88.86(82.96-94.64) | -0.08(-0.12--0.04) | -0.02(-0.04--0.01) | -0.1(-0.15--0.05) |
| Southern Latin America | Male | 99.56(99.28-99.91) | 90.94(86.12-95.69) | -0.08(-0.13--0.04) | 0(-0.01-0) | -0.09(-0.13--0.04) |
| Southern Sub-Saharan Africa | Both | 60.93(48.15-74.9) | 62.3(49.4-76.05) | 0.02(0.01-0.03) | 0(-0.01-0.02) | 0.02(0.01-0.04) |
| Southern Sub-Saharan Africa | Female | 58.82(45.88-73.17) | 60.35(47.29-74.61) | 0.02(0.01-0.04) | 0(-0.02-0.02) | 0.03(0.01-0.05) |
| Southern Sub-Saharan Africa | Male | 63.37(50.71-76.86) | 64.58(52.37-77.68) | 0.02(0.01-0.03) | 0(-0.02-0.02) | 0.02(0-0.04) |
| Tropical Latin America | Both | 77.96(69.42-87.13) | 72.56(61.79-83.9) | -0.06(-0.11--0.02) | -0.01(-0.02-0.01) | -0.07(-0.12--0.03) |
| Tropical Latin America | Female | 77.15(68.47-86.39) | 71.77(60.55-83.37) | -0.06(-0.12--0.01) | -0.01(-0.03-0.01) | -0.07(-0.14--0.02) |
| Tropical Latin America | Male | 78.83(69.74-88.15) | 73.42(62.57-84.72) | -0.06(-0.1--0.03) | -0.01(-0.02-0.01) | -0.07(-0.11--0.04) |
| Western Europe | Both | 89.23(84.61-93.93) | 90.34(86.04-94.73) | 0.01(0-0.02) | 0(0-0.01) | 0.01(0.01-0.02) |
| Western Europe | Female | 91.87(88.42-95.42) | 91.69(88.06-95.45) | 0(-0.01-0) | 0(-0.01-0) | 0(-0.01-0.01) |
| Western Europe | Male | 86.43(80.32-92.56) | 88.93(83.78-94.12) | 0.02(0.01-0.03) | 0.01(0-0.01) | 0.03(0.01-0.05) |
| Western Sub-Saharan Africa | Both | 83.92(76.89-90.85) | 80(71.41-88.21) | -0.04(-0.06--0.02) | -0.01(-0.02-0) | -0.05(-0.07--0.03) |
| Western Sub-Saharan Africa | Female | 82.91(75.51-90.14) | 78.79(69.68-87.45) | -0.04(-0.06--0.02) | -0.01(-0.02-0) | -0.05(-0.08--0.03) |
| Western Sub-Saharan Africa | Male | 84.88(78.22-91.52) | 81.33(73.23-89.17) | -0.03(-0.05--0.02) | -0.01(-0.02-0) | -0.04(-0.07--0.02) |

SDI, socio-demographic index; UI, uncertainty interval.
