# Supplementary material for: Dietary Risk-Related Colorectal Cancer Burden: Estimates From 1990 to 2019
Source: Front Nutr. 2021 Aug 24;8:690663. doi: 10.3389/fnut.2021.690663 (PMC8421520; doi:10.3389/fnut.2021.690663)
Supplement: Supplementary file 3 [file Data_Sheet_3.zip › Supplemental tables/Table S6.docx]

**Table S6** Age-standardized summary exposure value of diet low in fiber attributable to colorectal cancer and annualized rate of changes.

| **Location** | **Sex** | **Age-standardized summary exposure value (SEV) rate (per 100,000) (95% UI)** | | **Annualized rate of change (ARC, %) (95% UI)** | | |
| --- | --- | --- | --- | --- | --- | --- |
|  |  | **1990** | **2019** | **1990-2010** | **2010-2019** | **1990-2019** |
| Global | Both | 36.87(47.86-25.93) | 27.62(18.6-36.95) | -0.15(-0.11--0.19) | -0.12(-0.1--0.15) | -0.25(-0.21--0.3) |
| Global | Female | 37.09(48.37-25.95) | 28(18.82-37.56) | -0.14(-0.1--0.19) | -0.12(-0.09--0.16) | -0.25(-0.2--0.3) |
| Global | Male | 36.65(47.47-25.72) | 27.22(18.12-36.43) | -0.16(-0.11--0.21) | -0.12(-0.09--0.16) | -0.26(-0.21--0.31) |
| **Sociodemographic Index** | | | | | | |
| High SDI | Both | 35.53(48.05-23.07) | 29.88(19.06-40.83) | -0.09(-0.07--0.12) | -0.08(-0.05--0.11) | -0.16(-0.12--0.2) |
| High SDI | Female | 35.81(48.61-22.84) | 30.97(19.56-42.57) | -0.06(-0.04--0.08) | -0.08(-0.04--0.13) | -0.14(-0.09--0.18) |
| High SDI | Male | 35.2(47.34-22.97) | 28.79(18.19-39.41) | -0.12(-0.09--0.16) | -0.07(-0.03--0.11) | -0.18(-0.13--0.24) |
| High-middle SDI | Both | 29.43(40.47-18.89) | 21.66(13.32-30.77) | -0.14(-0.08--0.22) | -0.14(-0.1--0.19) | -0.26(-0.19--0.34) |
| High-middle SDI | Female | 29.68(40.76-18.92) | 22.25(13.43-31.89) | -0.13(-0.05--0.22) | -0.14(-0.08--0.2) | -0.25(-0.16--0.35) |
| High-middle SDI | Male | 29.18(40.09-18.61) | 21.08(12.4-30.17) | -0.16(-0.07--0.25) | -0.14(-0.08--0.21) | -0.28(-0.18--0.37) |
| Low SDI | Both | 28.27(38.21-18.44) | 24.01(15.19-32.84) | -0.05(-0.01--0.08) | -0.11(-0.07--0.14) | -0.15(-0.11--0.19) |
| Low SDI | Female | 27.8(37.57-17.95) | 23.57(14.96-32.46) | -0.05(0--0.09) | -0.11(-0.07--0.16) | -0.15(-0.1--0.21) |
| Low SDI | Male | 28.75(38.52-18.66) | 24.46(15.91-33.06) | -0.05(0--0.09) | -0.1(-0.06--0.15) | -0.15(-0.09--0.2) |
| Low-middle SDI | Both | 45.51(55.89-34.65) | 34.48(24.78-44.54) | -0.14(-0.11--0.18) | -0.12(-0.09--0.15) | -0.24(-0.2--0.29) |
| Low-middle SDI | Female | 45.4(55.96-34.17) | 34.51(24.48-44.51) | -0.14(-0.1--0.18) | -0.12(-0.08--0.16) | -0.24(-0.19--0.29) |
| Low-middle SDI | Male | 45.63(55.78-34.8) | 34.46(24.68-43.96) | -0.14(-0.11--0.18) | -0.12(-0.08--0.17) | -0.24(-0.2--0.3) |
| Middle SDI | Both | 41.22(51.82-30.2) | 27.36(18.77-36.39) | -0.23(-0.17--0.28) | -0.14(-0.11--0.18) | -0.34(-0.28--0.4) |
| Middle SDI | Female | 42.09(52.79-30.57) | 27.77(18.98-36.87) | -0.23(-0.17--0.3) | -0.15(-0.1--0.19) | -0.34(-0.27--0.41) |
| Middle SDI | Male | 40.32(51.11-28.84) | 26.92(18.33-35.76) | -0.22(-0.16--0.3) | -0.14(-0.09--0.19) | -0.33(-0.26--0.4) |
| **Region** | | | | | | |
| Andean Latin America | Both | 43.97(28.58-58.25) | 31.12(20.71-41.44) | -0.25(-0.28--0.2) | -0.06(-0.1--0.02) | -0.29(-0.24--0.34) |
| Andean Latin America | Female | 43.99(28.3-58.5) | 30.93(20.5-41.91) | -0.25(-0.29--0.2) | -0.06(-0.12--0.01) | -0.3(-0.23--0.36) |
| Andean Latin America | Male | 43.96(28.88-58.03) | 31.31(20.65-41.69) | -0.24(-0.28--0.2) | -0.06(-0.11-0) | -0.29(-0.23--0.34) |
| Australasia | Both | 42.2(27.45-55.96) | 35.65(22.76-48.87) | -0.04(-0.08--0.02) | -0.12(-0.18--0.06) | -0.16(-0.1--0.23) |
| Australasia | Female | 42.22(27.08-56.48) | 35.76(22.64-48.95) | -0.04(-0.09-0) | -0.11(-0.19--0.04) | -0.15(-0.08--0.24) |
| Australasia | Male | 42.17(27.69-55.84) | 35.48(22.58-48.31) | -0.05(-0.09-0) | -0.12(-0.2--0.05) | -0.16(-0.08--0.25) |
| Caribbean | Both | 37.88(23.81-51.25) | 24.53(14.93-34.52) | -0.35(-0.38--0.32) | -0.01(-0.05-0.03) | -0.35(-0.31--0.4) |
| Caribbean | Female | 37.82(23.75-51.55) | 24.51(14.88-34.68) | -0.35(-0.38--0.31) | -0.01(-0.07-0.05) | -0.35(-0.3--0.41) |
| Caribbean | Male | 37.94(24-51.04) | 24.56(15.35-34.16) | -0.35(-0.38--0.31) | -0.01(-0.07-0.04) | -0.35(-0.3--0.41) |
| Central Asia | Both | 38.14(24.6-51.45) | 26.67(16.09-37.87) | -0.16(-0.2--0.13) | -0.17(-0.22--0.13) | -0.3(-0.24--0.37) |
| Central Asia | Female | 37.98(24.2-51.41) | 26.46(15.77-38.33) | -0.16(-0.21--0.12) | -0.17(-0.23--0.12) | -0.3(-0.23--0.38) |
| Central Asia | Male | 38.36(25.06-51.45) | 26.92(16.3-37.89) | -0.16(-0.2--0.13) | -0.17(-0.23--0.12) | -0.3(-0.24--0.38) |
| Central Europe | Both | 24.38(14.43-35.11) | 22.27(13.33-32.02) | 0.03(0-0.07) | -0.11(-0.15--0.08) | -0.09(-0.04--0.12) |
| Central Europe | Female | 24.2(14.09-34.97) | 22.13(13.11-31.99) | 0.03(0-0.08) | -0.11(-0.16--0.07) | -0.09(-0.02--0.14) |
| Central Europe | Male | 24.59(14.78-35.43) | 22.42(13.63-31.88) | 0.03(-0.01-0.07) | -0.11(-0.16--0.06) | -0.09(-0.03--0.14) |
| Central Latin America | Both | 20.43(13.21-27.94) | 19.33(12.03-27.12) | -0.03(-0.08-0.01) | -0.03(-0.07-0.01) | -0.05(0--0.12) |
| Central Latin America | Female | 20.18(13.01-27.64) | 19.12(11.74-27.23) | -0.02(-0.09-0.02) | -0.03(-0.09-0.03) | -0.05(0.02--0.13) |
| Central Latin America | Male | 20.7(13.43-28.12) | 19.57(12.32-27.25) | -0.03(-0.08-0.01) | -0.03(-0.09-0.03) | -0.05(0.02--0.13) |
| Central Sub-Saharan Africa | Both | 18.78(10.91-27.42) | 27.05(15.95-39.35) | 0.64(0.51-0.82) | -0.12(-0.2--0.07) | 0.44(0.6-0.33) |
| Central Sub-Saharan Africa | Female | 18.36(10.6-27.11) | 26.73(15.4-39.57) | 0.66(0.53-0.87) | -0.13(-0.22--0.06) | 0.46(0.65-0.3) |
| Central Sub-Saharan Africa | Male | 19.24(11.3-28.01) | 27.37(16.14-39.64) | 0.61(0.48-0.79) | -0.12(-0.2--0.04) | 0.42(0.61-0.29) |
| East Asia | Both | 38.61(25.49-51.69) | 21.56(13.08-31.29) | -0.27(-0.39--0.16) | -0.24(-0.32--0.16) | -0.44(-0.32--0.56) |
| East Asia | Female | 39.97(25.73-53.69) | 22.91(13.19-33.63) | -0.26(-0.4--0.11) | -0.23(-0.34--0.12) | -0.43(-0.27--0.58) |
| East Asia | Male | 37.27(24.39-50.6) | 20.25(11.14-30.25) | -0.28(-0.44--0.13) | -0.24(-0.36--0.13) | -0.46(-0.3--0.6) |
| Eastern Europe | Both | 22.9(13.65-33.28) | 23.31(13.75-33.86) | 0.09(0.05-0.12) | -0.06(-0.13-0) | 0.02(0.1--0.06) |
| Eastern Europe | Female | 22.73(13.35-33.23) | 23.13(13.14-34.01) | 0.09(0.04-0.14) | -0.06(-0.16-0.03) | 0.02(0.13--0.1) |
| Eastern Europe | Male | 23.08(13.93-33.11) | 23.53(14.11-33.92) | 0.09(0.04-0.14) | -0.06(-0.16-0.03) | 0.02(0.14--0.09) |
| Eastern Sub-Saharan Africa | Both | 15.51(9.47-22.31) | 11.59(7.54-16.29) | -0.18(-0.21--0.13) | -0.08(-0.12--0.04) | -0.25(-0.18--0.3) |
| Eastern Sub-Saharan Africa | Female | 15.34(9.26-22.3) | 11.36(7.3-16.1) | -0.19(-0.23--0.13) | -0.09(-0.14--0.03) | -0.26(-0.19--0.31) |
| Eastern Sub-Saharan Africa | Male | 15.69(9.71-22.5) | 11.84(7.7-16.75) | -0.18(-0.21--0.13) | -0.08(-0.13--0.03) | -0.25(-0.18--0.3) |
| High-income Asia Pacific | Both | 30.73(19.49-42.08) | 39.57(26.82-51.77) | 0.3(0.22-0.42) | -0.01(-0.06-0.03) | 0.29(0.42-0.2) |
| High-income Asia Pacific | Female | 31.5(20.18-43.25) | 40.99(27.59-53.28) | 0.31(0.21-0.47) | -0.01(-0.06-0.04) | 0.3(0.47-0.18) |
| High-income Asia Pacific | Male | 29.82(18.85-41.09) | 38.11(25.47-50.14) | 0.3(0.2-0.44) | -0.02(-0.08-0.04) | 0.28(0.44-0.17) |
| High-income North America | Both | 39.2(25.7-52.35) | 25.73(15.24-36.02) | -0.26(-0.32--0.22) | -0.11(-0.2--0.05) | -0.34(-0.27--0.43) |
| High-income North America | Female | 39.31(25.49-52.61) | 26.91(16.06-38.36) | -0.21(-0.25--0.17) | -0.13(-0.25--0.04) | -0.32(-0.23--0.42) |
| High-income North America | Male | 39(25.65-51.9) | 24.46(14.35-34.58) | -0.31(-0.39--0.25) | -0.09(-0.21-0.02) | -0.37(-0.27--0.49) |
| North Africa and Middle East | Both | 15.53(9.46-22.42) | 14.08(8.75-20.15) | -0.1(-0.13--0.07) | 0.01(-0.02-0.05) | -0.09(-0.05--0.13) |
| North Africa and Middle East | Female | 15.29(9.21-22.19) | 13.97(8.67-19.91) | -0.1(-0.13--0.06) | 0.01(-0.03-0.06) | -0.09(-0.02--0.14) |
| North Africa and Middle East | Male | 15.78(9.63-22.86) | 14.19(8.85-20.4) | -0.11(-0.13--0.07) | 0(-0.04-0.06) | -0.1(-0.04--0.15) |
| Oceania | Both | 7.37(4.39-11.3) | 4.19(2.43-6.7) | -0.36(-0.44--0.29) | -0.11(-0.22-0.03) | -0.43(-0.32--0.53) |
| Oceania | Female | 7.2(4.24-10.93) | 3.96(2.3-6.41) | -0.38(-0.47--0.3) | -0.11(-0.26-0.08) | -0.45(-0.31--0.57) |
| Oceania | Male | 7.54(4.47-11.57) | 4.41(2.48-7.22) | -0.35(-0.43--0.26) | -0.1(-0.26-0.09) | -0.41(-0.27--0.53) |
| South Asia | Both | 45.13(32.74-56.96) | 33.64(23.25-44.48) | -0.12(-0.15--0.09) | -0.16(-0.2--0.11) | -0.25(-0.21--0.31) |
| South Asia | Female | 45.03(32.28-57.19) | 33.65(23.07-44.82) | -0.12(-0.15--0.09) | -0.15(-0.22--0.09) | -0.25(-0.19--0.32) |
| South Asia | Male | 45.23(32.9-56.83) | 33.63(23.26-44.16) | -0.12(-0.15--0.09) | -0.16(-0.23--0.1) | -0.26(-0.19--0.33) |
| Southeast Asia | Both | 72.66(62.95-80.56) | 57.6(44.05-69.22) | -0.15(-0.23--0.1) | -0.06(-0.09--0.04) | -0.21(-0.14--0.3) |
| Southeast Asia | Female | 73.12(63.28-81.03) | 57.81(44.21-69.62) | -0.16(-0.24--0.1) | -0.06(-0.1--0.04) | -0.21(-0.14--0.3) |
| Southeast Asia | Male | 72.14(62.33-80.08) | 57.35(43.98-68.73) | -0.15(-0.23--0.1) | -0.06(-0.1--0.04) | -0.2(-0.14--0.3) |
| Southern Latin America | Both | 44.81(29.66-59.13) | 38.53(24.42-52.18) | -0.09(-0.13--0.07) | -0.05(-0.1--0.01) | -0.14(-0.09--0.2) |
| Southern Latin America | Female | 44.86(29.2-59.5) | 38.52(24.51-52.06) | -0.1(-0.13--0.07) | -0.05(-0.12-0.01) | -0.14(-0.08--0.22) |
| Southern Latin America | Male | 44.76(29.9-58.8) | 38.54(24.21-51.81) | -0.09(-0.13--0.07) | -0.05(-0.12-0.01) | -0.14(-0.08--0.22) |
| Southern Sub-Saharan Africa | Both | 14.87(8.46-22.15) | 16.01(9.19-24.11) | 0.09(0.04-0.14) | -0.01(-0.1-0.09) | 0.08(0.21--0.04) |
| Southern Sub-Saharan Africa | Female | 14.61(8.13-21.89) | 15.76(8.85-24.32) | 0.09(0.02-0.17) | -0.01(-0.14-0.13) | 0.08(0.27--0.08) |
| Southern Sub-Saharan Africa | Male | 15.17(8.74-22.53) | 16.3(9.18-24.45) | 0.09(0.02-0.16) | -0.01(-0.13-0.12) | 0.07(0.26--0.08) |
| Tropical Latin America | Both | 38.62(25.95-51.14) | 28.27(18.01-39.28) | -0.29(-0.35--0.24) | 0.03(-0.05-0.1) | -0.27(-0.2--0.34) |
| Tropical Latin America | Female | 38.56(25.72-51.15) | 28.18(17.51-39.66) | -0.29(-0.35--0.24) | 0.03(-0.08-0.14) | -0.27(-0.19--0.36) |
| Tropical Latin America | Male | 38.67(26.26-50.91) | 28.37(18.41-39.28) | -0.28(-0.34--0.24) | 0.02(-0.07-0.13) | -0.27(-0.19--0.36) |
| Western Europe | Both | 31.39(19.45-43.66) | 30.16(18.53-42.63) | -0.03(-0.06-0) | -0.01(-0.04-0.02) | -0.04(0--0.08) |
| Western Europe | Female | 31.35(19.01-44) | 30.24(18.23-42.96) | -0.02(-0.07-0.01) | -0.01(-0.05-0.03) | -0.04(0.02--0.1) |
| Western Europe | Male | 31.43(19.72-43.51) | 30.09(18.56-42.08) | -0.03(-0.08-0.01) | -0.01(-0.05-0.04) | -0.04(0.01--0.1) |
| Western Sub-Saharan Africa | Both | 14.85(9.17-21.41) | 6.73(4.34-9.68) | -0.55(-0.58--0.5) | 0(-0.05-0.06) | -0.55(-0.49--0.58) |
| Western Sub-Saharan Africa | Female | 14.76(9.09-21.5) | 6.44(4.12-9.25) | -0.56(-0.59--0.51) | -0.01(-0.08-0.07) | -0.56(-0.51--0.61) |
| Western Sub-Saharan Africa | Male | 14.96(9.3-21.6) | 7.04(4.56-10.2) | -0.54(-0.57--0.49) | 0.02(-0.05-0.1) | -0.53(-0.47--0.57) |

SDI, socio-demographic index; UI, uncertainty interval.
