# Supplementary material for: Dietary Risk-Related Colorectal Cancer Burden: Estimates From 1990 to 2019
Source: Front Nutr. 2021 Aug 24;8:690663. doi: 10.3389/fnut.2021.690663 (PMC8421520; doi:10.3389/fnut.2021.690663)
Supplement: Supplementary file 3 [file Data_Sheet_3.zip › Supplemental tables/Table S7.docx]

**Table S7** Age-standardized summary exposure value of diet high in processed meat attributable to colorectal cancer and annualized rate of changes.

| **Location** | **Sex** | **Age-standardized summary exposure value (SEV) rate (per 100,000) (95% UI)** | | **Annualized rate of change (ARC, %) (95% UI)** | | |
| --- | --- | --- | --- | --- | --- | --- |
|  |  | **1990** | **2019** | **1990-2010** | **2010-2019** | **1990-2019** |
| Global | Both | 30.95(20.8-42.39) | 29.81(19.04-43.32) | -0.01(-0.05-0.03) | -0.02(-0.06-0.01) | -0.04(-0.11-0.04) |
| Global | Female | 32.78(22.52-43.99) | 31.35(20.62-44.97) | -0.02(-0.06-0.03) | -0.02(-0.06-0.01) | -0.04(-0.12-0.03) |
| Global | Male | 28.96(18.97-40.59) | 28.22(17.66-41.8) | 0(-0.04-0.04) | -0.02(-0.06-0.01) | -0.03(-0.1-0.05) |
| **Sociodemographic Index** | | | | | | |
| High SDI | Both | 69.58(47.25-88.24) | 72.29(53.82-87.89) | 0.07(0.01-0.18) | -0.03(-0.05--0.01) | 0.04(-0.01-0.14) |
| High SDI | Female | 73.08(51.05-91.33) | 77.53(59.96-92.41) | 0.08(0.02-0.2) | -0.02(-0.04-0) | 0.06(0.01-0.17) |
| High SDI | Male | 65.87(43.28-85.08) | 67.13(48.07-83.64) | 0.06(0.01-0.15) | -0.04(-0.06--0.02) | 0.02(-0.02-0.11) |
| High-middle SDI | Both | 39.01(29.14-50.76) | 35.4(21.91-51.89) | -0.06(-0.19-0.02) | -0.03(-0.08-0.01) | -0.09(-0.25-0.02) |
| High-middle SDI | Female | 43.13(32.94-54.76) | 39.47(24.73-55.67) | -0.07(-0.19-0.02) | -0.02(-0.07-0.02) | -0.08(-0.25-0.03) |
| High-middle SDI | Male | 34.37(24.71-46.64) | 31.23(18.78-48.17) | -0.06(-0.18-0.02) | -0.04(-0.09-0.01) | -0.09(-0.24-0.02) |
| Low SDI | Both | 21.13(11.2-36.99) | 23.36(12.73-39.75) | 0.08(0.02-0.16) | 0.03(0-0.06) | 0.11(0.04-0.2) |
| Low SDI | Female | 23.21(12.41-39.03) | 26.06(14.64-43.1) | 0.09(0.02-0.19) | 0.03(-0.01-0.07) | 0.12(0.03-0.25) |
| Low SDI | Male | 19.06(9.89-34.94) | 20.59(10.88-36.32) | 0.06(-0.01-0.15) | 0.02(-0.02-0.08) | 0.08(0-0.2) |
| Low-middle SDI | Both | 14.29(7.72-26.38) | 18.34(10.18-31.77) | 0.18(0.09-0.27) | 0.08(0.03-0.13) | 0.28(0.15-0.4) |
| Low-middle SDI | Female | 14.38(8-25.98) | 18.99(10.75-31.68) | 0.21(0.09-0.32) | 0.09(0.03-0.16) | 0.32(0.16-0.48) |
| Low-middle SDI | Male | 14.2(7.44-26.93) | 17.68(9.3-31.69) | 0.16(0.05-0.27) | 0.07(0.02-0.13) | 0.25(0.1-0.39) |
| Middle SDI | Both | 10.96(5.71-21.35) | 16.3(8.36-30.34) | 0.32(0.13-0.44) | 0.13(0.07-0.19) | 0.49(0.26-0.68) |
| Middle SDI | Female | 11.42(5.77-22.23) | 17.46(8.85-32.47) | 0.35(0.14-0.5) | 0.13(0.07-0.22) | 0.53(0.27-0.78) |
| Middle SDI | Male | 10.51(5.49-21.75) | 15.14(7.83-28.62) | 0.29(0.1-0.41) | 0.12(0.07-0.19) | 0.44(0.2-0.63) |
| **Region** | | | | | | |
| Andean Latin America | Both | 9.65(4.88-19.79) | 11.98(5.5-23.83) | 0.15(-0.06-0.3) | 0.08(0.01-0.15) | 0.24(-0.02-0.43) |
| Andean Latin America | Female | 9.71(4.72-19.66) | 12.13(5.46-25.25) | 0.16(-0.07-0.35) | 0.08(-0.01-0.18) | 0.25(-0.03-0.5) |
| Andean Latin America | Male | 9.58(4.79-19.65) | 11.82(5.48-23.02) | 0.15(-0.09-0.33) | 0.08(-0.02-0.18) | 0.23(-0.04-0.48) |
| Australasia | Both | 67.11(42.08-90.06) | 74.26(48.27-96.59) | 0.1(0.04-0.19) | 0.01(-0.01-0.03) | 0.11(0.04-0.21) |
| Australasia | Female | 70.26(43.79-93.82) | 76.02(50-97.98) | 0.07(0.02-0.17) | 0.01(-0.01-0.04) | 0.08(0.02-0.19) |
| Australasia | Male | 63.77(39.09-87.32) | 72.36(46.47-95.41) | 0.13(0.04-0.29) | 0.01(-0.02-0.04) | 0.13(0.04-0.31) |
| Caribbean | Both | 15.01(6.6-31.9) | 16.08(7.25-33.97) | 0.07(-0.01-0.17) | 0(-0.04-0.05) | 0.07(-0.01-0.19) |
| Caribbean | Female | 15.85(6.89-32.51) | 17.06(7.62-35.6) | 0.07(-0.03-0.22) | 0(-0.06-0.07) | 0.08(-0.04-0.25) |
| Caribbean | Male | 14.13(6.12-29.74) | 15.05(6.66-31.42) | 0.06(-0.04-0.19) | 0(-0.05-0.07) | 0.07(-0.04-0.23) |
| Central Asia | Both | 47.63(28.04-69.19) | 49.41(29.81-70.64) | -0.01(-0.08-0.06) | 0.04(0-0.1) | 0.04(-0.04-0.13) |
| Central Asia | Female | 50.92(30.82-72.22) | 53.26(32.12-74.27) | 0(-0.09-0.1) | 0.04(-0.01-0.11) | 0.05(-0.05-0.16) |
| Central Asia | Male | 43.83(25.8-66.07) | 45.14(26.53-67.67) | -0.02(-0.12-0.09) | 0.05(-0.01-0.14) | 0.03(-0.09-0.18) |
| Central Europe | Both | 43.23(21.51-66.8) | 54.89(30.27-78.26) | 0.23(0.09-0.45) | 0.03(0-0.07) | 0.27(0.11-0.54) |
| Central Europe | Female | 48.2(22.6-74.47) | 57.47(29.65-83.06) | 0.15(0.05-0.37) | 0.03(0-0.08) | 0.19(0.06-0.44) |
| Central Europe | Male | 37.95(19.59-61.07) | 52.16(30.78-74.12) | 0.33(0.12-0.62) | 0.03(-0.01-0.09) | 0.37(0.15-0.71) |
| Central Latin America | Both | 18.66(9.21-35.52) | 21.47(10.85-39.13) | 0.11(0.01-0.24) | 0.04(-0.01-0.11) | 0.15(0.03-0.31) |
| Central Latin America | Female | 19.83(10.1-37.29) | 22.68(11.3-40.38) | 0.1(-0.03-0.27) | 0.04(-0.04-0.13) | 0.14(-0.03-0.35) |
| Central Latin America | Male | 17.41(7.99-33.57) | 20.13(9.61-38.47) | 0.11(-0.02-0.29) | 0.04(-0.05-0.15) | 0.16(-0.01-0.39) |
| Central Sub-Saharan Africa | Both | 16.53(3.44-43.23) | 15.74(3.34-43.49) | -0.07(-0.26-0.08) | 0.02(-0.06-0.14) | -0.05(-0.26-0.16) |
| Central Sub-Saharan Africa | Male | 16.53(3.44-43.23) | 15.74(3.34-43.49) | -0.07(-0.26-0.08) | 0.02(-0.06-0.14) | -0.05(-0.26-0.16) |
| East Asia | Both | 8.81(4.07-19.17) | 16.61(7.44-34.03) | 0.54(0.09-0.88) | 0.23(0.11-0.39) | 0.89(0.22-1.48) |
| East Asia | Female | 9.18(4.15-19.61) | 17.99(8.12-35.54) | 0.58(0.12-0.97) | 0.24(0.1-0.47) | 0.96(0.31-1.67) |
| East Asia | Male | 8.45(3.97-19.37) | 15.26(6.59-32.1) | 0.5(0.06-0.87) | 0.21(0.08-0.41) | 0.81(0.16-1.42) |
| Eastern Europe | Both | 78.19(70.15-86.44) | 63.43(40.66-85.03) | -0.1(-0.28-0.03) | -0.1(-0.19--0.03) | -0.19(-0.41--0.01) |
| Eastern Europe | Female | 79.49(72.48-87.05) | 65.17(41.95-86.65) | -0.1(-0.28-0.04) | -0.09(-0.2--0.02) | -0.18(-0.43-0.01) |
| Eastern Europe | Male | 76.38(66.5-85.74) | 61.37(39.3-82.76) | -0.1(-0.29-0.03) | -0.1(-0.2--0.04) | -0.2(-0.41--0.03) |
| Eastern Sub-Saharan Africa | Both | 18.67(8.77-36.62) | 19.84(9.36-38.33) | 0.04(-0.02-0.1) | 0.03(0-0.07) | 0.06(0-0.16) |
| Eastern Sub-Saharan Africa | Female | 19.39(9.05-37.5) | 20.71(9.8-39.99) | 0.04(-0.04-0.13) | 0.03(-0.02-0.09) | 0.07(-0.02-0.2) |
| Eastern Sub-Saharan Africa | Male | 17.91(8.25-35.65) | 18.91(8.64-37.12) | 0.03(-0.04-0.13) | 0.02(-0.02-0.08) | 0.06(-0.03-0.18) |
| High-income Asia Pacific | Both | 59.89(35.56-82.39) | 64.87(40.79-87.01) | 0.17(0.08-0.33) | -0.08(-0.14--0.04) | 0.08(0.02-0.2) |
| High-income Asia Pacific | Female | 67.35(42.81-89.9) | 73.7(49.38-93.93) | 0.16(0.06-0.35) | -0.06(-0.13--0.02) | 0.09(0-0.24) |
| High-income Asia Pacific | Male | 52.17(27.6-76.07) | 56.22(30.49-81.35) | 0.19(0.08-0.39) | -0.1(-0.18--0.04) | 0.08(-0.03-0.23) |
| High-income North America | Both | 76.38(53.07-95.64) | 83.72(65.79-97.62) | 0.11(0.02-0.28) | -0.01(-0.03-0.01) | 0.1(0.02-0.26) |
| High-income North America | Female | 76.69(53.82-95.33) | 84.21(67.48-97.73) | 0.12(0.02-0.31) | -0.02(-0.05-0.01) | 0.1(0.02-0.27) |
| High-income North America | Male | 76.12(51.88-96.07) | 83.22(64.98-97.73) | 0.1(0.02-0.25) | 0(-0.03-0.02) | 0.09(0.01-0.25) |
| North Africa and Middle East | Both | 11.18(4.49-25.33) | 13.44(5.49-30.07) | 0.16(0.05-0.28) | 0.04(-0.01-0.09) | 0.2(0.07-0.36) |
| North Africa and Middle East | Female | 12.18(5.24-25.94) | 15.02(6.68-31.25) | 0.18(0.06-0.37) | 0.04(-0.02-0.13) | 0.23(0.08-0.46) |
| North Africa and Middle East | Male | 10.23(3.78-24.53) | 12(4.17-29.54) | 0.13(0.02-0.25) | 0.04(-0.03-0.09) | 0.17(0.02-0.32) |
| Oceania | Both | 9.68(4.11-20.53) | 9.98(4.33-20.98) | 0.02(-0.08-0.14) | 0.01(-0.07-0.09) | 0.03(-0.09-0.16) |
| Oceania | Female | 10.2(4.34-22.01) | 10.5(4.5-22.3) | 0.02(-0.13-0.2) | 0.01(-0.1-0.13) | 0.03(-0.14-0.26) |
| Oceania | Male | 9.19(3.93-19.26) | 9.47(4.04-20.27) | 0.02(-0.12-0.18) | 0.01(-0.11-0.13) | 0.03(-0.12-0.22) |
| South Asia | Both | 13.77(7.45-26.94) | 17.09(9.24-32.1) | 0.18(0.1-0.29) | 0.05(0.01-0.1) | 0.24(0.14-0.36) |
| South Asia | Female | 13.09(7.04-25.41) | 16.44(8.96-31.15) | 0.2(0.08-0.35) | 0.05(-0.01-0.12) | 0.26(0.11-0.44) |
| South Asia | Male | 14.4(7.66-28.66) | 17.74(9.42-33.65) | 0.17(0.06-0.31) | 0.05(0-0.11) | 0.23(0.11-0.39) |
| Southeast Asia | Both | 7.03(3.44-16.87) | 9.57(4.57-21.41) | 0.2(0.02-0.35) | 0.13(0.04-0.21) | 0.36(0.06-0.6) |
| Southeast Asia | Female | 7.5(3.72-17.69) | 10.47(5.04-22.39) | 0.22(0.01-0.4) | 0.14(0.03-0.23) | 0.4(0.06-0.66) |
| Southeast Asia | Male | 6.52(3.17-16) | 8.66(4.15-20.35) | 0.18(0.03-0.32) | 0.12(0.02-0.21) | 0.33(0.07-0.56) |
| Southern Latin America | Both | 46.67(28.41-67.77) | 59.43(38.19-81.78) | 0.16(0.08-0.28) | 0.09(0.04-0.18) | 0.27(0.13-0.47) |
| Southern Latin America | Female | 49.36(30.5-71.35) | 62.66(40.28-85.56) | 0.16(0.06-0.32) | 0.09(0.03-0.2) | 0.27(0.12-0.5) |
| Southern Latin America | Male | 43.79(26.26-64.4) | 55.98(34.94-78.06) | 0.17(0.07-0.33) | 0.09(0.02-0.19) | 0.28(0.12-0.53) |
| Southern Sub-Saharan Africa | Both | 15.88(6.97-33.08) | 18.8(8.7-37.12) | 0.13(0.04-0.25) | 0.04(-0.01-0.12) | 0.18(0.07-0.36) |
| Southern Sub-Saharan Africa | Female | 16.81(7.39-34.7) | 19.97(9.52-38.75) | 0.14(0.03-0.3) | 0.05(-0.03-0.15) | 0.19(0.05-0.42) |
| Southern Sub-Saharan Africa | Male | 14.83(6.51-31.31) | 17.49(7.78-35.58) | 0.13(0.03-0.29) | 0.04(-0.02-0.14) | 0.18(0.04-0.39) |
| Tropical Latin America | Both | 16.67(7.55-34.56) | 24.43(12.01-44.31) | 0.29(0.14-0.52) | 0.14(0.05-0.28) | 0.47(0.22-0.89) |
| Tropical Latin America | Female | 17.71(7.97-35.59) | 26.24(12.85-46.29) | 0.3(0.12-0.62) | 0.14(0.03-0.32) | 0.48(0.23-1.02) |
| Tropical Latin America | Male | 15.57(6.82-32.67) | 22.47(10.93-41.72) | 0.27(0.1-0.52) | 0.13(0.03-0.3) | 0.44(0.19-0.86) |
| Western Europe | Both | 74.68(51.69-93.9) | 78.23(56.08-96.39) | 0.05(0.03-0.1) | -0.01(-0.01-0) | 0.05(0.02-0.09) |
| Western Europe | Female | 76.34(53.49-95.16) | 79.83(58.5-97.22) | 0.05(0.02-0.1) | 0(-0.02-0) | 0.05(0.02-0.09) |
| Western Europe | Male | 72.82(49.21-92.51) | 76.55(53.89-95.62) | 0.06(0.03-0.1) | -0.01(-0.02-0) | 0.05(0.03-0.1) |
| Western Sub-Saharan Africa | Both | 30.72(15.95-50.76) | 35.69(20.04-57.22) | 0.14(0.05-0.3) | 0.02(-0.03-0.07) | 0.16(0.06-0.34) |
| Western Sub-Saharan Africa | Female | 33.11(17.18-54.43) | 38.72(21.96-59.92) | 0.15(0.03-0.33) | 0.01(-0.05-0.09) | 0.17(0.03-0.38) |
| Western Sub-Saharan Africa | Male | 28.44(14.65-48.28) | 32.34(17.4-53.22) | 0.13(0.01-0.34) | 0.01(-0.05-0.09) | 0.14(0-0.36) |

SDI, socio-demographic index; UI, uncertainty interval.
