# Supplementary material for: Dietary Risk-Related Colorectal Cancer Burden: Estimates From 1990 to 2019
Source: Front Nutr. 2021 Aug 24;8:690663. doi: 10.3389/fnut.2021.690663 (PMC8421520; doi:10.3389/fnut.2021.690663)
Supplement: Supplementary file 3 [file Data_Sheet_3.zip › Supplemental tables/Table S8.docx]

**Table S8** Global disease burden of dietary risk factors related colorectal cancer

| **Dietary risk factors** | **Sex** | **Deaths** | | **Percent (%)** | | **DALYs (No.×1000)** | | **Percent (%)** | |
| --- | --- | --- | --- | --- | --- | --- | --- | --- | --- |
|  |  | **1990** | **2019** | **1990** | **2019** | **1990** | **2019** | **1990** | **2019** |
| All factors | Both | 278251.82 | 583076.99 | 100.00 | 100.00 | 6703.23 | 13169.18 | 100.00 | 100.00 |
|  | Female | 136942.48 | 257500.59 | 100.00 | 100.00 | 3141.23 | 5457.52 | 100.00 | 100.00 |
|  | Male | 141309.33 | 325576.37 | 100.00 | 100.00 | 3561.99 | 7711.65 | 100.00 | 100.00 |
| Diet low in whole grains | Both | 83524.19 | 171487.29 | 30.02 | 29.41 | 1974.78 | 3806.86 | 29.46 | 28.91 |
|  | Female | 41692.40 | 76531.02 | 30.45 | 29.72 | 936.39 | 1592.83 | 29.81 | 29.19 |
|  | Male | 41831.79 | 94956.26 | 29.60 | 29.17 | 1038.39 | 2214.03 | 29.15 | 28.71 |
| Diet low in fiber | Both | 12547.51 | 20498.80 | 4.51 | 3.52 | 296.44 | 448.70 | 4.42 | 3.41 |
|  | Female | 6422.12 | 9742.16 | 4.69 | 3.78 | 143.01 | 197.33 | 4.55 | 3.62 |
|  | Male | 6125.39 | 10756.64 | 4.33 | 3.30 | 153.43 | 251.36 | 4.31 | 3.26 |
| Diet high in red meat | Both | 26087.14 | 52811.03 | 9.38 | 9.06 | 627.83 | 1234.68 | 9.37 | 9.38 |
|  | Female | 12951.40 | 22759.72 | 9.46 | 8.84 | 296.27 | 496.40 | 9.43 | 9.10 |
|  | Male | 13135.74 | 30051.31 | 9.30 | 9.23 | 331.56 | 738.28 | 9.31 | 9.57 |
| Diet high in processed meat | Both | 20185.18 | 33927.57 | 7.25 | 5.82 | 462.27 | 735.04 | 6.90 | 5.58 |
|  | Female | 10612.48 | 16191.08 | 7.75 | 6.29 | 230.27 | 329.55 | 7.33 | 6.04 |
|  | Male | 9572.69 | 17736.48 | 6.77 | 5.45 | 232.00 | 405.50 | 6.51 | 5.26 |
| Diet low in milk | Both | 72199.11 | 166456.36 | 25.95 | 28.55 | 1764.17 | 3799.30 | 26.32 | 28.85 |
|  | Female | 35728.96 | 74359.78 | 26.09 | 28.88 | 833.68 | 1597.63 | 26.54 | 29.27 |
|  | Male | 36470.15 | 92096.57 | 25.81 | 28.29 | 930.49 | 2201.66 | 26.12 | 28.55 |
| Diet low in calcium | Both | 63708.69 | 137895.94 | 22.90 | 23.65 | 1577.74 | 3144.60 | 23.54 | 23.88 |
|  | Female | 29535.12 | 57916.83 | 21.57 | 22.49 | 701.61 | 1243.78 | 22.34 | 22.79 |
|  | Male | 34173.57 | 79979.11 | 24.18 | 24.57 | 876.12 | 1900.82 | 24.60 | 24.65 |

DALYs, disability-adjusted life-years
