# Supplementary material for: Dietary Risk-Related Colorectal Cancer Burden: Estimates From 1990 to 2019
Source: Front Nutr. 2021 Aug 24;8:690663. doi: 10.3389/fnut.2021.690663 (PMC8421520; doi:10.3389/fnut.2021.690663)
Supplement: Supplementary file 3 [file Data_Sheet_3.zip › Supplemental tables/Table S9.docx]

**Table S9** Deaths, ASDRs and change trends of colorectal cancer attributable to diet low in whole grains between 1990 and 2019 by SDI, regions and sex.

| **Location** | **Sex** | **Deaths (95%UI)** | | **ASDR (95%UI)** | | **EAPC (95%CI)** |
| --- | --- | --- | --- | --- | --- | --- |
|  |  | **1990** | **2019** | **1990** | **2019** | **1990-2019** |
| Global | Both | 83524.19(33127.53-108898.15) | 171487.29(66721.33-225161.82) | 2.31(0.92-3) | 2.16(0.84-2.84) | -0.29(-0.36--0.22) |
| Global | Female | 41692.4(16597.11-53739.78) | 76531.02(29653.23-100395.3) | 2.07(0.82-2.66) | 1.75(0.68-2.29) | -0.7(-0.78--0.62) |
| Global | Male | 41831.79(16444.91-54511.04) | 94956.26(36396.04-125388.47) | 2.64(1.04-3.43) | 2.66(1.02-3.51) | 0.02(-0.05-0.1) |
| **Sociodemographic Index** | | | | | | |
| High SDI | Both | 33880.21(12819.82-44759.95) | 51307.18(19330.43-67629.65) | 3.23(1.22-4.26) | 2.55(0.96-3.35) | -0.97(-1.06--0.88) |
| High SDI | Female | 17002.15(6366.86-22516.13) | 23898.53(9049.83-31734.28) | 2.68(1-3.55) | 2.04(0.77-2.69) | -1.13(-1.23--1.02) |
| High SDI | Male | 16878.06(6381.92-22281.58) | 27408.65(10290.23-35748.25) | 4.02(1.52-5.31) | 3.18(1.19-4.14) | -0.98(-1.05--0.91) |
| High-middle SDI | Both | 29558.8(12446.88-37444.16) | 55270.38(21848.52-72006.25) | 2.93(1.23-3.7) | 2.75(1.09-3.58) | -0.34(-0.43--0.24) |
| High-middle SDI | Female | 15099.7(6396.82-19045.2) | 24373.27(9867.5-31738.13) | 2.57(1.09-3.24) | 2.12(0.86-2.76) | -0.85(-0.95--0.76) |
| High-middle SDI | Male | 14459.1(6061.52-18297.42) | 30897.11(12066.9-41010.89) | 3.49(1.46-4.42) | 3.6(1.41-4.74) | 0.06(-0.05-0.17) |
| Low SDI | Both | 2142.81(871.3-2938.42) | 5528.37(2194.9-7384.17) | 1(0.41-1.36) | 1.17(0.46-1.56) | 0.56(0.51-0.6) |
| Low SDI | Female | 955.09(390.69-1399.99) | 2622.37(1033.74-3520.1) | 0.9(0.37-1.31) | 1.08(0.42-1.44) | 0.66(0.59-0.73) |
| Low SDI | Male | 1187.72(480.83-1700.29) | 2905.99(1151.46-3925.79) | 1.1(0.44-1.57) | 1.27(0.5-1.72) | 0.5(0.47-0.53) |
| Low-middle SDI | Both | 5391.83(2145.04-7140) | 17246.37(6672.2-22951.46) | 0.99(0.39-1.31) | 1.34(0.52-1.79) | 1.08(1.03-1.12) |
| Low-middle SDI | Female | 2579.96(1036.65-3514.31) | 8203.67(3171.21-11192.06) | 0.95(0.38-1.28) | 1.22(0.47-1.66) | 0.83(0.77-0.89) |
| Low-middle SDI | Male | 2811.87(1113.94-3900.14) | 9042.7(3455.26-11999.04) | 1.02(0.4-1.4) | 1.48(0.57-1.97) | 1.33(1.27-1.38) |
| Middle SDI | Both | 12505.81(4819.89-16724.18) | 42038.48(16054.5-56386.37) | 1.32(0.51-1.77) | 1.8(0.69-2.41) | 1.24(1.09-1.4) |
| Middle SDI | Female | 6032.74(2314.41-8022.22) | 17386.77(6684.28-23709.9) | 1.23(0.47-1.63) | 1.41(0.54-1.92) | 0.55(0.44-0.66) |
| Middle SDI | Male | 6473.07(2478.73-8735.99) | 24651.72(9417.6-33675.61) | 1.43(0.55-1.92) | 2.23(0.86-3.03) | 1.83(1.63-2.02) |
| **Region** | | | | | | |
| Africa | Both | 2867.14(1137.16-3812.82) | 7610.18(2990.62-10320.25) | 1.11(0.44-1.48) | 1.35(0.53-1.82) | 0.73(0.69-0.77) |
| Africa | Female | 1352.6(548.74-1848.8) | 3609.54(1405.72-4979.69) | 1.04(1.4-0.42) | 1.24(0.48-1.7) | 0.72(0.67-0.77) |
| Africa | Male | 1514.54(610.29-2078.94) | 4000.64(1583.41-5373.67) | 1.19(0.48-1.61) | 1.48(0.58-1.97) | 0.76(0.72-0.8) |
| America | Both | 15404.44(5860.18-20268.54) | 26793.09(9978.98-35494.86) | 2.56(3.37-0.98) | 2.08(0.78-2.76) | -0.84(-0.9--0.77) |
| America | Female | 7794.32(2958.23-10264.69) | 12954.19(4849.17-17288.29) | 2.26(0.86-2.97) | 1.79(0.67-2.4) | -0.89(-0.95--0.83) |
| America | Male | 7610.13(2888.81-9984.77) | 13838.9(5137.65-18266.83) | 2.96(1.13-3.88) | 2.42(0.9-3.2) | -0.83(-0.91--0.75) |
| Asia | Both | 27269.83(10549.4-36410.15) | 85227.64(32678.8-113589.14) | 1.51(0.58-2) | 1.89(0.73-2.53) | 0.89(0.75-1.04) |
| Asia | Female | 12725.62(4861.6-16933.53) | 35517.83(13629.2-48153.23) | 1.35(0.52-1.8) | 1.48(0.57-2) | 0.32(0.21-0.42) |
| Asia | Male | 14544.21(5521.9-19595.36) | 49709.81(19023.98-67397.09) | 1.7(0.65-2.29) | 2.38(0.91-3.19) | 1.35(1.18-1.53) |
| Europe | Both | 37849.56(15428.62-48102.89) | 51553.81(20438.73-66290.78) | 3.67(1.5-4.67) | 3.18(1.26-4.08) | -0.74(-0.86--0.61) |
| Europe | Female | 19756.21(8128.19-25143.97) | 24308.12(9680.87-31706.15) | 3.11(1.28-3.95) | 2.5(1-3.27) | -1.02(-1.15--0.89) |
| Europe | Male | 18093.35(7361.55-23001.64) | 27245.69(10750.62-34875.62) | 4.61(1.88-5.87) | 4.11(1.63-5.26) | -0.6(-0.72--0.48) |
| Andean Latin America | Both | 238.53(90.76-313.86) | 848.89(311.32-1207.36) | 1.24(0.47-1.63) | 1.56(0.57-2.22) | 0.99(0.84-1.13) |
| Andean Latin America | Female | 128.75(49.93-171.55) | 457.15(169.13-649.48) | 1.31(0.51-1.75) | 1.6(0.59-2.27) | 0.75(0.61-0.89) |
| Andean Latin America | Male | 109.78(41.46-146.91) | 391.74(143.75-558.72) | 1.17(0.44-1.56) | 1.51(0.55-2.15) | 1.26(1.09-1.43) |
| Australasia | Both | 827.51(311.62-1109.57) | 1283.71(478.33-1718.05) | 3.58(1.35-4.81) | 2.49(0.93-3.31) | -1.59(-1.73--1.46) |
| Australasia | Female | 394.87(147.28-533.3) | 594.26(225.11-800.9) | 3.02(1.12-4.08) | 2.08(0.79-2.78) | -1.57(-1.69--1.45) |
| Australasia | Male | 432.64(163.38-574.3) | 689.46(252.42-922.97) | 4.32(1.64-5.74) | 2.96(1.09-3.96) | -1.69(-1.86--1.53) |
| Caribbean | Both | 536.68(202.92-695.7) | 1261.77(473.54-1729.73) | 2.15(0.81-2.79) | 2.44(0.92-3.34) | 0.46(0.4-0.53) |
| Caribbean | Female | 280.33(106.19-363.13) | 647.1(241.77-905.55) | 2.15(0.82-2.79) | 2.3(0.86-3.22) | 0.24(0.19-0.29) |
| Caribbean | Male | 256.34(97.29-329.46) | 614.67(232.37-833.16) | 2.14(0.81-2.75) | 2.59(0.98-3.49) | 0.7(0.61-0.78) |
| Central Asia | Both | 1084.14(536.52-1330.05) | 1593.65(786.23-1991.67) | 2.33(1.16-2.86) | 2.4(1.19-3.01) | 0.36(0.18-0.54) |
| Central Asia | Female | 563.47(279.46-695.36) | 791.7(389.92-996.47) | 2.05(1.02-2.53) | 2.08(1.03-2.62) | 0.3(0.15-0.46) |
| Central Asia | Male | 520.67(255.84-640.37) | 801.95(392.5-1009.14) | 2.76(1.36-3.39) | 2.84(1.38-3.59) | 0.38(0.16-0.59) |
| Central Europe | Both | 5238.07(2043.1-6716.74) | 8363.4(3077.36-11204.69) | 3.66(1.43-4.7) | 3.82(1.41-5.12) | 0.14(0.05-0.23) |
| Central Europe | Female | 2498.56(980.65-3194.86) | 3589.41(1341.4-4838.69) | 3(1.17-3.84) | 2.76(1.03-3.72) | -0.38(-0.47--0.29) |
| Central Europe | Male | 2739.51(1066.04-3529.42) | 4773.98(1762.04-6383.66) | 4.58(1.78-5.91) | 5.3(1.96-7.09) | 0.57(0.46-0.68) |
| Central Latin America | Both | 776.37(292.23-1043.75) | 3053.23(1125.92-4242.13) | 1(0.38-1.34) | 1.32(0.49-1.83) | 0.95(0.91-0.99) |
| Central Latin America | Female | 413.31(155.86-559.78) | 1500.75(554.44-2122.46) | 1.03(0.39-1.4) | 1.2(0.44-1.69) | 0.5(0.45-0.54) |
| Central Latin America | Male | 363.06(135.13-483.82) | 1552.49(565.41-2171.1) | 0.96(0.36-1.28) | 1.46(0.53-2.04) | 1.44(1.38-1.5) |
| Central Sub-Saharan Africa | Both | 218.15(83.36-316.83) | 524.97(187.72-771.98) | 1.09(0.41-1.58) | 1.11(0.39-1.66) | -0.05(-0.27-0.17) |
| Central Sub-Saharan Africa | Female | 95.74(37.08-143.46) | 247.38(84.98-372.12) | 0.91(0.36-1.34) | 0.94(0.33-1.45) | 0.05(-0.14-0.23) |
| Central Sub-Saharan Africa | Male | 122.41(46.17-186.74) | 277.59(97.71-444.12) | 1.29(0.48-2.02) | 1.33(0.47-2.2) | -0.02(-0.25-0.22) |
| East Asia | Both | 12911.39(4832.13-17333.44) | 43888.79(16195.5-60896.41) | 1.6(0.6-2.15) | 2.24(0.83-3.09) | 1.47(1.21-1.74) |
| East Asia | Female | 6005.34(2214.8-8136.63) | 16141.89(6011.91-22582.79) | 1.42(0.52-1.92) | 1.54(0.58-2.16) | 0.42(0.2-0.63) |
| East Asia | Male | 6906.05(2538.4-9400.47) | 27746.9(10216.84-38988.49) | 1.87(0.7-2.53) | 3.14(1.16-4.35) | 2.26(1.97-2.56) |
| Eastern Europe | Both | 9547.76(4160-11960.93) | 10871.32(4121.46-14232.24) | 3.45(1.5-4.31) | 3.13(1.19-4.1) | -0.8(-1.01--0.59) |
| Eastern Europe | Female | 5467.11(2384.27-6858.09) | 5699.31(2198.25-7713.02) | 3(1.31-3.77) | 2.56(0.99-3.47) | -1.01(-1.2--0.83) |
| Eastern Europe | Male | 4080.64(1764.95-5138.45) | 5172.02(1952.83-6931.68) | 4.44(1.92-5.58) | 4.15(1.57-5.56) | -0.72(-0.96--0.49) |
| Eastern Sub-Saharan Africa | Both | 722.37(277.24-1001.22) | 1860.02(678.37-2622.17) | 1.04(0.4-1.43) | 1.24(0.46-1.74) | 0.66(0.6-0.73) |
| Eastern Sub-Saharan Africa | Female | 324.7(126.46-480.34) | 874.11(326.15-1227.81) | 0.91(0.35-1.35) | 1.11(0.41-1.54) | 0.71(0.63-0.79) |
| Eastern Sub-Saharan Africa | Male | 397.67(152.51-595.44) | 985.91(353.52-1425.58) | 1.17(0.45-1.7) | 1.41(0.5-2.02) | 0.68(0.62-0.73) |
| High-income Asia Pacific | Both | 4617.08(1723.89-6391.22) | 11244.17(4217.15-15105.44) | 2.42(0.91-3.34) | 2.24(0.84-2.99) | -0.33(-0.42--0.24) |
| High-income Asia Pacific | Female | 2110.95(787.21-2937) | 5261.57(1975.32-7252.1) | 1.91(0.71-2.66) | 1.69(0.63-2.29) | -0.51(-0.57--0.45) |
| High-income Asia Pacific | Male | 2506.13(928.43-3447.54) | 5982.59(2266.07-7960.94) | 3.15(1.17-4.33) | 2.9(1.1-3.86) | -0.36(-0.48--0.24) |
| High-income North America | Both | 10993.07(4133.62-14609.35) | 14816.02(5557.28-19541.74) | 3.06(1.15-4.06) | 2.31(0.87-3.05) | -1.17(-1.27--1.08) |
| High-income North America | Female | 5549.98(2065.86-7391.01) | 7010.27(2653.17-9279.18) | 2.57(0.96-3.43) | 1.93(0.73-2.55) | -1.18(-1.26--1.09) |
| High-income North America | Male | 5443.08(2036.4-7189.92) | 7805.76(2927.11-10273.91) | 3.75(1.41-4.94) | 2.77(1.04-3.65) | -1.29(-1.42--1.17) |
| North Africa and Middle East | Both | 2616.58(1224.83-3420.36) | 7821.57(3657.14-10099.97) | 1.66(0.79-2.16) | 1.98(0.92-2.54) | 0.8(0.59-1.01) |
| North Africa and Middle East | Female | 1254.3(591.73-1672.65) | 3566.43(1667.56-4652.07) | 1.6(0.78-2.12) | 1.85(0.87-2.4) | 0.67(0.47-0.88) |
| North Africa and Middle East | Male | 1362.27(654.44-1884.76) | 4255.14(1963.87-5476.75) | 1.71(0.83-2.32) | 2.1(0.97-2.7) | 0.92(0.71-1.13) |
| Oceania | Both | 34.33(12.89-47.06) | 91.7(34.55-128.54) | 1.3(0.5-1.77) | 1.48(0.55-2.04) | 0.41(0.33-0.49) |
| Oceania | Female | 15.52(6.03-21.93) | 41.21(15.43-57.64) | 1.22(0.48-1.72) | 1.37(0.51-1.89) | 0.39(0.3-0.48) |
| Oceania | Male | 18.81(7.23-27.01) | 50.5(18.81-72.07) | 1.39(0.52-1.98) | 1.59(0.59-2.23) | 0.44(0.36-0.51) |
| South Asia | Both | 4010.86(1588.78-5431.08) | 13844.56(5413.1-18841.75) | 0.8(0.32-1.08) | 1.07(0.42-1.45) | 0.84(0.72-0.96) |
| South Asia | Female | 1834.09(737.78-2599.24) | 6860.29(2643.48-9589.47) | 0.76(0.31-1.08) | 1.03(0.4-1.45) | 0.88(0.73-1.04) |
| South Asia | Male | 2176.77(862.3-3003.44) | 6984.27(2744.08-9790.78) | 0.84(0.34-1.16) | 1.1(0.43-1.54) | 0.82(0.72-0.93) |
| Southeast Asia | Both | 3056.03(1159.95-4288.48) | 10359.09(3942.08-15323.74) | 1.28(0.49-1.78) | 1.82(0.69-2.69) | 1.13(1.06-1.2) |
| Southeast Asia | Female | 1465.57(547.24-2093.83) | 4504.19(1743.34-6921.83) | 1.15(0.43-1.61) | 1.46(0.56-2.24) | 0.74(0.66-0.83) |
| Southeast Asia | Male | 1590.45(609.69-2248.67) | 5854.89(2183.83-8628.23) | 1.43(0.55-2.03) | 2.25(0.84-3.33) | 1.49(1.43-1.55) |
| Southern Latin America | Both | 1688.71(711.19-2140.84) | 3089.37(1147.48-3993.9) | 3.82(1.6-4.85) | 3.65(1.36-4.72) | -0.18(-0.27--0.09) |
| Southern Latin America | Female | 806.57(332.24-1030.44) | 1488.08(549.29-1940.58) | 3.22(1.33-4.12) | 3.01(1.11-3.95) | -0.29(-0.39--0.19) |
| Southern Latin America | Male | 882.14(374.62-1120.96) | 1601.29(605.48-2074.37) | 4.58(1.96-5.82) | 4.49(1.7-5.82) | -0.06(-0.15-0.02) |
| Southern Sub-Saharan Africa | Both | 316.96(120.38-470.23) | 751.6(275.52-1076.1) | 1.25(0.47-1.87) | 1.45(0.54-2.09) | 0.53(0.31-0.76) |
| Southern Sub-Saharan Africa | Female | 159.34(61.78-237.68) | 367.58(136.49-539.05) | 1.1(0.42-1.66) | 1.21(0.45-1.77) | 0.45(0.3-0.6) |
| Southern Sub-Saharan Africa | Male | 157.62(57.45-239.28) | 384.02(143.29-549.51) | 1.44(0.52-2.22) | 1.81(0.68-2.59) | 0.73(0.41-1.05) |
| Tropical Latin America | Both | 1276.5(475.97-1683.01) | 3969.32(1478.6-5327.72) | 1.53(0.57-2.01) | 1.67(0.62-2.25) | 0.37(0.22-0.52) |
| Tropical Latin America | Female | 665.53(253.95-877.4) | 1966.83(730.32-2688.62) | 1.49(0.57-1.97) | 1.48(0.55-2.03) | 0.01(-0.14-0.17) |
| Tropical Latin America | Male | 610.97(225.85-806.46) | 2002.49(747.22-2672.64) | 1.56(0.58-2.06) | 1.91(0.71-2.54) | 0.78(0.63-0.93) |
| Western Europe | Both | 21985.93(8795.01-28247.37) | 29830.79(12050.76-38310.03) | 3.74(1.49-4.8) | 2.99(1.2-3.83) | -0.97(-1.14--0.81) |
| Western Europe | Female | 11278.71(4501.05-14445.43) | 13906.23(5630.62-18027.61) | 3.12(1.24-4) | 2.36(0.95-3.05) | -1.21(-1.38--1.03) |
| Western Europe | Male | 10707.22(4288.41-13648.08) | 15924.56(6402.14-20272.87) | 4.67(1.87-5.97) | 3.8(1.52-4.83) | -0.9(-1.05--0.75) |
| Western Sub-Saharan Africa | Both | 827.19(322.44-1163.7) | 2119.36(800.8-2911.1) | 1.06(0.41-1.49) | 1.3(0.49-1.78) | 0.91(0.81-1.01) |
| Western Sub-Saharan Africa | Female | 379.66(147.97-550.76) | 1015.3(377.1-1414.87) | 0.97(0.38-1.4) | 1.21(0.46-1.69) | 0.98(0.86-1.1) |
| Western Sub-Saharan Africa | Male | 447.53(171.15-650.22) | 1104.06(415.7-1534.71) | 1.14(0.44-1.64) | 1.4(0.53-1.94) | 0.89(0.8-0.98) |

ASDR, age-standardized death rate, SDI, socio-demographic index; UI, uncertainty interval.
