# Supplementary material for: Dietary Risk-Related Colorectal Cancer Burden: Estimates From 1990 to 2019
Source: Front Nutr. 2021 Aug 24;8:690663. doi: 10.3389/fnut.2021.690663 (PMC8421520; doi:10.3389/fnut.2021.690663)
Supplement: Supplementary file 3 [file Data_Sheet_3.zip › Supplemental tables/Appendix 2.docx]

**GBD Overview**

The Global Burden of Disease (GBD) is an approach to global descriptive epidemiology. It is a systematic, scientific effort to quantify the comparative magnitude of health loss due to diseases, injuries, and risk factors by age, sex, and geographies for specific points in time. IHME serves as the coordinating center for the GBD and affiliated projects.

Incidence data is obtained from individual cancer registries or aggregated databases of cancer registries, such as CI5 (cancer incidence of five continents), SEER, EUERG or NORDCAN. GBD study relies on a lot of data – over 90,000 data sources. GBD produces regular estimates of all‐cause mortality, deaths by cause, years of life lost due to premature mortality (YLLs), years lived with disability (YLDs), and disability‐adjusted life years (DALYs) for a cause list. The cause list is agreed upon annually by the Scientific Council. The critical milestones for ongoing estimation include regular updates to the GBD estimates, referred to as the “GBD round.” For each round, the entire time series back to 1990 is re‐estimated using all available data to ensure the most complete and highly comparable set of estimates possible. Previous results will be archived every time new results are released. The GBD provides cutting‐edge and timely results through scientific papers, policy reports, web content, and interactive visualizations.

We estimated numbers and rates of incidence, prevalence, deaths, and disability-adjusted life-years (DALYs) for the years 1990–2019.

GBD 2019 provides an independent estimation of population, for each of 204 countries and territories and the globe, using a standardized, replicable approach, as well as a comprehensive update on fertility. GBD 2019 incorporates major data additions and improvements, and methodological refinements. Mortality and life expectancy estimates have been extended back to 1950, and new causes have been added to the fatal and non-fatal cause lists, for a total of 369 diseases and injuries (http://ghdx.healthdata.org/GBD-resultstool).

Results from GBD 2019 are available through an interactive data downloading tool on the Global Health Data Exchange (GHDx). The GHDx is the world’s most comprehensive catalogue of surveys, censuses, vital statistics, and other health-related data. Results are measured in terabytes.

**Definition of indicator**

The GBD cause list is organized in a hierarchy. Levels 1 represent all garbage codes for which a Level 1 GBD cause cannot be directly assigned. Level 2 represent all garbage codes that can be assigned to Level 1 causes in the GBD cause list. Level 3 includes all garbage codes for which we know the Level 2 CoD and can redistribute onto Level 3 causes. Level 4 includes all garbage codes for underlying causes of death that can be redistributed within a Level 3 cause. In this publication, estimates for the GBD cancer groups, for both sexes, for the time from 1980 to 2019, and for the 5-year GBD age groups (0-5; 5-9; etc. until 95+) are presented for 204 countries or territories.

**Data Search List**

| Measure | Death, DALYs (Disability-Adjusted Life Years), years lived with disability (YLDs), years of life lost (YLLs) |
| --- | --- |
| Location (World region) | Asia, America, Africa, Europe |
| Location （Region） | Andean Latin America, Australasia, Caribbean, Central Asia, Central Europe, Central Latin America, Central Sub-Saharan Africa, East Asia, Eastern Europe, Eastern Sub-Saharan Africa, High-income Asia Pacific, High-income North America, North Africa and Middle East, Oceania, South Asia, Southeast Asia, Southern Latin America, Southern Sub-Saharan Africa, Tropical Latin America, Western Europe, Western Sub-Saharan Africa |
| Location （SDI） | High SDI, high-middle SDI, middle SDI, low-middle SDI, and low SDI |
| Location | Countries and territories |
| Sex | Male, female, both |
| Age | 25-29, 30-34, 35-39, 40-44, 45-49, 50-54, 55-59, 60-64, 65-70, 70-74, 75-79, 80 plus |
| Year | 1990,1991,1992,1993,1994,1995,1996,1997,1998,1999,2000,2001,2002,2003,2004,2005,2006,2007,2008,2009,2010,2011,2012,2013,2014,2015,2016,2017,2018,2019 |
| Metric | Number, Rate |
| Context | Cause, risk |
| Cause | Colon and rectum cancer |
| Risk | diet low in fiber, diet high in processed meat, diet high in red meat, diet low in calcium, diet low in milk, and diet low in whole grains |
| Year Range | 1990-2019 |
